# Optical Spectra of Oligofurans: A Theoretical Approach to the Transition Energies, Reorganization Energies, and the Vibronic Activity

**DOI:** 10.3390/molecules26237163

**Published:** 2021-11-26

**Authors:** Karolina Filipowska, Marek T. Pawlikowski, Marcin Andrzejak

**Affiliations:** K. Gumiński Department of Theoretical Chemistry, Jagiellonian University, ul. Gronostajowa 2, 30-387 Kraków, Poland; kaef195@gmail.com (K.F.); pawlikow@chemia.uj.edu.pl (M.T.P.)

**Keywords:** time-dependent DFT (TDDFT), CC2, ultraviolet and visible range (UV/VIS) spectra, vibronic coupling, huang-rhys (HR) factors

## Abstract

There is experimental evidence of high vibronic activity that accompanies the allowed transition between the ground state and the lowest electronic singlet excited state of oligofurans that contain two, three, and four furan rings. The absorption and emission spectra of the three lowest oligofurans measured at liquid nitrogen temperature show distinct fine structures that are reproduced using the projection-based model of vibronic coupling (with Dushinsky rotation included) parameterized utilizing either Density Functional Theory (DFT, with several different exchange-correlation functionals) or ab initio (CC2) quantum chemistry calculations. Using as a reference the experimental data concerning the electronic absorption and fluorescence for the eight lowest oligofurans, we first analyzed the performance of the exchange-correlation functionals for the electronic transition energies and the reorganization energies. Subsequently, we used the best functionals alongside with the CC2 method to explore how the reorganization energies are distributed among the totally symmetric vibrations, identify the normal modes that dominate in the fine structures present in the absorption and emission bands, and trace their evolution with the increasing number of rings in the oligofuran series. Confrontation of the simulated spectra with the experiment allows for the verification of the performance of the selected DFT functionals and the CC2 method.

## 1. Introduction

Oligofurans belong to the class of organic chromophores that, owing to the accessibility of the low-energy excited states and their high processability, are viewed as potential building blocks of organic electronic devices, such as light-emitting diodes or field effect transistors. Nowadays, the chromophores that are most commonly used for this purpose are based on oligoacenes, oligothiophenes, and their derivatives [1,2,3,4,5,6,7,8,9,10,11,12,13]. Recent experimental evidence suggests, however, that oligofurans may also be promising materials, owing to their higher rigidity and better solubility compared to the thiophene-based compounds, as well as their high fluorescence quantum yield [14,15,16,17,18]. Furan also has the advantage that it can be obtained entirely from renewable resources [19,20,21,22]. Moreover, furan and many of its derivatives are biodegradable [23,24,25]. It is believed that the replacing the highly polarizable sulfur atoms by oxygen is less advantageous for charge mobilities and explains the lower conductivity of oligofurans with respect to oligothiophenes [26]. It has been shown by Gidron et al. [27], however, that the tighter packing of the oligofuran films can overcompensate the smaller size and less diffuse nature of the oxygen orbitals, leading to charge mobilities that can surpass those observed for the oligothiophenes. This has opened the way to the development of new, environmentally friendly organic semiconductors.

Despite these encouraging reports, the number of both experimental and theoretical studies concerning excited states of oligofurans is still rather limited in comparison with the thoroughly studied oligothiophenes. One of the prominent experimental papers that gives an account of the photophysical properties of the lowest excited singlet state for the three smallest members of the oligofuran series (bifuran—2O, tertfuran—3O, and quaterfuran—4O) comes from the group of Becker [28] and provides detailed information on energetics and intensities of the transitions between the S_0_ and S_1_ states of the studied oligofurans, as well as the absorption and emission spectra measured at liquid nitrogen temperatures (77 K). The spectra display distinct fine structures for both absorption and emission, which indicate considerable vibronic coupling associated with the S_0_–S_1_ electronic transitions. Experimental UV/VIS spectra of higher oligothiophenes (5O–9O) were also reported [16,29], but owing to their low resolution, only some distortions of the band shape (absorption) or a single, effective vibronic progression (fluorescence) can be discerned. Nevertheless, they provide information on the vertical transition energies that we will use as a reference to select the optimum approximate version of the DFT exchange-correlation functional for studying the excited states of oligofurans in general, and for modeling the spectral vibronic activities in particular.

The vibronic activity is brought about by coupling of the nuclear and electronic degrees of freedom. This in turn occurs due to geometry changes induced by the electronic transitions. The primary effect—linear in the Taylor expansion—is the shift of the minimum of the potential energy surface (PES) for the nuclear motion. Other effects, such as changes of vibrational frequencies or normal mode mixing (Dushinsky rotations), are more subtle and often pass unnoticed unless the spectra display sufficiently high resolution (such as those obtained using the supersonic jet expansion or when recorded in the Shpolsky matrices cooled in liquid helium). In this study, we aim to calculate the global reorganization energies associated with the investigated electronic transitions, reproduce the available experimental absorption and emission spectra of oligofurans, and identify the normal modes that contribute to the observed vibronic structures. To achieve these goals, we employ the density functional theory (DFT) and its time-dependent variant (TDDFT) using several representative exchange-correlation functionals, as well as the ab initio approach using the approximate coupled clusters singles and doubles model (CC2) [30,31,32] that has already proven successful in similar applications [33,34,35,36,37,38] and can currently be regarded as the method of choice for theoretical analysis of spectroscopic properties of medium-sized (containing several tens of heavy atoms) organic systems. In order to select the best XC functionals for our purposes, as well as to further test the performance of CC2, we first calculate the electronic transition energies and the related reorganization energies for the oligofurans containing from two to nine rings and compare them with the available experimental data [16,28]. The best performing functionals and the CC2 method are then employed in theoretical analysis of the vibronic spectral activities for the three lowest oligofurans. It is worth noting that oligofurans are planar in their ground states, in contrast to oligothiophenes, which adopt trans-gauche conformations between the adjacent rings. This is caused by the relatively low aromaticity of furan [39], owing to which the π-electrons, being less confined within the rings, are considerably delocalized over the whole oligomer chain (which increases the coupling between the rings and hinders the rotations along the inter-ring bonds) [17]. This structural regularity and considerable electron delocalization make oligofurans an ideal, even if demanding (owing to their size), playground for testing the performance of theoretical methods. Such tests are especially important for Kohn–Sham DFT, as it suffers from the arbitrariness of choosing the appropriate version of the exchange-correlation functional from the ever-growing number of approximations. It is worth noting that selecting the functionals that can correctly reproduce the reorganization energies and vibronic activities is essential from the standpoint of organic electronics, as the nature and efficiency of the energy transport in the molecular crystals (and aggregates) are governed by the reorganization energy (relative to the charge/excitation transfer integral) and modulated by the same Franck–Condon overlap integrals (in the case of the coherent transfer) that determine the relative intensities of the vibronic features observed in the optical spectra [40,41,42].

One of the major sources of errors in DFT calculations, crucial for description of the extended, unsaturated systems, is the many-electron self-interaction error (SIE) [43,44], which manifests itself in the incorrect (exponential rather than r^−1^) long-distance behavior of the DFT exchange-correlation potentials that results in seriously underestimated values of Rydberg and charge-transfer states, overshot polarizabilities, and the tendency to overestimate electron delocalization shown by pure DFT functionals [45]. Since the Hartree–Fock (HF) method overly favors the localized structures, the so-called hybrid functionals that contain some portion of the non-local (or exact) exchange (calculated using the occupied Kohn–Sham orbitals in the same way as in the HF method) were designed as a remedy to these problems. Unfortunately, replacing the DFT exchange with the non-local one leaves unbalanced the SIE of the correlation part, which in pure DFT functionals is at least partly compensated by the SIE of the DFT exchange. Interestingly, the optimum fraction of the non-local exchange in the functional seems to depend not only on the particular property that a functional is dedicated to but also on the size of the molecule, especially if it contains extended systems of conjugated double (or triple) bonds.

Such behavior can be readily observed for the linear carbon chains (polyynes), which are one of the simplest extended systems of delocalized π-electrons. In this case, the calculated differences between the adjacent CC bonds (the bond length differences, BLDs) were found to be closely related to the admixture of the non-local exchange in the DFT functional: the pure general gradient approximation (GGA) functionals yielded strongly underestimated BLDs, while the hybrid functionals performed better, with the BHLYP (50% of the exact exchange) results being the closest to the experimental values [46]. The frequency of the dominant Raman active mode (the so-called α mode, or the Я mode in the Effective Conjugated Coordinate (ECC) Theory [47]), obtained using the DFT method, were found to decrease with the growing chain length in accordance with the experimental observations [48]. The frequencies themselves, however, as well as the degree of their changes are functional-dependent, since they are functions of the BLDs [49,50]. An analogous situation was found for the HOMO–LUMO gap, which was strongly underestimated by the GGA functionals and overshot by those with large contents of the non-local exchange (e.g., BHLYP) [46]. The errors were not constant but depended on both the functional and the chain length [51]. The quality of the BLDs obtained with the ab initio methods increased predictably with improvement of the model of the N-electron wavefunction, with the coupled cluster singles and doubles with perturbative correction from the triple amplitudes (CCSD(T)) yielding nearly accurate values for several short poliynes [46,52,53,54].

Organic chromophores are another example of such challenging systems, and since their size in most cases prevents doing the correlated ab initio calculations, DFT remains the method of choice. Owing to the potential sensitivity of the DFT results to the choice of the approximate exchange-correlation functional, we initially studied the behavior of the representative XC functionals for oligofurans with increasing chain length (up to 9O, containing 36 conjugated double bonds) and subsequently select the most suitable ones that will be used to model the experimental spectra of 2O, 3O, and 4O.

## 2. Computational Details

Preparation of the theoretical spectra presented in this paper require molecular geometries to be optimized for the ground and excited electronic states, as well the vibrational analyses to be performed at both stationary points. Differences between the molecular geometries in the ground and the S_1_ electronic states projected onto the normal coordinates of either of these states yield the Franck–Condon (FC) factors that govern the vibronic activity accompanying the electronic transitions. The FC factors are calculated using the Dushin program of Reimers [55]. The theoretical spectra are simulated using the ezSpectrum program [56] that, apart from the spectra based on the FC factors only (assuming that the normal modes in both electronic states are virtually the same—which is the so-called parallel approximation), allows also for the inclusion of the normal mode mixing (Dushinsky rotations) in the spectra. Since it did not increase computational time, we have decided to take the Dushinsky effect into account even if the impact of the normal mode mixing is likely to be negligible for spectra recorded at 77 K, owing to their limited resolution.

The quantum chemistry calculations have been performed employing predominantly DFT and TDDFT methods. Owing to the sensitivity of DFT results to the composition of the exchange-correlation (XC) functional (especially strong for extended unsaturated systems), we employed several functionals with different admixtures of the non-local exchange: BP(86)—pure GGA functional (0% of the non-local exchange) [57,58], TPSSh meta—GGA functional containing 10% of the non-local exchange [59,60], B3LYP—hybrid functional with 21% of the non-local exchange [61,62,63], PBE0—a hybrid functional with 25% of the non-local exchange [64], MN15—the most recent of the Minnesota meta-GGA hybrid functionals of Thrular et al., containing 44% of the non-local exchange [65], BHLYP—a hybrid functional with half of the exchange calculated in the HF-like manner [66], and three range-corrected functionals. For the latter, the contribution of the non-local exchange varies with the distance from the reference electron: CAM-B3LYP (with 19–65% of the non-local exchange) [67], tuned-CAM-B3LYP—a version of CAM-B3LYP originally dedicated to the description of the excited states of diarylethene derivatives (8–100% of the non-local exchange) [68], and ω-B97X (16–100% of the non-local exchange) [69]. To compare DFT with the ab initio calculations, we have selected the coupled clusters singles and doubles model, in which the amplitudes for the singly excited configurations are computed rigorously, whereas the amplitudes for doubles are added perturbatively [30]. Such a simplification of the parent CCSD model reduces the computational cost by an order of magnitude (from N6 to N5), which allows for tackling systems as large as hexaphenyl [70] with a reasonable basis set. Interestingly, the CC2 calculations often yield electronic transition energies that are more accurate than with those obtained using the more complete (CCSD) model [71].

All the calculations have been performed with the def2-TZVPP basis set, which provides accuracy close to the complete basis set limit (especially for the DFT methods) at still reasonable computational cost. Preliminary calculations with the cc pVDZ basis set showed that reduction of the basis set brought minor changes to the calculated energies. It is, thus, reasonable to expect that a further increase of the basis set (e.g., to QZVPP) would not lead to significant improvement of the results.

All the quantum chemistry calculations were done using the Turbomole 7.5 package [72], apart from those that employed the meta-GGA hybrid functionals (TPSSh and MN15), for which we used Gaussian 16 [73], as Turbomole lacks the current density response for meta-GGA functionals. Without it, some dependence of the results on the orbital rotation may occur. This is not likely to be strong, but we did observe some instability of the transition energies in exploratory calculations. The errors were negligible as far as the transition energies were concerned, but could be quite significant for the energy differences, or, in the present context, for the reorganization energies.

## 3. Results

### 3.1. Transition Energies and Reorganization Energies

**Figure 1:**
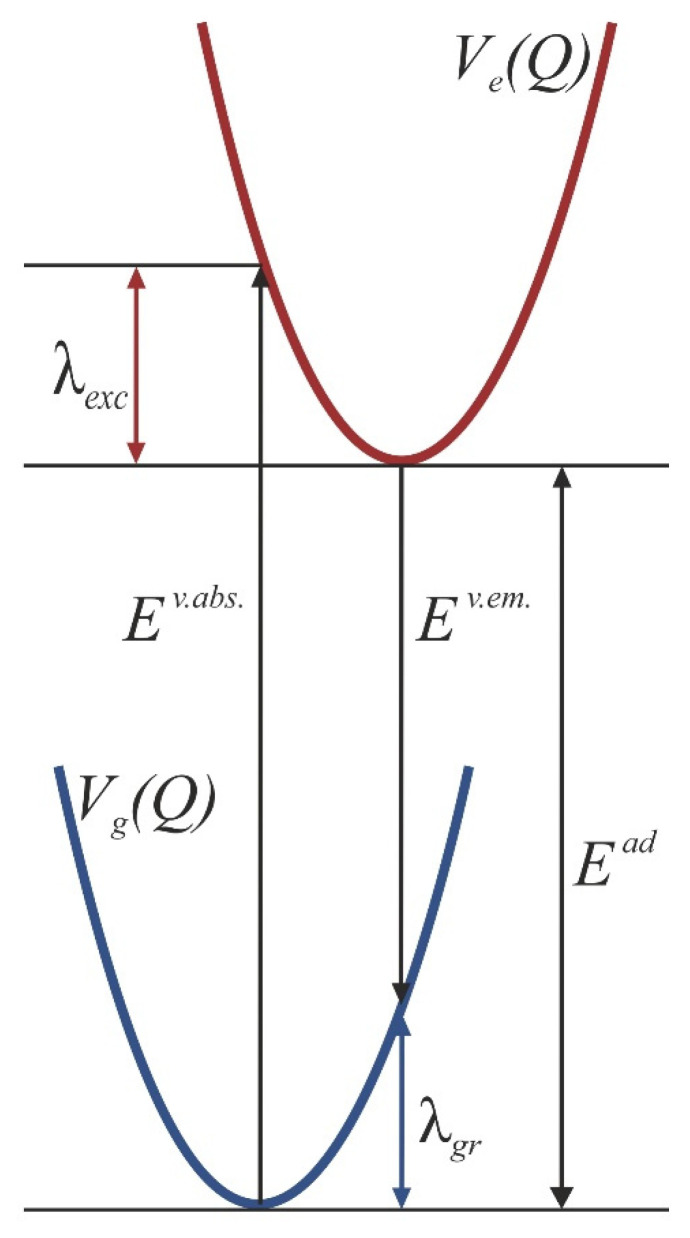
Scheme of the potential energe surfaces for the ground and for the excited state illustrating the concept of reorganization rnergies and the notation used in the text.

The adiabatic transition energies (*E^ad^*) between the ground state and the lowest excited singlet state are calculated as differences between the electronic energies corresponding to optimized geometries in both electronic states. The vertical absorption energy (*E^v.abs.^*) is the difference between the energies of the S_1_ state and the ground state when both are calculated in the optimized geometry of the ground state, while the vertical emission Energy (*E^v.em.^*) is the same difference but between the energies computed for the optimized geometry of the excited state. As follows, the excited state reorganization energy *λ^exc^* is given as *E^v.abs.^* − *E^ad.^*, whereas its ground state counterpart *λ^gr^* equals *E^ad^* − *E^v.em.^*. Thus, the defined energies are schematically depicted in Figure 1. *E^ad.^* corresponds to the experimentally discernible/observable 0-0 energy, provided that the zero-point energies in both electronic states are close to each other. *E^v.abs.^*/*E^v.em.^* is commonly attributed to the position (in the energy scale) of the maximum of the absorption/emission band. Strictly speaking, the vertical energies correspond to the centers of gravity of the respective transition bands. In the case of the room-temperature absorption spectra, the maxima are clearly visible, whereas estimation of the centers of gravity is difficult owing to the higher energy absorption bands that overlap with the signal of the S_0_–S_1_ excitation. Therefore, for our study, we chose to use directly the energies of the maxima given in the experimental papers. For the emission, the situation is quite different, as the spectral bands are not ‘contaminated’ by spurious signals, but even at room temperature, they display a distinct, vibronic structure, without a single dominating maximum. Therefore, the vertical emission energies for the studied oligofurans have been determined by calculating the centers of gravity of the respective fluorescence bands (which directly correspond to the vertical emission energies) [74]. Note that all the experimental spectra have been transferred from the wavelength to the wavenumber domain using the following conversion factors:

(1) $$\left(\lambda ,I\right)\to \left(\tilde{v}=\frac{1}{\lambda },\tilde{I}=I\cdot \lambda^{2}\right)$$

The experimental values of the vertical absorption and emission energies are given in Table 1 and Table 2, respectively, for comparison with the transition energies obtained from the quantum chemistry calculations. One should bear in mind, however, that the spectra were recorded in solutions (acetonitrile or dioxan) and are slightly red-shifted with respect to measurements in vacuum. Therefore, to serve as the reference for the quantum chemistry results, the experimental transition energies have been increased by 0.15 eV, which is a typical value of gas-to-solvent shift for organic chromophores in non-polar solvents (This value is corroborated by the DFT/TDDFT calculations performed for 2O using all of the selected XC functionals and the COSMO model of solvation for two experimentally used solvents—acetonitrile and 1,4 dioxane. The resulting shifts are rather uniform and range from 0.122 to 0.164 eV (cf. Supplementary Materials Table S4 of the electronic supplement)). The calculated values of vertical absorption and emission energies are collected in Table 1 and Table 2, respectively. The electronic transition energies monotonously decrease as the number of furan rings in the oligomer grows, or as the system of the delocalized π-electrons becomes more extended. This dependence is to a good approximation linear with respect to the reciprocal of the number of rings (1/*n*), but saturation has been observed for larger *n* (deviations from the linear trend begin at *n* > 12 [75]) for several unsaturated oligomer series studied at various levels of theory [76,77,78,79].

**Figure 2:**
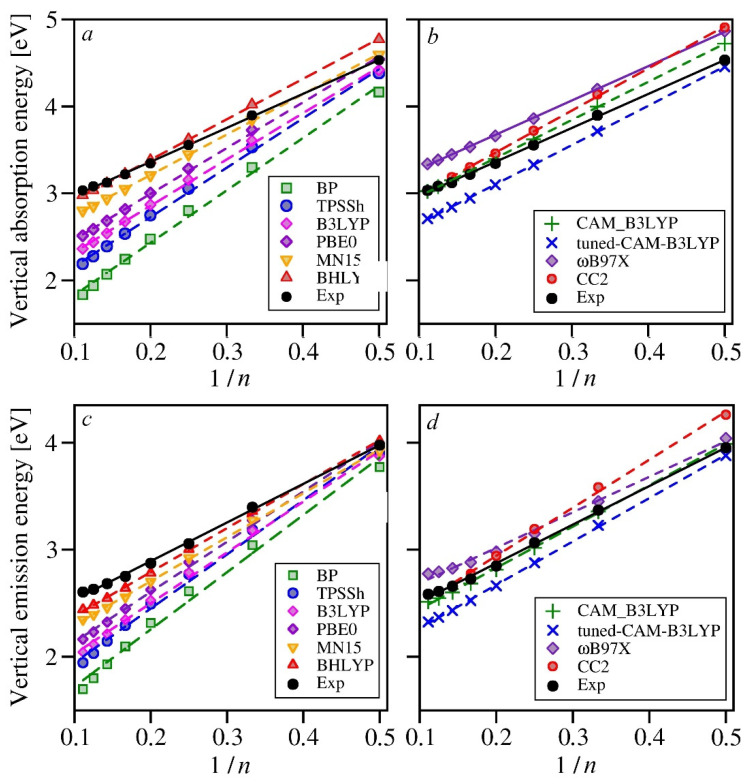
Experimental and calculated values of the electronic transition energies (absorption: panels (**a**,**b**), emission: panels (**c**,**d**) for the studied oligofurans with respect to the reciprocal number of rings (1/*n*). Dashed lines represent linear fies to the computed energies. Fitted parameters are collected in Table 4. (For the sake of clarity the results obtained with the GGA and hybrid functionals are shown in panels (**a**,**c**), while those yielded by the range corrected functionals and CC2 are shown in panels (**b**,**d**).

**Figure 3:**
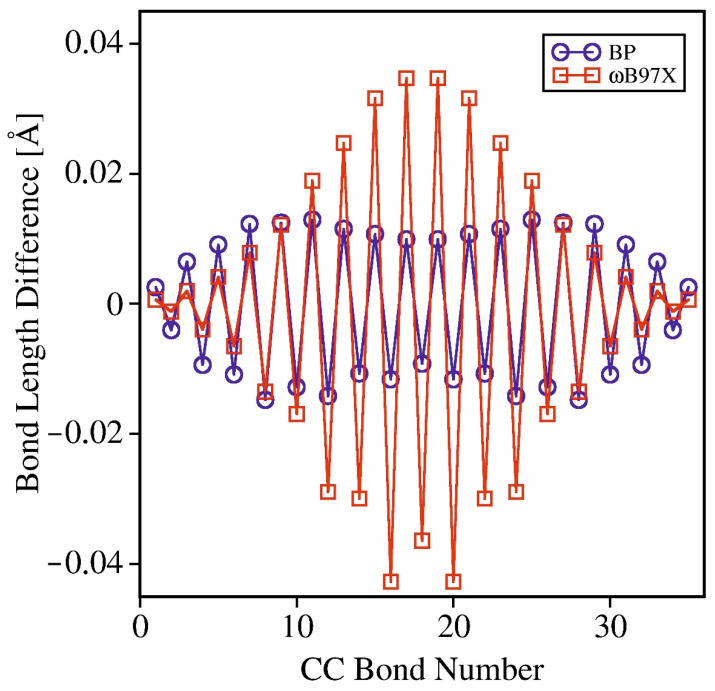
Respective CC bond length differences along the carbon skeleton of 9O, caculated for the excited and the ground electronic states using the pure DFT functional and the range-corrected functional containing up to 100% of the non-local exchange.

Our present calculations for oligomers up to nonafuran confirm the linearity of the relation *E*(1/*n*) (see Figure 2 and Figure 3), even if some deviations can be noticed for the pure DFT functional (BP), for which the dependence is slightly concave, and also for the ωB97X, for which it becomes convex for longer oligomers. Note that experimental energies also seem to closely follow the linear trends. Figure 2 clearly shows that not only the calculated energy values but also the rates of their changes with respect to 1/*n* strongly depend on the choice of the approximate exchange-correlation functionals. All of the functionals with the fixed contributions of the non-local exchange (from BP to BHLYP) generally underestimate the transition energies, and the deviations from the experimental values increase with the oligomer length (cf. Figure S2 of the electronic supplement). Only the BHLYP functional slightly overshoots the absorption energies for the three shortest oligofurans (2O–4O), yet for the longer ones, the errors become negative, even if relatively small (not exceeding 0.23 eV for 9O). This agreement, however, is more accidental than systematic, as the slope of the *E*(1/*n*) dependence for this functional is considerably steeper than for the experimentally based trend (cf. Table 3), so for longer oligomers, the errors would most certainly increase.

The functionals containing less non-local exchange than BHLYP perform even worse, yielding negative errors already for bifuran. As the slopes of the *E*(1/*n*) functions are even steeper than that for BHLYP, the errors quickly grow with the increasing number of rings in the oligomer (cf. Figure 2, as well as Figures S2 and S3 of the electronic supplement). It is evident that the less non-local exchange in the functional, the more underestimated transition energies; the longer the unsaturated oligomer chain, the larger the deviations from the experiment. For the range-corrected functionals, the results deviate less from the experimental values; not all of the errors are negative, and the slopes of the *E*(1/*n*) linear functions fitted to the calculated transition energies are smaller than for the classic hybrid functionals, thus being closer to the experimental trend. This is especially true for the ωB97X functional, for which the absorption energies are uniformly overestimated by little more than 0.3 eV for all of the studied oligomers. The error is quite substantial, but being constant, it can be simply subtracted from the absorption energies calculated with the ωB97X functional for oligofurans of any length. This is quite a desirable property, especially if it could also be confirmed for other extended unsaturated systems, line oligothiophenes, oligopyrroles, or oligophenyls. Unfortunately, for the emission energies, ωB97X does not work equally well, as the discrepancies between the computed and experimental values seem to grow with the number of rings, even if the increase is relatively slow. The smallest errors for the emission energies have been obtained with the CAM-B3LYP functional, for which also the *E*(1/*n*) function follows the experimental trend the most closely.

The reoptimized version of CAM-B3LYP (tuned-CAM-B3LYP) yields larger errors than the original version of this functional: the transition energies (both for absorption and emission) are underestimated for all oligofurans, with the errors growing for longer oligomers. It may seem surprising at first, because the long-range contribution of the non-local exchange reaches 100% in the tuned version of CAM-B3LYP, yet it behaves more like the classic hybrid functionals, such as MN15 or BHLYP, than the parent CAM-B3YP functional or ωB97X, which also has 100% contribution of the exact exchange in the long-range limit. Tuned CAM-B3LYP, however, contains considerably less non-local exchange at short distances (from the reference electron), i.e., 8%, while for ωB97X and CAM-B3LYP, this is 16% and 19%, respectively. Additionally, the long-range limit is reached at considerably larger distances (The *μ* parameter is reduced from 0.33 in the original CAM-B3LYP functional, or 0.3 in ωB97X, to 0.15, which decreases the steepness of the error function that governs the change from short-range to the long-range behavior, expanding the transition region between the two regimes). Thus, it appears that the short-range content of the non-local exchange and the size of the transition region are equally important for the correct reproduction of the transition energies as the long-range composition of the exchange part of the functional. The interplay of these three factors may offer sufficient flexibility for a successful optimization of the range-corrected functional (e.g., CAM-B3LYP) for the specific case of the extended unsaturated systems (oligomers and other organic chromophores) and their excited states.

The transition energies obtained using the CC2 method have relatively large errors for both the absorption and emission energies. The energies also decrease more steeply with 1/*n* as compared to the experiment (for both absorption and emission). Such discrepancies have not been expected from the method that was used previously in several successful attempts of theoretical analyses of the optical spectra of unsaturated organic systems [33,34,35,36,37]. A closer inspection, however, shows that the respective errors of the CC2-based absorption and emission energies are close to each other, and the behavior is mimicked only by two of the tested DFT functionals: MN15 and tuned-CAM-B3LYP (cf. Figures S1 and S2 of the electronic supplement). Moreover, the slopes of *E*(1/*n*) functions fitted to the CC2 absorption and emission energies differ by the same amount (0.3) from one another as the slopes of the functions fitted to the experimental energies. Such performance, unique among the tested methods, is essential for modeling the optical spectra, as it leads to the correct reproduction of the reorganization energies, which determine the width, shape, and—distributed among the totally symmetric normal modes—the fine structure of the spectral band.

To obtain the reorganization energies based on the experimental data, one needs to estimate the vertical absorption and emission energies, as well as locate the adiabatic energies. The former can be determined from the first moment of the spectral bands (or their “centers of gravity”) [74], or even approximated as the position of their maxima if the bands lack the fine structure and are not overly asymmetric. Finding the adiabatic energies is more difficult, especially for the spectra recorded at room temperature, because the onset of the band does not necessarily correspond to the 0-0 line, which is often poorly resolved or overlapped by the hot bands. Bearing in mind that even minute errors of the adiabatic energies may lead to quite large errors in reorganization energies (as they are given as differences between the two similar and relatively large values), we have decided to eliminate the adiabatic energies from the picture by taking as the experimental reference the halved differences between the vertical absorption and emission energies. Thus, defined values correspond to the average of the excited state and the ground state reorganization energies. For the planar, organic molecules, such as oligofurans, for which the S_0_–S_1_ transitions are of ππ* origin (dominated by the HOMO–LUMO electronic excitations), these two reorganization energies can be expected not to differ much from one another. Browsing through the calculated values of the reorganization energies for all of the studied oligofurans (cf. Table S2 of the electronic supplement) shows that this is, indeed, true.

The reorganization energies do not follow the linear trend with respect to the reciprocal number of rings so closely as the transition energies do. This, however, is quite understandable as the reorganization energies are strongly sensitive to even small inaccuracies of the transition energies (either calculated or estimated from experiment). Additionally, for long oligomer chains, the calculated reorganization energies can be expected to saturate with increasing chain length, because the electronic transition-induced geometry changes tend to localize in the central region of the oligomer chain, leaving the terminal rings virtually intact. The degree of this localization, however, depends strongly on the choice of XC functional. It is the most pronounced for the functionals with large contributions of the exact exchange, while it does not seem to occur whatsoever for pure DFT functionals.

Such behavior can be rationalized in terms of the inherent preference of the HF method for localized electronic structures. Very similar observations were reported for oligothiophene cations containing up to 50 thiophene rings for the functionals with high large content of the non-local exchange [80]. For the oligofurans analyzed here, the chains are too short to appreciate the full extent of this effect, but the tendency can already be observed, as demonstrated in Figure 3. It shows the striking difference between the pictures obtained using BP (pure GGA functional) and the ωB97X functional (in which the contribution of the exact exchange reaches 100% for long distances from the reference electron). The differences between the respective CC bonds in the excited and the ground electronic states are concentrated in the center of the chain for the ωB97X-based geometries, while for BP, the differences are delocalized over the whole molecule (the central part is even somewhat less affected). There is no direct experimental evidence that might confirm the picture obtained with either of the two functionals, yet the overall improvement of the transition energies and the reorganization energies observed for the hybrid functionals suggests that it is these functionals that can grasp (at least qualitatively) the genuine physical effect. This conjecture is indirectly corroborated by an analogous mechanism operational, e.g., in molecular crystals, in which the geometry distortions localized within the range of several nearest neighbors in the crystal lattice accompany the electronic excitation, leading to self-trapping of the excitons and to the formation of polarons.

**Figure 4:**
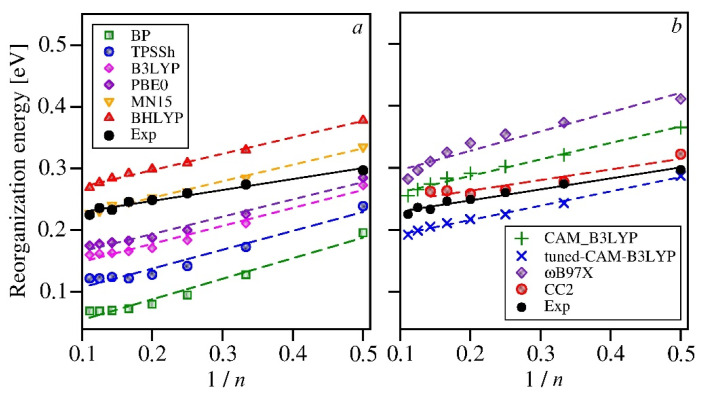
Experimental and caculated values of the averaged reorganization energies for the studied oligofurans with respect to the reciprocal number of rings (1/*n*). Dashed lines represent linear fits to the computed energies. Fitted parameters are collected in Table 4. (For the sake of clarity the results obtained with the GGA and hybrid functionals are shown in panel (**a**), while those yielded by the range corrected functionals and CC2 are shown in panel (**b**)).

Given the above rationale, however, one might expect that the calculated reorganization energies would start to saturate with the number of rings first for the functionals that contain high contributions of the non-local exchange (BHLYP, ωB97X). Figure 4 shows, however, that the most pronounced saturation occurs for the pure DFT functional (BP) and those with small admixtures of the exact exchange (TPSSh, B3LYP). The nearly linear dependence (no visible saturation) has been obtained for PBE0, MN15, and BHLYP. The reorganization energies calculated with the range corrected functionals behave in a somewhat peculiar way, as they seem to decrease faster (instead of showing saturation) for long oligofurans. A deeper investigation into this issue falls beyond the scope of this study. One may speculate, however, that the varying contribution of the non-local exchange in the range-corrected functionals is at least partly responsible—it may have a beneficial influence on the transition energies, but it may also introduce subtle errors, which are revealed only when the difference values (such as the reorganization energies) are calculated.

The reorganization energies calculated in our study using the DFT functionals with the fixed contribution of the non-local exchange are strongly underestimated for all the studied oligofurans by the functionals, for which the contribution of the non-local exchange does not exceed 25% (Figure 4). The BP functional is completely inadequate for computing the reorganization energies (and for further modeling of the spectra), as the errors reach nearly 70% for longer oligofurans. The TPSSh does perform better, but the 10% admixture of the exact exchange is insufficient to reach even semi-quantitative agreement with the experiment. B3LYP and PBE0 can be used 2O, or 3O, but even for the shortest oligofurans the errors are quite substantial. Some increase of the content of the non-local exchange in the functional leads to larger values of the reorganization energies, thus reducing the deviations from the experimental estimates. The reorganization energies obtained with MN15 (44% of the exact exchange) are surprisingly accurate, with the errors less than 4% for all the studied oligofurans except for 2O (for which the calculated value is overestimated by 14%). BHLYP-based reorganization energies, however, being considerably higher than those obtained with MN15, exceed the experimentally based estimates by about 20%. Apparently, the fixed 50% contribution of the non-local exchange is already too large for modeling of the optical spectra of oligofurans. From among the range-corrected functionals, the tuned-CAM-B3LYP yielded the smallest errors, underestimating the reorganization energies by less than 15%. Using its general-purpose counterpart (CAM-B3LYP) has led to reorganization energies that are by 13–23% larger (the errors decrease for longer oligomers) than the experimental estimates. The reorganization energies obtained with the ωB97X functional bear relatively high, positive errors for all the oligofurans under study.

**Figure 5:**
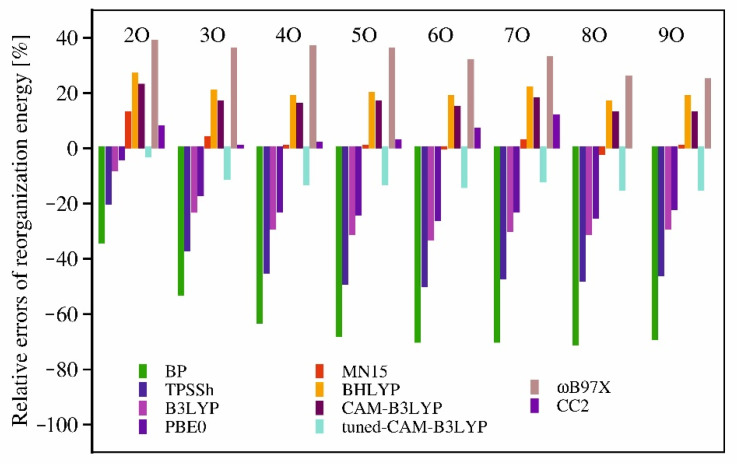
Relative errors of the calculated reorganization energies for the studied oligofurans.

The values of reorganization energies obtained with all DFT functionals considered here decrease too rapidly, with the slope that depends on the content of the non-local exchange. This is clearly visible in Figure 5 (and also in Figure S4, which shows the actual error values), as well as in Table 4, which contains the slope parameters of the linear functions *λ*(1/*n*) fitted to the calculated and experimental values of the reorganization energies. The reorganization energies computed using CC2, on the other hand, seem to follow the trend that is nearly parallel to the experimental one. Hence, the deviations of the CC2 reorganization energies from experiment are not only small (1–12%), but also similar for all of the oligomers (up to 7O; unfortunately for 8O and 9O the calculations could not be converged for technical reasons). This feature of the CC2 method is likely to be the foundation of its good performance in modeling the optical spectra of organic chromophores. This method will, therefore, be the basis of our theoretical analysis of the vibronic structures in the optical spectra of 2O, 3O, and 4O. For comparison, we will also demonstrate the theoretical spectra obtained using the results of the MN15 and the tuned CAM-B3LYP calculations, as those two functionals yielded the smallest errors of the reorganization energies.

### 3.2. Theoretical Modeling of the Optical Spectra

Vibronic activity observed in the optical spectra of organic chromophores is often characterized in terms of Huang-Rhys (HR) factors S_i_ = *b_i_*^2^/2. The Franck–Condon (FC) parameters, *b_i_*, can be evaluated from the formula:

(2) $$b_{i}=\left(\frac{\omega_{i}}{\hbar }\right)\cdot \left[x_{A}-x_{B}\right]M^{1/2}L_{i}^{A},$$

where ***x****_A_* and ***x****_B_* are the 3N-dimensional vectors of the equilibrium Cartesian coordinates in the *A*-th and *B*-th states, respectively, ***M*** is the 3N × 3N diagonal matrix of the atomic masses, and $L_{i}^{A}$ is the 3N vector of the mass-weighted Cartesian displacements constituting the i-th normal coordinate calculated for the *A*-th (final) electronic state. For symmetry reasons, the HR factors are non-zero only for the totally symmetric normal modes. The sum of the HR factors multiplied by the respective oscillation quanta is equal (within the accuracy of the calculated harmonic frequencies) to the reorganization energy for the final electronic state in the studied transition. The HR factors for 2O, 3O, and 4O are collected in Table 5. For the sake of clarity, the normal modes with contribution to the total reorganization energy below 1% are omitted (the complete sets of the FC parameters, HR factors, and the fraction of the reorganization energies for the three smallest oligofurans, calculated using all of the methods studied in this work, can be found in Table S3 of the electronic supplement). We have chosen the HR factors instead of the FC parameters since the former better discriminate the progression-forming modes. The HR factors are also direct measures of the relative intensity the fundamental replicas in the FC progressions with respect to the 0-0 line.

The largest values of the HR factors for all three oligofurans are associated with the normal mode around 1700 cm^−1^, in which the adjacent single and double CC bonds of the carbon skeleton of the molecule vibrate in antiphase. This is the same mode that is termed the Я mode in the framework of the Effective Conjugation Coordinate (ECC) Theory [47], according to which for the π-conjugated systems with strong electron-phonon coupling such a normal mode must manifest itself in the Raman spectrum as a single, very strong scattering at about 1500–1700 cm^−1^. The scattering frequency decreases as the extent of the electron delocalization gets larger. The ECC theory was used to estimate the effective conjugation length in oligofurans, yielding the estimate of 14.5 repeat units [81]. It turns out that the Я mode dominates the vibronic activity for the analyzed oligofurans, being responsible for 55% of the total reorganization energy, and together with the satellite vibrations from the spectral region above 1425 cm^−1^, they carry from 59% (2O, emission) to 72% (4O, absorption) of the total reorganization energy. The other type of vibrations that have a noticeable impact on the vibronic activity are the ring breathing modes, located between 815 and 1035 cm^−1^, contributing from 20% (4O, absorption) to 33% (2O, emission) to the total reorganization energy. The remaining normal modes cover for less than 10% of the reorganization energy and cannot be resolved in the spectra recorded at liquid nitrogen temperature. Nonetheless, those vibrations cannot be neglected, as their contribution is important for the overall width of the model spectra.

The theoretical spectra are formed by the overlapping vibronic bands, the energy of which (with respect to the 0-0 line) is given as:

(3) $$E_{v_{1},\ldots ,v_{k}}=\sum_{i=1}^{k}v_{i}\hbar \omega_{i},$$

where $v_{i}$ denotes the numbers of vibrational quanta of the totally symmetric normal modes that are excited during the electronic transition. The vibronic bands are represented as a Lorenz-type shape function with the width of 250 cm^−1^ for the absorption spectra, and half of that for the fluorescence. The relative intensities of the individual vibronic bands are determined by the FC overlap integrals, available from the well-known recursion formulas [82] using the computed HR parameters.

By virtue of the ezSpectrum software of Krylov et al., we have included the Dushinsky mixing of the normal modes induced by the electronic transition, even though this effect has turned out to be quite small for the spectra of medium resolution that we model in this work (Figure S5 of the electronic supplement shows a comparison of the selected spectra obtained with and without the Dushinsky mixing). The intensity throughout the calculated emission bands is multiplied by the factor ${\tilde{\nu }}^{3}$ to account for the fact that the intensity distribution in the emission spectrum is proportional to the third power of the energy of the emitted light, according to the relation [83]:

(4) $$A=\frac{8\pi^{2}}{3\hbar \varepsilon_{0}}{\tilde{\nu }}^{3}{\left|\mu \right|}^{2},$$

in which *A* is the Einstein coefficient for the spontaneous emission, $\varepsilon_{0}$ is the permittivity of the vacuum, and *μ* represents the electric transition dipole moment. Analogous correction for the absorption spectra requires multiplication by $\tilde{\nu }$, as the oscillator strength is proportional to the first power of the energy of the absorbed radiation:

(5) $$f=\frac{4\pi m_{e}}{3\hbar e^{2}c}\tilde{\nu }{\left|\mu \right|}^{2},$$

where $m_{e}$ is the mass of an electron, *e* is its charge, and *c* is the speed of light. The transition dipole moments were computed together with the transition energies, yet we have not used them, as the absolute intensities of the experimental spectra were not reported in the study of Seixas de Melo et al. [28]. Therefore, we chose instead to normalize the theoretical spectra so that they match the total (integral) intensity of the experimental ones. Note that the vibrational frequencies have been multiplied by 0.95, which is an appropriate scaling factor for all of the three methods selected for this analysis [84,85].

**Figure 6:**
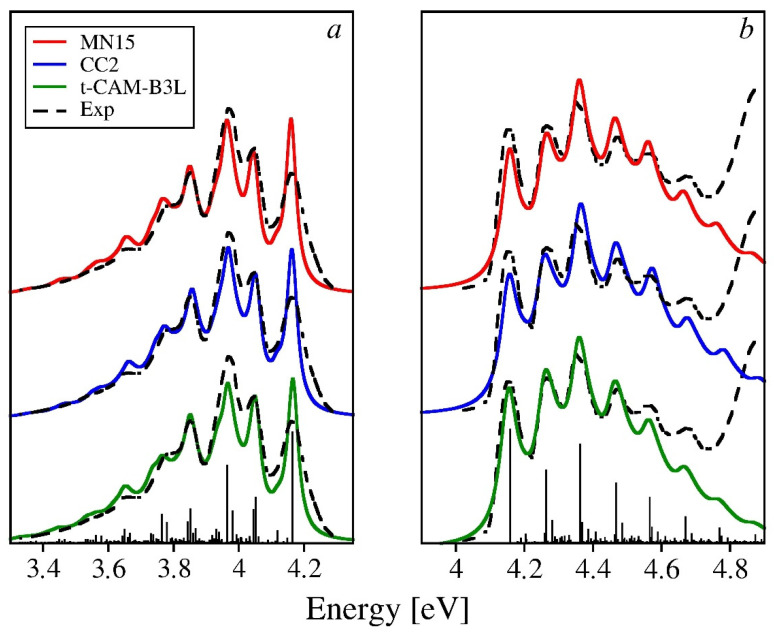
Emission (**a**) and absorption (**b**) spectra of 2O calculated using three theoretical methods selected in the previous section vs. the experimental spectrum by Seixas de Melo et al. [28]. The integral intensities of the theoretical spectra have been normalized to match the experiment. To ease the comparisons, the onsets of the computed spectra have been aligned to the experimental one. The stick spectrum has been obtained using the MN15 functional.

The experimental absorption and emission spectra of 2O and their theoretical counterparts based on the vibronic parameters calculated with the MN15, tuned-CAM-B3LYP exchange-correlation functionals, as well as with the CC2 method, are presented in Figure 6. The reorganization energies obtained with either of the selected theoretical methods are close to the experimental estimates: MN15 has yielded a value that is overshot by 13%, the error of the CC2 result is 8%, while the value of tuned-CAM-B3LYP is −3%. A good match between the theoretical and experimental spectra can, therefore, be expected. The actual picture shows that while the frequencies of the vibronic features are reproduced almost quantitatively (after the appropriate scaling), some minor discrepancies between theory and experiment can be observed in the intensity domain. For the theoretical absorption spectra, the first two maxima are too low with respect to the third one, which might suggest that the HR parameters for the dominant normal mode (1776 cm^−1^) are somewhat overestimated. This deviation is more pronounced in the spectra based on the MN15 and CC2 results, while the best agreement with experimental absorption spectrum has been reached indeed for the tuned-CAM-B3LYP functional (which is consistent with the smallest error for the reorganization energy). The relative intensities of the first four vibronic features are here reproduced nearly perfectly (the third maximum is very slightly overshot). Some intensity deficiency visible at higher energies (4.5–4.7 eV) is probably an experimental artifact, owing to the considerable background intensity in that spectral region brought about by the long-wavelength tail of some higher energy absorption band, clearly visible as the steeply rising intensity above 4.8 eV.

Theoretical fluorescence spectra follow closely the experimental ones in the low energy (tail) region, which indicates that the overall reorganization energy for the ground state (*λ_gr_*) governing the width of the spectral band has been indeed correctly reproduced. Deviations from the experiment are mainly observed in the higher energy part of the band (close to the onset): the intensity of the first maximum (corresponding to the 0-0 transition) is noticeably overshot, while the next two vibronic features have slightly insufficient intensities (especially for the spectrum based on the tuned-CAM-B3LYP results). It might seem that the HR parameters for the dominant vibrations (1687 cm^−1^ and 930 cm^−1^) are underestimated, thus excessively accentuating the 0-0 line and underestimating the fundamental vibronic replicas. A closer look at the values of the calculated reorganization energies (Table S2 of the electronic supplement) shows that *λ_exc_* values are larger than *λ_gr_* by 7–9% for all three theoretical methods selected in the previous section, which may affect the HR parameters for the progression forming modes and account at least for part of the observed discrepancies. The situation, however, is probably more complex, as the spectral region of the second and third maxima is shaped not only by the FC replicas of two dominant vibrations, but also by numerous combination bands involving other normal modes, contributing to the background intensity (cf. the stick spectrum in Figure 6). Moreover, larger values of the HR factors for the two abovementioned normal modes would have to result in the elevated intensity of the low-energy tail of the calculated spectra and, as a consequence, in deteriorated agreement with the experiment. It appears that some non-Condon mechanism influences the intensity pattern in the experimental emission spectrum. One of the possibilities is the frequently occurring self-absorption that consumes some of the intensity of the first maximum, modifying the intensity ratio of the first and two following peaks. Whether this conjecture is true, however, would have to be verified in an independent experiment.

**Figure 7:**
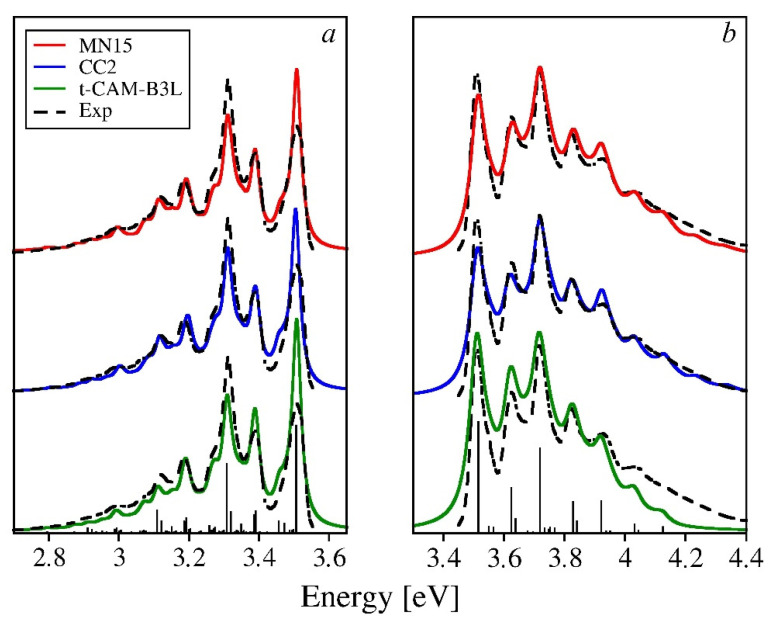
Emission (**a**) and absorption (**b**) spectra of 3O calculated using three theoretical methods selected in the previous section vs. the experimental spectrum by Seixas de Melo et al. [28]. The integral intensities of the theoretical spectra have been normalized to match the experiment. To ease the comparisons, the onsets of the computed spectra have been aligned to the experimental one. The stick spectrum has been obtained using the MN15 functional.

The calculated spectra of 3O are displayed in Figure 7. The agreement with the experiment is nearly perfect for the frequencies, while the intensities show discrepancies that are of the same character as those for 2O. Again, the 0-0 line in the absorption spectrum is slightly underestimated, mostly for the MN15 and CC2 based spectra, while the high-energy tails of the theoretical spectra follow closely the experimental one. The tuned-CAM-B3LYP-based spectrum best reproduces the experimental intensity ratio of the third maximum and the 0-0 line. The second maximum, however, is slightly overshot, while the high-energy tail carries insufficient intensity. The latter makes the band appear too narrow, which is in accordance with the reorganization energy underestimated by this functional by 11%. With the respective errors of 4% (MN15) and 1% (CC2), the intensity of the theoretical spectra based on those two methods are in nearly perfect agreement with the experimental one at high energies (perfectly reproduced overall width of the spectral band). The intensity of the 0-0 line, however, is again slightly too low. On the other hand, it is overestimated in the theoretical fluorescence spectra. Since the calculated values of *λ_exc_* are larger than *λ_gr_* by 7% for tuned-CAM-B3LYP, 8% for MN15, and 12% for CC2 (cf. Table S2 of the electronic supplement), it means that *λ_exc_* values are actually overestimated and *λ_gr_* values are underestimated, by a few percent (especially when the average reorganization energy is practically exact). This might then explain the slightly underestimated intensity of the 0-0 line with respect to the second or the third line in the absorption spectra, as if the related HR factors were a little too large. This conjecture is corroborated by the second vibronic replica in the FC progression of the mode 1759 cm^−1^ (the fifth maximum in the spectrum), also being a little overshot with respect to the experiment. The overestimated intensity of the 0-0 line in the fluorescence spectra and too low intensity of the third maximum seem to be consistent with the negative errors of the *λ_gr_*. Whether these errors account for all of the discrepancies, or whether self-absorption was also operational as postulated for the case of 2O, cannot be resolved at the moment without additional experiments. Apart from the minor deviations from the experiment discussed above, the overall quality of the theoretical spectra can be regarded as quite satisfactory.

**Figure 8:**
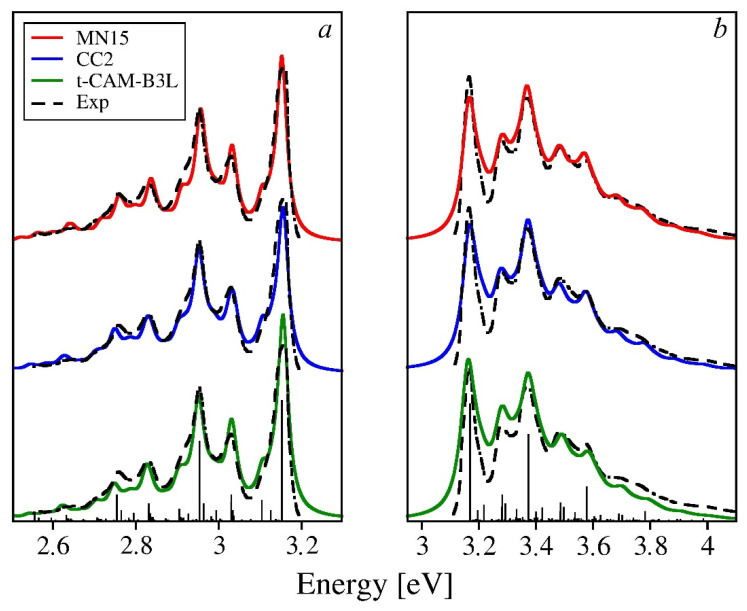
Emission (**a**) and absorption (**b**) spectra of 4O calculated using three theoretical methods selected in the previous section vs. the experimental spectrum by Seixas de Melo et al. [28]. The integral intensities of the theoretical spectra have been normalized to match the experiment. To ease the comparisons, the onsets of the computed spectra have been aligned to the experimental one. The stick spectrum has been obtained using the MN15 functional.

The theoretical spectra calculated for 4O (Figure 8) show the best agreement with the experiment for both absorption and fluorescence. Both frequencies and intensities of all the vibronic features in the spectra are nearly quantitatively reproduced. The discrepancies observed for 2O and 3O in the intensity domain are strongly reduced or inexistent. For the tuned-CAM-B3LYP-based spectra, slight intensity deficiencies can be observed far from the onset, which reflects the fact that the reorganization energies are underestimated by this functional by 13%. It is worth mentioning that the MN15 and CC2 reproduced the reorganization energy for 4O with quantitative accuracy (with errors of 2% and 1%, respectively), which shows in the excellent quality of the relevant theoretical spectra. It would require the experimental spectra to be recorded at liquid helium temperature to further verify the performance of these methods, concerning the fine details of reorganization energy distribution among the normal modes, which are not resolved in the available spectra recorded at 77 K. Preparations for such measurements are underway in our laboratory.

At the moment, however, one must conclude that increasing the chain length 2O to 4O brings about little changes to the overall pattern of vibronic activity, formed by the two dominant types of molecular motions—the ring breathing modes and the CC stretches along the carbon skeleton of the molecules. The maxima in the absorption spectra are more regularly spaced than in fluorescence, owing to the frequency shifts of the progression forming modes due to the electronic excitation (the averaged frequencies of the ring breathing modes is down-shifted by 30–50 cm^−1^, while its counterpart for the CC stretches increases by about 50 cm^−1^). As far as the evolution of vibronic activity is concerned, the two dominating types of normal modes behave differently. The HR factor for the CC stretching mode of about 1750 cm^−1^ remains virtually unchanged, while its counterparts for the ring breathing modes (800–1000 cm^−1^) undergo a significant reduction: the total HR factor for these modes drops from 0.9 for 2O to 0.5 for 4O. The impact of this decrease on the actual spectra can be observed as a gradual reduction of the intensity of the second maximum with respect to both the third maximum and the 0-0 line.

## 4. Conclusions

We have tested a representative set of exchange-correlation functionals with different contributions of the non-local exchange (from 0 to 100%) for the reproduction of the vibronic activities that accompany electronic excitations of organic chromophores. Specifically, we focused on the vertical electronic transition energies that led in consequence to the reorganization energies, the key parameters that govern the degree of vibronic coupling, and determine the shape and width of the spectral bands.

We have observed that for oligofurans containing from two to nine rings, the vertical transition energies depend linearly on the reciprocal number of rings in the molecule. The slopes of the functions *E*(1/*n*) fitted to the calculated values appear to decrease with growing contribution of the exact exchange in the XC functional. The functional that yielded the smallest errors in transition energies both for absorption and emission was CAM-B3LYP. Nonetheless, the errors were size-dependent, growing with increasing chain length, so for even longer oligofurans, they could become substantial. Only the ωB97X functional yielded absorption energies that were uniformly overestimated with the constant error of approximately 0.3 eV. The emission energies reproduced by this functional were also overestimated (by 0.14 eV on average), yet the errors here were not constant, but slowly increased with the growing chain length. Similar behavior was not observed for the tuned-CAM-B3LYP functional, which similar to ωB97X has the long-range contribution of the exact exchange reaching 100%. Its performance, however, was more similar to those of MN15 and BHLYP—the classic hybrid-functionals containing fixed, even if considerable, admixtures of the non-local exchange. Apparently, the exact-exchange dominated long-range limit of the range-corrected functional is not the only factor that affects the excitation energies of highly delocalized systems. Others (such as the short-range content of the exact-exchange or the transition rate between the limiting regimes) appear to be equally important. There seems to be room for improvement—simultaneous optimization of these factors may lead to functionals that are better suited for reproducing accurately both the values of the electronic transition energies of the unsaturated oligomers (and more generally, of the organic chromophores) and of the trends of their changes with the size of the system.

The hybrid functionals with fixed amounts of the exact exchange did not perform as well as the range-corrected ones, as they managed to yield small errors for only one or two oligomers at best, while for others, the deviations from experiment were quite large both for absorption and emission. The CC2 method seems to work in a similar fashion as the hybrid functionals, with moderate admixtures of the non-local exchange. It is, however, the only method which has yielded reorganization energies with relatively small (<12%) and approximately uniform deviations from the experimental estimates. For all of the DFT methods used in our study, the errors in reorganization energies are distinctly size-dependent. They are also larger than for CC2, with the exception of the MN15 functional, which has performed remarkably well, yielding errors below 4% for all of the oligomers but for the smallest one (for 2O, the calculated reorganization energy has been overestimated by 14%). MN15 is closely followed by tuned-CAM-B3LYP, for which the errors are slightly larger (3–15%) and negative. Nevertheless, for the reorganization energies, which as difference values are very sensitive to inaccuracies of the vertical transition energies (both theoretical and experimental), the performance of the CC2 method and the two abovementioned DFT functionals has to be regarded as very good.

This assessment is confirmed by the high quality of the theoretical spectra obtained using the HR factors based on the quantum chemistry results. Besides nearly quantitative agreement with the experiment of the frequencies of the vibronic features observed in the absorption and emission bands, the overall bandwidths are also reproduced with excellent accuracy, which is consistent with the small errors of the calculated reorganization energies. Some discrepancies of the intensities, observed for the 0-0 line and the vibronic fundamental replicas of the progression forming modes are tentatively put down to a slight imbalance of the calculated reorganization energies for the excited states, which are larger than their counterparts for the ground state by at most 12% (depending on the oligomer and theoretical method involved). This conjecture, however, requires further investigation that would employ an alternative theoretical approach, in which the excited state is not accessed by the response theory, as was done for CC2 and TDDFT. CASPT2 might be a good choice, as it treats the ground state and the excited states on equal terms. At the same time, an alternative experiment would be beneficial, especially concerning the fluorescence of oligofurans, in order to assess (or exclude) the impact of self-absorption on the intensity of the 0-0 line. New spectra, if measured at the liquid helium temperature, would shed more light on details of the vibronic activity of the remaining totally symmetric normal modes (apart from the CC stretching and the ring breathing ones), providing new reference data for further testing of the theoretical methods on such excellent model systems as the oligofurans.

## Figures and Tables

**Table 1:** Vertical absorption energies calculated for the studied oligofurans using the selected variants of DFT/TDDFT and the CC2 method with the def2-TZVPP basis set.

	2O	3O	4O	5O	6O	7O	8O	9O
**BP**	4.165	3.298	2.802	2.475	2.242	2.070	1.938	1.835
**TPSSh**	4.380	3.531	3.055	2.748	2.536	2.391	2.276	2.188
**B3LYP**	4.418	3.604	3.154	2.870	2.677	2.539	2.439	2.363
**PBE0**	4.541	3.727	3.282	3.003	2.815	2.683	2.586	2.513
**MN15**	4.593	3.847	3.449	3.208	3.050	2.941	2.855	2.803
**BHLYP**	4.771	4.020	3.621	3.380	3.223	3.114	3.036	2.978
**CAM-B3LYP**	4.720	3.998	3.621	3.396	3.250	3.149	3.078	3.023
**tuned** **CAM-B3LYP**	4.456	3.715	3.329	3.097	2.946	2.842	2.767	2.709
**ωB97X**	4.864	4.198	3.859	3.660	3.533	3.446	3.385	3.338
**CC2**	4.906	4.137	3.720	3.457	3.300	3.186	3.105	3.045
**Experiment ^1^**	**4.531**	**3.896**	**3.556**	**3.345**	**3.219**	**3.123**	**3.081**	**3.033**

^1^ Data from refs [28] (2O–4O) and [16] (for 5O–9O) have been corrected for the gas-to-solvent shift (for details, see text). The value appears in bold: It is desirable as it highlights the experimental data that serve as reference for all the calculated values.

**Table 2:** Vertical emission energies calculated for the studied oligofurans using the selected variants of DFT/TDDFT and the CC2 method with the def2-TZVPP basis set.

	2O	3O	4O	5O	6O	7O	8O	9O
**BP**	3.773	3.043	2.612	2.315	2.096	1.929	1.799	1.697
**TPSSh**	3.902	3.185	2.770	2.492	2.292	2.144	2.031	1.944
**B3LYP**	3.873	3.181	2.787	2.528	2.346	2.213	2.115	2.042
**PBE0**	3.971	3.274	2.882	2.626	2.449	2.323	2.231	2.163
**MN15**	3.923	3.277	2.924	2.703	2.557	2.460	2.393	2.347
**BHLYP**	4.015	3.360	3.003	2.782	2.640	2.545	2.482	2.440
**CAM-B3LYP**	3.990	3.357	3.017	2.813	2.684	2.601	2.547	2.513
**tuned** **CAM-B3LYP**	3.881	3.228	2.878	2.663	2.524	2.431	2.368	2.325
**ωB97X**	4.041	3.452	3.150	2.981	2.882	2.826	2.793	2.775
**CC2 ^1^**	4.262	3.584	3.193	2.942	2.775	2.663		
**Experiment ^2^**	**3.953**	**3.370**	**3.065**	**2.847**	**2.727**	**2.658**	**2.610**	**2.583**

^1^ The results for 8T and 9T are not available, owing to prohibitively large computational cost. ^2^ Data from refs [28] (2O–4O) and [16] (for 5O–9O) have been corrected for the gas-to-solvent shift (for details, see text). The value appears in bold: It highlights the experimental values that serve as reference to all the calculated ones.

**Table 3:** Reorganization energies calculated for the studied oligofurans using the selected variants of DFT/TDDFT and the CC2 method with the def2-TZVPP basis set.

	2O	3O	4O	5O	6O	7O	8O	9O
**BP**	0.196	0.128	0.095	0.080	0.073	0.070	0.069	0.069
**TPSSh**	0.239	0.173	0.142	0.128	0.122	0.124	0.122	0.122
**B3LYP**	0.273	0.211	0.184	0.171	0.166	0.163	0.162	0.160
**PBE0**	0.285	0.226	0.200	0.188	0.183	0.180	0.178	0.175
**MN15**	0.335	0.285	0.263	0.253	0.246	0.241	0.231	0.228
**BHLYP**	0.378	0.330	0.309	0.299	0.292	0.284	0.277	0.269
**CAM-B3LYP**	0.365	0.321	0.302	0.291	0.283	0.274	0.265	0.255
**tuned** **CAM-B3LYP**	0.287	0.243	0.225	0.217	0.211	0.205	0.199	0.192
**ωB97X**	0.411	0.373	0.354	0.340	0.325	0.310	0.296	0.282
**CC2 ^1^**	0.322	0.277	0.264	0.258	0.263	0.262		
**Experiment ^2^**	0.297	0.274	0.260	0.249	0.246	0.233	0.236	0.225

^1^ The results for 8T and 9T are not available, owing to prohibitively large computational cost. ^2^ Estimated as (*E^v.abs.^* − *E^v.em.^*)/2 (for details, see text). The value appears in bold: It is desirable as it highlights the experimental data that serve as reference for all the calculated values.

**Table 4:** Parameters of the linear functions of 1/*n* fitted to the calculated and experimental values of the electronic transition energies, and the reorganization energies for the studied oligofurans.

	Absorption		Emission		Reorganization	
	Slope	Intercept	Slope	Intercept	Slope	Intercept
**BP**	6.019	1.227	5.351	1.185	0.334	0.021
**TPSSh**	5.67	1.592	5.06	1.438	0.305	0.078
**B3LYP**	5.325	1.791	4.745	1.551	0.290	0.12
**PBE0**	5.255	1.942	4.692	1.667	0.281	0.138
**MN15**	4.646	2.456	4.113	1.969	0.267	0.199
**BHLYP**	4.644	2.282	4.11	1.883	0.267	0.244
**CAM-B3LYP**	4.392	2.525	3.862	2.057	0.265	0.234
**tuned** **CAM-B3LYP**	4.517	2.2	4.055	1.86	0.231	0.17
**ωB97X**	3.945	2.883	3.32	2.353	0.313	0.265
**CC2**	4.832	2.502	4.526	2.032	0.171	0.229
**Experiment ^1^**	**3.904**	**2.581**	**3.610**	**2.171**	**0.176**	**0.202**

^1^ Based on data from refs [28] (for 2O–4O) and [16] (for 5O–9O). The value appears in bold: It highlights the experimental values that serve as reference to all the calculated ones.

**Table 5:** The frequencies [in cm^−1^] and HR factors (italicized) calculated at the CC2/def2-TZVPP level of theory for three lowest members of the oligofuran series. The normal modes that contribute less than 1% to the total reorganization energy are omitted for clarity.

2O				3O				4O			
Absorption		Emission		Absorption		Emission		Absorption		Emission	
383	*0.099*	388	*0.104*	421	*0.114*	418	*0.136*	408	*0.169*	405	*0.189*
816	*0.236*	876	*0.012*	895	*0.430*	948	*0.386*	888	*0.057*	943	*0.040*
853	*0.463*	930	*0.712*	1030	*0.125*	1021	*0.211*	915	*0.280*	959	*0.258*
1020	*0.193*	1021	*0.187*					1033	*0.128*	1021	*0.202*
1193	*0.037*	1174	*0.099*	1314	*0.017*	1184	*0.023*	1347	*0.036*	1187	*0.008*
1308	*0.023*	1333	*0.025*	1363	*0.026*	1335	*0.064*	1392	*0.017*	1337	*0.089*
1447	*0.018*	1431	*0.062*	1435	*0.020*	1425	*0.017*	1488	*0.038*	1487	*0.010*
1492	*0.092*	1537	*0.183*	1516	*0.057*	1545	*0.092*	1519	*0.037*	1547	*0.057*
1776	*0.873*	1687	*0.688*	1759	*0.867*	1672	*0.700*	1660	*0.063*	1660	*0.712*
								1751	*0.780*		

The value appears in italic: The italicized numbers refer to the HR factors, while the other ones are wavenumbers.

## Data Availability

Not applicable.

## Supplementary Materials

The following are available online, Table S1: Optimized Cartesian coordinates for the studied oligofurans calculated using the selected theoretical methods (exchange-correlation DFT functionals) and CC2 with the def2-TZVPP basis set; Table S2: The excited state and the ground state reorganization energies calculated using the studied exchange correlation functionals with the def2-TZVPP basis set; Table S3: Vibrational frequencies, FC parameters, HR factors, and the related reorganization energies for the totally symmetric normal modes of 2O, 3O, and 4O, calculated using the studied exchange correlation functionals with the def2-TZVPP basis set; Table S4: Vertical excitation energies for 2O calculated at the DFT/TDDFT/def2-TZVPP level of theory in vacuum, as well as in acetonitrile and 1,4-dioxane using the COSMO model of solvation (for MN15, the calculations were also done using the PCM model for comparative purposes); Figure S1: Schematic drawing of the molecular structure of bifuran (2O), tertfuran (3O), and quaterfuran (4O); Figure S2: Deviations from the experimental values of the vertical absorption energies calculated using all the studied theoretical methods with the def2-TZVPP basis set; Figure S3: Deviations from the experimental values of the vertical emission energies calculated using all the studied theoretical methods with the def2-TZVPP basis set; Figure S4: Deviations from the experimental values of the averaged reorganization energies: 0.5∗(*λ_gr_* + *λ_ex_*), calculated using all the studied theoretical methods with the def2-TZVPP basis set. The experimental reference values have been estimated as 0.5∗(*E^v.abs.^* − *E^v.em.^*); Figure S5: Comparison of the absorption and emission spectrum of 2O obtained at the CC2/def2-TZVPP level of theory with and without the Dushinsky rotation of the normal modes.

## Sample Availability

Not available.
