# Supplementary material for: Optical Spectra of Oligofurans: A Theoretical Approach to the Transition Energies, Reorganization Energies, and the Vibronic Activity
Source: Molecules. 2021 Nov 26;26(23):7163. doi: 10.3390/molecules26237163 (PMC8659192; doi:10.3390/molecules26237163)

Table S1. Optimized cartesian coordinates for the studied oligofurans calculated using the selected theoretical methods (exchange-correlation DFT functionals) and CC2 with the def2-TZVPP basis set.

Part 1.

**2O/BP**	**Ground state**			**Excited state**		
C	-2.8300959	0.4365768	0.0000000	-2.8224440	0.4476766	0.0000000
C	-2.8079253	-0.9290084	0.0000000	-2.7928955	-0.9427104	0.0000000
C	-1.4317100	-1.3123968	0.0000000	-1.4462633	-1.3356537	0.0000000
C	-0.7027945	-0.1446012	0.0000000	-0.6845698	-0.1335271	0.0000000
C	0.7027945	0.1446012	0.0000000	0.6845698	0.1335271	0.0000000
C	1.4317100	1.3123968	0.0000000	1.4462633	1.3356537	0.0000000
C	2.8079253	0.9290084	0.0000000	2.7928955	0.9427104	0.0000000
C	2.8300959	-0.4365768	0.0000000	2.8224440	-0.4476766	0.0000000
O	-1.5540498	0.9365544	0.0000000	-1.5473754	0.9759807	0.0000000
O	1.5540498	-0.9365544	0.0000000	1.5473754	-0.9759807	0.0000000
H	-3.6215146	1.1757085	0.0000000	-3.6291854	1.1715651	0.0000000
H	-3.6715531	-1.5848992	0.0000000	-3.6657851	-1.5873117	0.0000000
H	-1.0235539	-2.3162402	0.0000000	-1.0336319	-2.3375854	0.0000000
H	1.0235539	2.3162402	0.0000000	1.0336319	2.3375854	0.0000000
H	3.6715531	1.5848992	0.0000000	3.6657851	1.5873117	0.0000000
H	3.6215146	-1.1757085	0.0000000	3.6291854	-1.1715651	0.0000000
**3O/BP**	**Ground state**			**Excited state**		
O	3.4893371	0.0000000	0.8468783	3.4949827	0.0000000	0.8618028
C	4.5905360	0.0000000	0.0313440	4.5801514	0.0000000	0.0181425
C	4.2131964	0.0000000	-1.2816386	4.1764241	0.0000000	-1.3001209
C	2.7854336	0.0000000	-1.2938134	2.7641562	0.0000000	-1.3015294
C	2.3848704	0.0000000	0.0247082	2.3682678	0.0000000	0.0472240
C	1.1067417	0.0000000	0.6699482	1.1123126	0.0000000	0.6759543
O	0.0000000	0.0000000	-0.1489916	0.0000000	0.0000000	-0.1642063
C	-1.1067417	0.0000000	0.6699482	-1.1123126	0.0000000	0.6759543
C	-0.7102443	0.0000000	1.9907970	-0.6993405	0.0000000	2.0230545
C	0.7102443	0.0000000	1.9907970	0.6993405	0.0000000	2.0230545
C	-2.3848704	0.0000000	0.0247082	-2.3682678	0.0000000	0.0472240
O	-3.4893371	0.0000000	0.8468783	-3.4949827	0.0000000	0.8618028
C	-4.5905360	0.0000000	0.0313440	-4.5801514	0.0000000	0.0181425
C	-4.2131964	0.0000000	-1.2816386	-4.1764241	0.0000000	-1.3001209
C	-2.7854336	0.0000000	-1.2938134	-2.7641562	0.0000000	-1.3015294
H	5.5474170	0.0000000	0.5383374	5.5496609	0.0000000	0.5018140
H	4.8763602	0.0000000	-2.1396881	4.8338507	0.0000000	-2.1632994
H	2.1307170	0.0000000	-2.1572215	2.0952029	0.0000000	-2.1542637
H	-2.1307170	0.0000000	-2.1572215	-2.0952029	0.0000000	-2.1542637
H	-5.5474170	0.0000000	0.5383374	-5.5496609	0.0000000	0.5018140
H	-4.8763602	0.0000000	-2.1396881	-4.8338507	0.0000000	-2.1632994
H	1.3709898	0.0000000	2.8498467	1.3650126	0.0000000	2.8783268
H	-1.3709898	0.0000000	2.8498467	-1.3650126	0.0000000	2.8783268
**4O/BP**	**Ground state**			**Excited state**		
O	-1.7785469	0.3587455	0.0000000	-1.7791961	0.3793094	0.0000000
C	-2.8173427	-0.5446709	0.0000000	-2.8196837	-0.5396912	0.0000000
C	-2.3181112	-1.8309539	0.0000000	-2.3038268	-1.8413363	0.0000000
C	-0.9031682	-1.7203703	0.0000000	-0.9059014	-1.7305322	0.0000000

C	-0.6100044	-0.3710587	0.0000000	-0.6042575	-0.3601624	0.0000000
C	0.6100044	0.3710587	0.0000000	0.6042575	0.3601624	0.0000000
O	1.7785469	-0.3587455	0.0000000	1.7791961	-0.3793094	0.0000000
C	2.8173427	0.5446709	0.0000000	2.8196837	0.5396912	0.0000000
C	2.3181112	1.8309539	0.0000000	2.3038268	1.8413363	0.0000000
C	0.9031682	1.7203703	0.0000000	0.9059014	1.7305322	0.0000000
C	4.1420467	0.0029759	0.0000000	4.1292217	0.0158639	0.0000000
O	5.1781125	0.9100662	0.0000000	5.1763784	0.9241844	0.0000000
C	6.3402278	0.1841341	0.0000000	6.3333711	0.1864035	0.0000000
C	6.0679739	-1.1547040	0.0000000	6.0515427	-1.1591208	0.0000000
C	4.6458754	-1.2799287	0.0000000	4.6408129	-1.2844687	0.0000000
H	-2.9098310	-2.7389447	0.0000000	-2.8971320	-2.7484101	0.0000000
H	-0.1780123	-2.5256989	0.0000000	-0.1757460	-2.5315761	0.0000000
H	4.0616357	-2.1925004	0.0000000	4.0528188	-2.1947798	0.0000000
H	7.2540659	0.7651467	0.0000000	7.2535853	0.7579792	0.0000000
H	6.7970019	-1.9575359	0.0000000	6.7816266	-1.9616056	0.0000000
H	0.1780123	2.5256989	0.0000000	0.1757460	2.5315761	0.0000000
H	2.9098310	2.7389447	0.0000000	2.8971320	2.7484101	0.0000000
C	-4.1420467	-0.0029759	0.0000000	-4.1292217	-0.0158639	0.0000000
O	-5.1781125	-0.9100662	0.0000000	-5.1763784	-0.9241844	0.0000000
C	-6.3402278	-0.1841341	0.0000000	-6.3333711	-0.1864035	0.0000000
C	-6.0679739	1.1547040	0.0000000	-6.0515427	1.1591208	0.0000000
C	-4.6458754	1.2799287	0.0000000	-4.6408129	1.2844687	0.0000000
H	-4.0616357	2.1925004	0.0000000	-4.0528188	2.1947798	0.0000000
H	-7.2540659	-0.7651467	0.0000000	-7.2535853	-0.7579792	0.0000000
H	-6.7970019	1.9575359	0.0000000	-6.7816266	1.9616056	0.0000000
**5O/BP**	**Ground state**			**Excited state**		
O	3.4891881	0.0000000	0.2790949	3.4885593	0.0000000	0.3023461
C	4.5960688	0.0000000	-0.5393788	4.5959260	0.0000000	-0.5293016
C	4.2000867	0.0000000	-1.8613207	4.1834120	0.0000000	-1.8633945
C	2.7811622	0.0000000	-1.8631617	2.7796553	0.0000000	-1.8605168
C	2.3818733	0.0000000	-0.5409943	2.3744713	0.0000000	-0.5211837
C	1.1074582	0.0000000	0.1016384	1.1110319	0.0000000	0.1041543
O	0.0000000	0.0000000	-0.7177990	0.0000000	0.0000000	-0.7250302
C	-1.1074582	0.0000000	0.1016384	-1.1110319	0.0000000	0.1041543
C	-0.7089569	0.0000000	1.4244841	-0.7023845	0.0000000	1.4413089
C	0.7089569	0.0000000	1.4244841	0.7023845	0.0000000	1.4413089
C	-2.3818733	0.0000000	-0.5409943	-2.3744713	0.0000000	-0.5211837
O	-3.4891881	0.0000000	0.2790949	-3.4885593	0.0000000	0.3023461
C	-4.5960688	0.0000000	-0.5393788	-4.5959260	0.0000000	-0.5293016
C	-4.2000867	0.0000000	-1.8613207	-4.1834120	0.0000000	-1.8633945
C	-2.7811622	0.0000000	-1.8631617	-2.7796553	0.0000000	-1.8605168
C	5.8738210	0.0000000	0.1049865	5.8652768	0.0000000	0.0930487
O	6.9781829	0.0000000	-0.7176350	6.9736315	0.0000000	-0.7362722
C	8.0793919	0.0000000	0.0976371	8.0750730	0.0000000	0.0804499
C	7.7024751	0.0000000	1.4108994	7.6986801	0.0000000	1.4004254
C	6.2750051	0.0000000	1.4236704	6.2802090	0.0000000	1.4227767
C	-5.8738210	0.0000000	0.1049865	-5.8652768	0.0000000	0.0930487
O	-6.9781829	0.0000000	-0.7176350	-6.9736315	0.0000000	-0.7362722
H	-4.8618489	0.0000000	-2.7196034	-4.8438754	0.0000000	-2.7228881
H	-2.1220997	0.0000000	-2.7234201	-2.1147036	0.0000000	-2.7165082
H	-1.3690953	0.0000000	2.2839360	-1.3651843	0.0000000	2.2987701

H	1.3690953	0.0000000	2.2839360	1.3651843	0.0000000	2.2987701
H	2.1220997	0.0000000	-2.7234201	2.1147036	0.0000000	-2.7165082
H	4.8618489	0.0000000	-2.7196034	4.8438754	0.0000000	-2.7228881
H	5.6206829	0.0000000	2.2873754	5.6290259	0.0000000	2.2889010
H	8.3659827	0.0000000	2.2686700	8.3682297	0.0000000	2.2539395
C	-8.0793919	0.0000000	0.0976371	-8.0750730	0.0000000	0.0804499
C	-7.7024751	0.0000000	1.4108994	-7.6986801	0.0000000	1.4004254
C	-6.2750051	0.0000000	1.4236704	-6.2802090	0.0000000	1.4227767
H	-5.6206829	0.0000000	2.2873754	-5.6290259	0.0000000	2.2889010
H	-9.0362001	0.0000000	-0.4095103	-9.0330422	0.0000000	-0.4250721
H	-8.3659827	0.0000000	2.2686700	-8.3682297	0.0000000	2.2539395
H	9.0362001	0.0000000	-0.4095103	9.0330422	0.0000000	-0.4250721
**6O/BP**	**Ground state**			**Excited state**		
O	1.8144441	-0.0058103	0.0000000	1.8174727	0.0050666	0.0000000
C	0.5226866	-0.4853221	0.0000000	0.5202788	-0.4775213	0.0000000
C	0.5381572	-1.8671772	0.0000000	0.5374649	-1.8731743	0.0000000
C	1.8999293	-2.2609314	0.0000000	1.8866074	-2.2673611	0.0000000
C	2.6504714	-1.1007336	0.0000000	2.6526298	-1.0999436	0.0000000
C	4.0531634	-0.8379807	0.0000000	4.0414268	-0.8534460	0.0000000
O	4.8887239	-1.9337264	0.0000000	4.8804739	-1.9545379	0.0000000
C	6.1795192	-1.4554076	0.0000000	6.1752427	-1.4688930	0.0000000
C	6.1668590	-0.0754144	0.0000000	6.1541360	-0.0748859	0.0000000
C	4.8045382	0.3210050	0.0000000	4.8052504	0.3167286	0.0000000
H	7.0412530	0.5649050	0.0000000	7.0290302	0.5651122	0.0000000
H	2.2953414	-3.2699357	0.0000000	2.2814509	-3.2766243	0.0000000
H	-0.3347158	-2.5094677	0.0000000	-0.3387679	-2.5110667	0.0000000
C	7.2276699	-2.4296342	0.0000000	7.2214438	-2.4232609	0.0000000
O	8.5172213	-1.9465368	0.0000000	8.5154943	-1.9353032	0.0000000
C	9.3482587	-3.0358545	0.0000000	9.3470135	-3.0249796	0.0000000
C	8.6210347	-4.1925384	0.0000000	8.6196057	-4.1873308	0.0000000
C	7.2463405	-3.8078845	0.0000000	7.2494914	-3.8130562	0.0000000
H	6.3776698	-4.4556005	0.0000000	6.3833214	-4.4640931	0.0000000
H	10.4083599	-2.8147906	0.0000000	10.4074775	-2.8047002	0.0000000
H	9.0198570	-5.2009758	0.0000000	9.0246968	-5.1935647	0.0000000
C	-0.5226866	0.4853221	0.0000000	-0.5202788	0.4775213	0.0000000
O	-1.8144441	0.0058103	0.0000000	-1.8174727	-0.0050666	0.0000000
C	-2.6504714	1.1007336	0.0000000	-2.6526298	1.0999436	0.0000000
C	-1.8999293	2.2609314	0.0000000	-1.8866074	2.2673611	0.0000000
C	-0.5381572	1.8671772	0.0000000	-0.5374649	1.8731743	0.0000000
H	-2.2953414	3.2699357	0.0000000	-2.2814509	3.2766243	0.0000000
H	0.3347158	2.5094677	0.0000000	0.3387679	2.5110667	0.0000000
H	4.4107932	1.3306459	0.0000000	4.4082911	1.3253119	0.0000000
C	-4.0531634	0.8379807	0.0000000	-4.0414268	0.8534460	0.0000000
C	-4.8045382	-0.3210050	0.0000000	-4.8052504	-0.3167286	0.0000000
C	-6.1668590	0.0754144	0.0000000	-6.1541360	0.0748859	0.0000000
C	-6.1795192	1.4554076	0.0000000	-6.1752427	1.4688930	0.0000000
O	-4.8887239	1.9337264	0.0000000	-4.8804739	1.9545379	0.0000000
H	-7.0412530	-0.5649050	0.0000000	-7.0290302	-0.5651122	0.0000000
H	-4.4107932	-1.3306459	0.0000000	-4.4082911	-1.3253119	0.0000000
C	-7.2276699	2.4296342	0.0000000	-7.2214438	2.4232609	0.0000000
C	-7.2463405	3.8078845	0.0000000	-7.2494914	3.8130562	0.0000000
C	-8.6210347	4.1925384	0.0000000	-8.6196057	4.1873308	0.0000000

C	-9.3482587	3.0358545	0.0000000	-9.3470135	3.0249796	0.0000000
O	-8.5172213	1.9465368	0.0000000	-8.5154943	1.9353032	0.0000000
H	-9.0198570	5.2009758	0.0000000	-9.0246968	5.1935647	0.0000000
H	-10.408359	2.8147906	0.0000000	-10.407477	2.8047002	0.0000000
H	-6.3776698	4.4556005	0.0000000	-6.3833214	4.4640931	0.0000000
**7O/BP**	**Ground state**			**Excited state**		
C	-9.7639698	0.0000000	-1.4990653	-9.7675455	0.0000000	-1.5027171
C	-9.3626496	0.0000000	-0.1804034	-9.3554743	0.0000000	-0.1773665
O	-10.466916	0.0000000	0.6423464	-10.461958	0.0000000	0.6506286
C	-11.568147	0.0000000	-0.1728036	-11.563899	0.0000000	-0.1640661
C	-11.191391	0.0000000	-1.4861377	-11.188993	0.0000000	-1.4819771
C	-8.0849004	0.0000000	0.4638409	-8.0840956	0.0000000	0.4509105
O	-6.9780591	0.0000000	-0.3545556	-6.9752063	0.0000000	-0.3730026
C	-5.8707487	0.0000000	0.4656494	-5.8638633	0.0000000	0.4514860
C	-6.2702206	0.0000000	1.7878776	-6.2734078	0.0000000	1.7861728
C	-7.6890015	0.0000000	1.7859162	-7.6789091	0.0000000	1.7829916
C	-4.5964807	0.0000000	-0.1767396	-4.5977651	0.0000000	-0.1709043
O	-3.4889524	0.0000000	0.6424465	-3.4880701	0.0000000	0.6566229
C	-2.3814866	0.0000000	-0.1774619	-2.3766200	0.0000000	-0.1665088
C	-2.7807860	0.0000000	-1.5005597	-2.7816084	0.0000000	-1.5014543
C	-4.1982337	0.0000000	-1.4999909	-4.1867431	0.0000000	-1.5046451
C	-1.1075419	0.0000000	0.4639884	-1.1102991	0.0000000	0.4604624
O	0.0000000	0.0000000	-0.3556499	0.0000000	0.0000000	-0.3648840
C	1.1075419	0.0000000	0.4639884	1.1102991	0.0000000	0.4604624
C	0.7085952	0.0000000	1.7873419	0.7035160	0.0000000	1.7937882
C	-0.7085952	0.0000000	1.7873419	-0.7035160	0.0000000	1.7937882
C	2.3814866	0.0000000	-0.1774619	2.3766200	0.0000000	-0.1665088
O	3.4889524	0.0000000	0.6424465	3.4880701	0.0000000	0.6566229
C	4.5964807	0.0000000	-0.1767396	4.5977651	0.0000000	-0.1709043
C	4.1982337	0.0000000	-1.4999909	4.1867431	0.0000000	-1.5046451
C	2.7807860	0.0000000	-1.5005597	2.7816084	0.0000000	-1.5014543
C	5.8707487	0.0000000	0.4656494	5.8638633	0.0000000	0.4514860
O	6.9780591	0.0000000	-0.3545556	6.9752063	0.0000000	-0.3730026
C	8.0849004	0.0000000	0.4638409	8.0840956	0.0000000	0.4509105
C	7.6890015	0.0000000	1.7859162	7.6789091	0.0000000	1.7829916
C	6.2702206	0.0000000	1.7878776	6.2734078	0.0000000	1.7861728
C	9.3626496	0.0000000	-0.1804034	9.3554743	0.0000000	-0.1773665
C	9.7639698	0.0000000	-1.4990653	9.7675455	0.0000000	-1.5027171
C	11.1913910	0.0000000	-1.4861377	11.1889937	0.0000000	-1.4819771
C	11.5681477	0.0000000	-0.1728036	11.5638996	0.0000000	-0.1640661
O	10.4669167	0.0000000	0.6423464	10.4619586	0.0000000	0.6506286
H	-1.3686883	0.0000000	2.6468317	-1.3652130	0.0000000	2.6521076
H	2.1210394	0.0000000	-2.3603167	2.1179238	0.0000000	-2.3583681
H	4.8587085	0.0000000	-2.3591822	4.8464219	0.0000000	-2.3645440
H	-2.1210394	0.0000000	-2.3603167	-2.1179238	0.0000000	-2.3583681
H	-4.8587085	0.0000000	-2.3591822	-4.8464219	0.0000000	-2.3645440
H	8.3508522	0.0000000	2.6441256	8.3423191	0.0000000	2.6402907
H	5.6112709	0.0000000	2.6482253	5.6133264	0.0000000	2.6458265
H	1.3686883	0.0000000	2.6468317	1.3652130	0.0000000	2.6521076
H	11.8549982	0.0000000	-2.3438247	11.8574301	0.0000000	-2.3361782
H	9.1097630	0.0000000	-2.3628592	9.1165512	0.0000000	-2.3688871
H	-5.6112709	0.0000000	2.6482253	-5.6133264	0.0000000	2.6458265

H	-8.3508522	0.0000000	2.6441256	-8.3423191	0.0000000	2.6402907
H	-9.1097630	0.0000000	-2.3628592	-9.1165512	0.0000000	-2.3688871
H	-11.854998	0.0000000	-2.3438247	-11.857430	0.0000000	-2.3361782
H	-12.524913	0.0000000	0.3344288	-12.520885	0.0000000	0.3430667
H	12.5249135	0.0000000	0.3344288	12.5208850	0.0000000	0.3430667
**8O/BP**	**Ground state**			**Excited state**		
O	-11.9493588	2.5647623	0.0000000	-11.945986	2.5577313	0.0000000
C	-10.6746677	3.0857915	0.0000000	-10.668491	3.0826894	0.0000000
C	-10.7340802	4.4628900	0.0000000	-10.735492	4.4673015	0.0000000
C	-12.1195241	4.8067166	0.0000000	-12.117858	4.8033367	0.0000000
C	-12.8122125	3.6290109	0.0000000	-12.809659	3.6214771	0.0000000
C	-9.5982033	2.1429721	0.0000000	-9.5939216	2.1544629	0.0000000
C	-9.5448983	0.7639049	0.0000000	-9.5358081	0.7649811	0.0000000
C	-8.1715439	0.4078201	0.0000000	-8.1748201	0.4095330	0.0000000
C	-7.4546279	1.5884621	0.0000000	-7.4450903	1.5982721	0.0000000
O	-8.3221193	2.6591311	0.0000000	-8.3149142	2.6738773	0.0000000
C	-6.0603645	1.8924365	0.0000000	-6.0631910	1.8850820	0.0000000
O	-5.1923381	0.8227923	0.0000000	-5.1945700	0.8081320	0.0000000
C	-3.9152848	1.3404060	0.0000000	-3.9131548	1.3276928	0.0000000
C	-3.9717855	2.7213128	0.0000000	-3.9734098	2.7213892	0.0000000
C	-5.3444867	3.0744740	0.0000000	-5.3331236	3.0739239	0.0000000
C	-2.8417429	0.4013992	0.0000000	-2.8426265	0.4060787	0.0000000
O	-1.5647153	0.9186454	0.0000000	-1.5617746	0.9284299	0.0000000
C	-0.6967728	-0.1515369	0.0000000	-0.6919690	-0.1460501	0.0000000
C	-1.4135252	-1.3334725	0.0000000	-1.4168655	-1.3355828	0.0000000
C	-2.7857448	-0.9797336	0.0000000	-2.7797971	-0.9861836	0.0000000
C	0.6967728	0.1515369	0.0000000	0.6919690	0.1460501	0.0000000
O	1.5647153	-0.9186454	0.0000000	1.5617746	-0.9284299	0.0000000
C	2.8417429	-0.4013992	0.0000000	2.8426265	-0.4060787	0.0000000
C	2.7857448	0.9797336	0.0000000	2.7797971	0.9861836	0.0000000
C	1.4135252	1.3334725	0.0000000	1.4168655	1.3355828	0.0000000
C	3.9152848	-1.3404060	0.0000000	3.9131548	-1.3276928	0.0000000
O	5.1923381	-0.8227923	0.0000000	5.1945700	-0.8081320	0.0000000
C	6.0603645	-1.8924365	0.0000000	6.0631910	-1.8850820	0.0000000
C	5.3444867	-3.0744740	0.0000000	5.3331236	-3.0739239	0.0000000
C	3.9717855	-2.7213128	0.0000000	3.9734098	-2.7213892	0.0000000
H	3.6395061	1.6472173	0.0000000	3.6337308	1.6535270	0.0000000
H	-0.9888915	-2.3305362	0.0000000	-0.9894216	-2.3315460	0.0000000
H	-3.6395061	-1.6472173	0.0000000	-3.6337308	-1.6535270	0.0000000
H	3.1183649	-3.3892384	0.0000000	3.1175824	-3.3864812	0.0000000
C	7.4546279	-1.5884621	0.0000000	7.4450903	-1.5982721	0.0000000
H	5.7696639	-4.0712976	0.0000000	5.7577766	-4.0711082	0.0000000
H	-5.7696639	4.0712976	0.0000000	-5.7577766	4.0711082	0.0000000
H	-3.1183649	3.3892384	0.0000000	-3.1175824	3.3864812	0.0000000
H	0.9888915	2.3305362	0.0000000	0.9894216	2.3315460	0.0000000
H	-10.4000470	0.0981059	0.0000000	-10.392664	0.1010028	0.0000000
H	-7.7482430	-0.5897929	0.0000000	-7.7513965	-0.5881284	0.0000000
H	-12.5480098	5.8029086	0.0000000	-12.551145	5.7976320	0.0000000
H	-13.8653146	3.3766959	0.0000000	-13.862850	3.3690411	0.0000000
H	-9.8849489	5.1360198	0.0000000	-9.8886256	5.1432019	0.0000000
O	8.3221193	-2.6591311	0.0000000	8.3149142	-2.6738773	0.0000000
C	9.5982033	-2.1429721	0.0000000	9.5939216	-2.1544629	0.0000000

C	9.5448983	-0.7639049	0.0000000	9.5358081	-0.7649811	0.0000000
C	8.1715439	-0.4078201	0.0000000	8.1748201	-0.4095330	0.0000000
H	7.7482430	0.5897929	0.0000000	7.7513965	0.5881284	0.0000000
H	10.4000470	-0.0981059	0.0000000	10.3926647	-0.1010028	0.0000000
C	10.6746677	-3.0857915	0.0000000	10.6684916	-3.0826894	0.0000000
O	11.9493588	-2.5647623	0.0000000	11.9459867	-2.5577313	0.0000000
C	12.8122125	-3.6290109	0.0000000	12.8096595	-3.6214771	0.0000000
C	12.1195241	-4.8067166	0.0000000	12.1178584	-4.8033367	0.0000000
C	10.7340802	-4.4628900	0.0000000	10.7354929	-4.4673015	0.0000000
H	9.8849489	-5.1360198	0.0000000	9.8886256	-5.1432019	0.0000000
H	12.5480098	-5.8029086	0.0000000	12.5511452	-5.7976320	0.0000000
H	13.8653146	-3.3766959	0.0000000	13.8628503	-3.3690411	0.0000000
**9O/BP**	**Ground state**			**Excited state**		
C	-13.2528363	0.0000000	-1.5354357	-13.254085	0.0000000	-1.5391724
C	-12.8514250	0.0000000	-0.2168026	-12.844958	0.0000000	-0.2161265
O	-13.9556198	0.0000000	0.6060341	-13.950593	0.0000000	0.6104383
C	-15.0568884	0.0000000	-0.2090272	-15.052364	0.0000000	-0.2039517
C	-14.6802414	0.0000000	-1.5224021	-14.677406	0.0000000	-1.5204645
C	-11.5736461	0.0000000	0.4273678	-11.571904	0.0000000	0.4164978
O	-10.4668510	0.0000000	-0.3910352	-10.463219	0.0000000	-0.4046309
C	-9.3595296	0.0000000	0.4291635	-9.3528978	0.0000000	0.4195647
C	-9.7589848	0.0000000	1.7514017	-9.7625000	0.0000000	1.7515329
C	-11.1777375	0.0000000	1.7494658	-11.170367	0.0000000	1.7464176
C	-8.0853048	0.0000000	-0.2132556	-8.0857985	0.0000000	-0.2040412
O	-6.9777423	0.0000000	0.6058072	-6.9757950	0.0000000	0.6208413
C	-5.8703463	0.0000000	-0.2142411	-5.8648103	0.0000000	-0.2021112
C	-6.2698099	0.0000000	-1.5373193	-6.2720465	0.0000000	-1.5363218
C	-7.6871965	0.0000000	-1.5366027	-7.6766371	0.0000000	-1.5370485
C	-4.5964111	0.0000000	0.4270519	-4.5980775	0.0000000	0.4218090
O	-3.4888988	0.0000000	-0.3925168	-3.4880815	0.0000000	-0.4034736
C	-2.3813386	0.0000000	0.4272372	-2.3775730	0.0000000	0.4191367
C	-2.7804845	0.0000000	1.7506607	-2.7826046	0.0000000	1.7519698
C	-4.1975190	0.0000000	1.7505482	-4.1889250	0.0000000	1.7542264
C	-1.1075512	0.0000000	-0.2139450	-1.1100014	0.0000000	-0.2084973
O	0.0000000	0.0000000	0.6057147	0.0000000	0.0000000	0.6152514
C	1.1075512	0.0000000	-0.2139450	1.1100014	0.0000000	-0.2084973
C	0.7084848	0.0000000	-1.5374580	0.7038785	0.0000000	-1.5402198
C	-0.7084848	0.0000000	-1.5374580	-0.7038785	0.0000000	-1.5402198
C	2.3813386	0.0000000	0.4272372	2.3775730	0.0000000	0.4191367
O	3.4888988	0.0000000	-0.3925168	3.4880815	0.0000000	-0.4034736
C	4.5964111	0.0000000	0.4270519	4.5980775	0.0000000	0.4218090
C	4.1975190	0.0000000	1.7505482	4.1889250	0.0000000	1.7542264
C	2.7804845	0.0000000	1.7506607	2.7826046	0.0000000	1.7519698
C	5.8703463	0.0000000	-0.2142411	5.8648103	0.0000000	-0.2021112
C	6.2698099	0.0000000	-1.5373193	6.2720465	0.0000000	-1.5363218
C	7.6871965	0.0000000	-1.5366027	7.6766371	0.0000000	-1.5370485
C	8.0853048	0.0000000	-0.2132556	8.0857985	0.0000000	-0.2040412
O	6.9777423	0.0000000	0.6058072	6.9757950	0.0000000	0.6208413
C	9.3595296	0.0000000	0.4291635	9.3528978	0.0000000	0.4195647
O	10.4668510	0.0000000	-0.3910352	10.4632194	0.0000000	-0.4046309
C	11.5736461	0.0000000	0.4273678	11.5719048	0.0000000	0.4164978
C	11.1777375	0.0000000	1.7494658	11.1703677	0.0000000	1.7464176

C	9.7589848	0.0000000	1.7514017	9.7625000	0.0000000	1.7515329
H	-4.8576949	0.0000000	2.6099700	-4.8488221	0.0000000	2.6139630
H	-1.3685747	0.0000000	-2.3969507	-1.3650879	0.0000000	-2.3989364
H	1.3685747	0.0000000	-2.3969507	1.3650879	0.0000000	-2.3989364
H	-5.6101754	0.0000000	-2.3971657	-5.6101710	0.0000000	-2.3946189
H	-8.3477658	0.0000000	-2.3957174	-8.3372817	0.0000000	-2.3963136
H	4.8576949	0.0000000	2.6099700	4.8488221	0.0000000	2.6139630
H	2.1204892	0.0000000	2.6102296	2.1200195	0.0000000	2.6096964
H	-2.1204892	0.0000000	2.6102296	-2.1200195	0.0000000	2.6096964
H	8.3477658	0.0000000	-2.3957174	8.3372817	0.0000000	-2.3963136
H	5.6101754	0.0000000	-2.3971657	5.6101710	0.0000000	-2.3946189
H	11.8395799	0.0000000	2.6076786	11.8346081	0.0000000	2.6030520
C	12.8514250	0.0000000	-0.2168026	12.8449582	0.0000000	-0.2161265
H	9.1000355	0.0000000	2.6117514	9.1040057	0.0000000	2.6123031
H	-9.1000355	0.0000000	2.6117514	-9.1040057	0.0000000	2.6123031
H	-11.839579	0.0000000	2.6076786	-11.834608	0.0000000	2.6030520
H	-12.5987036	0.0000000	-2.3992869	-12.602384	0.0000000	-2.4047981
H	-15.3439155	0.0000000	-2.3800346	-15.344583	0.0000000	-2.3755378
H	-16.0136204	0.0000000	0.2982712	-16.009123	0.0000000	0.3034890
O	13.9556198	0.0000000	0.6060341	13.9505935	0.0000000	0.6104383
C	15.0568884	0.0000000	-0.2090272	15.0523644	0.0000000	-0.2039517
C	14.6802414	0.0000000	-1.5224021	14.6774063	0.0000000	-1.5204645
C	13.2528363	0.0000000	-1.5354357	13.2540855	0.0000000	-1.5391724
H	12.5987036	0.0000000	-2.3992869	12.6023846	0.0000000	-2.4047981
H	15.3439155	0.0000000	-2.3800346	15.3445835	0.0000000	-2.3755378
H	16.0136204	0.0000000	0.2982712	16.0091233	0.0000000	0.3034890

Part 2.

**2O/ TPSSh**	**Ground state**			**Excited state**		
C	-2.8183862	0.4334075	0.0000000	-2.8084512	0.4449548	0.0000000
C	-2.7980694	-0.9242583	0.0000000	-2.7800261	-0.9384330	0.0000000
C	-1.4245153	-1.3069473	0.0000000	-1.4404738	-1.3321118	0.0000000
C	-0.7018155	-0.1455675	0.0000000	-0.6797095	-0.1326912	0.0000000
C	0.7018155	0.1455675	0.0000000	0.6797095	0.1326912	0.0000000
C	1.4245153	1.3069473	0.0000000	1.4404738	1.3321118	0.0000000
C	2.7980694	0.9242583	0.0000000	2.7800261	0.9384330	0.0000000
C	2.8183862	-0.4334075	0.0000000	2.8084512	-0.4449548	0.0000000
O	-1.5481839	0.9292588	0.0000000	-1.5409844	0.9687817	0.0000000
O	1.5481839	-0.9292588	0.0000000	1.5409844	-0.9687817	0.0000000
H	-3.6035178	1.1692253	0.0000000	-3.6100628	1.1643397	0.0000000
H	-3.6569230	-1.5756293	0.0000000	-3.6492779	-1.5770079	0.0000000
H	-1.0181188	-2.3044725	0.0000000	-1.0298963	-2.3278064	0.0000000
H	1.0181188	2.3044725	0.0000000	1.0298963	2.3278064	0.0000000
H	3.6569230	1.5756293	0.0000000	3.6492779	1.5770079	0.0000000
H	3.6035178	-1.1692253	0.0000000	3.6100628	-1.1643397	0.0000000
**3O/ TPSSh**	**Ground state**			**Excited state**		
O	3.4749960	0.0000000	0.8393779	3.4786274	0.0000000	0.8556377

C	4.5709333	0.0000000	0.0284136	4.5590116	0.0000000	0.0192121
C	4.1962749	0.0000000	-1.2770268	4.1605691	0.0000000	-1.2923799
C	2.7711738	0.0000000	-1.2878112	2.7525260	0.0000000	-1.2965567
C	2.3762803	0.0000000	0.0231454	2.3573282	0.0000000	0.0462034
C	1.1009696	0.0000000	0.6709683	1.1078560	0.0000000	0.6694023
O	0.0000000	0.0000000	-0.1426157	0.0000000	0.0000000	-0.1654335
C	-1.1009696	0.0000000	0.6709683	-1.1078560	0.0000000	0.6694023
C	-0.7091193	0.0000000	1.9838914	-0.6939836	0.0000000	2.0141465
C	0.7091193	0.0000000	1.9838914	0.6939836	0.0000000	2.0141465
C	-2.3762803	0.0000000	0.0231454	-2.3573282	0.0000000	0.0462034
O	-3.4749960	0.0000000	0.8393779	-3.4786274	0.0000000	0.8556377
C	-4.5709333	0.0000000	0.0284136	-4.5590116	0.0000000	0.0192121
C	-4.1962749	0.0000000	-1.2770268	-4.1605691	0.0000000	-1.2923799
C	-2.7711738	0.0000000	-1.2878112	-2.7525260	0.0000000	-1.2965567
H	5.5213076	0.0000000	0.5330248	5.5214519	0.0000000	0.5017625
H	4.8552345	0.0000000	-2.1300596	4.8163561	0.0000000	-2.1485825
H	2.1188255	0.0000000	-2.1448858	2.0877986	0.0000000	-2.1443771
H	-2.1188255	0.0000000	-2.1448858	-2.0877986	0.0000000	-2.1443771
H	-5.5213076	0.0000000	0.5330248	-5.5214519	0.0000000	0.5017625
H	-4.8552345	0.0000000	-2.1300596	-4.8163561	0.0000000	-2.1485825
H	1.3665026	0.0000000	2.8372721	1.3570699	0.0000000	2.8632508
H	-1.3665026	0.0000000	2.8372721	-1.3570699	0.0000000	2.8632508
**4O/ TPSSh**	**Ground state**			**Excited state**		
O	-1.7709338	0.3518479	0.0000000	-1.7715972	0.3791061	0.0000000
C	-2.8041099	-0.5460791	0.0000000	-2.8067382	-0.5341768	0.0000000
C	-2.3097151	-1.8245799	0.0000000	-2.2927166	-1.8316138	0.0000000
C	-0.8969509	-1.7135527	0.0000000	-0.9039843	-1.7244158	0.0000000
C	-0.6084336	-0.3725493	0.0000000	-0.6002125	-0.3556400	0.0000000
C	0.6084336	0.3725493	0.0000000	0.6002125	0.3556400	0.0000000
O	1.7709338	-0.3518479	0.0000000	1.7715972	-0.3791061	0.0000000
C	2.8041099	0.5460791	0.0000000	2.8067382	0.5341768	0.0000000
C	2.3097151	1.8245799	0.0000000	2.2927166	1.8316138	0.0000000
C	0.8969509	1.7135527	0.0000000	0.9039843	1.7244158	0.0000000
C	4.1263916	0.0018573	0.0000000	4.1116515	0.0133113	0.0000000
O	5.1569339	0.9028178	0.0000000	5.1527267	0.9162443	0.0000000
C	6.3136398	0.1813444	0.0000000	6.3049242	0.1856361	0.0000000
C	6.0438038	-1.1498225	0.0000000	6.0282183	-1.1523946	0.0000000
C	4.6242583	-1.2737866	0.0000000	4.6206861	-1.2794994	0.0000000
H	-2.8981604	-2.7268823	0.0000000	-2.8850828	-2.7316406	0.0000000
H	-0.1751811	-2.5131712	0.0000000	-0.1780623	-2.5205402	0.0000000
H	4.0420985	-2.1799972	0.0000000	4.0364415	-2.1845334	0.0000000
H	7.2210124	0.7597305	0.0000000	7.2177463	0.7561530	0.0000000
H	6.7684185	-1.9478262	0.0000000	6.7556550	-1.9484229	0.0000000
H	0.1751811	2.5131712	0.0000000	0.1780623	2.5205402	0.0000000
H	2.8981604	2.7268823	0.0000000	2.8850828	2.7316406	0.0000000
C	-4.1263916	-0.0018573	0.0000000	-4.1116515	-0.0133113	0.0000000
O	-5.1569339	-0.9028178	0.0000000	-5.1527267	-0.9162443	0.0000000
C	-6.3136398	-0.1813444	0.0000000	-6.3049242	-0.1856361	0.0000000

C	-6.0438038	1.1498225	0.0000000	-6.0282183	1.1523946	0.0000000
C	-4.6242583	1.2737866	0.0000000	-4.6206861	1.2794994	0.0000000
H	-4.0420985	2.1799972	0.0000000	-4.0364415	2.1845334	0.0000000
H	-7.2210124	-0.7597305	0.0000000	-7.2177463	-0.7561530	0.0000000
H	-6.7684185	1.9478262	0.0000000	-6.7556550	1.9484229	0.0000000
**5O/ TPSSh**	**Ground state**			**Excited state**		
O	3.4748873	0.0000000	0.2726931	3.4728369	0.0000000	0.2988335
C	4.5758134	0.0000000	-0.5406406	4.5746371	0.0000000	-0.5261926
C	4.1841723	0.0000000	-1.8544522	4.1664975	0.0000000	-1.8544192
C	2.7673449	0.0000000	-1.8557792	2.7697183	0.0000000	-1.8557289
C	2.3732802	0.0000000	-0.5416140	2.3628453	0.0000000	-0.5200704
C	1.1016006	0.0000000	0.1041228	1.1070073	0.0000000	0.0970978
O	0.0000000	0.0000000	-0.7097659	0.0000000	0.0000000	-0.7277878
C	-1.1016006	0.0000000	0.1041228	-1.1070073	0.0000000	0.0970978
C	-0.7079593	0.0000000	1.4188296	-0.6966661	0.0000000	1.4326193
C	0.7079593	0.0000000	1.4188296	0.6966661	0.0000000	1.4326193
C	-2.3732802	0.0000000	-0.5416140	-2.3628453	0.0000000	-0.5200704
O	-3.4748873	0.0000000	0.2726931	-3.4728369	0.0000000	0.2988335
C	-4.5758134	0.0000000	-0.5406406	-4.5746371	0.0000000	-0.5261926
C	-4.1841723	0.0000000	-1.8544522	-4.1664975	0.0000000	-1.8544192
C	-2.7673449	0.0000000	-1.8557792	-2.7697183	0.0000000	-1.8557289
C	5.8509313	0.0000000	0.1061624	5.8402979	0.0000000	0.0964854
O	6.9493116	0.0000000	-0.7107598	6.9425202	0.0000000	-0.7266292
C	8.0454709	0.0000000	0.0996527	8.0390457	0.0000000	0.0838231
C	7.6715538	0.0000000	1.4054448	7.6667857	0.0000000	1.3960565
C	6.2467140	0.0000000	1.4171332	6.2506992	0.0000000	1.4180759
C	-5.8509313	0.0000000	0.1061624	-5.8402979	0.0000000	0.0964854
O	-6.9493116	0.0000000	-0.7107598	-6.9425202	0.0000000	-0.7266292
H	-4.8422850	0.0000000	-2.7072717	-4.8260643	0.0000000	-2.7064868
H	-2.1112568	0.0000000	-2.7101144	-2.1095225	0.0000000	-2.7071545
H	-1.3648079	0.0000000	2.2725889	-1.3568613	0.0000000	2.2840008
H	1.3648079	0.0000000	2.2725889	1.3568613	0.0000000	2.2840008
H	2.1112568	0.0000000	-2.7101144	2.1095225	0.0000000	-2.7071545
H	4.8422850	0.0000000	-2.7072717	4.8260643	0.0000000	-2.7064868
H	5.5949554	0.0000000	2.2746548	5.6024943	0.0000000	2.2783599
H	8.3310443	0.0000000	2.2580504	8.3328056	0.0000000	2.2439941
C	-8.0454709	0.0000000	0.0996527	-8.0390457	0.0000000	0.0838231
C	-7.6715538	0.0000000	1.4054448	-7.6667857	0.0000000	1.3960565
C	-6.2467140	0.0000000	1.4171332	-6.2506992	0.0000000	1.4180759
H	-5.5949554	0.0000000	2.2746548	-5.6024943	0.0000000	2.2783599
H	-8.9956433	0.0000000	-0.4053494	-8.9900111	0.0000000	-0.4203023
H	-8.3310443	0.0000000	2.2580504	-8.3328056	0.0000000	2.2439941
H	8.9956433	0.0000000	-0.4053494	8.9900111	0.0000000	-0.4203023
**6O/ TPSSh**	**Ground state**			**Excited state**		
O	1.8055465	-0.0102666	0.0000000	1.8102479	0.0090146	0.0000000
C	0.5210946	-0.4863451	0.0000000	0.5178960	-0.4712815	0.0000000

C	0.5344173	-1.8589609	0.0000000	0.5387195	-1.8661524	0.0000000
C	1.8943841	-2.2519014	0.0000000	1.8786255	-2.2546018	0.0000000
C	2.6377269	-1.0980407	0.0000000	2.6428584	-1.0896051	0.0000000
C	4.0386660	-0.8313770	0.0000000	4.0241375	-0.8458709	0.0000000
O	4.8702988	-1.9200085	0.0000000	4.8603181	-1.9404755	0.0000000
C	6.1539838	-1.4450774	0.0000000	6.1475449	-1.4597129	0.0000000
C	6.1433059	-0.0741101	0.0000000	6.1294112	-0.0741374	0.0000000
C	4.7828547	0.3213107	0.0000000	4.7860269	0.3191951	0.0000000
H	7.0126496	0.5620109	0.0000000	7.0008108	0.5596729	0.0000000
H	2.2883579	-3.2544584	0.0000000	2.2741665	-3.2567412	0.0000000
H	-0.3335432	-2.4969283	0.0000000	-0.3321420	-2.5004449	0.0000000
C	7.1987360	-2.4211459	0.0000000	7.1902354	-2.4154408	0.0000000
O	8.4811043	-1.9422563	0.0000000	8.4765453	-1.9322065	0.0000000
C	9.3083119	-3.0257740	0.0000000	9.3047176	-3.0149862	0.0000000
C	8.5856765	-4.1758784	0.0000000	8.5828221	-4.1705963	0.0000000
C	7.2139404	-3.7904856	0.0000000	7.2150841	-3.7955259	0.0000000
H	6.3492871	-4.4327522	0.0000000	6.3530577	-4.4413337	0.0000000
H	10.3615258	-2.8053021	0.0000000	10.3581559	-2.7945502	0.0000000
H	8.9817070	-5.1783781	0.0000000	8.9852497	-5.1708139	0.0000000
C	-0.5210946	0.4863451	0.0000000	-0.5178960	0.4712815	0.0000000
O	-1.8055465	0.0102666	0.0000000	-1.8102479	-0.0090146	0.0000000
C	-2.6377269	1.0980407	0.0000000	-2.6428584	1.0896051	0.0000000
C	-1.8943841	2.2519014	0.0000000	-1.8786255	2.2546018	0.0000000
C	-0.5344173	1.8589609	0.0000000	-0.5387195	1.8661524	0.0000000
H	-2.2883579	3.2544584	0.0000000	-2.2741665	3.2567412	0.0000000
H	0.3335432	2.4969283	0.0000000	0.3321420	2.5004449	0.0000000
H	4.3904514	1.3244830	0.0000000	4.3920984	1.3219428	0.0000000
C	-4.0386660	0.8313770	0.0000000	-4.0241375	0.8458709	0.0000000
C	-4.7828547	-0.3213107	0.0000000	-4.7860269	-0.3191951	0.0000000
C	-6.1433059	0.0741101	0.0000000	-6.1294112	0.0741374	0.0000000
C	-6.1539838	1.4450774	0.0000000	-6.1475449	1.4597129	0.0000000
O	-4.8702988	1.9200085	0.0000000	-4.8603181	1.9404755	0.0000000
H	-7.0126496	-0.5620109	0.0000000	-7.0008108	-0.5596729	0.0000000
H	-4.3904514	-1.3244830	0.0000000	-4.3920984	-1.3219428	0.0000000
C	-7.1987360	2.4211459	0.0000000	-7.1902354	2.4154408	0.0000000
C	-7.2139404	3.7904856	0.0000000	-7.2150841	3.7955259	0.0000000
C	-8.5856765	4.1758784	0.0000000	-8.5828221	4.1705963	0.0000000
C	-9.3083119	3.0257740	0.0000000	-9.3047176	3.0149862	0.0000000
O	-8.4811043	1.9422563	0.0000000	-8.4765453	1.9322065	0.0000000
H	-8.9817070	5.1783781	0.0000000	-8.9852497	5.1708139	0.0000000
H	-10.3615258	2.8053021	0.0000000	-10.358155	2.7945502	0.0000000
H	-6.3492871	4.4327522	0.0000000	-6.3530577	4.4413337	0.0000000
**7O/ TPSSh**	**Ground state**			**Excited state**		
C	-9.7217870	0.0000000	-1.4912276	9.720856	0.0000000	1.540989
C	-9.3254584	0.0000000	-0.1804120	9.315849	0.0000000	0.224011
O	-10.4234747	0.0000000	0.6369888	10.416584	0.0000000	-0.596579
C	-11.5199246	0.0000000	-0.1729510	11.512904	0.0000000	0.213288
C	-11.1465844	0.0000000	-1.4789295	11.140391	0.0000000	1.522915

C	-8.0501239	0.0000000	0.4658754	8.047364	0.0000000	-0.408355
O	-6.9494986	0.0000000	-0.3477352	6.944121	0.0000000	0.408173
C	-5.8476422	0.0000000	0.4663153	5.838257	0.0000000	-0.410884
C	-6.2414331	0.0000000	1.7806467	6.246165	0.0000000	-1.738456
C	-7.6581411	0.0000000	1.7796756	7.648454	0.0000000	-1.732472
C	-4.5762936	0.0000000	-0.1796529	4.577071	0.0000000	0.209296
O	-3.4743915	0.0000000	0.6336442	3.472502	0.0000000	-0.612620
C	-2.3730510	0.0000000	-0.1809844	2.364907	0.0000000	0.206013
C	-2.7677759	0.0000000	-1.4957588	2.771717	0.0000000	1.538176
C	-4.1833009	0.0000000	-1.4948313	4.168425	0.0000000	1.538333
C	-1.1016387	0.0000000	0.4634719	1.106417	0.0000000	-0.412137
O	0.0000000	0.0000000	-0.3505933	0.000000	0.0000000	0.409062
C	1.1016387	0.0000000	0.4634719	-1.106417	0.0000000	-0.412137
C	0.7076388	0.0000000	1.7786009	-0.697847	0.0000000	-1.743834
C	-0.7076388	0.0000000	1.7786009	0.697847	0.0000000	-1.743834
C	2.3730510	0.0000000	-0.1809844	-2.364907	0.0000000	0.206013
O	3.4743915	0.0000000	0.6336442	-3.472502	0.0000000	-0.612620
C	4.5762936	0.0000000	-0.1796529	-4.577071	0.0000000	0.209296
C	4.1833009	0.0000000	-1.4948313	-4.168425	0.0000000	1.538333
C	2.7677759	0.0000000	-1.4957588	-2.771717	0.0000000	1.538176
C	5.8476422	0.0000000	0.4663153	-5.838257	0.0000000	-0.410884
O	6.9494986	0.0000000	-0.3477352	-6.944121	0.0000000	0.408173
C	8.0501239	0.0000000	0.4658754	-8.047364	0.0000000	-0.408355
C	7.6581411	0.0000000	1.7796756	-7.648454	0.0000000	-1.732472
C	6.2414331	0.0000000	1.7806467	-6.246165	0.0000000	-1.738456
C	9.3254584	0.0000000	-0.1804120	-9.315849	0.0000000	0.224011
C	9.7217870	0.0000000	-1.4912276	-9.720856	0.0000000	1.540989
C	11.1465844	0.0000000	-1.4789295	-11.140391	0.0000000	1.522915
C	11.5199246	0.0000000	-0.1729510	-11.512904	0.0000000	0.213288
O	10.4234747	0.0000000	0.6369888	-10.416584	0.0000000	-0.596579
H	-1.3644498	0.0000000	2.6323869	1.356825	0.0000000	-2.596159
H	2.1115464	0.0000000	-2.3499996	-2.112278	0.0000000	2.390147
H	4.8406940	0.0000000	-2.3481643	-4.826602	0.0000000	2.391334
H	-2.1115464	0.0000000	-2.3499996	2.112278	0.0000000	2.390147
H	-4.8406940	0.0000000	-2.3481643	4.826602	0.0000000	2.391334
H	8.3160484	0.0000000	2.6326473	-8.310003	0.0000000	-2.582974
H	5.5851711	0.0000000	2.6348519	-5.590705	0.0000000	-2.593417
H	1.3644498	0.0000000	2.6323869	-1.356825	0.0000000	-2.596159
H	11.8064358	0.0000000	-2.3312492	-11.803862	0.0000000	2.372657
H	9.0704225	0.0000000	-2.3490501	-9.071727	0.0000000	2.400490
H	-5.5851711	0.0000000	2.6348519	5.590705	0.0000000	-2.593417
H	-8.3160484	0.0000000	2.6326473	8.310003	0.0000000	-2.582974
H	-9.0704225	0.0000000	-2.3490501	9.071727	0.0000000	2.400490
H	-11.8064358	0.0000000	-2.3312492	11.803862	0.0000000	2.372657
H	-12.4698968	0.0000000	0.3324309	12.463420	0.0000000	-0.291412
H	12.4698968	0.0000000	0.3324309	-12.463420	0.0000000	-0.291412
**8O/ TPSSh**	**Ground state**			**Excited state**		
O	5.057667	11.070292	0.000000	4.935522	11.117223	0.000000

C	5.289589	9.721227	0.000000	5.183935	9.767806	0.000000
C	6.637844	9.481362	0.000000	6.542175	9.547264	0.000000
C	7.271735	10.757418	0.000000	7.155670	10.828697	0.000000
C	6.276147	11.681426	0.000000	6.146802	11.742001	0.000000
C	4.136127	8.876427	0.000000	4.052405	8.910348	0.000000
C	2.787116	9.121263	0.000000	2.690886	9.139757	0.000000
C	2.145296	7.858293	0.000000	2.063752	7.883040	0.000000
C	3.139247	6.912452	0.000000	3.072067	6.931879	0.000000
O	4.363721	7.526810	0.000000	4.295114	7.560513	0.000000
C	3.140330	5.486425	0.000000	3.072067	5.523875	0.000000
O	1.916691	4.871378	0.000000	1.848118	4.896284	0.000000
C	2.145296	3.520698	0.000000	2.091790	3.541750	0.000000
C	3.496489	3.278271	0.000000	3.464568	3.317725	0.000000
C	4.135700	4.541192	0.000000	4.082487	4.572130	0.000000
C	0.995705	2.678002	0.000000	0.979098	2.685975	0.000000
O	1.223907	1.327415	0.000000	1.225815	1.331212	0.000000
C	-0.000211	0.712627	0.000000	0.001305	0.701421	0.000000
C	-0.995254	1.658621	0.000000	-1.012006	1.655809	0.000000
C	-0.355577	2.920993	0.000000	-0.394189	2.908241	0.000000
C	0.000211	-0.712627	0.000000	-0.001305	-0.701421	0.000000
O	-1.223907	-1.327415	0.000000	-1.225815	-1.331212	0.000000
C	-0.995705	-2.678002	0.000000	-0.979098	-2.685975	0.000000
C	0.355577	-2.920993	0.000000	0.394189	-2.908241	0.000000
C	0.995254	-1.658621	0.000000	1.012006	-1.655809	0.000000
C	-2.145296	-3.520698	0.000000	-2.091790	-3.541750	0.000000
O	-1.916691	-4.871378	0.000000	-1.848118	-4.896284	0.000000
C	-3.140330	-5.486425	0.000000	-3.072067	-5.523875	0.000000
C	-4.135700	-4.541192	0.000000	-4.082487	-4.572130	0.000000
C	-3.496489	-3.278271	0.000000	-3.464568	-3.317725	0.000000
H	0.820351	-3.892759	0.000000	0.867685	-3.875995	0.000000
H	-2.053746	1.458761	0.000000	-2.067944	1.442048	0.000000
H	-0.820351	3.892759	0.000000	-0.867685	3.875995	0.000000
H	-3.961765	-2.306735	0.000000	-3.937986	-2.349975	0.000000
C	-3.139247	-6.912452	0.000000	-3.072067	-6.931879	0.000000
H	-5.194073	-4.741628	0.000000	-5.138626	-4.785216	0.000000
H	5.194073	4.741628	0.000000	5.138626	4.785216	0.000000
H	3.961765	2.306735	0.000000	3.937986	2.349975	0.000000
H	2.053746	-1.458761	0.000000	2.067944	-1.442048	0.000000
H	2.324015	10.093855	0.000000	2.220962	10.109344	0.000000
H	1.086589	7.659594	0.000000	1.006892	7.674368	0.000000
H	8.330413	10.960009	0.000000	8.211417	11.046739	0.000000
H	6.255471	12.757267	0.000000	6.114599	12.817682	0.000000
H	7.108039	8.512317	0.000000	7.025268	8.584606	0.000000
O	-4.363721	-7.526810	0.000000	-4.295114	-7.560513	0.000000
C	-4.136127	-8.876427	0.000000	-4.052405	-8.910348	0.000000
C	-2.787116	-9.121263	0.000000	-2.690886	-9.139757	0.000000
C	-2.145296	-7.858293	0.000000	-2.063752	-7.883040	0.000000
H	-1.086589	-7.659594	0.000000	-1.006892	-7.674368	0.000000
H	-2.324015	-10.093855	0.000000	-2.220962	-10.109344	0.000000
C	-5.289589	-9.721227	0.000000	-5.183935	-9.767806	0.000000

O	-5.057667	-11.070292	0.000000	-4.935522	-11.117223	0.000000
C	-6.276147	-11.681426	0.000000	-6.146802	-11.742001	0.000000
C	-7.271735	-10.757418	0.000000	-7.155670	-10.828697	0.000000
C	-6.637844	-9.481362	0.000000	-6.542175	-9.547264	0.000000
H	-7.108039	-8.512317	0.000000	-7.025268	-8.584606	0.000000
H	-8.330413	-10.960009	0.000000	-8.211417	-11.046739	0.000000
H	-6.255471	-12.757267	0.000000	-6.114599	-12.817682	0.000000
**9O/BP**	**Ground state**			**Excited state**		
C	0.000000	13.194603	-1.558878	-0.000000	13.192354	-1.562677
C	0.000000	12.799487	-0.247690	-0.000000	12.790675	-0.248019
O	0.000000	13.898239	0.568697	-0.000000	13.890530	0.571198
C	0.000000	14.993929	-0.242250	-0.000000	14.986664	-0.238699
C	0.000000	14.619379	-1.547896	-0.000000	14.613791	-1.546931
C	0.000000	11.524683	0.399630	-0.000000	11.520110	0.389608
O	0.000000	10.423514	-0.413186	-0.000000	10.417230	-0.424367
C	0.000000	9.322212	0.401612	-0.000000	9.313040	0.393850
C	0.000000	9.716920	1.715676	-0.000000	9.718533	1.716984
C	0.000000	11.133605	1.713731	-0.000000	11.124936	1.710733
C	0.000000	8.050553	-0.243695	-0.000000	8.049102	-0.231982
O	0.000000	6.948957	0.569941	-0.000000	6.944944	0.586371
C	0.000000	5.847358	-0.244368	-0.000000	5.838728	-0.231061
C	0.000000	6.241695	-1.559284	-0.000000	6.244771	-1.559444
C	0.000000	7.657146	-1.558802	-0.000000	7.644803	-1.557699
C	0.000000	4.576114	0.400285	-0.000000	4.577789	0.389543
O	0.000000	3.474442	-0.413607	-0.000000	3.472461	-0.430382
C	0.000000	2.372835	0.400595	-0.000000	2.365984	0.387951
C	0.000000	2.767088	1.715747	-0.000000	2.772799	1.718062
C	0.000000	4.182245	1.715565	-0.000000	4.170256	1.717951
C	0.000000	1.101647	-0.243846	-0.000000	1.106085	-0.231035
O	0.000000	0.000000	0.570200	-0.000000	0.000000	0.588508
C	0.000000	-1.101647	-0.243846	-0.000000	-1.106085	-0.231035
C	0.000000	-0.707550	-1.559104	0.000000	-0.698451	-1.560850
C	0.000000	0.707550	-1.559104	0.000000	0.698451	-1.560850
C	0.000000	-2.372835	0.400595	-0.000000	-2.365984	0.387951
O	0.000000	-3.474442	-0.413607	-0.000000	-3.472461	-0.430382
C	0.000000	-4.576114	0.400285	-0.000000	-4.577789	0.389543
C	0.000000	-4.182245	1.715565	-0.000000	-4.170256	1.717951
C	0.000000	-2.767088	1.715747	-0.000000	-2.772799	1.718062
C	0.000000	-5.847358	-0.244368	-0.000000	-5.838728	-0.231061
C	0.000000	-6.241695	-1.559284	-0.000000	-6.244771	-1.559444
C	0.000000	-7.657146	-1.558802	-0.000000	-7.644803	-1.557699
C	0.000000	-8.050553	-0.243695	-0.000000	-8.049102	-0.231982
O	0.000000	-6.948957	0.569941	-0.000000	-6.944944	0.586371
C	0.000000	-9.322212	0.401612	-0.000000	-9.313040	0.393850
O	0.000000	-10.423514	-0.413186	-0.000000	-10.417230	-0.424367
C	0.000000	-11.524683	0.399630	-0.000000	-11.520110	0.389608
C	0.000000	-11.133605	1.713731	-0.000000	-11.124936	1.710733
C	0.000000	-9.716920	1.715676	-0.000000	-9.718533	1.716984
H	0.000000	4.839117	2.569297	-0.000000	4.828376	2.570986

H	0.000000	1.364296	-2.412938	0.000000	1.356917	-2.413577
H	0.000000	-1.364296	-2.412938	-0.000000	-1.356917	-2.413577
H	0.000000	5.585258	-2.413366	-0.000000	5.587188	-2.412784
H	0.000000	8.314236	-2.412362	-0.000000	8.303456	-2.410352
H	0.000000	-4.839117	2.569297	-0.000000	-4.828376	2.570986
H	0.000000	-2.110483	2.569695	-0.000000	-2.114187	2.570652
H	0.000000	2.110483	2.569695	0.000000	2.114187	2.570652
H	0.000000	-8.314236	-2.412362	-0.000000	-8.303456	-2.410352
H	0.000000	-5.585258	-2.413366	-0.000000	-5.587188	-2.412784
H	0.000000	-11.792041	2.566290	0.000000	-11.786373	2.561233
C	0.000000	-12.799487	-0.247690	-0.000000	-12.790675	-0.248019
H	0.000000	-9.061320	2.570390	0.000000	-9.064068	2.572625
H	0.000000	9.061320	2.570390	0.000000	9.064068	2.572625
H	0.000000	11.792041	2.566290	0.000000	11.786373	2.561233
H	0.000000	12.542504	-2.416141	-0.000000	12.542378	-2.421523
H	0.000000	15.278418	-2.400839	-0.000000	15.275808	-2.397698
H	0.000000	15.944381	0.262233	-0.000000	15.937035	0.266139
O	0.000000	-13.898239	0.568697	-0.000000	-13.890530	0.571198
C	0.000000	-14.993929	-0.242250	-0.000000	-14.986664	-0.238699
C	0.000000	-14.619379	-1.547896	-0.000000	-14.613791	-1.546931
C	0.000000	-13.194603	-1.558878	-0.000000	-13.192354	-1.562677
H	0.000000	-12.542504	-2.416141	-0.000000	-12.542378	-2.421523
H	0.000000	-15.278418	-2.400839	-0.000000	-15.275808	-2.397698
H	0.000000	-15.944381	0.262233	-0.000000	-15.937035	0.266139

Part 3.

**2O/ B3LYP**	**Ground state**			**Excited state**		
C	-2.8190940	0.4341628	0.0000000	-2.8081144	0.4456540	0.0000000
C	-2.7982727	-0.9215637	0.0000000	-2.7787890	-0.9358735	0.0000000
C	-1.4230031	-1.3034771	0.0000000	-1.4399029	-1.3295354	0.0000000
C	-0.7029923	-0.1431515	0.0000000	-0.6784797	-0.1295999	0.0000000
C	0.7029923	0.1431515	0.0000000	0.6784797	0.1295999	0.0000000
C	1.4230031	1.3034771	0.0000000	1.4399029	1.3295354	0.0000000
C	2.7982727	0.9215637	0.0000000	2.7787890	0.9358735	0.0000000
C	2.8190940	-0.4341628	0.0000000	2.8081144	-0.4456540	0.0000000
O	-1.5496748	0.9282192	0.0000000	-1.5423451	0.9671495	0.0000000
O	1.5496748	-0.9282192	0.0000000	1.5423451	-0.9671495	0.0000000
H	-3.6075069	1.1649149	0.0000000	-3.6127580	1.1598791	0.0000000
H	-3.6556326	-1.5727312	0.0000000	-3.6464983	-1.5744265	0.0000000
H	-1.0164377	-2.2995183	0.0000000	-1.0309084	-2.3243734	0.0000000
H	1.0164377	2.2995183	0.0000000	1.0309084	2.3243734	0.0000000
H	3.6556326	1.5727312	0.0000000	3.6464983	1.5744265	0.0000000
H	3.6075069	-1.1649149	0.0000000	3.6127580	-1.1598791	0.0000000
**3O/ B3LYP**	**Ground state**			**Excited state**		
O	3.4775663	0.0000000	0.8384332	3.4787911	0.0000000	0.8555899
C	4.5735628	0.0000000	0.0298076	4.5608832	0.0000000	0.0248102

C	4.1993641	0.0000000	-1.2736859	4.1664147	0.0000000	-1.2857471
C	2.7727129	0.0000000	-1.2836711	2.7577923	0.0000000	-1.2936817
C	2.3797687	0.0000000	0.0253420	2.3593977	0.0000000	0.0472835
C	1.1005172	0.0000000	0.6688564	1.1082590	0.0000000	0.6613023
O	0.0000000	0.0000000	-0.1418624	0.0000000	0.0000000	-0.1702929
C	-1.1005172	0.0000000	0.6688564	-1.1082590	0.0000000	0.6613023
C	-0.7101102	0.0000000	1.9793499	-0.6925231	0.0000000	2.0067728
C	0.7101102	0.0000000	1.9793499	0.6925231	0.0000000	2.0067728
H	1.3664370	0.0000000	2.8318808	1.3541078	0.0000000	2.8554274
H	-1.3664370	0.0000000	2.8318808	-1.3541078	0.0000000	2.8554274
H	2.1209240	0.0000000	-2.1394969	2.0968036	0.0000000	-2.1426473
H	5.5255620	0.0000000	0.5290851	5.5235283	0.0000000	0.5045041
H	4.8570748	0.0000000	-2.1260224	4.8232493	0.0000000	-2.1395203
C	-2.3797687	0.0000000	0.0253420	-2.3593977	0.0000000	0.0472835
O	-3.4775663	0.0000000	0.8384332	-3.4787911	0.0000000	0.8555899
C	-4.5735628	0.0000000	0.0298076	-4.5608832	0.0000000	0.0248102
C	-4.1993641	0.0000000	-1.2736859	-4.1664147	0.0000000	-1.2857471
C	-2.7727129	0.0000000	-1.2836711	-2.7577923	0.0000000	-1.2936817
H	-2.1209240	0.0000000	-2.1394969	-2.0968036	0.0000000	-2.1426473
H	-5.5255620	0.0000000	0.5290851	-5.5235283	0.0000000	0.5045041
H	-4.8570748	0.0000000	-2.1260224	-4.8232493	0.0000000	-2.1395203
**4O/ B3LYP**	**Ground state**			**Excited state**		
O	-1.5236148	0.9711279	0.0000000	-1.5126462	0.9996816	0.0000000
C	-2.8112942	0.5128477	0.0000000	-2.8078149	0.5285364	0.0000000
C	-2.8161982	-0.8550739	0.0000000	-2.8006710	-0.8667632	0.0000000
C	-1.4576380	-1.2657473	0.0000000	-1.4706860	-1.2731669	0.0000000
C	-0.7036363	-0.1233262	0.0000000	-0.6869517	-0.1083646	0.0000000
C	0.7036363	0.1233262	0.0000000	0.6869517	0.1083646	0.0000000
O	1.5236148	-0.9711279	0.0000000	1.5126462	-0.9996816	0.0000000
C	2.8112942	-0.5128477	0.0000000	2.8078149	-0.5285364	0.0000000
C	2.8161982	0.8550739	0.0000000	2.8006710	0.8667632	0.0000000
C	1.4576380	1.2657473	0.0000000	1.4706860	1.2731669	0.0000000
H	1.0765223	2.2718360	0.0000000	1.0860779	2.2781268	0.0000000
H	3.6911369	1.4812311	0.0000000	3.6790594	1.4886421	0.0000000
H	-1.0765223	-2.2718360	0.0000000	-1.0860779	-2.2781268	0.0000000
H	-3.6911369	-1.4812311	0.0000000	-3.6790594	-1.4886421	0.0000000
C	3.8501140	-1.4977865	0.0000000	3.8383841	-1.4823601	0.0000000
O	5.1359941	-1.0361984	0.0000000	5.1345333	-1.0219489	0.0000000
C	5.9517306	-2.1268172	0.0000000	5.9439690	-2.1179023	0.0000000
C	5.2171317	-3.2668616	0.0000000	5.2021786	-3.2625796	0.0000000
C	3.8484718	-2.8646799	0.0000000	3.8432401	-2.8698182	0.0000000
H	2.9774001	-3.4959332	0.0000000	2.9718422	-3.5008152	0.0000000
H	7.0073628	-1.9237593	0.0000000	7.0014700	-1.9226646	0.0000000
H	5.6008424	-4.2727455	0.0000000	5.5904265	-4.2671992	0.0000000
C	-3.8501140	1.4977865	0.0000000	-3.8383841	1.4823601	0.0000000
O	-5.1359941	1.0361984	0.0000000	-5.1345333	1.0219489	0.0000000
C	-5.9517306	2.1268172	0.0000000	-5.9439690	2.1179023	0.0000000
C	-5.2171317	3.2668616	0.0000000	-5.2021786	3.2625796	0.0000000

C	-3.8484718	2.8646799	0.0000000	-3.8432401	2.8698182	0.0000000
H	-7.0073628	1.9237593	0.0000000	-7.0014700	1.9226646	0.0000000
H	-5.6008424	4.2727455	0.0000000	-5.5904265	4.2671992	0.0000000
H	-2.9774001	3.4959332	0.0000000	-2.9718422	3.5008152	0.0000000
**5O/ B3LYP**	**Ground state**			**Excited state**		
O	6.9559344	0.0000000	0.7035637	6.9465007	0.0000000	0.7186730
C	8.0504797	0.0000000	-0.1068254	8.0420281	0.0000000	-0.0902842
C	7.6742720	0.0000000	-1.4098516	7.6689115	0.0000000	-1.3997698
C	6.2478414	0.0000000	-1.4177071	6.2511260	0.0000000	-1.4193380
C	5.8568253	0.0000000	-0.1078932	5.8441214	0.0000000	-0.0994274
C	4.5788012	0.0000000	0.5368190	4.5761588	0.0000000	0.5197813
O	3.4774361	0.0000000	-0.2724227	3.4739420	0.0000000	-0.3003125
C	2.3771881	0.0000000	0.5400038	2.3641547	0.0000000	0.5169278
C	2.7709891	0.0000000	1.8511819	2.7746010	0.0000000	1.8521627
C	4.1900329	0.0000000	1.8484861	4.1706354	0.0000000	1.8471762
C	1.1010492	0.0000000	-0.1011576	1.1075615	0.0000000	-0.0902460
O	0.0000000	0.0000000	0.7098091	0.0000000	0.0000000	0.7320178
C	-1.1010492	0.0000000	-0.1011576	-1.1075615	0.0000000	-0.0902460
C	-0.7090883	0.0000000	-1.4132524	-0.6950020	0.0000000	-1.4270688
C	0.7090883	0.0000000	-1.4132524	0.6950020	0.0000000	-1.4270688
C	-2.3771881	0.0000000	0.5400038	-2.3641547	0.0000000	0.5169278
O	-3.4774361	0.0000000	-0.2724227	-3.4739420	0.0000000	-0.3003125
C	-4.5788012	0.0000000	0.5368190	-4.5761588	0.0000000	0.5197813
C	-4.1900329	0.0000000	1.8484861	-4.1706354	0.0000000	1.8471762
C	-2.7709891	0.0000000	1.8511819	-2.7746010	0.0000000	1.8521627
C	-5.8568253	0.0000000	-0.1078932	-5.8441214	0.0000000	-0.0994274
O	-6.9559344	0.0000000	0.7035637	-6.9465007	0.0000000	0.7186730
C	-8.0504797	0.0000000	-0.1068254	-8.0420281	0.0000000	-0.0902842
C	-7.6742720	0.0000000	-1.4098516	-7.6689115	0.0000000	-1.3997698
C	-6.2478414	0.0000000	-1.4177071	-6.2511260	0.0000000	-1.4193380
H	9.0033318	0.0000000	0.3908211	8.9950589	0.0000000	0.4076213
H	8.3307028	0.0000000	-2.2631568	8.3325239	0.0000000	-2.2479160
H	5.5947629	0.0000000	-2.2725444	5.6027659	0.0000000	-2.2778131
H	4.8479222	0.0000000	2.6998226	4.8308821	0.0000000	2.6971459
H	2.1167456	0.0000000	2.7052446	2.1176686	0.0000000	2.7043960
H	1.3650427	0.0000000	-2.2660360	1.3540588	0.0000000	-2.2777172
H	-1.3650427	0.0000000	-2.2660360	-1.3540588	0.0000000	-2.2777172
H	-2.1167456	0.0000000	2.7052446	-2.1176686	0.0000000	2.7043960
H	-4.8479222	0.0000000	2.6998226	-4.8308821	0.0000000	2.6971459
H	-5.5947629	0.0000000	-2.2725444	-5.6027659	0.0000000	-2.2778131
H	-9.0033318	0.0000000	0.3908211	-8.9950589	0.0000000	0.4076213
H	-8.3307028	0.0000000	-2.2631568	-8.3325239	0.0000000	-2.2479160
**6O/ B3LYP**	**Ground state**			**Excited state**		
O	1.8067172	0.0016196	0.0000000	1.8116201	0.0257952	0.0000000
C	0.5271621	-0.4812338	0.0000000	0.5230121	-0.4621603	0.0000000
C	0.5497113	-1.8506904	0.0000000	0.5572329	-1.8588275	0.0000000

C	1.9148421	-2.2342040	0.0000000	1.8979479	-2.2346249	0.0000000
C	2.6472430	-1.0769420	0.0000000	2.6538590	-1.0633485	0.0000000
C	4.0490464	-0.8044948	0.0000000	4.0324587	-0.8168063	0.0000000
O	4.8893948	-1.8835869	0.0000000	4.8778738	-1.9022694	0.0000000
C	6.1681491	-1.4013038	0.0000000	6.1598686	-1.4153759	0.0000000
C	6.1475571	-0.0333321	0.0000000	6.1325677	-0.0324252	0.0000000
C	4.7819053	0.3519394	0.0000000	4.7862116	0.3526153	0.0000000
H	7.0107211	0.6089465	0.0000000	6.9985242	0.6066599	0.0000000
H	2.3157233	-3.2326043	0.0000000	2.3014471	-3.2322453	0.0000000
H	-0.3124449	-2.4942652	0.0000000	-0.3069000	-2.5000644	0.0000000
C	7.2252908	-2.3663672	0.0000000	7.2140947	-2.3604990	0.0000000
O	8.5022336	-1.8806123	0.0000000	8.4949258	-1.8712704	0.0000000
C	9.3383015	-2.9556243	0.0000000	9.3316399	-2.9454535	0.0000000
C	8.6253377	-4.1093692	0.0000000	8.6193319	-4.1041542	0.0000000
C	7.2494151	-3.7330916	0.0000000	7.2473754	-3.7374363	0.0000000
H	6.3903972	-4.3806500	0.0000000	6.3909116	-4.3883917	0.0000000
H	10.3899335	-2.7327664	0.0000000	10.3835013	-2.7227003	0.0000000
H	9.0279758	-5.1078174	0.0000000	9.0277193	-5.1005245	0.0000000
C	-0.5271621	0.4812338	0.0000000	-0.5230121	0.4621603	0.0000000
O	-1.8067172	-0.0016196	0.0000000	-1.8116201	-0.0257952	0.0000000
C	-2.6472430	1.0769420	0.0000000	-2.6538590	1.0633485	0.0000000
C	-1.9148421	2.2342040	0.0000000	-1.8979479	2.2346249	0.0000000
C	-0.5497113	1.8506904	0.0000000	-0.5572329	1.8588275	0.0000000
H	-2.3157233	3.2326043	0.0000000	-2.3014471	3.2322453	0.0000000
H	0.3124449	2.4942652	0.0000000	0.3069000	2.5000644	0.0000000
H	4.3821459	1.3507685	0.0000000	4.3866636	1.3516819	0.0000000
C	-4.0490464	0.8044948	0.0000000	-4.0324587	0.8168063	0.0000000
C	-4.7819053	-0.3519394	0.0000000	-4.7862116	-0.3526153	0.0000000
C	-6.1475571	0.0333321	0.0000000	-6.1325677	0.0324252	0.0000000
C	-6.1681491	1.4013038	0.0000000	-6.1598686	1.4153759	0.0000000
O	-4.8893948	1.8835869	0.0000000	-4.8778738	1.9022694	0.0000000
H	-7.0107211	-0.6089465	0.0000000	-6.9985242	-0.6066599	0.0000000
H	-4.3821459	-1.3507685	0.0000000	-4.3866636	-1.3516819	0.0000000
C	-7.2252908	2.3663672	0.0000000	-7.2140947	2.3604990	0.0000000
C	-7.2494151	3.7330916	0.0000000	-7.2473754	3.7374363	0.0000000
C	-8.6253377	4.1093692	0.0000000	-8.6193319	4.1041542	0.0000000
C	-9.3383015	2.9556243	0.0000000	-9.3316399	2.9454535	0.0000000
O	-8.5022336	1.8806123	0.0000000	-8.4949258	1.8712704	0.0000000
H	-9.0279758	5.1078174	0.0000000	-9.0277193	5.1005245	0.0000000
H	-10.3899335	2.7327664	0.0000000	-10.383501	2.7227003	0.0000000
H	-6.3903972	4.3806500	0.0000000	-6.3909116	4.3883917	0.0000000
**7O/ B3LYP**	**Ground state**			**Excited state**		
C	-9.7272152	0.0000000	-1.4887993	-9.7242204	0.0000000	-1.4936583
C	-9.3341612	0.0000000	-0.1795247	-9.3220984	0.0000000	-0.1790129
O	-10.4320487	0.0000000	0.6336557	-10.422019	0.0000000	0.6376150
C	-11.5278003	0.0000000	-0.1750216	-11.518155	0.0000000	-0.1699831
C	-11.1536268	0.0000000	-1.4786918	-11.145783	0.0000000	-1.4771687
C	-8.0554316	0.0000000	0.4636535	-8.0498432	0.0000000	0.4501606

O	-6.9548985	0.0000000	-0.3465832	-6.9473329	0.0000000	-0.3626887
C	-5.8539816	0.0000000	0.4649276	-5.8415501	0.0000000	0.4530980
C	-6.2466181	0.0000000	1.7766021	-6.2492095	0.0000000	1.7788173
C	-7.6655632	0.0000000	1.7751289	-7.6529940	0.0000000	1.7717708
C	-4.5784733	0.0000000	-0.1769608	-4.5783752	0.0000000	-0.1614684
O	-3.4773774	0.0000000	0.6337311	-3.4737661	0.0000000	0.6567677
C	-2.3763245	0.0000000	-0.1775127	-2.3657733	0.0000000	-0.1595849
C	-2.7688496	0.0000000	-1.4898335	-2.7752448	0.0000000	-1.4921941
C	-4.1867309	0.0000000	-1.4894195	-4.1704530	0.0000000	-1.4902054
C	-1.1011023	0.0000000	0.4637143	-1.1069898	0.0000000	0.4488417
O	0.0000000	0.0000000	-0.3473177	0.0000000	0.0000000	-0.3698296
C	1.1011023	0.0000000	0.4637143	1.1069898	0.0000000	0.4488417
C	0.7088438	0.0000000	1.7762324	0.6962327	0.0000000	1.7819429
C	-0.7088438	0.0000000	1.7762324	-0.6962327	0.0000000	1.7819429
C	2.3763245	0.0000000	-0.1775127	2.3657733	0.0000000	-0.1595849
O	3.4773774	0.0000000	0.6337311	3.4737661	0.0000000	0.6567677
C	4.5784733	0.0000000	-0.1769608	4.5783752	0.0000000	-0.1614684
C	4.1867309	0.0000000	-1.4894195	4.1704530	0.0000000	-1.4902054
C	2.7688496	0.0000000	-1.4898335	2.7752448	0.0000000	-1.4921941
C	5.8539816	0.0000000	0.4649276	5.8415501	0.0000000	0.4530980
O	6.9548985	0.0000000	-0.3465832	6.9473329	0.0000000	-0.3626887
C	8.0554316	0.0000000	0.4636535	8.0498432	0.0000000	0.4501606
C	7.6655632	0.0000000	1.7751289	7.6529940	0.0000000	1.7717708
C	6.2466181	0.0000000	1.7766021	6.2492095	0.0000000	1.7788173
C	9.3341612	0.0000000	-0.1795247	9.3220984	0.0000000	-0.1790129
C	9.7272152	0.0000000	-1.4887993	9.7242204	0.0000000	-1.4936583
C	11.1536268	0.0000000	-1.4786918	11.1457831	0.0000000	-1.4771687
C	11.5278003	0.0000000	-0.1750216	11.5181551	0.0000000	-0.1699831
O	10.4320487	0.0000000	0.6336557	10.4220192	0.0000000	0.6376150
H	-1.3644407	0.0000000	2.6292835	-1.3540347	0.0000000	2.6335766
H	2.1135020	0.0000000	-2.3430814	2.1181532	0.0000000	-2.3443148
H	4.8426283	0.0000000	-2.3422323	4.8284386	0.0000000	-2.3417708
H	-2.1135020	0.0000000	-2.3430814	-2.1181532	0.0000000	-2.3443148
H	-4.8426283	0.0000000	-2.3422323	-4.8284386	0.0000000	-2.3417708
H	8.3226746	0.0000000	2.6270695	8.3138044	0.0000000	2.6212268
H	5.5918519	0.0000000	2.6302891	5.5956574	0.0000000	2.6335597
H	1.3644407	0.0000000	2.6292835	1.3540347	0.0000000	2.6335766
H	11.8113759	0.0000000	-2.3309925	11.8074364	0.0000000	-2.3266697
H	9.0756938	0.0000000	-2.3448504	9.0754983	0.0000000	-2.3518313
H	-5.5918519	0.0000000	2.6302891	-5.5956574	0.0000000	2.6335597
H	-8.3226746	0.0000000	2.6270695	-8.3138044	0.0000000	2.6212268
H	-9.0756938	0.0000000	-2.3448504	-9.0754983	0.0000000	-2.3518313
H	-11.8113759	0.0000000	-2.3309925	-11.807436	0.0000000	-2.3266697
H	-12.4798565	0.0000000	0.3241678	-12.470290	0.0000000	0.3293818
H	12.4798565	0.0000000	0.3241678	12.4702902	0.0000000	0.3293818
**8O/ B3LYP**	**Ground state**			**Excited state**		
O	-11.9080393	2.5620270	0.0000000	-11.898616	2.5562592	0.0000000
C	-10.6419639	3.0755120	0.0000000	-10.630510	3.0722554	0.0000000

C	-10.6958567	4.4414491	0.0000000	-10.690566	4.4438303	0.0000000
C	-12.0796568	4.7876146	0.0000000	-12.072077	4.7839349	0.0000000
C	-12.7673135	3.6185556	0.0000000	-12.758667	3.6117098	0.0000000
C	-9.5641963	2.1335764	0.0000000	-9.5548219	2.1422481	0.0000000
C	-9.5139647	0.7662900	0.0000000	-9.5017287	0.7658870	0.0000000
C	-8.1402932	0.4107554	0.0000000	-8.1412258	0.4085151	0.0000000
C	-7.4327366	1.5829408	0.0000000	-7.4185477	1.5882435	0.0000000
O	-8.2962977	2.6435211	0.0000000	-8.2849984	2.6531541	0.0000000
C	-6.0374104	1.8862303	0.0000000	-6.0388455	1.8719169	0.0000000
O	-5.1734326	0.8264610	0.0000000	-5.1731079	0.8063129	0.0000000
C	-3.9047849	1.3373492	0.0000000	-3.8976973	1.3198164	0.0000000
C	-3.9574771	2.7061092	0.0000000	-3.9614357	2.7092631	0.0000000
C	-5.3305933	3.0594653	0.0000000	-5.3160834	3.0551142	0.0000000
C	-2.8299000	0.3982548	0.0000000	-2.8291241	0.4137489	0.0000000
O	-1.5612832	0.9088368	0.0000000	-1.5540473	0.9298704	0.0000000
C	-0.6973858	-0.1513381	0.0000000	-0.6855796	-0.1375161	0.0000000
C	-1.4048442	-1.3244644	0.0000000	-1.4149407	-1.3254078	0.0000000
C	-2.7776069	-0.9706826	0.0000000	-2.7645209	-0.9769786	0.0000000
C	0.6973858	0.1513381	0.0000000	0.6855796	0.1375161	0.0000000
O	1.5612832	-0.9088368	0.0000000	1.5540473	-0.9298704	0.0000000
C	2.8299000	-0.3982548	0.0000000	2.8291241	-0.4137489	0.0000000
C	2.7776069	0.9706826	0.0000000	2.7645209	0.9769786	0.0000000
C	1.4048442	1.3244644	0.0000000	1.4149407	1.3254078	0.0000000
C	3.9047849	-1.3373492	0.0000000	3.8976973	-1.3198164	0.0000000
O	5.1734326	-0.8264610	0.0000000	5.1731079	-0.8063129	0.0000000
C	6.0374104	-1.8862303	0.0000000	6.0388455	-1.8719169	0.0000000
C	5.3305933	-3.0594653	0.0000000	5.3160834	-3.0551142	0.0000000
C	3.9574771	-2.7061092	0.0000000	3.9614357	-2.7092631	0.0000000
H	3.6253557	1.6331176	0.0000000	3.6138222	1.6378312	0.0000000
H	-0.9829075	-2.3141495	0.0000000	-0.9909289	-2.3144186	0.0000000
H	-3.6253557	-1.6331176	0.0000000	-3.6138222	-1.6378312	0.0000000
H	3.1099806	-3.3688807	0.0000000	3.1134056	-3.3715770	0.0000000
C	7.4327366	-1.5829408	0.0000000	7.4185477	-1.5882435	0.0000000
H	5.7529667	-4.0489539	0.0000000	5.7410771	-4.0437919	0.0000000
H	-5.7529667	4.0489539	0.0000000	-5.7410771	4.0437919	0.0000000
H	-3.1099806	3.3688807	0.0000000	-3.1134056	3.3715770	0.0000000
H	0.9829075	2.3141495	0.0000000	0.9909289	2.3144186	0.0000000
H	-10.3628957	0.1052970	0.0000000	-10.353289	0.1078713	0.0000000
H	-7.7193014	-0.5793295	0.0000000	-7.7219266	-0.5823603	0.0000000
H	-12.5039084	5.7770885	0.0000000	-12.500061	5.7719446	0.0000000
H	-13.8138214	3.3727447	0.0000000	-13.805186	3.3654397	0.0000000
H	-9.8513291	5.1078410	0.0000000	-9.8479439	5.1126170	0.0000000
O	8.2962977	-2.6435211	0.0000000	8.2849984	-2.6531541	0.0000000
C	9.5641963	-2.1335764	0.0000000	9.5548219	-2.1422481	0.0000000
C	9.5139647	-0.7662900	0.0000000	9.5017287	-0.7658870	0.0000000
C	8.1402932	-0.4107554	0.0000000	8.1412258	-0.4085151	0.0000000
H	7.7193014	0.5793295	0.0000000	7.7219266	0.5823603	0.0000000
H	10.3628957	-0.1052970	0.0000000	10.3532891	-0.1078713	0.0000000
C	10.6419639	-3.0755120	0.0000000	10.6305106	-3.0722554	0.0000000
O	11.9080393	-2.5620270	0.0000000	11.8986164	-2.5562592	0.0000000

C	12.7673135	-3.6185556	0.0000000	12.7586672	-3.6117098	0.0000000
C	12.0796568	-4.7876146	0.0000000	12.0720774	-4.7839349	0.0000000
C	10.6958567	-4.4414491	0.0000000	10.6905663	-4.4438303	0.0000000
H	9.8513291	-5.1078410	0.0000000	9.8479439	-5.1126170	0.0000000
H	12.5039084	-5.7770885	0.0000000	12.5000618	-5.7719446	0.0000000
H	13.8138214	-3.3727447	0.0000000	13.8051864	-3.3654397	0.0000000
**9O/ B3LYP**	**Ground state**			**Excited state**		
C	-13.2044812	0.0000000	-1.5252232	-13.198212	0.0000000	-1.5288123
C	-12.8113756	0.0000000	-0.2159653	-12.799422	0.0000000	-0.2165507
O	-13.9092169	0.0000000	0.5972666	-13.898323	0.0000000	0.5989124
C	-15.0049909	0.0000000	-0.2113594	-14.994405	0.0000000	-0.2087278
C	-14.6308813	0.0000000	-1.5150530	-14.621718	0.0000000	-1.5145848
C	-11.5326271	0.0000000	0.4271662	-11.524674	0.0000000	0.4181110
O	-10.4321211	0.0000000	-0.3830645	-10.422709	0.0000000	-0.3926209
C	-9.3312050	0.0000000	0.4284529	-9.3188522	0.0000000	0.4219438
C	-9.7238384	0.0000000	1.7401266	-9.7224203	0.0000000	1.7423190
C	-11.1427649	0.0000000	1.7386608	-11.131392	0.0000000	1.7361474
C	-8.0557183	0.0000000	-0.2134502	-8.0517211	0.0000000	-0.2007502
O	-6.9546086	0.0000000	0.5971573	-6.9479619	0.0000000	0.6135609
C	-5.8536049	0.0000000	-0.2141828	-5.8418909	0.0000000	-0.2011882
C	-6.2462298	0.0000000	-1.5264803	-6.2487411	0.0000000	-1.5280914
C	-7.6640731	0.0000000	-1.5259731	-7.6496018	0.0000000	-1.5246098
C	-4.5783869	0.0000000	0.4269595	-4.5791993	0.0000000	0.4127956
O	-3.4773102	0.0000000	-0.3839979	-3.4738399	0.0000000	-0.4038607
C	-2.3762124	0.0000000	0.4271246	-2.3668796	0.0000000	0.4120311
C	-2.7686086	0.0000000	1.7396658	-2.7759153	0.0000000	1.7426938
C	-4.1861977	0.0000000	1.7395800	-4.1716755	0.0000000	1.7411751
C	-1.1010943	0.0000000	-0.2139509	-1.1066009	0.0000000	-0.1975658
O	0.0000000	0.0000000	0.5970791	0.0000000	0.0000000	0.6194097
C	1.1010943	0.0000000	-0.2139509	1.1066009	0.0000000	-0.1975658
C	0.7087735	0.0000000	-1.5265664	0.6970098	0.0000000	-1.5285707
C	-0.7087735	0.0000000	-1.5265664	-0.6970098	0.0000000	-1.5285707
C	2.3762124	0.0000000	0.4271246	2.3668796	0.0000000	0.4120311
O	3.4773102	0.0000000	-0.3839979	3.4738399	0.0000000	-0.4038607
C	4.5783869	0.0000000	0.4269595	4.5791993	0.0000000	0.4127956
C	4.1861977	0.0000000	1.7395800	4.1716755	0.0000000	1.7411751
C	2.7686086	0.0000000	1.7396658	2.7759153	0.0000000	1.7426938
C	5.8536049	0.0000000	-0.2141828	5.8418909	0.0000000	-0.2011882
C	6.2462298	0.0000000	-1.5264803	6.2487411	0.0000000	-1.5280914
C	7.6640731	0.0000000	-1.5259731	7.6496018	0.0000000	-1.5246098
C	8.0557183	0.0000000	-0.2134502	8.0517211	0.0000000	-0.2007502
O	6.9546086	0.0000000	0.5971573	6.9479619	0.0000000	0.6135609
C	9.3312050	0.0000000	0.4284529	9.3188522	0.0000000	0.4219438
O	10.4321211	0.0000000	-0.3830645	10.4227090	0.0000000	-0.3926209
C	11.5326271	0.0000000	0.4271662	11.5246747	0.0000000	0.4181110
C	11.1427649	0.0000000	1.7386608	11.1313924	0.0000000	1.7361474
C	9.7238384	0.0000000	1.7401266	9.7224203	0.0000000	1.7423190
H	-4.8418595	0.0000000	2.5925752	-4.8292720	0.0000000	2.5930161

H	-1.3643620	0.0000000	-2.3796229	-1.3543181	0.0000000	-2.3805834
H	1.3643620	0.0000000	-2.3796229	1.3543181	0.0000000	-2.3805834
H	-5.5909608	0.0000000	-2.3797921	-5.5934400	0.0000000	-2.3815145
H	-8.3200310	0.0000000	-2.3787352	-8.3078341	0.0000000	-2.3759556
H	4.8418595	0.0000000	2.5925752	4.8292720	0.0000000	2.5930161
H	2.1130988	0.0000000	2.5927882	2.1192292	0.0000000	2.5951226
H	-2.1130988	0.0000000	2.5927882	-2.1192292	0.0000000	2.5951226
H	8.3200310	0.0000000	-2.3787352	8.3078341	0.0000000	-2.3759556
H	5.5909608	0.0000000	-2.3797921	5.5934400	0.0000000	-2.3815145
H	11.7998782	0.0000000	2.5905971	11.7915062	0.0000000	2.5860234
C	12.8113756	0.0000000	-0.2159653	12.7994221	0.0000000	-0.2165507
H	9.0690812	0.0000000	2.5938221	9.0692829	0.0000000	2.5973099
H	-9.0690812	0.0000000	2.5938221	-9.0692829	0.0000000	2.5973099
H	-11.7998782	0.0000000	2.5905971	-11.791506	0.0000000	2.5860234
H	-12.5530072	0.0000000	-2.3813112	-12.548646	0.0000000	-2.3863355
H	-15.2886668	0.0000000	-2.3673236	-15.281994	0.0000000	-2.3650521
H	-15.9570270	0.0000000	0.2878688	-15.946383	0.0000000	0.2908068
O	13.9092169	0.0000000	0.5972666	13.8983238	0.0000000	0.5989124
C	15.0049909	0.0000000	-0.2113594	14.9944052	0.0000000	-0.2087278
C	14.6308813	0.0000000	-1.5150530	14.6217188	0.0000000	-1.5145848
C	13.2044812	0.0000000	-1.5252232	13.1982129	0.0000000	-1.5288123
H	12.5530072	0.0000000	-2.3813112	12.5486461	0.0000000	-2.3863355
H	15.2886668	0.0000000	-2.3673236	15.2819944	0.0000000	-2.3650521
H	15.9570270	0.0000000	0.2878688	15.9463830	0.0000000	0.2908068

Part 4.

**2O/ PBE0**	**Ground state**			**Excited state**		
C	-2.8019400	0.4335265	0.0000000	-2.7896848	0.4449416	0.0000000
C	-2.7873208	-0.9201693	0.0000000	-2.7670467	-0.9340221	0.0000000
C	-1.4168361	-1.3022381	0.0000000	-1.4324512	-1.3281350	0.0000000
C	-0.7020601	-0.1421696	0.0000000	-0.6772813	-0.1278106	0.0000000
C	0.7020601	0.1421696	0.0000000	0.6772813	0.1278106	0.0000000
C	1.4168361	1.3022381	0.0000000	1.4324512	1.3281350	0.0000000
C	2.7873208	0.9201693	0.0000000	2.7670467	0.9340221	0.0000000
C	2.8019400	-0.4335265	0.0000000	2.7896848	-0.4449416	0.0000000
O	-1.5409886	0.9215955	0.0000000	-1.5326338	0.9606364	0.0000000
O	1.5409886	-0.9215955	0.0000000	1.5326338	-0.9606364	0.0000000
H	-3.5905478	1.1662342	0.0000000	-3.5949874	1.1608028	0.0000000
H	-3.6481223	-1.5684999	0.0000000	-3.6383551	-1.5695130	0.0000000
H	-1.0068822	-2.2981827	0.0000000	-1.0203277	-2.3230511	0.0000000
H	1.0068822	2.2981827	0.0000000	1.0203277	2.3230511	0.0000000
H	3.6481223	1.5684999	0.0000000	3.6383551	1.5695130	0.0000000
H	3.5905478	-1.1662342	0.0000000	3.5949874	-1.1608028	0.0000000
**3O/ PBE0**	**Ground state**			**Excited state**		
O	3.4583749	0.0000000	0.8315297	3.4593820	0.0000000	0.8487667
C	4.5464821	0.0000000	0.0291529	4.5327317	0.0000000	0.0233276

C	4.1767175	0.0000000	-1.2734029	4.1420904	0.0000000	-1.2857617
C	2.7545994	0.0000000	-1.2824632	2.7374186	0.0000000	-1.2916707
C	2.3688621	0.0000000	0.0254887	2.3479072	0.0000000	0.0485762
C	1.0917222	0.0000000	0.6685318	1.0990937	0.0000000	0.6606933
O	0.0000000	0.0000000	-0.1357129	0.0000000	0.0000000	-0.1645660
C	-1.0917222	0.0000000	0.6685318	-1.0990937	0.0000000	0.6606933
C	-0.7081531	0.0000000	1.9778930	-0.6902602	0.0000000	2.0055916
C	0.7081531	0.0000000	1.9778930	0.6902602	0.0000000	2.0055916
C	-2.3688621	0.0000000	0.0254887	-2.3479072	0.0000000	0.0485762
O	-3.4583749	0.0000000	0.8315297	-3.4593820	0.0000000	0.8487667
C	-4.5464821	0.0000000	0.0291529	-4.5327317	0.0000000	0.0233276
C	-4.1767175	0.0000000	-1.2734029	-4.1420904	0.0000000	-1.2857617
C	-2.7545994	0.0000000	-1.2824632	-2.7374186	0.0000000	-1.2916707
H	5.4999988	0.0000000	0.5287181	5.4974306	0.0000000	0.5023639
H	4.8371374	0.0000000	-2.1249508	4.8010727	0.0000000	-2.1392026
H	2.0982346	0.0000000	-2.1363103	2.0711290	0.0000000	-2.1381007
H	-2.0982346	0.0000000	-2.1363103	-2.0711290	0.0000000	-2.1381007
H	-5.4999988	0.0000000	0.5287181	-5.4974306	0.0000000	0.5023639
H	-4.8371374	0.0000000	-2.1249508	-4.8010727	0.0000000	-2.1392026
H	1.3686750	0.0000000	2.8286717	1.3558620	0.0000000	2.8527016
H	-1.3686750	0.0000000	2.8286717	-1.3558620	0.0000000	2.8527016
**4O/ PBE0**	**Ground state**			**Excited state**		
O	-1.7622633	0.3445737	0.0000000	-1.7624997	0.3765569	0.0000000
C	-2.7865876	-0.5437700	0.0000000	-2.7886039	-0.5267268	0.0000000
C	-2.3002088	-1.8190298	0.0000000	-2.2810910	-1.8233623	0.0000000
C	-0.8892151	-1.7073449	0.0000000	-0.8987537	-1.7185898	0.0000000
C	-0.6095548	-0.3706287	0.0000000	-0.5993055	-0.3492399	0.0000000
C	0.6095548	0.3706287	0.0000000	0.5993055	0.3492399	0.0000000
O	1.7622633	-0.3445737	0.0000000	1.7624997	-0.3765569	0.0000000
C	2.7865876	0.5437700	0.0000000	2.7886039	0.5267268	0.0000000
C	2.3002088	1.8190298	0.0000000	2.2810910	1.8233623	0.0000000
C	0.8892151	1.7073449	0.0000000	0.8987537	1.7185898	0.0000000
C	4.1106133	0.0048969	0.0000000	4.0938335	0.0152246	0.0000000
O	5.1324032	0.8953566	0.0000000	5.1253866	0.9081513	0.0000000
C	6.2809267	0.1822915	0.0000000	6.2699099	0.1878785	0.0000000
C	6.0162735	-1.1456929	0.0000000	6.0006633	-1.1467823	0.0000000
C	4.5995683	-1.2682416	0.0000000	4.5958602	-1.2745290	0.0000000
H	-2.8914673	-2.7193248	0.0000000	-2.8772166	-2.7209188	0.0000000
H	-0.1641650	-2.5037929	0.0000000	-0.1710844	-2.5129758	0.0000000
H	4.0135256	-2.1718045	0.0000000	4.0094160	-2.1780096	0.0000000
H	7.1916005	0.7562634	0.0000000	7.1851746	0.7553153	0.0000000
H	6.7425350	-1.9418185	0.0000000	6.7307437	-1.9400145	0.0000000
H	0.1641650	2.5037929	0.0000000	0.1710844	2.5129758	0.0000000
H	2.8914673	2.7193248	0.0000000	2.8772166	2.7209188	0.0000000
C	-4.1106133	-0.0048969	0.0000000	-4.0938335	-0.0152246	0.0000000
O	-5.1324032	-0.8953566	0.0000000	-5.1253866	-0.9081513	0.0000000
C	-6.2809267	-0.1822915	0.0000000	-6.2699099	-0.1878785	0.0000000
C	-6.0162735	1.1456929	0.0000000	-6.0006633	1.1467823	0.0000000

C	-4.5995683	1.2682416	0.0000000	-4.5958602	1.2745290	0.0000000
H	-4.0135256	2.1718045	0.0000000	-4.0094160	2.1780096	0.0000000
H	-7.1916005	-0.7562634	0.0000000	-7.1851746	-0.7553153	0.0000000
H	-6.7425350	1.9418185	0.0000000	-6.7307437	1.9400145	0.0000000
**5O/ PBE0**	**Ground state**			**Excited state**		
O	3.4583005	0.0000000	0.2652565	3.4543768	0.0000000	0.2929201
C	4.5501069	0.0000000	-0.5385886	4.5471257	0.0000000	-0.5211871
C	4.1669640	0.0000000	-1.8487095	4.1483471	0.0000000	-1.8472249
C	2.7517755	0.0000000	-1.8500085	2.7562470	0.0000000	-1.8521886
C	2.3662147	0.0000000	-0.5396974	2.3529960	0.0000000	-0.5174478
C	1.0922158	0.0000000	0.1015676	1.0987271	0.0000000	0.0868404
O	0.0000000	0.0000000	-0.7028729	0.0000000	0.0000000	-0.7292294
C	-1.0922158	0.0000000	0.1015676	-1.0987271	0.0000000	0.0868404
C	-0.7072225	0.0000000	1.4123810	-0.6926181	0.0000000	1.4233685
C	0.7072225	0.0000000	1.4123810	0.6926181	0.0000000	1.4233685
C	-2.3662147	0.0000000	-0.5396974	-2.3529960	0.0000000	-0.5174478
O	-3.4583005	0.0000000	0.2652565	-3.4543768	0.0000000	0.2929201
C	-4.5501069	0.0000000	-0.5385886	-4.5471257	0.0000000	-0.5211871
C	-4.1669640	0.0000000	-1.8487095	-4.1483471	0.0000000	-1.8472249
C	-2.7517755	0.0000000	-1.8500085	-2.7562470	0.0000000	-1.8521886
C	5.8269940	0.0000000	0.1038728	5.8139210	0.0000000	0.0960875
O	6.9163603	0.0000000	-0.7025186	6.9069941	0.0000000	-0.7158090
C	8.0044856	0.0000000	0.0996127	7.9953687	0.0000000	0.0857856
C	7.6350868	0.0000000	1.4023811	7.6282736	0.0000000	1.3944502
C	6.2131717	0.0000000	1.4118920	6.2144567	0.0000000	1.4142219
C	-5.8269940	0.0000000	0.1038728	-5.8139210	0.0000000	0.0960875
O	-6.9163603	0.0000000	-0.7025186	-6.9069941	0.0000000	-0.7158090
H	-4.8281761	0.0000000	-2.6989487	-4.8126001	0.0000000	-2.6956153
H	-2.0926711	0.0000000	-2.7018386	-2.0952148	0.0000000	-2.7028007
H	-1.3670992	0.0000000	2.2636133	-1.3555884	0.0000000	2.2725483
H	1.3670992	0.0000000	2.2636133	1.3555884	0.0000000	2.2725483
H	2.0926711	0.0000000	-2.7018386	2.0952148	0.0000000	-2.7028007
H	4.8281761	0.0000000	-2.6989487	4.8126001	0.0000000	-2.6956153
H	5.5571609	0.0000000	2.2660142	5.5623367	0.0000000	2.2714134
H	8.2957556	0.0000000	2.2537217	8.2953342	0.0000000	2.2412042
C	-8.0044856	0.0000000	0.0996127	-7.9953687	0.0000000	0.0857856
C	-7.6350868	0.0000000	1.4023811	-7.6282736	0.0000000	1.3944502
C	-6.2131717	0.0000000	1.4118920	-6.2144567	0.0000000	1.4142219
H	-5.5571609	0.0000000	2.2660142	-5.5623367	0.0000000	2.2714134
H	-8.9579331	0.0000000	-0.4000981	-8.9493910	0.0000000	-0.4134834
H	-8.2957556	0.0000000	2.2537217	-8.2953342	0.0000000	2.2412042
H	8.9579331	0.0000000	-0.4000981	8.9493910	0.0000000	-0.4134834
**6O/ PBE0**	**Ground state**			**Excited state**		
O	1.7955626	-0.0149158	0.0000000	1.8010614	0.0107363	0.0000000
C	0.5228434	-0.4846099	0.0000000	0.5189176	-0.4640284	0.0000000
C	0.5289798	-1.8509896	0.0000000	0.5367796	-1.8588996	0.0000000

C	1.8876446	-2.2434744	0.0000000	1.8703641	-2.2430171	0.0000000
C	2.6214534	-1.0909223	0.0000000	2.6283164	-1.0761262	0.0000000
C	4.0233469	-0.8288035	0.0000000	4.0062217	-0.8415852	0.0000000
O	4.8488455	-1.9054686	0.0000000	4.8368694	-1.9245862	0.0000000
C	6.1209359	-1.4365705	0.0000000	6.1119001	-1.4510332	0.0000000
C	6.1168473	-0.0715334	0.0000000	6.1016474	-0.0713445	0.0000000
C	4.7577793	0.3228426	0.0000000	4.7617294	0.3230847	0.0000000
H	6.9882408	0.5615365	0.0000000	6.9759140	0.5583922	0.0000000
H	2.2853325	-3.2444087	0.0000000	2.2707048	-3.2432385	0.0000000
H	-0.3411772	-2.4857093	0.0000000	-0.3352893	-2.4913968	0.0000000
C	7.1691173	-2.4084049	0.0000000	7.1579709	-2.4027583	0.0000000
O	8.4395904	-1.9363041	0.0000000	8.4319325	-1.9269912	0.0000000
C	9.2620775	-3.0090971	0.0000000	9.2553676	-2.9986725	0.0000000
C	8.5453696	-4.1580201	0.0000000	8.5398867	-4.1525963	0.0000000
C	7.1767839	-3.7722205	0.0000000	7.1746801	-3.7763538	0.0000000
H	6.3093609	-4.4105425	0.0000000	6.3098987	-4.4183072	0.0000000
H	10.3168102	-2.7938853	0.0000000	10.3102626	-2.7831493	0.0000000
H	8.9435779	-5.1593631	0.0000000	8.9440304	-5.1518124	0.0000000
C	-0.5228434	0.4846099	0.0000000	-0.5189176	0.4640284	0.0000000
O	-1.7955626	0.0149158	0.0000000	-1.8010614	-0.0107363	0.0000000
C	-2.6214534	1.0909223	0.0000000	-2.6283164	1.0761262	0.0000000
C	-1.8876446	2.2434744	0.0000000	-1.8703641	2.2430171	0.0000000
C	-0.5289798	1.8509896	0.0000000	-0.5367796	1.8588996	0.0000000
H	-2.2853325	3.2444087	0.0000000	-2.2707048	3.2432385	0.0000000
H	0.3411772	2.4857093	0.0000000	0.3352893	2.4913968	0.0000000
H	4.3612956	1.3242611	0.0000000	4.3656934	1.3248581	0.0000000
C	-4.0233469	0.8288035	0.0000000	-4.0062217	0.8415852	0.0000000
C	-4.7577793	-0.3228426	0.0000000	-4.7617294	-0.3230847	0.0000000
C	-6.1168473	0.0715334	0.0000000	-6.1016474	0.0713445	0.0000000
C	-6.1209359	1.4365705	0.0000000	-6.1119001	1.4510332	0.0000000
O	-4.8488455	1.9054686	0.0000000	-4.8368694	1.9245862	0.0000000
H	-6.9882408	-0.5615365	0.0000000	-6.9759140	-0.5583922	0.0000000
H	-4.3612956	-1.3242611	0.0000000	-4.3656934	-1.3248581	0.0000000
C	-7.1691173	2.4084049	0.0000000	-7.1579709	2.4027583	0.0000000
C	-7.1767839	3.7722205	0.0000000	-7.1746801	3.7763538	0.0000000
C	-8.5453696	4.1580201	0.0000000	-8.5398867	4.1525963	0.0000000
C	-9.2620775	3.0090971	0.0000000	-9.2553676	2.9986725	0.0000000
O	-8.4395904	1.9363041	0.0000000	-8.4319325	1.9269912	0.0000000
H	-8.9435779	5.1593631	0.0000000	-8.9440304	5.1518124	0.0000000
H	-10.3168102	2.7938853	0.0000000	-10.310262	2.7831493	0.0000000
H	-6.3093609	4.4105425	0.0000000	-6.3098987	4.4183072	0.0000000
**7O/ PBE0**	**Ground state**			**Excited state**		
C	-9.6712412	0.0000000	-1.4872084	-9.6677781	0.0000000	-1.4920121
C	-9.2849801	0.0000000	-0.1792117	-9.2729820	0.0000000	-0.1790145
O	-10.3742669	0.0000000	0.6272762	-10.364066	0.0000000	0.6307268
C	-11.4624095	0.0000000	-0.1747739	-11.452588	0.0000000	-0.1702014
C	-11.0931253	0.0000000	-1.4775906	-11.085265	0.0000000	-1.4763211
C	-8.0080858	0.0000000	0.4631824	-8.0021054	0.0000000	0.4496456

O	-6.9162984	0.0000000	-0.3405735	-6.9085222	0.0000000	-0.3564801
C	-5.8242552	0.0000000	0.4644872	-5.8117039	0.0000000	0.4528345
C	-6.2099431	0.0000000	1.7748038	-6.2123700	0.0000000	1.7770326
C	-7.6250512	0.0000000	1.7734022	-7.6128339	0.0000000	1.7696872
C	-4.5503255	0.0000000	-0.1766369	-4.5499584	0.0000000	-0.1598922
O	-3.4580822	0.0000000	0.6275829	-3.4543454	0.0000000	0.6518710
C	-2.3659096	0.0000000	-0.1771889	-2.3550350	0.0000000	-0.1582010
C	-2.7514112	0.0000000	-1.4881161	-2.7581410	0.0000000	-1.4901223
C	-4.1655714	0.0000000	-1.4877249	-4.1492222	0.0000000	-1.4875694
C	-1.0922263	0.0000000	0.4633387	-1.0982494	0.0000000	0.4468739
O	0.0000000	0.0000000	-0.3412158	0.0000000	0.0000000	-0.3656048
C	1.0922263	0.0000000	0.4633387	1.0982494	0.0000000	0.4468739
C	0.7069957	0.0000000	1.7744589	0.6938825	0.0000000	1.7795672
C	-0.7069957	0.0000000	1.7744589	-0.6938825	0.0000000	1.7795672
C	2.3659096	0.0000000	-0.1771889	2.3550350	0.0000000	-0.1582010
O	3.4580822	0.0000000	0.6275829	3.4543454	0.0000000	0.6518710
C	4.5503255	0.0000000	-0.1766369	4.5499584	0.0000000	-0.1598922
C	4.1655714	0.0000000	-1.4877249	4.1492222	0.0000000	-1.4875694
C	2.7514112	0.0000000	-1.4881161	2.7581410	0.0000000	-1.4901223
C	5.8242552	0.0000000	0.4644872	5.8117039	0.0000000	0.4528345
O	6.9162984	0.0000000	-0.3405735	6.9085222	0.0000000	-0.3564801
C	8.0080858	0.0000000	0.4631824	8.0021054	0.0000000	0.4496456
C	7.6250512	0.0000000	1.7734022	7.6128339	0.0000000	1.7696872
C	6.2099431	0.0000000	1.7748038	6.2123700	0.0000000	1.7770326
C	9.2849801	0.0000000	-0.1792117	9.2729820	0.0000000	-0.1790145
C	9.6712412	0.0000000	-1.4872084	9.6677781	0.0000000	-1.4920121
C	11.0931253	0.0000000	-1.4775906	11.0852654	0.0000000	-1.4763211
C	11.4624095	0.0000000	-0.1747739	11.4525882	0.0000000	-0.1702014
O	10.3742669	0.0000000	0.6272762	10.3640663	0.0000000	0.6307268
H	-1.3667800	0.0000000	2.6257631	-1.3557994	0.0000000	2.6295588
H	2.0918709	0.0000000	-2.3396160	2.0972167	0.0000000	-2.3408025
H	4.8256537	0.0000000	-2.3387886	4.8116050	0.0000000	-2.3372666
H	-2.0918709	0.0000000	-2.3396160	-2.0972167	0.0000000	-2.3408025
H	-4.8256537	0.0000000	-2.3387886	-4.8116050	0.0000000	-2.3372666
H	8.2863233	0.0000000	2.6235891	8.2779744	0.0000000	2.6172557
H	5.5509594	0.0000000	2.6267325	5.5548980	0.0000000	2.6302750
H	1.3667800	0.0000000	2.6257631	1.3557994	0.0000000	2.6295588
H	11.7538577	0.0000000	-2.3288764	11.7497664	0.0000000	-2.3248865
H	9.0153193	0.0000000	-2.3414009	9.0145809	0.0000000	-2.3483078
H	-5.5509594	0.0000000	2.6267325	-5.5548980	0.0000000	2.6302750
H	-8.2863233	0.0000000	2.6235891	-8.2779744	0.0000000	2.6172557
H	-9.0153193	0.0000000	-2.3414009	-9.0145809	0.0000000	-2.3483078
H	-11.7538577	0.0000000	-2.3288764	-11.749766	0.0000000	-2.3248865
H	-12.4158311	0.0000000	0.3249908	-12.406048	0.0000000	0.3298446
H	12.4158311	0.0000000	0.3249908	12.4060488	0.0000000	0.3298446
**8O/ PBE0**	**Ground state**			**Excited state**		
O	-11.8415162	2.5496746	0.0000000	-11.831959	2.5442751	0.0000000
C	-10.5854893	3.0589560	0.0000000	-10.574135	3.0558845	0.0000000

C	-10.6332663	4.4219533	0.0000000	-10.627597	4.4240663	0.0000000
C	-12.0125923	4.7673226	0.0000000	-12.004928	4.7638358	0.0000000
C	-12.6951827	3.5977976	0.0000000	-12.686359	3.5913418	0.0000000
C	-9.5092063	2.1183479	0.0000000	-9.4996275	2.1266798	0.0000000
C	-9.4650962	0.7539887	0.0000000	-9.4532436	0.7537807	0.0000000
C	-8.0950735	0.3996651	0.0000000	-8.0958104	0.3972633	0.0000000
C	-7.3947414	1.5723614	0.0000000	-7.3806891	1.5772294	0.0000000
O	-8.2514613	2.6243641	0.0000000	-8.2401575	2.6334631	0.0000000
C	-6.0011796	1.8755282	0.0000000	-6.0025478	1.8602896	0.0000000
O	-5.1439969	0.8243672	0.0000000	-5.1437089	0.8034953	0.0000000
C	-3.8856438	1.3313974	0.0000000	-3.8784816	1.3132578	0.0000000
C	-3.9320895	2.6970435	0.0000000	-3.9363221	2.7000030	0.0000000
C	-5.3016520	3.0492849	0.0000000	-5.2875737	3.0441745	0.0000000
C	-2.8119405	0.3935064	0.0000000	-2.8107831	0.4103992	0.0000000
O	-1.5536156	0.9002273	0.0000000	-1.5459340	0.9228343	0.0000000
C	-0.6965372	-0.1513221	0.0000000	-0.6841363	-0.1362253	0.0000000
C	-1.3966414	-1.3249510	0.0000000	-1.4070676	-1.3253555	0.0000000
C	-2.7658911	-0.9723106	0.0000000	-2.7522962	-0.9779861	0.0000000
C	0.6965372	0.1513221	0.0000000	0.6841363	0.1362253	0.0000000
O	1.5536156	-0.9002273	0.0000000	1.5459340	-0.9228343	0.0000000
C	2.8119405	-0.3935064	0.0000000	2.8107831	-0.4103992	0.0000000
C	2.7658911	0.9723106	0.0000000	2.7522962	0.9779861	0.0000000
C	1.3966414	1.3249510	0.0000000	1.4070676	1.3253555	0.0000000
C	3.8856438	-1.3313974	0.0000000	3.8784816	-1.3132578	0.0000000
O	5.1439969	-0.8243672	0.0000000	5.1437089	-0.8034953	0.0000000
C	6.0011796	-1.8755282	0.0000000	6.0025478	-1.8602896	0.0000000
C	5.3016520	-3.0492849	0.0000000	5.2875737	-3.0441745	0.0000000
C	3.9320895	-2.6970435	0.0000000	3.9363221	-2.7000030	0.0000000
H	3.6171649	1.6321302	0.0000000	3.6051690	1.6362231	0.0000000
H	-0.9700427	-2.3139167	0.0000000	-0.9786541	-2.3137896	0.0000000
H	-3.6171649	-1.6321302	0.0000000	-3.6051690	-1.6362231	0.0000000
H	3.0810560	-3.3571896	0.0000000	3.0850682	-3.3601254	0.0000000
C	7.3947414	-1.5723614	0.0000000	7.3806891	-1.5772294	0.0000000
H	5.7286890	-4.0380487	0.0000000	5.7174695	-4.0320464	0.0000000
H	-5.7286890	4.0380487	0.0000000	-5.7174695	4.0320464	0.0000000
H	-3.0810560	3.3571896	0.0000000	-3.0850682	3.3601254	0.0000000
H	0.9700427	2.3139167	0.0000000	0.9786541	2.3137896	0.0000000
H	-10.3175431	0.0956337	0.0000000	-10.308368	0.0984795	0.0000000
H	-7.6694113	-0.5897069	0.0000000	-7.6721230	-0.5930425	0.0000000
H	-12.4401107	5.7565046	0.0000000	-12.435940	5.7516374	0.0000000
H	-13.7431278	3.3516447	0.0000000	-13.734319	3.3447085	0.0000000
H	-9.7850017	5.0855254	0.0000000	-9.7811439	5.0899543	0.0000000
O	8.2514613	-2.6243641	0.0000000	8.2401575	-2.6334631	0.0000000
C	9.5092063	-2.1183479	0.0000000	9.4996275	-2.1266798	0.0000000
C	9.4650962	-0.7539887	0.0000000	9.4532436	-0.7537807	0.0000000
C	8.0950735	-0.3996651	0.0000000	8.0958104	-0.3972633	0.0000000
H	7.6694113	0.5897069	0.0000000	7.6721230	0.5930425	0.0000000
H	10.3175431	-0.0956337	0.0000000	10.3083683	-0.0984795	0.0000000
C	10.5854893	-3.0589560	0.0000000	10.5741351	-3.0558845	0.0000000
O	11.8415162	-2.5496746	0.0000000	11.8319598	-2.5442751	0.0000000

C	12.6951827	-3.5977976	0.0000000	12.6863594	-3.5913418	0.0000000
C	12.0125923	-4.7673226	0.0000000	12.0049287	-4.7638358	0.0000000
C	10.6332663	-4.4219533	0.0000000	10.6275970	-4.4240663	0.0000000
H	9.7850017	-5.0855254	0.0000000	9.7811439	-5.0899543	0.0000000
H	12.4401107	-5.7565046	0.0000000	12.4359403	-5.7516374	0.0000000
H	13.7431278	-3.3516447	0.0000000	13.7343198	-3.3447085	0.0000000
**9O/ PBE0**	**Ground state**			**Excited state**		
C	-13.1291733	0.0000000	-1.5237444	-13.122630	0.0000000	-1.5269235
C	-12.7428928	0.0000000	-0.2157564	-12.731110	0.0000000	-0.2162461
O	-13.8321544	0.0000000	0.5907582	-13.821229	0.0000000	0.5923546
C	-14.9202976	0.0000000	-0.2112673	-14.909698	0.0000000	-0.2086599
C	-14.5510474	0.0000000	-1.5140995	-14.541951	0.0000000	-1.5134874
C	-11.4659972	0.0000000	0.4266312	-11.457788	0.0000000	0.4180933
O	-10.3742153	0.0000000	-0.3770918	-10.364731	0.0000000	-0.3860067
C	-9.2821949	0.0000000	0.4280045	-9.2699050	0.0000000	0.4219714
C	-9.6679106	0.0000000	1.7383080	-9.6660698	0.0000000	1.7405585
C	-11.0830027	0.0000000	1.7368787	-11.071904	0.0000000	1.7344621
C	-8.0082710	0.0000000	-0.2131139	-8.0039688	0.0000000	-0.2000775
O	-6.9160305	0.0000000	0.5910459	-6.9092134	0.0000000	0.6075038
C	-5.8238936	0.0000000	-0.2137989	-5.8120573	0.0000000	-0.2008660
C	-6.2094602	0.0000000	-1.5247062	-6.2120602	0.0000000	-1.5263489
C	-7.6235879	0.0000000	-1.5242547	-7.6095401	0.0000000	-1.5223979
C	-4.5502231	0.0000000	0.4266864	-4.5508809	0.0000000	0.4111636
O	-3.4580075	0.0000000	-0.3777731	-3.4544516	0.0000000	-0.3990348
C	-2.3658105	0.0000000	0.4268924	-2.3562031	0.0000000	0.4105903
C	-2.7511840	0.0000000	1.7380137	-2.7588142	0.0000000	1.7405110
C	-4.1650922	0.0000000	1.7379090	-4.1504547	0.0000000	1.7385583
C	-1.0922095	0.0000000	-0.2135100	-1.0978571	0.0000000	-0.1959349
O	0.0000000	0.0000000	0.5910430	0.0000000	0.0000000	0.6147979
C	1.0922095	0.0000000	-0.2135100	1.0978571	0.0000000	-0.1959349
C	0.7069360	0.0000000	-1.5247119	0.6947563	0.0000000	-1.5263998
C	-0.7069360	0.0000000	-1.5247119	-0.6947563	0.0000000	-1.5263998
C	2.3658105	0.0000000	0.4268924	2.3562031	0.0000000	0.4105903
O	3.4580075	0.0000000	-0.3777731	3.4544516	0.0000000	-0.3990348
C	4.5502231	0.0000000	0.4266864	4.5508809	0.0000000	0.4111636
C	4.1650922	0.0000000	1.7379090	4.1504547	0.0000000	1.7385583
C	2.7511840	0.0000000	1.7380137	2.7588142	0.0000000	1.7405110
C	5.8238936	0.0000000	-0.2137989	5.8120573	0.0000000	-0.2008660
C	6.2094602	0.0000000	-1.5247062	6.2120602	0.0000000	-1.5263489
C	7.6235879	0.0000000	-1.5242547	7.6095401	0.0000000	-1.5223979
C	8.0082710	0.0000000	-0.2131139	8.0039688	0.0000000	-0.2000775
O	6.9160305	0.0000000	0.5910459	6.9092134	0.0000000	0.6075038
C	9.2821949	0.0000000	0.4280045	9.2699050	0.0000000	0.4219714
O	10.3742153	0.0000000	-0.3770918	10.3647310	0.0000000	-0.3860067
C	11.4659972	0.0000000	0.4266312	11.4577885	0.0000000	0.4180933
C	11.0830027	0.0000000	1.7368787	11.0719045	0.0000000	1.7344621
C	9.6679106	0.0000000	1.7383080	9.6660698	0.0000000	1.7405585
H	-4.8249477	0.0000000	2.5891515	-4.8123769	0.0000000	2.5885810

H	-1.3666971	0.0000000	-2.3760328	-1.3561725	0.0000000	-2.3767673
H	1.3666971	0.0000000	-2.3760328	1.3561725	0.0000000	-2.3767673
H	-5.5499861	0.0000000	-2.3762612	-5.5529303	0.0000000	-2.3783281
H	-8.2837075	0.0000000	-2.3752849	-8.2721576	0.0000000	-2.3718510
H	4.8249477	0.0000000	2.5891515	4.8123769	0.0000000	2.5885810
H	2.0915121	0.0000000	2.5894102	2.0982380	0.0000000	2.5914537
H	-2.0915121	0.0000000	2.5894102	-2.0982380	0.0000000	2.5914537
H	8.2837075	0.0000000	-2.3752849	8.2721576	0.0000000	-2.3718510
H	5.5499861	0.0000000	-2.3762612	5.5529303	0.0000000	-2.3783281
H	11.7442953	0.0000000	2.5870471	11.7361440	0.0000000	2.5825951
C	12.7428928	0.0000000	-0.2157564	12.7311101	0.0000000	-0.2162461
H	9.0089621	0.0000000	2.5902659	9.0088085	0.0000000	2.5938888
H	-9.0089621	0.0000000	2.5902659	-9.0088085	0.0000000	2.5938888
H	-11.7442953	0.0000000	2.5870471	-11.736144	0.0000000	2.5825951
H	-12.4732790	0.0000000	-2.3779592	-12.468566	0.0000000	-2.3825420
H	-15.2117968	0.0000000	-2.3653699	-15.205055	0.0000000	-2.3630418
H	-15.8737127	0.0000000	0.2885114	-15.863008	0.0000000	0.2915286
O	13.8321544	0.0000000	0.5907582	13.8212290	0.0000000	0.5923546
C	14.9202976	0.0000000	-0.2112673	14.9096989	0.0000000	-0.2086599
C	14.5510474	0.0000000	-1.5140995	14.5419517	0.0000000	-1.5134874
C	13.1291733	0.0000000	-1.5237444	13.1226300	0.0000000	-1.5269235
H	12.4732790	0.0000000	-2.3779592	12.4685662	0.0000000	-2.3825420
H	15.2117968	0.0000000	-2.3653699	15.2050558	0.0000000	-2.3630418
H	15.8737127	0.0000000	0.2885114	15.8630086	0.0000000	0.2915286

Part 5.

2O/ MN15	Ground state			Excited state		
C	-0.675128	2.759693	0.0000000	-0.688555	2.742964	0.000000
C	0.675128	2.861322	0.0000000	0.688555	2.840332	0.000000
C	1.175267	1.523793	0.0000000	1.199642	1.543930	0.000000
C	0.080960	0.714963	0.0000000	0.066450	0.685413	0.000000
C	-0.080960	-0.714963	0.0000000	-0.066450	-0.685413	-0.000000
C	-1.175267	-1.523793	0.0000000	-1.199642	-1.543930	-0.000000
C	-0.675128	-2.861322	0.0000000	-0.688555	-2.840332	-0.000000
C	0.675128	-2.759693	0.0000000	0.688555	-2.742964	-0.000000
O	-1.050765	1.458875	0.0000000	-1.089818	1.444383	0.000000
O	1.050765	-1.458875	0.0000000	1.089818	-1.444383	-0.000000
H	-1.470199	3.483640	0.0000000	-1.469158	3.484068	0.000000
H	1.246152	3.773834	0.0000000	1.243871	3.763434	0.000000
H	2.200642	1.196954	0.0000000	2.224872	1.216497	0.000000
H	-2.200642	-1.196954	0.0000000	-2.224872	-1.216497	-0.000000
H	-1.246152	-3.773834	0.0000000	-1.243871	-3.763434	-0.000000
H	1.470199	-3.483640	0.0000000	1.469158	-3.484068	-0.000000
3O/ MN15	Ground state			Excited state		
O	0.000000	3.464974	-0.713390	-0.000000	3.463632	-0.728483
C	-0.000000	4.555854	0.088428	-0.000000	4.538924	0.096144

C	-0.000000	4.185007	1.390938	-0.000000	4.146660	1.405127
C	-0.000000	2.757546	1.398861	-0.000000	2.737856	1.409939
C	-0.000000	2.375683	0.091887	-0.000000	2.351178	0.068551
C	0.000000	1.092100	-0.553514	-0.000000	1.100181	-0.540676
O	-0.000000	0.000000	0.250788	0.000000	-0.000000	0.283644
C	-0.000000	-1.092100	-0.553514	-0.000000	-1.100181	-0.540676
C	0.000000	-0.711449	-1.861649	0.000000	-0.689888	-1.889845
C	0.000000	0.711449	-1.861649	0.000000	0.689888	-1.889845
C	-0.000000	-2.375683	0.091887	-0.000000	-2.351178	0.068551
O	-0.000000	-3.464974	-0.713390	-0.000000	-3.463632	-0.728483
C	-0.000000	-4.555854	0.088428	-0.000000	-4.538924	0.096144
C	-0.000000	-4.185007	1.390938	-0.000000	-4.146660	1.405127
C	-0.000000	-2.757546	1.398861	-0.000000	-2.737856	1.409939
H	0.000000	5.509876	-0.407618	-0.000000	5.504192	-0.379141
H	-0.000000	4.844355	2.241811	-0.000000	4.804460	2.257951
H	-0.000000	2.097488	2.248842	-0.000000	2.067477	2.252281
H	-0.000000	-2.097488	2.248842	-0.000000	-2.067477	2.252281
H	-0.000000	-5.509876	-0.407618	-0.000000	-5.504192	-0.379141
H	-0.000000	-4.844355	2.241811	-0.000000	-4.804460	2.257951
H	0.000000	1.375323	-2.708778	-0.000000	1.359037	-2.733244
H	-0.000000	-1.375323	-2.708778	-0.000000	-1.359037	-2.733244
**4O/ MN15**	**Ground state**			**Excited state**		
O	-0.307939	1.772253	-0.000000	-0.354471	1.769954	-0.000000
C	0.600655	2.779161	0.000000	0.561627	2.784111	-0.000000
C	1.864962	2.270620	0.000000	1.853469	2.259760	-0.000000
C	1.724454	0.855376	0.000000	1.732139	0.877955	-0.000000
C	0.384201	0.605386	0.000000	0.354471	0.595243	-0.000000
C	-0.384201	-0.605386	0.000000	-0.354471	-0.595243	-0.000000
O	0.307939	-1.772253	-0.000000	0.354471	-1.769954	-0.000000
C	-0.600655	-2.779161	0.000000	-0.561627	-2.784111	-0.000000
C	-1.864962	-2.270620	0.000000	-1.853469	-2.259760	-0.000000
C	-1.724454	-0.855376	0.000000	-1.732139	-0.877955	-0.000000
C	-0.086586	-4.120444	-0.000000	-0.069359	-4.100668	0.000000
O	-0.996741	-5.123848	-0.000000	-0.974717	-5.119681	0.000000
C	-0.307939	-6.289287	-0.000000	-0.272255	-6.276794	0.000000
C	1.025157	-6.050521	0.000000	1.065835	-6.025607	0.000000
C	1.175764	-4.631090	0.000000	1.212844	-4.617441	0.000000
H	2.773571	2.847466	0.000000	2.754948	2.848577	-0.000000
H	2.502287	0.111583	0.000000	2.513262	0.137138	-0.000000
H	2.087545	-4.059443	0.000000	2.121109	-4.039804	0.000000
H	-0.896782	-7.189000	-0.000000	-0.849502	-7.184501	0.000000
H	1.805878	-6.791566	0.000000	1.848147	-6.765571	0.000000
H	-2.502287	-0.111583	0.000000	-2.513262	-0.137138	-0.000000
H	-2.773571	-2.847466	0.000000	-2.754948	-2.848577	-0.000000
C	0.086586	4.120444	-0.000000	0.069359	4.100668	0.000000
O	0.996741	5.123848	-0.000000	0.974717	5.119681	0.000000
C	0.307939	6.289287	-0.000000	0.272255	6.276794	0.000000
C	-1.025157	6.050521	0.000000	-1.065835	6.025607	0.000000

C	-1.175764	4.631090	0.000000	-1.212844	4.617441	0.000000
H	-2.087545	4.059443	0.000000	-2.121109	4.039804	0.000000
H	0.896782	7.189000	-0.000000	0.849502	7.184501	0.000000
H	-1.805878	6.791566	0.000000	-1.848147	6.765571	0.000000
**5O/ MN15**	**Ground state**			**Excited state**		
O	3.4656174	0.0000000	0.2634834	0.000000	3.465535	-0.342374
C	4.5579164	0.0000000	-0.5403334	0.000000	4.557904	0.461324
C	4.1778183	0.0000000	-1.8490906	0.000000	4.177932	1.770130
C	2.7557729	0.0000000	-1.8503003	0.000000	2.755886	1.771469
C	2.3734238	0.0000000	-0.5415039	0.000000	2.373424	0.462694
C	1.0924518	0.0000000	0.1023650	0.000000	1.092439	-0.181143
O	0.0000000	0.0000000	-0.7020291	0.000000	-0.000000	0.623242
C	-1.0924518	0.0000000	0.1023650	-0.000000	-1.092439	-0.181143
C	-0.7107322	0.0000000	1.4116147	0.000000	-0.710728	-1.490408
C	0.7107322	0.0000000	1.4116147	0.000000	0.710728	-1.490408
C	-2.3734238	0.0000000	-0.5415039	-0.000000	-2.373424	0.462694
O	-3.4656174	0.0000000	0.2634834	-0.000000	-3.465535	-0.342374
C	-4.5579164	0.0000000	-0.5403334	-0.000000	-4.557904	0.461324
C	-4.1778183	0.0000000	-1.8490906	-0.000000	-4.177932	1.770130
C	-2.7557729	0.0000000	-1.8503003	-0.000000	-2.755886	1.771469
C	5.8413408	0.0000000	0.1046223	0.000000	5.841187	-0.183904
O	6.9303757	0.0000000	-0.7010950	0.000000	6.930464	0.621463
C	8.0213915	0.0000000	0.1003632	0.000000	8.021212	-0.180356
C	7.6510317	0.0000000	1.4030803	0.000000	7.650424	-1.482965
C	6.2237083	0.0000000	1.4115620	0.000000	6.223116	-1.490980
C	-5.8413408	0.0000000	0.1046223	-0.000000	-5.841187	-0.183904
O	-6.9303757	0.0000000	-0.7010950	-0.000000	-6.930464	0.621463
H	-4.8422747	0.0000000	-2.6957347	0.000000	-4.842507	2.616694
H	-2.0932659	0.0000000	-2.6984425	0.000000	-2.093419	2.619656
H	-1.3739771	0.0000000	2.2591697	0.000000	-1.374017	-2.337942
H	1.3739771	0.0000000	2.2591697	0.000000	1.374017	-2.337942
H	2.0932659	0.0000000	-2.6984425	0.000000	2.093419	2.619656
H	4.8422747	0.0000000	-2.6957347	0.000000	4.842507	2.616694
H	5.5641254	0.0000000	2.2619012	0.000000	5.563209	-2.341080
H	8.3106149	0.0000000	2.2537370	0.000000	8.309791	-2.333803
C	-8.0213915	0.0000000	0.1003632	-0.000000	-8.021212	-0.180356
C	-7.6510317	0.0000000	1.4030803	-0.000000	-7.650424	-1.482965
C	-6.2237083	0.0000000	1.4115620	-0.000000	-6.223116	-1.490980
H	-5.5641254	0.0000000	2.2619012	-0.000000	-5.563209	-2.341080
H	-8.9752988	0.0000000	-0.3959153	0.000000	-8.975263	0.315637
H	-8.3106149	0.0000000	2.2537370	-0.000000	-8.309791	-2.333803
H	8.9752988	0.0000000	-0.3959153	0.000000	8.975263	0.315637
**6O/ MN15**	**Ground state**			**Excited state**		
O	-0.512586	1.724185	0.000000	-0.549644	1.718290	0.000000
C	0.310692	0.645806	0.000000	0.285364	0.634380	0.000000
C	1.613237	1.050276	0.000000	1.615183	1.069399	0.000000

C	1.588535	2.471364	0.000000	1.582616	2.457632	0.000000
C	0.272740	2.830335	0.000000	0.240160	2.832758	0.000000
C	-0.393063	4.099983	0.000000	-0.392564	4.081703	0.000000
O	0.393063	5.205820	0.000000	0.392564	5.198363	0.000000
C	-0.429338	6.284128	0.000000	-0.438228	6.274345	0.000000
C	-1.731432	5.881655	0.000000	-1.751392	5.856627	0.000000
C	-1.708256	4.459838	0.000000	-1.729821	4.454928	0.000000
H	-2.589345	6.531509	0.000000	-2.609031	6.507481	0.000000
H	2.424408	3.149277	0.000000	2.413542	3.142225	0.000000
H	2.472259	0.401927	0.000000	2.475782	0.422626	0.000000
C	0.193591	7.578362	0.000000	0.160953	7.563111	0.000000
O	-0.630496	8.653543	0.000000	-0.671493	8.636668	0.000000
C	0.152353	9.757968	0.000000	0.104788	9.745086	0.000000
C	1.461191	9.409749	0.000000	1.419057	9.405302	0.000000
C	1.493868	7.982810	0.000000	1.465463	7.984026	0.000000
H	2.355288	7.337747	0.000000	2.332310	7.346165	0.000000
H	-0.360046	10.703307	0.000000	-0.412385	10.688058	0.000000
H	2.300503	10.083721	0.000000	2.251942	10.087509	0.000000
C	-0.310692	-0.645806	0.000000	-0.285364	-0.634380	0.000000
O	0.512586	-1.724185	0.000000	0.549644	-1.718290	0.000000
C	-0.272740	-2.830335	0.000000	-0.240160	-2.832758	0.000000
C	-1.588535	-2.471364	0.000000	-1.582616	-2.457632	0.000000
C	-1.613237	-1.050276	0.000000	-1.615183	-1.069399	0.000000
H	-2.424408	-3.149277	0.000000	-2.413542	-3.142225	0.000000
H	-2.472259	-0.401927	0.000000	-2.475782	-0.422626	0.000000
H	-2.544898	3.782849	0.000000	-2.564820	3.775589	0.000000
C	0.393063	-4.099983	0.000000	0.392564	-4.081703	0.000000
C	1.708256	-4.459838	0.000000	1.729821	-4.454928	0.000000
C	1.731432	-5.881655	0.000000	1.751392	-5.856627	0.000000
C	0.429338	-6.284128	0.000000	0.438228	-6.274345	0.000000
O	-0.393063	-5.205820	0.000000	-0.392564	-5.198363	0.000000
H	2.589345	-6.531509	0.000000	2.609031	-6.507481	0.000000
H	2.544898	-3.782849	0.000000	2.564820	-3.775589	0.000000
C	-0.193591	-7.578362	0.000000	-0.160953	-7.563111	0.000000
C	-1.493868	-7.982810	0.000000	-1.465463	-7.984026	0.000000
C	-1.461191	-9.409749	0.000000	-1.419057	-9.405302	0.000000
C	-0.152353	-9.757968	0.000000	-0.104788	-9.745086	0.000000
O	0.630496	-8.653543	0.000000	0.671493	-8.636668	0.000000
H	-2.300503	-10.083721	0.000000	-2.251942	-10.087509	0.000000
H	0.360046	-10.703307	0.000000	0.412385	-10.688058	0.000000
H	-2.355288	-7.337747	0.000000	-2.332310	-7.346165	0.000000
**7O/ MN15**	**Ground state**			**Excited state**		
C	0.000000	9.688272	1.531453	-0.000000	9.679534	1.536985
C	0.000000	9.306517	0.224320	-0.000000	9.290517	0.225498
O	0.000000	10.395877	-0.580921	-0.000000	10.381538	-0.582012
C	0.000000	11.486492	0.221020	-0.000000	11.472377	0.218839
C	0.000000	11.115554	1.523608	-0.000000	11.103059	1.524139
C	0.000000	8.023350	-0.421113	-0.000000	8.013266	-0.407631

O	0.000000	6.930835	0.382295	-0.000000	6.919027	0.396553
C	0.000000	5.838933	-0.423085	-0.000000	5.822294	-0.412584
C	0.000000	6.221742	-1.731773	-0.000000	6.221773	-1.735532
C	0.000000	7.643728	-1.730069	-0.000000	7.628629	-1.726440
C	0.000000	4.557871	0.220441	-0.000000	4.555243	0.199869
O	0.000000	3.465558	-0.583957	-0.000000	3.459554	-0.611026
C	0.000000	2.373046	0.220502	-0.000000	2.358537	0.198031
C	0.000000	2.754870	1.529882	-0.000000	2.762227	1.532395
C	0.000000	4.176128	1.529845	-0.000000	4.156011	1.528444
C	0.000000	1.092424	-0.423113	-0.000000	1.099578	-0.402968
O	-0.000000	-0.000000	0.381349	-0.000000	0.000000	0.408953
C	-0.000000	-1.092424	-0.423113	-0.000000	-1.099578	-0.402968
C	-0.000000	-0.710577	-1.732582	0.000000	-0.694108	-1.739222
C	0.000000	0.710577	-1.732582	0.000000	0.694108	-1.739222
C	-0.000000	-2.373046	0.220502	-0.000000	-2.358537	0.198031
O	-0.000000	-3.465558	-0.583957	-0.000000	-3.459554	-0.611026
C	-0.000000	-4.557871	0.220441	-0.000000	-4.555243	0.199869
C	-0.000000	-4.176128	1.529845	-0.000000	-4.156011	1.528444
C	-0.000000	-2.754870	1.529882	-0.000000	-2.762227	1.532395
C	-0.000000	-5.838933	-0.423085	-0.000000	-5.822294	-0.412584
O	-0.000000	-6.930835	0.382295	-0.000000	-6.919027	0.396553
C	-0.000000	-8.023350	-0.421113	-0.000000	-8.013266	-0.407631
C	-0.000000	-7.643728	-1.730069	-0.000000	-7.628629	-1.726440
C	-0.000000	-6.221742	-1.731773	-0.000000	-6.221773	-1.735532
C	-0.000000	-9.306517	0.224320	-0.000000	-9.290517	0.225498
C	-0.000000	-9.688272	1.531453	-0.000000	-9.679534	1.536985
C	-0.000000	-11.115554	1.523608	-0.000000	-11.103059	1.524139
C	-0.000000	-11.486492	0.221020	-0.000000	-11.472377	0.218839
O	-0.000000	-10.395877	-0.580921	-0.000000	-10.381538	-0.582012
H	0.000000	1.373755	-2.580198	-0.000000	1.359622	-2.585444
H	-0.000000	-2.091708	2.377517	0.000000	-2.098367	2.379809
H	-0.000000	-4.839415	2.377365	0.000000	-4.822463	2.373967
H	0.000000	2.091708	2.377517	0.000000	2.098367	2.379809
H	0.000000	4.839415	2.377365	0.000000	4.822463	2.373967
H	-0.000000	-8.308520	-2.576451	0.000000	-8.297982	-2.569609
H	-0.000000	-5.559548	-2.580172	0.000000	-5.561987	-2.585988
H	-0.000000	-1.373755	-2.580198	-0.000000	-1.359622	-2.585444
H	-0.000000	-11.774806	2.374526	0.000000	-11.765375	2.372850
H	-0.000000	-9.028294	2.381497	0.000000	-9.021846	2.388848
H	0.000000	5.559548	-2.580172	0.000000	5.561987	-2.585988
H	0.000000	8.308520	-2.576451	0.000000	8.297982	-2.569609
H	0.000000	9.028294	2.381497	0.000000	9.021846	2.388848
H	0.000000	11.774806	2.374526	0.000000	11.765375	2.372850
H	0.000000	12.440611	-0.274840	-0.000000	12.426514	-0.277309
H	-0.000000	-12.440611	-0.274840	-0.000000	-12.426514	-0.277309
**8O/ MN15**	**Ground state**			**Excited state**		
O	5.016504	11.052781	0.000000	4.879182	11.097890	0.000000
C	5.247311	9.717917	0.000000	5.126710	9.764085	0.000000

C	6.586758	9.472566	0.000000	6.474006	9.537970	0.000000
C	7.220191	10.751622	0.000000	7.087846	10.823586	0.000000
C	6.222543	11.667590	0.000000	6.077135	11.727443	0.000000
C	4.094666	8.860888	0.000000	3.993997	8.893588	0.000000
C	2.754570	9.109175	0.000000	2.643143	9.126555	0.000000
C	2.114805	7.839244	0.000000	2.017818	7.862057	0.000000
C	3.112434	6.909757	0.000000	3.029825	6.928003	0.000000
O	4.322219	7.524016	0.000000	4.236493	7.558272	0.000000
C	3.112434	5.476154	0.000000	3.029825	5.514686	0.000000
O	1.903286	4.861234	0.000000	1.823414	4.884636	0.000000
C	2.131622	3.523848	0.000000	2.067834	3.542253	0.000000
C	3.473050	3.277190	0.000000	3.438506	3.320121	0.000000
C	4.111086	4.547175	0.000000	4.047310	4.579595	0.000000
C	0.981496	2.668626	0.000000	0.969534	2.673292	0.000000
O	1.209555	1.331306	0.000000	1.216780	1.330960	0.000000
C	0.000123	0.716596	0.000000	0.006225	0.697522	0.000000
C	-0.998306	1.645954	0.000000	-1.016038	1.646742	0.000000
C	-0.359995	2.915646	0.000000	-0.404447	2.895786	0.000000
C	-0.000123	-0.716596	0.000000	-0.006225	-0.697522	0.000000
O	-1.209555	-1.331306	0.000000	-1.216780	-1.330960	0.000000
C	-0.981496	-2.668626	0.000000	-0.969534	-2.673292	0.000000
C	0.359995	-2.915646	0.000000	0.404447	-2.895786	0.000000
C	0.998306	-1.645954	0.000000	1.016038	-1.646742	0.000000
C	-2.131622	-3.523848	0.000000	-2.067834	-3.542253	0.000000
O	-1.903286	-4.861234	0.000000	-1.823414	-4.884636	0.000000
C	-3.112434	-5.476154	0.000000	-3.029825	-5.514686	0.000000
C	-4.111086	-4.547175	0.000000	-4.047310	-4.579595	0.000000
C	-3.473050	-3.277190	0.000000	-3.438506	-3.320121	0.000000
H	0.819479	-3.888849	0.000000	0.872849	-3.865121	0.000000
H	-2.053495	1.434234	0.000000	-2.068605	1.420965	0.000000
H	-0.819479	3.888849	0.000000	-0.872849	3.865121	0.000000
H	-3.932841	-2.304118	0.000000	-3.910581	-2.352733	0.000000
C	-3.112434	-6.909757	0.000000	-3.029825	-6.928003	0.000000
H	-5.166181	-4.759306	0.000000	-5.099311	-4.808169	0.000000
H	5.166181	4.759306	0.000000	5.099311	4.808169	0.000000
H	3.932841	2.304118	0.000000	3.910581	2.352733	0.000000
H	2.053495	-1.434234	0.000000	2.068605	-1.420965	0.000000
H	2.296553	10.083102	0.000000	2.177738	10.097228	0.000000
H	1.059439	7.628348	0.000000	0.964292	7.641684	0.000000
H	8.276448	10.958939	0.000000	8.141114	11.046055	0.000000
H	6.207529	12.742765	0.000000	6.049170	12.802466	0.000000
H	7.050366	8.501372	0.000000	6.951022	8.573270	0.000000
O	-4.322219	-7.524016	0.000000	-4.236493	-7.558272	0.000000
C	-4.094666	-8.860888	0.000000	-3.993997	-8.893588	0.000000
C	-2.754570	-9.109175	0.000000	-2.643143	-9.126555	0.000000
C	-2.114805	-7.839244	0.000000	-2.017818	-7.862057	0.000000
H	-1.059439	-7.628348	0.000000	-0.964292	-7.641684	0.000000
H	-2.296553	-10.083102	0.000000	-2.177738	-10.097228	0.000000
C	-5.247311	-9.717917	0.000000	-5.126710	-9.764085	0.000000
O	-5.016504	-11.052781	0.000000	-4.879182	-11.097890	0.000000

C	-6.222543	-11.667590	0.000000	-6.077135	-11.727443	0.000000
C	-7.220191	-10.751622	0.000000	-7.087846	-10.823586	0.000000
C	-6.586758	-9.472566	0.000000	-6.474006	-9.537970	0.000000
H	-7.050366	-8.501372	0.000000	-6.951022	-8.573270	0.000000
H	-8.276448	-10.958939	0.000000	-8.141114	-11.046055	0.000000
H	-6.207529	-12.742765	0.000000	-6.049170	-12.802466	0.000000
**9O/ MN15**	**Ground state**			**Excited state**		
C	-0.000000	13.153255	-1.554934	0.000000	13.141273	-1.557887
C	-0.000000	12.771770	-0.247733	0.000000	12.755707	-0.248596
O	-0.000000	13.861277	0.557302	0.000000	13.845895	0.557843
C	-0.000000	14.951719	-0.244864	0.000000	14.936595	-0.243419
C	-0.000000	14.580534	-1.547378	0.000000	14.566589	-1.547438
C	-0.000000	11.488738	0.397970	0.000000	11.475800	0.390685
O	-0.000000	10.396059	-0.405180	0.000000	10.382010	-0.411963
C	-0.000000	9.304345	0.400450	0.000000	9.287756	0.395973
C	-0.000000	9.687445	1.709048	0.000000	9.681063	1.712107
C	-0.000000	11.109420	1.707029	0.000000	11.094551	1.705216
C	-0.000000	8.023154	-0.242813	0.000000	8.014793	-0.228924
O	-0.000000	6.931009	0.561760	0.000000	6.920321	0.577636
C	-0.000000	5.838369	-0.242532	0.000000	5.822281	-0.229639
C	-0.000000	6.219966	-1.551975	0.000000	6.220329	-1.554658
C	-0.000000	7.641201	-1.552180	0.000000	7.623745	-1.549961
C	-0.000000	4.557886	0.401330	0.000000	4.556587	0.382784
O	-0.000000	3.465302	-0.402813	0.000000	3.459518	-0.426065
C	-0.000000	2.373088	0.401962	0.000000	2.360150	0.383227
C	-0.000000	2.755300	1.711337	0.000000	2.763650	1.715076
C	-0.000000	4.176403	1.710959	0.000000	4.158155	1.711436
C	-0.000000	1.092388	-0.241343	0.000000	1.099025	-0.219728
O	-0.000000	0.000000	0.563125	-0.000000	-0.000000	0.590448
C	-0.000000	-1.092388	-0.241343	-0.000000	-1.099025	-0.219728
C	0.000000	-0.710532	-1.550864	-0.000000	-0.695401	-1.553107
C	0.000000	0.710532	-1.550864	0.000000	0.695401	-1.553107
C	-0.000000	-2.373088	0.401962	-0.000000	-2.360150	0.383227
O	-0.000000	-3.465302	-0.402813	-0.000000	-3.459518	-0.426065
C	-0.000000	-4.557886	0.401330	-0.000000	-4.556587	0.382784
C	-0.000000	-4.176403	1.710959	-0.000000	-4.158155	1.711436
C	-0.000000	-2.755300	1.711337	-0.000000	-2.763650	1.715076
C	-0.000000	-5.838369	-0.242532	-0.000000	-5.822281	-0.229639
C	-0.000000	-6.219966	-1.551975	-0.000000	-6.220329	-1.554658
C	-0.000000	-7.641201	-1.552180	-0.000000	-7.623745	-1.549961
C	-0.000000	-8.023154	-0.242813	-0.000000	-8.014793	-0.228924
O	-0.000000	-6.931009	0.561760	-0.000000	-6.920321	0.577636
C	-0.000000	-9.304345	0.400450	-0.000000	-9.287756	0.395973
O	-0.000000	-10.396059	-0.405180	-0.000000	-10.382010	-0.411963
C	-0.000000	-11.488738	0.397970	-0.000000	-11.475800	0.390685
C	-0.000000	-11.109420	1.707029	-0.000000	-11.094551	1.705216
C	-0.000000	-9.687445	1.709048	-0.000000	-9.681063	1.712107
H	-0.000000	4.839809	2.558389	0.000000	4.824384	2.557082

H	-0.000000	1.373700	-2.398485	0.000000	1.360408	-2.399688
H	-0.000000	-1.373700	-2.398485	-0.000000	-1.360408	-2.399688
H	-0.000000	5.556685	-2.399523	0.000000	5.558245	-2.403330
H	-0.000000	8.304342	-2.399811	0.000000	8.290036	-2.395477
H	-0.000000	-4.839809	2.558389	-0.000000	-4.824384	2.557082
H	0.000000	-2.092400	2.559175	-0.000000	-2.100456	2.562990
H	0.000000	2.092400	2.559175	0.000000	2.100456	2.562990
H	-0.000000	-8.304342	-2.399811	-0.000000	-8.290036	-2.395477
H	-0.000000	-5.556685	-2.399523	-0.000000	-5.558245	-2.403330
H	0.000000	-11.774403	2.553261	-0.000000	-11.762496	2.549333
C	-0.000000	-12.771770	-0.247733	-0.000000	-12.755707	-0.248596
H	0.000000	-9.025455	2.557611	-0.000000	-9.021101	2.562331
H	0.000000	9.025455	2.557611	0.000000	9.021101	2.562331
H	0.000000	11.774403	2.553261	0.000000	11.762496	2.549333
H	-0.000000	12.493112	-2.404854	0.000000	12.482594	-2.408956
H	-0.000000	15.239620	-2.398426	0.000000	15.227436	-2.397204
H	-0.000000	15.905939	0.250805	0.000000	15.890705	0.252635
O	-0.000000	-13.861277	0.557302	-0.000000	-13.845895	0.557843
C	-0.000000	-14.951719	-0.244864	-0.000000	-14.936595	-0.243419
C	-0.000000	-14.580534	-1.547378	-0.000000	-14.566589	-1.547438
C	-0.000000	-13.153255	-1.554934	-0.000000	-13.141273	-1.557887
H	-0.000000	-12.493112	-2.404854	-0.000000	-12.482594	-2.408956
H	-0.000000	-15.239620	-2.398426	-0.000000	-15.227436	-2.397204
H	-0.000000	-15.905939	0.250805	-0.000000	-15.890705	0.252635

Part 6.

**2O/ BHLYP**	**Ground state**			**Excited state**		
C	-2.7942947	0.4306224	0.0000000	-2.7798253	0.4424734	0.0000000
C	-2.7795830	-0.9121528	0.0000000	-2.7559747	-0.9274615	0.0000000
C	-1.4078103	-1.2929655	0.0000000	-1.4268481	-1.3206805	0.0000000
C	-0.7020587	-0.1414932	0.0000000	-0.6719927	-0.1245134	0.0000000
C	0.7020587	0.1414932	0.0000000	0.6719927	0.1245134	0.0000000
C	1.4078103	1.2929655	0.0000000	1.4268481	1.3206805	0.0000000
C	2.7795830	0.9121528	0.0000000	2.7559747	0.9274615	0.0000000
C	2.7942947	-0.4306224	0.0000000	2.7798253	-0.4424734	0.0000000
O	-1.5380239	0.9152181	0.0000000	-1.5290121	0.9530923	0.0000000
O	1.5380239	-0.9152181	0.0000000	1.5290121	-0.9530923	0.0000000
H	-3.5788801	1.1541557	0.0000000	-3.5811571	1.1487363	0.0000000
H	-3.6326128	-1.5559461	0.0000000	-3.6197818	-1.5579935	0.0000000
H	-1.0003897	-2.2802706	0.0000000	-1.0187783	-2.3075548	0.0000000
H	1.0003897	2.2802706	0.0000000	1.0187783	2.3075548	0.0000000
H	3.6326128	1.5559461	0.0000000	3.6197818	1.5579935	0.0000000
H	3.5788801	-1.1541557	0.0000000	3.5811571	-1.1487363	0.0000000
**3O/ BHLYP**	**Ground state**			**Excited state**		
O	3.4496439	0.0000000	0.8248372	3.4466952	0.0000000	0.8428822
C	4.5343170	0.0000000	0.0271949	4.5182538	0.0000000	0.0266231

C	4.1674217	0.0000000	-1.2647366	4.1343364	0.0000000	-1.2733929
C	2.7442493	0.0000000	-1.2722085	2.7314694	0.0000000	-1.2833439
C	2.3650614	0.0000000	0.0248654	2.3405394	0.0000000	0.0477173
C	1.0872544	0.0000000	0.6669273	1.0964573	0.0000000	0.6493462
O	0.0000000	0.0000000	-0.1318675	0.0000000	0.0000000	-0.1684272
C	-1.0872544	0.0000000	0.6669273	-1.0964573	0.0000000	0.6493462
C	-0.7091209	0.0000000	1.9646642	-0.6852127	0.0000000	1.9906614
C	0.7091209	0.0000000	1.9646642	0.6852127	0.0000000	1.9906614
C	-2.3650614	0.0000000	0.0248654	-2.3405394	0.0000000	0.0477173
O	-3.4496439	0.0000000	0.8248372	-3.4466952	0.0000000	0.8428822
C	-4.5343170	0.0000000	0.0271949	-4.5182538	0.0000000	0.0266231
C	-4.1674217	0.0000000	-1.2647366	-4.1343364	0.0000000	-1.2733929
C	-2.7442493	0.0000000	-1.2722085	-2.7314694	0.0000000	-1.2833439
H	5.4814566	0.0000000	0.5191168	5.4750057	0.0000000	0.5008627
H	4.8214939	0.0000000	-2.1098931	4.7898656	0.0000000	-2.1181448
H	2.0926407	0.0000000	-2.1184091	2.0735142	0.0000000	-2.1248925
H	-2.0926407	0.0000000	-2.1184091	-2.0735142	0.0000000	-2.1248925
H	-5.4814566	0.0000000	0.5191168	-5.4750057	0.0000000	0.5008627
H	-4.8214939	0.0000000	-2.1098931	-4.7898656	0.0000000	-2.1181448
H	1.3638375	0.0000000	2.8085774	1.3451022	0.0000000	2.8308972
H	-1.3638375	0.0000000	2.8085774	-1.3451022	0.0000000	2.8308972
**4O/ BHLYP**	**Ground state**			**Excited state**		
O	-1.7576760	0.3395703	0.0000000	-1.7566989	0.3764238	0.0000000
C	-2.7780834	-0.5429051	0.0000000	-2.7793659	-0.5191027	0.0000000
C	-2.2981301	-1.8068782	0.0000000	-2.2742783	-1.8094487	0.0000000
C	-0.8849960	-1.6949061	0.0000000	-0.8993276	-1.7087752	0.0000000
C	-0.6100551	-0.3704653	0.0000000	-0.5958818	-0.3429749	0.0000000
C	0.6100551	0.3704653	0.0000000	0.5958818	0.3429749	0.0000000
O	1.7576760	-0.3395703	0.0000000	1.7566989	-0.3764238	0.0000000
C	2.7780834	0.5429051	0.0000000	2.7793659	0.5191027	0.0000000
C	2.2981301	1.8068782	0.0000000	2.2742783	1.8094487	0.0000000
C	0.8849960	1.6949061	0.0000000	0.8993276	1.7087752	0.0000000
C	4.1026874	0.0047273	0.0000000	4.0819560	0.0133846	0.0000000
O	5.1201657	0.8885591	0.0000000	5.1085106	0.9001436	0.0000000
C	6.2647911	0.1797559	0.0000000	6.2499347	0.1871450	0.0000000
C	6.0019251	-1.1373411	0.0000000	5.9850849	-1.1369034	0.0000000
C	4.5839790	-1.2581222	0.0000000	4.5802993	-1.2657862	0.0000000
H	-2.8840511	-2.6999234	0.0000000	-2.8669619	-2.6985877	0.0000000
H	-0.1662712	-2.4849512	0.0000000	-0.1795157	-2.4981548	0.0000000
H	4.0018980	-2.1535753	0.0000000	4.0000067	-2.1625682	0.0000000
H	7.1698065	0.7454536	0.0000000	7.1582973	0.7481844	0.0000000
H	6.7211945	-1.9277422	0.0000000	6.7095328	-1.9230991	0.0000000
H	0.1662712	2.4849512	0.0000000	0.1795157	2.4981548	0.0000000
H	2.8840511	2.6999234	0.0000000	2.8669619	2.6985877	0.0000000
C	-4.1026874	-0.0047273	0.0000000	-4.0819560	-0.0133846	0.0000000
O	-5.1201657	-0.8885591	0.0000000	-5.1085106	-0.9001436	0.0000000
C	-6.2647911	-0.1797559	0.0000000	-6.2499347	-0.1871450	0.0000000
C	-6.0019251	1.1373411	0.0000000	-5.9850849	1.1369034	0.0000000

C	-4.5839790	1.2581222	0.0000000	-4.5802993	1.2657862	0.0000000
H	-4.0018980	2.1535753	0.0000000	-4.0000067	2.1625682	0.0000000
H	-7.1698065	-0.7454536	0.0000000	-7.1582973	-0.7481844	0.0000000
H	-6.7211945	1.9277422	0.0000000	-6.7095328	1.9230991	0.0000000
**5O/ BHLYP**	**Ground state**			**Excited state**		
O	3.4501103	0.0000000	0.2606311	3.4427058	0.0000000	0.2893216
C	4.5374179	0.0000000	-0.5378611	4.5311625	0.0000000	-0.5168685
C	4.1595622	0.0000000	-1.8361109	4.1372962	0.0000000	-1.8340483
C	2.7421496	0.0000000	-1.8370042	2.7493163	0.0000000	-1.8429180
C	2.3625819	0.0000000	-0.5385522	2.3441799	0.0000000	-0.5149889
C	1.0876422	0.0000000	0.1027018	1.0961136	0.0000000	0.0790337
O	0.0000000	0.0000000	-0.6960879	0.0000000	0.0000000	-0.7306215
C	-1.0876422	0.0000000	0.1027018	-1.0961136	0.0000000	0.0790337
C	-0.7084249	0.0000000	1.4015022	-0.6879354	0.0000000	1.4118006
C	0.7084249	0.0000000	1.4015022	0.6879354	0.0000000	1.4118006
C	-2.3625819	0.0000000	-0.5385522	-2.3441799	0.0000000	-0.5149889
O	-3.4501103	0.0000000	0.2606311	-3.4427058	0.0000000	0.2893216
C	-4.5374179	0.0000000	-0.5378611	-4.5311625	0.0000000	-0.5168685
C	-4.1595622	0.0000000	-1.8361109	-4.1372962	0.0000000	-1.8340483
C	-2.7421496	0.0000000	-1.8370042	-2.7493163	0.0000000	-1.8429180
C	5.8150457	0.0000000	0.1038158	5.7971648	0.0000000	0.0983190
O	6.8994832	0.0000000	-0.6964355	6.8851250	0.0000000	-0.7067923
C	7.9841694	0.0000000	0.1010046	7.9702093	0.0000000	0.0888980
C	7.6175708	0.0000000	1.3930971	7.6066599	0.0000000	1.3865960
C	6.1945447	0.0000000	1.4009087	6.1918263	0.0000000	1.4051020
C	-5.8150457	0.0000000	0.1038158	-5.7971648	0.0000000	0.0983190
O	-6.8994832	0.0000000	-0.6964355	-6.8851250	0.0000000	-0.7067923
H	-4.8146938	0.0000000	-2.6796890	-4.7976454	0.0000000	-2.6741242
H	-2.0886708	0.0000000	-2.6818186	-2.0961302	0.0000000	-2.6882155
H	-1.3624154	0.0000000	2.2459096	-1.3451916	0.0000000	2.2540954
H	1.3624154	0.0000000	2.2459096	1.3451916	0.0000000	2.2540954
H	2.0886708	0.0000000	-2.6818186	2.0961302	0.0000000	-2.6882155
H	4.8146938	0.0000000	-2.6796890	4.7976454	0.0000000	-2.6741242
H	5.5432289	0.0000000	2.2473390	5.5449689	0.0000000	2.2550366
H	8.2718142	0.0000000	2.2381058	8.2673139	0.0000000	2.2269494
C	-7.9841694	0.0000000	0.1010046	-7.9702093	0.0000000	0.0888980
C	-7.6175708	0.0000000	1.3930971	-7.6066599	0.0000000	1.3865960
C	-6.1945447	0.0000000	1.4009087	-6.1918263	0.0000000	1.4051020
H	-5.5432289	0.0000000	2.2473390	-5.5449689	0.0000000	2.2550366
H	-8.9312543	0.0000000	-0.3910318	-8.9174474	0.0000000	-0.4034177
H	-8.2718142	0.0000000	2.2381058	-8.2673139	0.0000000	2.2269494
H	8.9312543	0.0000000	-0.3910318	8.9174474	0.0000000	-0.4034177
**6O/ BHLYP**	**Ground state**			**Excited state**		
O	1.7900256	-0.0195119	0.0000000	1.7953315	0.0123704	0.0000000
C	0.5232597	-0.4848937	0.0000000	0.5172030	-0.4575373	0.0000000
C	0.5269619	-1.8380375	0.0000000	0.5391745	-1.8489687	0.0000000

C	1.8879507	-2.2314050	0.0000000	1.8661582	-2.2284688	0.0000000
C	2.6129772	-1.0889134	0.0000000	2.6213318	-1.0667356	0.0000000
C	4.0158311	-0.8271436	0.0000000	3.9935453	-0.8372754	0.0000000
O	4.8384693	-1.8970513	0.0000000	4.8224826	-1.9132897	0.0000000
C	6.1047921	-1.4321414	0.0000000	6.0909795	-1.4457572	0.0000000
C	6.1025813	-0.0799912	0.0000000	6.0835008	-0.0779093	0.0000000
C	4.7412710	0.3147094	0.0000000	4.7455141	0.3187181	0.0000000
H	6.9663192	0.5483171	0.0000000	6.9511728	0.5455991	0.0000000
H	2.2817270	-3.2242070	0.0000000	2.2643764	-3.2199133	0.0000000
H	-0.3357291	-2.4677211	0.0000000	-0.3245087	-2.4777359	0.0000000
C	7.1538291	-2.4035233	0.0000000	7.1372938	-2.3973830	0.0000000
O	8.4178989	-1.9360515	0.0000000	8.4043970	-1.9269428	0.0000000
C	9.2383077	-3.0034483	0.0000000	9.2259340	-2.9926254	0.0000000
C	8.5271670	-4.1428336	0.0000000	8.5163718	-4.1365799	0.0000000
C	7.1580095	-3.7549836	0.0000000	7.1503631	-3.7578159	0.0000000
H	6.2971866	-4.3871569	0.0000000	6.2922375	-4.3937046	0.0000000
H	10.2848117	-2.7939229	0.0000000	10.2724763	-2.7823445	0.0000000
H	8.9208791	-5.1363423	0.0000000	8.9155528	-5.1281066	0.0000000
C	-0.5232597	0.4848937	0.0000000	-0.5172030	0.4575373	0.0000000
O	-1.7900256	0.0195119	0.0000000	-1.7953315	-0.0123704	0.0000000
C	-2.6129772	1.0889134	0.0000000	-2.6213318	1.0667356	0.0000000
C	-1.8879507	2.2314050	0.0000000	-1.8661582	2.2284688	0.0000000
C	-0.5269619	1.8380375	0.0000000	-0.5391745	1.8489687	0.0000000
H	-2.2817270	3.2242070	0.0000000	-2.2643764	3.2199133	0.0000000
H	0.3357291	2.4677211	0.0000000	0.3245087	2.4777359	0.0000000
H	4.3483104	1.3078518	0.0000000	4.3549890	1.3129801	0.0000000
C	-4.0158311	0.8271436	0.0000000	-3.9935453	0.8372754	0.0000000
C	-4.7412710	-0.3147094	0.0000000	-4.7455141	-0.3187181	0.0000000
C	-6.1025813	0.0799912	0.0000000	-6.0835008	0.0779093	0.0000000
C	-6.1047921	1.4321414	0.0000000	-6.0909795	1.4457572	0.0000000
O	-4.8384693	1.8970513	0.0000000	-4.8224826	1.9132897	0.0000000
H	-6.9663192	-0.5483171	0.0000000	-6.9511728	-0.5455991	0.0000000
H	-4.3483104	-1.3078518	0.0000000	-4.3549890	-1.3129801	0.0000000
C	-7.1538291	2.4035233	0.0000000	-7.1372938	2.3973830	0.0000000
C	-7.1580095	3.7549836	0.0000000	-7.1503631	3.7578159	0.0000000
C	-8.5271670	4.1428336	0.0000000	-8.5163718	4.1365799	0.0000000
C	-9.2383077	3.0034483	0.0000000	-9.2259340	2.9926254	0.0000000
O	-8.4178989	1.9360515	0.0000000	-8.4043970	1.9269428	0.0000000
H	-8.9208791	5.1363423	0.0000000	-8.9155528	5.1281066	0.0000000
H	-10.2848117	2.7939229	0.0000000	-10.272476	2.7823445	0.0000000
H	-6.2971866	4.3871569	0.0000000	-6.2922375	4.3937046	0.0000000
**7O/ BHLYP**	**Ground state**			**Excited state**		
C	-9.6444763	0.0000000	-1.4758579	-9.6337910	0.0000000	-1.4815556
C	-9.2648830	0.0000000	-0.1787996	-9.2470235	0.0000000	-0.1801939
O	-10.3492333	0.0000000	0.6215585	-10.333173	0.0000000	0.6222448
C	-11.4339593	0.0000000	-0.1757913	-11.417961	0.0000000	-0.1740543
C	-11.0674825	0.0000000	-1.4679284	-11.052955	0.0000000	-1.4687856
C	-7.9872270	0.0000000	0.4628095	-7.9754897	0.0000000	0.4495124

O	-6.8999633	0.0000000	-0.3356419	-6.8865123	0.0000000	-0.3492650
C	-5.8124521	0.0000000	0.4635903	-5.7939833	0.0000000	0.4538717
C	-6.1920607	0.0000000	1.7620398	-6.1915445	0.0000000	1.7669557
C	-7.6094233	0.0000000	1.7611187	-7.5927634	0.0000000	1.7579341
C	-4.5375689	0.0000000	-0.1776396	-4.5342606	0.0000000	-0.1556193
O	-3.4499053	0.0000000	0.6209486	-3.4427714	0.0000000	0.6487566
C	-2.3623387	0.0000000	-0.1781162	-2.3462835	0.0000000	-0.1553741
C	-2.7419178	0.0000000	-1.4769488	-2.7508570	0.0000000	-1.4808126
C	-4.1585815	0.0000000	-1.4766409	-4.1371033	0.0000000	-1.4752708
C	-1.0876171	0.0000000	0.4626838	-1.0954260	0.0000000	0.4401645
O	0.0000000	0.0000000	-0.3361932	0.0000000	0.0000000	-0.3661628
C	1.0876171	0.0000000	0.4626838	1.0954260	0.0000000	0.4401645
C	0.7082730	0.0000000	1.7616710	0.6897897	0.0000000	1.7682997
C	-0.7082730	0.0000000	1.7616710	-0.6897897	0.0000000	1.7682997
C	2.3623387	0.0000000	-0.1781162	2.3462835	0.0000000	-0.1553741
O	3.4499053	0.0000000	0.6209486	3.4427714	0.0000000	0.6487566
C	4.5375689	0.0000000	-0.1776396	4.5342606	0.0000000	-0.1556193
C	4.1585815	0.0000000	-1.4766409	4.1371033	0.0000000	-1.4752708
C	2.7419178	0.0000000	-1.4769488	2.7508570	0.0000000	-1.4808126
C	5.8124521	0.0000000	0.4635903	5.7939833	0.0000000	0.4538717
O	6.8999633	0.0000000	-0.3356419	6.8865123	0.0000000	-0.3492650
C	7.9872270	0.0000000	0.4628095	7.9754897	0.0000000	0.4495124
C	7.6094233	0.0000000	1.7611187	7.5927634	0.0000000	1.7579341
C	6.1920607	0.0000000	1.7620398	6.1915445	0.0000000	1.7669557
C	9.2648830	0.0000000	-0.1787996	9.2470235	0.0000000	-0.1801939
C	9.6444763	0.0000000	-1.4758579	9.6337910	0.0000000	-1.4815556
C	11.0674825	0.0000000	-1.4679284	11.0529558	0.0000000	-1.4687856
C	11.4339593	0.0000000	-0.1757913	11.4179613	0.0000000	-0.1740543
O	10.3492333	0.0000000	0.6215585	10.3331737	0.0000000	0.6222448
H	-1.3621725	0.0000000	2.6061479	-1.3460228	0.0000000	2.6113664
H	2.0882071	0.0000000	-2.3215811	2.0972763	0.0000000	-2.3258038
H	4.8127278	0.0000000	-2.3209162	4.7951075	0.0000000	-2.3170190
H	-2.0882071	0.0000000	-2.3215811	-2.0972763	0.0000000	-2.3258038
H	-4.8127278	0.0000000	-2.3209162	-4.7951075	0.0000000	-2.3170190
H	8.2645781	0.0000000	2.6046722	8.2527830	0.0000000	2.5980583
H	5.5386438	0.0000000	2.6069065	5.5410794	0.0000000	2.6142222
H	1.3621725	0.0000000	2.6061479	1.3460228	0.0000000	2.6113664
H	11.7217900	0.0000000	-2.3128831	11.7101642	0.0000000	-2.3116507
H	8.9932499	0.0000000	-2.3223595	8.9848444	0.0000000	-2.3298268
H	-5.5386438	0.0000000	2.6069065	-5.5410794	0.0000000	2.6142222
H	-8.2645781	0.0000000	2.6046722	-8.2527830	0.0000000	2.5980583
H	-8.9932499	0.0000000	-2.3223595	-8.9848444	0.0000000	-2.3298268
H	-11.7217900	0.0000000	-2.3128831	-11.710164	0.0000000	-2.3116507
H	-12.3809925	0.0000000	0.3163476	-12.365074	0.0000000	0.3182195
H	12.3809925	0.0000000	0.3163476	12.3650747	0.0000000	0.3182195
**8O/ BHLYP**	**Ground state**			**Excited state**		
O	-11.8119587	2.5478886	0.0000000	-11.795926	2.5422622	0.0000000

C	-10.5622291	3.0524403	0.0000000	-<br>10.5447425	3.0482398	0.0000000
C	-10.6062567	4.4031861	0.0000000	-10.593298	4.4031087	0.0000000
C	-11.9862438	4.7504918	0.0000000	-11.971599	4.7460874	0.0000000
C	-12.6634705	3.5906196	0.0000000	-12.647938	3.5839956	0.0000000
C	-9.4850399	2.1123756	0.0000000	-9.4695414	2.1175459	0.0000000
C	-9.4430919	0.7608536	0.0000000	-9.4263249	0.7581481	0.0000000
C	-8.0707866	0.4063595	0.0000000	-8.0670796	0.4005502	0.0000000
C	-7.3792362	1.5690425	0.0000000	-7.3597323	1.5700050	0.0000000
O	-8.2329669	2.6143180	0.0000000	-8.2163519	2.6189411	0.0000000
C	-5.9847029	1.8719672	0.0000000	-5.9823351	1.8526139	0.0000000
O	-5.1306309	0.8273278	0.0000000	-5.1259831	0.8042719	0.0000000
C	-3.8781204	1.3298751	0.0000000	-3.8656005	1.3090775	0.0000000
C	-3.9217342	2.6823313	0.0000000	-3.9251304	2.6872576	0.0000000
C	-5.2936760	3.0353943	0.0000000	-5.2749573	3.0282921	0.0000000
C	-2.8035432	0.3913733	0.0000000	-2.8003503	0.4136538	0.0000000
O	-1.5510566	0.8936584	0.0000000	-1.5404953	0.9205265	0.0000000
C	-0.6971036	-0.1512922	0.0000000	-0.6799039	-0.1321804	0.0000000
C	-1.3885345	-1.3145801	0.0000000	-1.4031443	-1.3158739	0.0000000
C	-2.7602573	-0.9612143	0.0000000	-2.7413536	-0.9686294	0.0000000
C	0.6971036	0.1512922	0.0000000	0.6799039	0.1321804	0.0000000
O	1.5510566	-0.8936584	0.0000000	1.5404953	-0.9205265	0.0000000
C	2.8035432	-0.3913733	0.0000000	2.8003503	-0.4136538	0.0000000
C	2.7602573	0.9612143	0.0000000	2.7413536	0.9686294	0.0000000
C	1.3885345	1.3145801	0.0000000	1.4031443	1.3158739	0.0000000
C	3.8781204	-1.3298751	0.0000000	3.8656005	-1.3090775	0.0000000
O	5.1306309	-0.8273278	0.0000000	5.1259831	-0.8042719	0.0000000
C	5.9847029	-1.8719672	0.0000000	5.9823351	-1.8526139	0.0000000
C	5.2936760	-3.0353943	0.0000000	5.2749573	-3.0282921	0.0000000
C	3.9217342	-2.6823313	0.0000000	3.9251304	-2.6872576	0.0000000
H	3.6041478	1.6158650	0.0000000	3.5875695	1.6208298	0.0000000
H	-0.9660192	-2.2955036	0.0000000	-0.9794888	-2.2965851	0.0000000
H	-3.6041478	-1.6158650	0.0000000	-3.5875695	-1.6208298	0.0000000
H	3.0780213	-3.3372315	0.0000000	3.0824361	-3.3437185	0.0000000
C	7.3792362	-1.5690425	0.0000000	7.3597323	-1.5700050	0.0000000
H	5.7165457	-4.0161518	0.0000000	5.7023657	-4.0074400	0.0000000
H	-5.7165457	4.0161518	0.0000000	-5.7023657	4.0074400	0.0000000
H	-3.0780213	3.3372315	0.0000000	-3.0824361	3.3437185	0.0000000
H	0.9660192	2.2955036	0.0000000	0.9794888	2.2965851	0.0000000
H	-10.2879749	0.1074160	0.0000000	-10.274172	0.1081995	0.0000000
H	-7.6488331	-0.5748187	0.0000000	-7.6480579	-0.5819542	0.0000000
H	-12.4090913	5.7319524	0.0000000	-12.397037	5.7265268	0.0000000
H	-13.7033421	3.3503271	0.0000000	-13.687817	3.3433029	0.0000000
H	-9.7644655	5.0604908	0.0000000	-9.7529596	5.0622823	0.0000000
O	8.2329669	-2.6143180	0.0000000	8.2163519	-2.6189411	0.0000000
C	9.4850399	-2.1123756	0.0000000	9.4695414	-2.1175459	0.0000000
C	9.4430919	-0.7608536	0.0000000	9.4263249	-0.7581481	0.0000000
C	8.0707866	-0.4063595	0.0000000	8.0670796	-0.4005502	0.0000000
H	7.6488331	0.5748187	0.0000000	7.6480579	0.5819542	0.0000000
H	10.2879749	-0.1074160	0.0000000	10.2741720	-0.1081995	0.0000000

C	10.5622291	-3.0524403	0.0000000	10.5447425	-3.0482398	0.0000000
O	11.8119587	-2.5478886	0.0000000	11.7959262	-2.5422622	0.0000000
C	12.6634705	-3.5906196	0.0000000	12.6479387	-3.5839956	0.0000000
C	11.9862438	-4.7504918	0.0000000	11.9715997	-4.7460874	0.0000000
C	10.6062567	-4.4031861	0.0000000	10.5932983	-4.4031087	0.0000000
H	9.7644655	-5.0604908	0.0000000	9.7529596	-5.0622823	0.0000000
H	12.4090913	-5.7319524	0.0000000	12.3970373	-5.7265268	0.0000000
H	13.7033421	-3.3503271	0.0000000	13.6878170	-3.3433029	0.0000000
**9O/ BHLYP**	**Ground state**			**Excited state**		
C	-13.0943270	0.0000000	-1.5120234	-13.079962	0.0000000	-1.5147050
C	-12.7146676	0.0000000	-0.2149875	-12.696394	0.0000000	-0.2156734
O	-13.7989667	0.0000000	0.5854345	-13.781423	0.0000000	0.5860203
C	-14.8837259	0.0000000	-0.2118542	-14.866350	0.0000000	-0.2104197
C	-14.5173256	0.0000000	-1.5040172	-14.501017	0.0000000	-1.5039789
C	-11.4369840	0.0000000	0.4265662	-11.421806	0.0000000	0.4198310
O	-10.3497571	0.0000000	-0.3719035	-10.333608	0.0000000	-0.3780421
C	-9.2622314	0.0000000	0.4273120	-9.2434510	0.0000000	0.4233852
C	-9.6418075	0.0000000	1.7257649	-9.6334183	0.0000000	1.7293469
C	-11.0591589	0.0000000	1.7248811	-11.042034	0.0000000	1.7235201
C	-7.9873756	0.0000000	-0.2139676	-7.9772151	0.0000000	-0.1998790
O	-6.8996863	0.0000000	0.5845326	-6.8871129	0.0000000	0.6000936
C	-5.8121793	0.0000000	-0.2146307	-5.7940656	0.0000000	-0.2023140
C	-6.1918510	0.0000000	-1.5134271	-6.1917792	0.0000000	-1.5172291
C	-7.6084938	0.0000000	-1.5130222	-7.5892622	0.0000000	-1.5110811
C	-4.5374399	0.0000000	0.4261079	-4.5353496	0.0000000	0.4059817
O	-3.4498616	0.0000000	-0.3727233	-3.4430006	0.0000000	-0.3972380
C	-2.3622525	0.0000000	0.4262047	-2.3477381	0.0000000	0.4064628
C	-2.7416478	0.0000000	1.7251859	-2.7510072	0.0000000	1.7296376
C	-4.1581423	0.0000000	1.7251577	-4.1379792	0.0000000	1.7255070
C	-1.0875944	0.0000000	-0.2145768	-1.0947896	0.0000000	-0.1916383
O	0.0000000	0.0000000	0.5842866	0.0000000	0.0000000	0.6130074
C	1.0875944	0.0000000	-0.2145768	1.0947896	0.0000000	-0.1916383
C	0.7082383	0.0000000	-1.5136096	0.6912419	0.0000000	-1.5167108
C	-0.7082383	0.0000000	-1.5136096	-0.6912419	0.0000000	-1.5167108
C	2.3622525	0.0000000	0.4262047	2.3477381	0.0000000	0.4064628
O	3.4498616	0.0000000	-0.3727233	3.4430006	0.0000000	-0.3972380
C	4.5374399	0.0000000	0.4261079	4.5353496	0.0000000	0.4059817
C	4.1581423	0.0000000	1.7251577	4.1379792	0.0000000	1.7255070
C	2.7416478	0.0000000	1.7251859	2.7510072	0.0000000	1.7296376
C	5.8121793	0.0000000	-0.2146307	5.7940656	0.0000000	-0.2023140
C	6.1918510	0.0000000	-1.5134271	6.1917792	0.0000000	-1.5172291
C	7.6084938	0.0000000	-1.5130222	7.5892622	0.0000000	-1.5110811
C	7.9873756	0.0000000	-0.2139676	7.9772151	0.0000000	-0.1998790
O	6.8996863	0.0000000	0.5845326	6.8871129	0.0000000	0.6000936
C	9.2622314	0.0000000	0.4273120	9.2434510	0.0000000	0.4233852
O	10.3497571	0.0000000	-0.3719035	10.3336087	0.0000000	-0.3780421
C	11.4369840	0.0000000	0.4265662	11.4218063	0.0000000	0.4198310
C	11.0591589	0.0000000	1.7248811	11.0420349	0.0000000	1.7235201

C	9.6418075	0.0000000	1.7257649	9.6334183	0.0000000	1.7293469
H	-4.8120656	0.0000000	2.5696092	-4.7951502	0.0000000	2.5678536
H	-1.3621108	0.0000000	-2.3581055	-1.3469584	0.0000000	-2.3601470
H	1.3621108	0.0000000	-2.3581055	1.3469584	0.0000000	-2.3601470
H	-5.5382248	0.0000000	-2.3581284	-5.5399283	0.0000000	-2.3634438
H	-8.2626976	0.0000000	-2.3572489	-8.2470331	0.0000000	-2.3528777
H	4.8120656	0.0000000	2.5696092	4.7951502	0.0000000	2.5678536
H	2.0878110	0.0000000	2.5697162	2.0973620	0.0000000	2.5745750
H	-2.0878110	0.0000000	2.5697162	-2.0973620	0.0000000	2.5745750
H	8.2626976	0.0000000	-2.3572489	8.2470331	0.0000000	-2.3528777
H	5.5382248	0.0000000	-2.3581284	5.5399283	0.0000000	-2.3634438
H	11.7142953	0.0000000	2.5684466	11.7000934	0.0000000	2.5650149
C	12.7146676	0.0000000	-0.2149875	12.6963945	0.0000000	-0.2156734
H	8.9883829	0.0000000	2.5706275	8.9821469	0.0000000	2.5759244
H	-8.9883829	0.0000000	2.5706275	-8.9821469	0.0000000	2.5759244
H	-11.7142953	0.0000000	2.5684466	-11.700093	0.0000000	2.5650149
H	-12.4431544	0.0000000	-2.3585673	-12.430190	0.0000000	-2.3623304
H	-15.1716786	0.0000000	-2.3489351	-15.157000	0.0000000	-2.3477126
H	-15.8307322	0.0000000	0.2803374	-15.813286	0.0000000	0.2820624
O	13.7989667	0.0000000	0.5854345	13.7814235	0.0000000	0.5860203
C	14.8837259	0.0000000	-0.2118542	14.8663505	0.0000000	-0.2104197
C	14.5173256	0.0000000	-1.5040172	14.5010179	0.0000000	-1.5039789
C	13.0943270	0.0000000	-1.5120234	13.0799625	0.0000000	-1.5147050
H	12.4431544	0.0000000	-2.3585673	12.4301908	0.0000000	-2.3623304
H	15.1716786	0.0000000	-2.3489351	15.1570007	0.0000000	-2.3477126
H	15.8307322	0.0000000	0.2803374	15.8132862	0.0000000	0.2820624

Part 7.

**2O/ CAM-B3LYP**	**Ground state**			**Excited state**		
C	-2.8069982	0.4321278	0.0000000	-2.7915557	0.4438111	0.0000000
C	-2.7890134	-0.9162819	0.0000000	-2.7650333	-0.9310852	0.0000000
C	-1.4151178	-1.2975944	0.0000000	-1.4329128	-1.3258813	0.0000000
C	-0.7042793	-0.1431766	0.0000000	-0.6737216	-0.1267289	0.0000000
C	0.7042793	0.1431766	0.0000000	0.6737216	0.1267289	0.0000000
C	1.4151178	1.2975944	0.0000000	1.4329128	1.3258813	0.0000000
C	2.7890134	0.9162819	0.0000000	2.7650333	0.9310852	0.0000000
C	2.8069982	-0.4321278	0.0000000	2.7915557	-0.4438111	0.0000000
O	-1.5434843	0.9216052	0.0000000	-1.5341415	0.9604119	0.0000000
O	1.5434843	-0.9216052	0.0000000	1.5341415	-0.9604119	0.0000000
H	-3.5953909	1.1620968	0.0000000	-3.5969815	1.1564078	0.0000000
H	-3.6467412	-1.5654675	0.0000000	-3.6340400	-1.5664761	0.0000000
H	-1.0056051	-2.2916376	0.0000000	-1.0227187	-2.3194262	0.0000000
H	1.0056051	2.2916376	0.0000000	1.0227187	2.3194262	0.0000000
H	3.6467412	1.5654675	0.0000000	3.6340400	1.5664761	0.0000000
H	3.5953909	-1.1620968	0.0000000	3.5969815	-1.1564078	0.0000000
**3O/**	**Ground state**			**Excited state**		

**CAM-B3LYP**						
O	3.4636806	0.0000000	0.8307793	3.4601217	0.0000000	0.8491542
C	4.5539626	0.0000000	0.0263544	4.5369712	0.0000000	0.0258074
C	4.1822288	0.0000000	-1.2700525	4.1486534	0.0000000	-1.2782988
C	2.7567961	0.0000000	-1.2771940	2.7427451	0.0000000	-1.2881158
C	2.3737816	0.0000000	0.0240387	2.3484524	0.0000000	0.0467134
C	1.0925440	0.0000000	0.6709158	1.1015879	0.0000000	0.6530121
O	0.0000000	0.0000000	-0.1347980	0.0000000	0.0000000	-0.1728940
C	-1.0925440	0.0000000	0.6709158	-1.1015879	0.0000000	0.6530121
C	-0.7105149	0.0000000	1.9726903	-0.6868370	0.0000000	1.9984862
C	0.7105149	0.0000000	1.9726903	0.6868370	0.0000000	1.9984862
C	-2.3737816	0.0000000	0.0240387	-2.3484524	0.0000000	0.0467134
O	-3.4636806	0.0000000	0.8307793	-3.4601217	0.0000000	0.8491542
C	-4.5539626	0.0000000	0.0263544	-4.5369712	0.0000000	0.0258074
C	-4.1822288	0.0000000	-1.2700525	-4.1486534	0.0000000	-1.2782988
C	-2.7567961	0.0000000	-1.2771940	-2.7427451	0.0000000	-1.2881158
H	5.5065255	0.0000000	0.5233731	5.4991990	0.0000000	0.5052139
H	4.8392695	0.0000000	-2.1217600	4.8072930	0.0000000	-2.1295297
H	2.1012901	0.0000000	-2.1292648	2.0807481	0.0000000	-2.1354002
H	-2.1012901	0.0000000	-2.1292648	-2.0807481	0.0000000	-2.1354002
H	-5.5065255	0.0000000	0.5233731	-5.4991990	0.0000000	0.5052139
H	-4.8392695	0.0000000	-2.1217600	-4.8072930	0.0000000	-2.1295297
H	1.3690805	0.0000000	2.8225211	1.3508887	0.0000000	2.8444066
H	-1.3690805	0.0000000	2.8225211	-1.3508887	0.0000000	2.8444066
**4O/ CAM-B3LYP**	**Ground state**			**Excited state**		
O	-1.7650332	0.3432868	0.0000000	-1.7638355	0.3812312	0.0000000
C	-2.7901351	-0.5465590	0.0000000	-2.7907832	-0.5223304	0.0000000
C	-2.3059544	-1.8141624	0.0000000	-2.2819645	-1.8162115	0.0000000
C	-0.8899219	-1.7018469	0.0000000	-0.9036273	-1.7156113	0.0000000
C	-0.6116205	-0.3731702	0.0000000	-0.5969494	-0.3454042	0.0000000
C	0.6116205	0.3731702	0.0000000	0.5969494	0.3454042	0.0000000
O	1.7650332	-0.3432868	0.0000000	1.7638355	-0.3812312	0.0000000
C	2.7901351	0.5465590	0.0000000	2.7907832	0.5223304	0.0000000
C	2.3059544	1.8141624	0.0000000	2.2819645	1.8162115	0.0000000
C	0.8899219	1.7018469	0.0000000	0.9036273	1.7156113	0.0000000
C	4.1185892	0.0038401	0.0000000	4.0970805	0.0121268	0.0000000
O	5.1407925	0.8948621	0.0000000	5.1282309	0.9064490	0.0000000
C	6.2915781	0.1797744	0.0000000	6.2757841	0.1871856	0.0000000
C	6.0242719	-1.1421629	0.0000000	6.0069279	-1.1413911	0.0000000
C	4.6040315	-1.2628246	0.0000000	4.5991837	-1.2703024	0.0000000
H	-2.8951840	-2.7134503	0.0000000	-2.8783753	-2.7113500	0.0000000
H	-0.1668670	-2.4974641	0.0000000	-0.1794073	-2.5104720	0.0000000
H	4.0185538	-2.1644540	0.0000000	4.0154749	-2.1732348	0.0000000
H	7.2015723	0.7510189	0.0000000	7.1890221	0.7539968	0.0000000
H	6.7470394	-1.9388427	0.0000000	6.7349733	-1.9338031	0.0000000
H	0.1668670	2.4974641	0.0000000	0.1794073	2.5104720	0.0000000

H	2.8951840	2.7134503	0.0000000	2.8783753	2.7113500	0.0000000
C	-4.1185892	-0.0038401	0.0000000	-4.0970805	-0.0121268	0.0000000
O	-5.1407925	-0.8948621	0.0000000	-5.1282309	-0.9064490	0.0000000
C	-6.2915781	-0.1797744	0.0000000	-6.2757841	-0.1871856	0.0000000
C	-6.0242719	1.1421629	0.0000000	-6.0069279	1.1413911	0.0000000
C	-4.6040315	1.2628246	0.0000000	-4.5991837	1.2703024	0.0000000
H	-4.0185538	2.1644540	0.0000000	-4.0154749	2.1732348	0.0000000
H	-7.2015723	-0.7510189	0.0000000	-7.1890221	-0.7539968	0.0000000
H	-6.7470394	1.9388427	0.0000000	-6.7349733	1.9338031	0.0000000
**5O/ CAM-B3LYP**	**Ground state**			**Excited state**		
O	3.4641773	0.0000000	0.2640775	3.4561443	0.0000000	0.2931210
C	4.5567745	0.0000000	-0.5413693	4.5495609	0.0000000	-0.5203194
C	4.1750216	0.0000000	-1.8435630	4.1524758	0.0000000	-1.8410925
C	2.7546642	0.0000000	-1.8443638	2.7608548	0.0000000	-1.8502766
C	2.3714198	0.0000000	-0.5419852	2.3522305	0.0000000	-0.5186129
C	1.0928780	0.0000000	0.1044464	1.1013192	0.0000000	0.0801626
O	0.0000000	0.0000000	-0.7012612	0.0000000	0.0000000	-0.7372372
C	-1.0928780	0.0000000	0.1044464	-1.1013192	0.0000000	0.0801626
C	-0.7099537	0.0000000	1.4071130	-0.6895763	0.0000000	1.4170177
C	0.7099537	0.0000000	1.4071130	0.6895763	0.0000000	1.4170177
C	-2.3714198	0.0000000	-0.5419852	-2.3522305	0.0000000	-0.5186129
O	-3.4641773	0.0000000	0.2640775	-3.4561443	0.0000000	0.2931210
C	-4.5567745	0.0000000	-0.5413693	-4.5495609	0.0000000	-0.5203194
C	-4.1750216	0.0000000	-1.8435630	-4.1524758	0.0000000	-1.8410925
C	-2.7546642	0.0000000	-1.8443638	-2.7608548	0.0000000	-1.8502766
C	5.8378721	0.0000000	0.1051798	5.8191376	0.0000000	0.1001060
O	6.9276026	0.0000000	-0.7018432	6.9122719	0.0000000	-0.7119114
C	8.0179204	0.0000000	0.1023732	8.0029537	0.0000000	0.0905896
C	7.6464839	0.0000000	1.3989265	7.6347769	0.0000000	1.3924724
C	6.2211751	0.0000000	1.4064098	6.2170651	0.0000000	1.4104633
C	-5.8378721	0.0000000	0.1051798	-5.8191376	0.0000000	0.1001060
O	-6.9276026	0.0000000	-0.7018432	-6.9122719	0.0000000	-0.7119114
H	-4.8339608	0.0000000	-2.6930916	-4.8169508	0.0000000	-2.6868960
H	-2.0973275	0.0000000	-2.6950888	-2.1038051	0.0000000	-2.7014604
H	-1.3677630	0.0000000	2.2574608	-1.3509017	0.0000000	2.2650669
H	1.3677630	0.0000000	2.2574608	1.3509017	0.0000000	2.2650669
H	2.0973275	0.0000000	-2.6950888	2.1038051	0.0000000	-2.7014604
H	4.8339608	0.0000000	-2.6930916	4.8169508	0.0000000	-2.6868960
H	5.5659676	0.0000000	2.2587152	5.5662079	0.0000000	2.2661820
H	8.3037000	0.0000000	2.2504846	8.2983409	0.0000000	2.2394441
C	-8.0179204	0.0000000	0.1023732	-8.0029537	0.0000000	0.0905896
C	-7.6464839	0.0000000	1.3989265	-7.6347769	0.0000000	1.3924724
C	-6.2211751	0.0000000	1.4064098	-6.2170651	0.0000000	1.4104633
H	-5.5659676	0.0000000	2.2587152	-5.5662079	0.0000000	2.2661820
H	-8.9704161	0.0000000	-0.3947829	-8.9555497	0.0000000	-0.4069694
H	-8.3037000	0.0000000	2.2504846	-8.2983409	0.0000000	2.2394441
H	8.9704161	0.0000000	-0.3947829	8.9555497	0.0000000	-0.4069694

**6O/ CAM-B3LYP**	**Ground state**			**Excited state**		
O	1.7979777	-0.0173306	0.0000000	1.8032517	0.0156501	0.0000000
C	0.5242841	-0.4879137	0.0000000	0.5179053	-0.4601501	0.0000000
C	0.5304537	-1.8457693	0.0000000	0.5423088	-1.8563907	0.0000000
C	1.8944106	-2.2399922	0.0000000	1.8727512	-2.2365162	0.0000000
C	2.6240532	-1.0948279	0.0000000	2.6320549	-1.0720108	0.0000000
C	4.0318150	-0.8290555	0.0000000	4.0088497	-0.8384016	0.0000000
O	4.8575682	-1.9070148	0.0000000	4.8408517	-1.9227881	0.0000000
C	6.1309057	-1.4368871	0.0000000	6.1161267	-1.4498317	0.0000000
C	6.1260359	-0.0798711	0.0000000	6.1065113	-0.0777634	0.0000000
C	4.7618610	0.3155524	0.0000000	4.7648570	0.3199930	0.0000000
H	6.9950818	0.5531005	0.0000000	6.9796156	0.5502536	0.0000000
H	2.2902090	-3.2395609	0.0000000	2.2734158	-3.2345720	0.0000000
H	-0.3375522	-2.4801040	0.0000000	-0.3267325	-2.4897385	0.0000000
C	7.1819227	-2.4139175	0.0000000	7.1644699	-2.4077410	0.0000000
O	8.4529585	-1.9414157	0.0000000	8.4384361	-1.9322460	0.0000000
C	9.2768944	-3.0168896	0.0000000	9.2634278	-3.0060773	0.0000000
C	8.5598629	-4.1592115	0.0000000	8.5480998	-4.1527293	0.0000000
C	7.1886030	-3.7704087	0.0000000	7.1795884	-3.7725717	0.0000000
H	6.3224078	-4.4071426	0.0000000	6.3159725	-4.4128556	0.0000000
H	10.3300185	-2.8039489	0.0000000	10.3165871	-2.7923444	0.0000000
H	8.9546092	-5.1598387	0.0000000	8.9481316	-5.1514575	0.0000000
C	-0.5242841	0.4879137	0.0000000	-0.5179053	0.4601501	0.0000000
O	-1.7979777	0.0173306	0.0000000	-1.8032517	-0.0156501	0.0000000
C	-2.6240532	1.0948279	0.0000000	-2.6320549	1.0720108	0.0000000
C	-1.8944106	2.2399922	0.0000000	-1.8727512	2.2365162	0.0000000
C	-0.5304537	1.8457693	0.0000000	-0.5423088	1.8563907	0.0000000
H	-2.2902090	3.2395609	0.0000000	-2.2734158	3.2345720	0.0000000
H	0.3375522	2.4801040	0.0000000	0.3267325	2.4897385	0.0000000
H	4.3668306	1.3154419	0.0000000	4.3722186	1.3209704	0.0000000
C	-4.0318150	0.8290555	0.0000000	-4.0088497	0.8384016	0.0000000
C	-4.7618610	-0.3155524	0.0000000	-4.7648570	-0.3199930	0.0000000
C	-6.1260359	0.0798711	0.0000000	-6.1065113	0.0777634	0.0000000
C	-6.1309057	1.4368871	0.0000000	-6.1161267	1.4498317	0.0000000
O	-4.8575682	1.9070148	0.0000000	-4.8408517	1.9227881	0.0000000
H	-6.9950818	-0.5531005	0.0000000	-6.9796156	-0.5502536	0.0000000
H	-4.3668306	-1.3154419	0.0000000	-4.3722186	-1.3209704	0.0000000
C	-7.1819227	2.4139175	0.0000000	-7.1644699	2.4077410	0.0000000
C	-7.1886030	3.7704087	0.0000000	-7.1795884	3.7725717	0.0000000
C	-8.5598629	4.1592115	0.0000000	-8.5480998	4.1527293	0.0000000
C	-9.2768944	3.0168896	0.0000000	-9.2634278	3.0060773	0.0000000
O	-8.4529585	1.9414157	0.0000000	-8.4384361	1.9322460	0.0000000
H	-8.9546092	5.1598387	0.0000000	-8.9481316	5.1514575	0.0000000
H	-10.3300185	2.8039489	0.0000000	-10.316587	2.7923444	0.0000000
H	-6.3224078	4.4071426	0.0000000	-6.3159725	4.4128556	0.0000000
**7O/**	**Ground state**			**Excited state**		

**CAM-B3LYP**						
C	-9.6852529	0.0000000	-1.4814586	-9.6729270	0.0000000	-1.4872265
C	-9.3018122	0.0000000	-0.1802721	-9.2830157	0.0000000	-0.1820960
O	-10.3914209	0.0000000	0.6268900	-10.374420	0.0000000	0.6269815
C	-11.4818060	0.0000000	-0.1771908	-11.464743	0.0000000	-0.1762729
C	-11.1105391	0.0000000	-1.4738039	-11.094790	0.0000000	-1.4752039
C	-8.0206541	0.0000000	0.4661430	-8.0077906	0.0000000	0.4532163
O	-6.9281386	0.0000000	-0.3393139	-6.9137053	0.0000000	-0.3525366
C	-5.8353670	0.0000000	0.4667299	-5.8160509	0.0000000	0.4577119
C	-6.2185739	0.0000000	1.7691291	-6.2167113	0.0000000	1.7740583
C	-7.6388819	0.0000000	1.7683726	-7.6218256	0.0000000	1.7650866
C	-4.5568992	0.0000000	-0.1797400	-4.5527113	0.0000000	-0.1574205
O	-3.4639718	0.0000000	0.6257387	-3.4562745	0.0000000	0.6542167
C	-2.3712117	0.0000000	-0.1802538	-2.3544734	0.0000000	-0.1573365
C	-2.7544797	0.0000000	-1.4829220	-2.7624681	0.0000000	-1.4864153
C	-4.1742339	0.0000000	-1.4825928	-4.1525217	0.0000000	-1.4805790
C	-1.0928382	0.0000000	0.4657889	-1.1006111	0.0000000	0.4431881
O	0.0000000	0.0000000	-0.3399953	0.0000000	0.0000000	-0.3707147
C	1.0928382	0.0000000	0.4657889	1.1006111	0.0000000	0.4431881
C	0.7098288	0.0000000	1.7686056	0.6915378	0.0000000	1.7752220
C	-0.7098288	0.0000000	1.7686056	-0.6915378	0.0000000	1.7752220
C	2.3712117	0.0000000	-0.1802538	2.3544734	0.0000000	-0.1573365
O	3.4639718	0.0000000	0.6257387	3.4562745	0.0000000	0.6542167
C	4.5568992	0.0000000	-0.1797400	4.5527113	0.0000000	-0.1574205
C	4.1742339	0.0000000	-1.4825928	4.1525217	0.0000000	-1.4805790
C	2.7544797	0.0000000	-1.4829220	2.7624681	0.0000000	-1.4864153
C	5.8353670	0.0000000	0.4667299	5.8160509	0.0000000	0.4577119
O	6.9281386	0.0000000	-0.3393139	6.9137053	0.0000000	-0.3525366
C	8.0206541	0.0000000	0.4661430	8.0077906	0.0000000	0.4532163
C	7.6388819	0.0000000	1.7683726	7.6218256	0.0000000	1.7650866
C	6.2185739	0.0000000	1.7691291	6.2167113	0.0000000	1.7740583
C	9.3018122	0.0000000	-0.1802721	9.2830157	0.0000000	-0.1820960
C	9.6852529	0.0000000	-1.4814586	9.6729270	0.0000000	-1.4872265
C	11.1105391	0.0000000	-1.4738039	11.0947904	0.0000000	-1.4752039
C	11.4818060	0.0000000	-0.1771908	11.4647431	0.0000000	-0.1762729
O	10.3914209	0.0000000	0.6268900	10.3744203	0.0000000	0.6269815
H	-1.3675678	0.0000000	2.6190056	-1.3517972	0.0000000	2.6240712
H	2.0969570	0.0000000	-2.3334979	2.1050317	0.0000000	-2.3373046
H	4.8322253	0.0000000	-2.3327880	4.8146208	0.0000000	-2.3280664
H	-2.0969570	0.0000000	-2.3334979	-2.1050317	0.0000000	-2.3373046
H	-4.8322253	0.0000000	-2.3327880	-4.8146208	0.0000000	-2.3280664
H	8.2978273	0.0000000	2.6178892	8.2857369	0.0000000	2.6111003
H	5.5612311	0.0000000	2.6198515	5.5623584	0.0000000	2.6271987
H	1.3675678	0.0000000	2.6190056	1.3517972	0.0000000	2.6240712
H	11.7678786	0.0000000	-2.3252614	11.7547438	0.0000000	-2.3248015
H	9.0301373	0.0000000	-2.3338351	9.0198638	0.0000000	-2.3411989
H	-5.5612311	0.0000000	2.6198515	-5.5623584	0.0000000	2.6271987
H	-8.2978273	0.0000000	2.6178892	-8.2857369	0.0000000	2.6111003
H	-9.0301373	0.0000000	-2.3338351	-9.0198638	0.0000000	-2.3411989

H	-11.7678786	0.0000000	-2.3252614	-11.754743	0.0000000	-2.3248015
H	-12.4342439	0.0000000	0.3200770	-12.417302	0.0000000	0.3210573
H	12.4342439	0.0000000	0.3200770	12.4173027	0.0000000	0.3210573
**8O/ CAM-B3LYP**	**Ground state**			**Excited state**		
O	-11.8615544	2.5559043	0.0000000	-11.843833	2.5500893	0.0000000
C	-10.6050749	3.0657362	0.0000000	-10.585905	3.0611773	0.0000000
C	-10.6518207	4.4213912	0.0000000	-10.636637	4.4205769	0.0000000
C	-12.0339530	4.7695426	0.0000000	-12.017302	4.7648877	0.0000000
C	-12.7168961	3.6065475	0.0000000	-12.699498	3.5999249	0.0000000
C	-9.5255691	2.1202237	0.0000000	-9.5084990	2.1242050	0.0000000
C	-9.4807359	0.7639580	0.0000000	-9.4628487	0.7606103	0.0000000
C	-8.1055154	0.4088764	0.0000000	-8.0998145	0.4022591	0.0000000
C	-7.4094584	1.5743944	0.0000000	-7.3887646	1.5741252	0.0000000
O	-8.2666161	2.6275998	0.0000000	-8.2485890	2.6310953	0.0000000
C	-6.0099660	1.8811938	0.0000000	-6.0061946	1.8615609	0.0000000
O	-5.1525371	0.8285396	0.0000000	-5.1467860	0.8051373	0.0000000
C	-3.8932182	1.3364353	0.0000000	-3.8795476	1.3157179	0.0000000
C	-3.9394100	2.6934744	0.0000000	-3.9413825	2.6979330	0.0000000
C	-5.3144039	3.0473554	0.0000000	-5.2951872	3.0397603	0.0000000
C	-2.8162262	0.3920022	0.0000000	-2.8123892	0.4142294	0.0000000
O	-1.5569174	0.8996541	0.0000000	-1.5458050	0.9270385	0.0000000
C	-0.6996087	-0.1532797	0.0000000	-0.6819813	-0.1342387	0.0000000
C	-1.3955054	-1.3193071	0.0000000	-1.4094730	-1.3207325	0.0000000
C	-2.7703325	-0.9651495	0.0000000	-2.7512189	-0.9723980	0.0000000
C	0.6996087	0.1532797	0.0000000	0.6819813	0.1342387	0.0000000
O	1.5569174	-0.8996541	0.0000000	1.5458050	-0.9270385	0.0000000
C	2.8162262	-0.3920022	0.0000000	2.8123892	-0.4142294	0.0000000
C	2.7703325	0.9651495	0.0000000	2.7512189	0.9723980	0.0000000
C	1.3955054	1.3193071	0.0000000	1.4094730	1.3207325	0.0000000
C	3.8932182	-1.3364353	0.0000000	3.8795476	-1.3157179	0.0000000
O	5.1525371	-0.8285396	0.0000000	5.1467860	-0.8051373	0.0000000
C	6.0099660	-1.8811938	0.0000000	6.0061946	-1.8615609	0.0000000
C	5.3144039	-3.0473554	0.0000000	5.2951872	-3.0397603	0.0000000
C	3.9394100	-2.6934744	0.0000000	3.9413825	-2.6979330	0.0000000
H	3.6196924	1.6242259	0.0000000	3.6028293	1.6291275	0.0000000
H	-0.9703449	-2.3067591	0.0000000	-0.9834330	-2.3080704	0.0000000
H	-3.6196924	-1.6242259	0.0000000	-3.6028293	-1.6291275	0.0000000
H	3.0902347	-3.3527987	0.0000000	3.0934990	-3.3591614	0.0000000
C	7.4094584	-1.5743944	0.0000000	7.3887646	-1.5741252	0.0000000
H	5.7398382	-4.0346733	0.0000000	5.7250895	-4.0254981	0.0000000
H	-5.7398382	4.0346733	0.0000000	-5.7250895	4.0254981	0.0000000
H	-3.0902347	3.3527987	0.0000000	-3.0934990	3.3591614	0.0000000
H	0.9703449	2.3067591	0.0000000	0.9834330	2.3080704	0.0000000
H	-10.3308522	0.1058191	0.0000000	-10.315853	0.1058343	0.0000000
H	-7.6809165	-0.5788126	0.0000000	-7.6784066	-0.5868685	0.0000000
H	-12.4581065	5.7580374	0.0000000	-12.443740	5.7525178	0.0000000
H	-13.7632914	3.3626675	0.0000000	-13.745914	3.3557111	0.0000000

H	-9.8046135	5.0831539	0.0000000	-9.7909047	5.0842611	0.0000000
O	8.2666161	-2.6275998	0.0000000	8.2485890	-2.6310953	0.0000000
C	9.5255691	-2.1202237	0.0000000	9.5084990	-2.1242050	0.0000000
C	9.4807359	-0.7639580	0.0000000	9.4628487	-0.7606103	0.0000000
C	8.1055154	-0.4088764	0.0000000	8.0998145	-0.4022591	0.0000000
H	7.6809165	0.5788126	0.0000000	7.6784066	0.5868685	0.0000000
H	10.3308522	-0.1058191	0.0000000	10.3158538	-0.1058343	0.0000000
C	10.6050749	-3.0657362	0.0000000	10.5859054	-3.0611773	0.0000000
O	11.8615544	-2.5559043	0.0000000	11.8438331	-2.5500893	0.0000000
C	12.7168961	-3.6065475	0.0000000	12.6994989	-3.5999249	0.0000000
C	12.0339530	-4.7695426	0.0000000	12.0173021	-4.7648877	0.0000000
C	10.6518207	-4.4213912	0.0000000	10.6366378	-4.4205769	0.0000000
H	9.8046135	-5.0831539	0.0000000	9.7909047	-5.0842611	0.0000000
H	12.4581065	-5.7580374	0.0000000	12.4437409	-5.7525178	0.0000000
H	13.7632914	-3.3626675	0.0000000	13.7459140	-3.3557111	0.0000000
**9O/ CAM-B3LYP**	**Ground state**			**Excited state**		
C	-13.1492556	0.0000000	-1.5175576	-13.133486	0.0000000	-1.5202581
C	-12.7657053	0.0000000	-0.2164060	-12.746509	0.0000000	-0.2173303
O	-13.8552356	0.0000000	0.5908563	-13.836756	0.0000000	0.5910701
C	-14.9456823	0.0000000	-0.2131271	-14.927304	0.0000000	-0.2122248
C	-14.5745348	0.0000000	-1.5097775	-14.557088	0.0000000	-1.5100870
C	-11.4844984	0.0000000	0.4299121	-11.468217	0.0000000	0.4235372
O	-10.3920442	0.0000000	-0.3756005	-10.374882	0.0000000	-0.3813969
C	-9.2992320	0.0000000	0.4303891	-9.2795987	0.0000000	0.4269043
C	-9.6823613	0.0000000	1.7328057	-9.6724961	0.0000000	1.7362118
C	-11.1026601	0.0000000	1.7321321	-11.084859	0.0000000	1.7307218
C	-8.0208137	0.0000000	-0.2161754	-8.0094919	0.0000000	-0.2024628
O	-6.9278293	0.0000000	0.5891806	-6.9143832	0.0000000	0.6045058
C	-5.8351584	0.0000000	-0.2169458	-5.8162190	0.0000000	-0.2050822
C	-6.2185588	0.0000000	-1.5195654	-6.2169886	0.0000000	-1.5231738
C	-7.6382955	0.0000000	-1.5190897	-7.6186185	0.0000000	-1.5169455
C	-4.5567447	0.0000000	0.4290005	-4.5538705	0.0000000	0.4090236
O	-3.4639654	0.0000000	-0.3767769	-3.4565556	0.0000000	-0.4014430
C	-2.3711163	0.0000000	0.4290191	-2.3560557	0.0000000	0.4096558
C	-2.7541201	0.0000000	1.7318394	-2.7626324	0.0000000	1.7363589
C	-4.1737372	0.0000000	1.7318571	-4.1535383	0.0000000	1.7319971
C	-1.0928172	0.0000000	-0.2170675	-1.0999335	0.0000000	-0.1937035
O	0.0000000	0.0000000	0.5887045	0.0000000	0.0000000	0.6184191
C	1.0928172	0.0000000	-0.2170675	1.0999335	0.0000000	-0.1937035
C	0.7098041	0.0000000	-1.5199160	0.6930949	0.0000000	-1.5224958
C	-0.7098041	0.0000000	-1.5199160	-0.6930949	0.0000000	-1.5224958
C	2.3711163	0.0000000	0.4290191	2.3560557	0.0000000	0.4096558
O	3.4639654	0.0000000	-0.3767769	3.4565556	0.0000000	-0.4014430
C	4.5567447	0.0000000	0.4290005	4.5538705	0.0000000	0.4090236
C	4.1737372	0.0000000	1.7318571	4.1535383	0.0000000	1.7319971
C	2.7541201	0.0000000	1.7318394	2.7626324	0.0000000	1.7363589
C	5.8351584	0.0000000	-0.2169458	5.8162190	0.0000000	-0.2050822

C	6.2185588	0.0000000	-1.5195654	6.2169886	0.0000000	-1.5231738
C	7.6382955	0.0000000	-1.5190897	7.6186185	0.0000000	-1.5169455
C	8.0208137	0.0000000	-0.2161754	8.0094919	0.0000000	-0.2024628
O	6.9278293	0.0000000	0.5891806	6.9143832	0.0000000	0.6045058
C	9.2992320	0.0000000	0.4303891	9.2795987	0.0000000	0.4269043
O	10.3920442	0.0000000	-0.3756005	10.3748820	0.0000000	-0.3813969
C	11.4844984	0.0000000	0.4299121	11.4682173	0.0000000	0.4235372
C	11.1026601	0.0000000	1.7321321	11.0848594	0.0000000	1.7307218
C	9.6823613	0.0000000	1.7328057	9.6724961	0.0000000	1.7362118
H	-4.8314718	0.0000000	2.5822542	-4.8147802	0.0000000	2.5800935
H	-1.3674977	0.0000000	-2.3703496	-1.3528207	0.0000000	-2.3717238
H	1.3674977	0.0000000	-2.3703496	1.3528207	0.0000000	-2.3717238
H	-5.5611531	0.0000000	-2.3702349	-5.5612998	0.0000000	-2.3753010
H	-8.2963727	0.0000000	-2.3692147	-8.2803224	0.0000000	-2.3645943
H	4.8314718	0.0000000	2.5822542	4.8147802	0.0000000	2.5800935
H	2.0964193	0.0000000	2.5822731	2.1051206	0.0000000	2.5871880
H	-2.0964193	0.0000000	2.5822731	-2.1051206	0.0000000	2.5871880
H	8.2963727	0.0000000	-2.3692147	8.2803224	0.0000000	-2.3645943
H	5.5611531	0.0000000	-2.3702349	5.5612998	0.0000000	-2.3753010
H	11.7615581	0.0000000	2.5816834	11.7466087	0.0000000	2.5782547
C	12.7657053	0.0000000	-0.2164060	12.7465096	0.0000000	-0.2173303
H	9.0249849	0.0000000	2.5835037	9.0172245	0.0000000	2.5885834
H	-9.0249849	0.0000000	2.5835037	-9.0172245	0.0000000	2.5885834
H	-11.7615581	0.0000000	2.5816834	-11.746608	0.0000000	2.5782547
H	-12.4942248	0.0000000	-2.3700000	-12.479716	0.0000000	-2.3736763
H	-15.2319488	0.0000000	-2.3611760	-15.215916	0.0000000	-2.3604733
H	-15.8980783	0.0000000	0.2842218	-15.879661	0.0000000	0.2853564
O	13.8552356	0.0000000	0.5908563	13.8367566	0.0000000	0.5910701
C	14.9456823	0.0000000	-0.2131271	14.9273046	0.0000000	-0.2122248
C	14.5745348	0.0000000	-1.5097775	14.5570881	0.0000000	-1.5100870
C	13.1492556	0.0000000	-1.5175576	13.1334868	0.0000000	-1.5202581
H	12.4942248	0.0000000	-2.3700000	12.4797166	0.0000000	-2.3736763
H	15.2319488	0.0000000	-2.3611760	15.2159160	0.0000000	-2.3604733
H	15.8980783	0.0000000	0.2842218	15.8796611	0.0000000	0.2853564

Part 8.

**2O/ tuned CAM-B3LYP**	**Ground state**			**Excited state**		
C	-2.8154702	0.4351089	0.0000000	-2.8019706	0.4462376	0.0000000
C	-2.7949175	-0.9216310	0.0000000	-2.7737648	-0.9352589	0.0000000
C	-1.4203835	-1.3041345	0.0000000	-1.4359322	-1.3314138	0.0000000
C	-0.7024517	-0.1437360	0.0000000	-0.6765628	-0.1298065	0.0000000
C	0.7024517	0.1437360	0.0000000	0.6765628	0.1298065	0.0000000
C	1.4203835	1.3041345	0.0000000	1.4359322	1.3314138	0.0000000
C	2.7949175	0.9216310	0.0000000	2.7737648	0.9352589	0.0000000
C	2.8154702	-0.4351089	0.0000000	2.8019706	-0.4462376	0.0000000
O	-1.5451798	0.9288480	0.0000000	-1.5366637	0.9685030	0.0000000

O	1.5451798	-0.9288480	0.0000000	1.5366637	-0.9685030	0.0000000
H	-3.6046137	1.1700248	0.0000000	-3.6080571	1.1641213	0.0000000
H	-3.6556240	-1.5737204	0.0000000	-3.6456327	-1.5737849	0.0000000
H	-1.0086863	-2.3019325	0.0000000	-1.0224588	-2.3282168	0.0000000
H	1.0086863	2.3019325	0.0000000	1.0224588	2.3282168	0.0000000
H	3.6556240	1.5737204	0.0000000	3.6456327	1.5737849	0.0000000
H	3.6046137	-1.1700248	0.0000000	3.6080571	-1.1641213	0.0000000
**3O/ tuned CAM-B3LYP**	**Ground state**			**Excited state**		
O	3.4707821	0.0000000	0.8380634	3.4715436	0.0000000	0.8545795
C	4.5662467	0.0000000	0.0274333	4.5497382	0.0000000	0.0186253
C	4.1895810	0.0000000	-1.2762584	4.1503801	0.0000000	-1.2901229
C	2.7632162	0.0000000	-1.2841166	2.7411388	0.0000000	-1.2928738
C	2.3752233	0.0000000	0.0248869	2.3519124	0.0000000	0.0494479
C	1.0978822	0.0000000	0.6718883	1.1054495	0.0000000	0.6651984
O	0.0000000	0.0000000	-0.1409397	0.0000000	0.0000000	-0.1700297
C	-1.0978822	0.0000000	0.6718883	-1.1054495	0.0000000	0.6651984
C	-0.7109240	0.0000000	1.9816205	-0.6910643	0.0000000	2.0132531
C	0.7109240	0.0000000	1.9816205	0.6910643	0.0000000	2.0132531
C	-2.3752233	0.0000000	0.0248869	-2.3519124	0.0000000	0.0494479
O	-3.4707821	0.0000000	0.8380634	-3.4715436	0.0000000	0.8545795
C	-4.5662467	0.0000000	0.0274333	-4.5497382	0.0000000	0.0186253
C	-4.1895810	0.0000000	-1.2762584	-4.1503801	0.0000000	-1.2901229
C	-2.7632162	0.0000000	-1.2841166	-2.7411388	0.0000000	-1.2928738
H	5.5209718	0.0000000	0.5287736	5.5165809	0.0000000	0.4976519
H	4.8485878	0.0000000	-2.1316616	4.8066139	0.0000000	-2.1484555
H	2.1044003	0.0000000	-2.1390823	2.0706056	0.0000000	-2.1389224
H	-2.1044003	0.0000000	-2.1390823	-2.0706056	0.0000000	-2.1389224
H	-5.5209718	0.0000000	0.5287736	-5.5165809	0.0000000	0.4976519
H	-4.8485878	0.0000000	-2.1316616	-4.8066139	0.0000000	-2.1484555
H	1.3733693	0.0000000	2.8339253	1.3589157	0.0000000	2.8616355
H	-1.3733693	0.0000000	2.8339253	-1.3589157	0.0000000	2.8616355
**4O/ tuned CAM-B3LYP**	**Ground state**			**Excited state**		
O	-1.7690821	0.3501555	0.0000000	-1.7690082	0.3843483	0.0000000
C	-2.7988295	-0.5473459	0.0000000	-2.7991614	-0.5294327	0.0000000
C	-2.3090440	-1.8224667	0.0000000	-2.2867738	-1.8271215	0.0000000
C	-0.8921920	-1.7100408	0.0000000	-0.9019073	-1.7234065	0.0000000
C	-0.6097027	-0.3729722	0.0000000	-0.5974191	-0.3497493	0.0000000
C	0.6097027	0.3729722	0.0000000	0.5974191	0.3497493	0.0000000
O	1.7690821	-0.3501555	0.0000000	1.7690082	-0.3843483	0.0000000
C	2.7988295	0.5473459	0.0000000	2.7991614	0.5294327	0.0000000
C	2.3090440	1.8224667	0.0000000	2.2867738	1.8271215	0.0000000
C	0.8921920	1.7100408	0.0000000	0.9019073	1.7234065	0.0000000
C	4.1234750	0.0043576	0.0000000	4.1045303	0.0146934	0.0000000

O	5.1506997	0.9023753	0.0000000	5.1416539	0.9148752	0.0000000
C	6.3072212	0.1816555	0.0000000	6.2935759	0.1868964	0.0000000
C	6.0357473	-1.1479737	0.0000000	6.0179270	-1.1485428	0.0000000
C	4.6146569	-1.2696028	0.0000000	4.6078727	-1.2752788	0.0000000
H	-2.9019287	-2.7245405	0.0000000	-2.8854866	-2.7259046	0.0000000
H	-0.1649935	-2.5077577	0.0000000	-0.1720729	-2.5189807	0.0000000
H	4.0262265	-2.1744561	0.0000000	4.0186937	-2.1797969	0.0000000
H	7.2189613	0.7574855	0.0000000	7.2098406	0.7563381	0.0000000
H	6.7608739	-1.9480859	0.0000000	6.7467014	-1.9459296	0.0000000
H	0.1649935	2.5077577	0.0000000	0.1720729	2.5189807	0.0000000
H	2.9019287	2.7245405	0.0000000	2.8854866	2.7259046	0.0000000
C	-4.1234750	-0.0043576	0.0000000	-4.1045303	-0.0146934	0.0000000
O	-5.1506997	-0.9023753	0.0000000	-5.1416539	-0.9148752	0.0000000
C	-6.3072212	-0.1816555	0.0000000	-6.2935759	-0.1868964	0.0000000
C	-6.0357473	1.1479737	0.0000000	-6.0179270	1.1485428	0.0000000
C	-4.6146569	1.2696028	0.0000000	-4.6078727	1.2752788	0.0000000
H	-4.0262265	2.1744561	0.0000000	-4.0186937	2.1797969	0.0000000
H	-7.2189613	-0.7574855	0.0000000	-7.2098406	-0.7563381	0.0000000
H	-6.7608739	1.9480859	0.0000000	-6.7467014	1.9459296	0.0000000
**5O/ tuned CAM-B3LYP**	**Ground state**			**Excited state**		
O	3.4710422	0.0000000	0.2707202	3.4654277	0.0000000	0.2988331
C	4.5689731	0.0000000	-0.5418495	4.5631949	0.0000000	-0.5241846
C	4.1824026	0.0000000	-1.8520395	4.1615705	0.0000000	-1.8505340
C	2.7612279	0.0000000	-1.8529812	2.7651473	0.0000000	-1.8573093
C	2.3729664	0.0000000	-0.5426147	2.3569592	0.0000000	-0.5204547
C	1.0981990	0.0000000	0.1035594	1.1052136	0.0000000	0.0849574
O	0.0000000	0.0000000	-0.7094230	0.0000000	0.0000000	-0.7411551
C	-1.0981990	0.0000000	0.1035594	-1.1052136	0.0000000	0.0849574
C	-0.7103693	0.0000000	1.4142401	-0.6932107	0.0000000	1.4251692
C	0.7103693	0.0000000	1.4142401	0.6932107	0.0000000	1.4251692
C	-2.3729664	0.0000000	-0.5426147	-2.3569592	0.0000000	-0.5204547
O	-3.4710422	0.0000000	0.2707202	-3.4654277	0.0000000	0.2988331
C	-4.5689731	0.0000000	-0.5418495	-4.5631949	0.0000000	-0.5241846
C	-4.1824026	0.0000000	-1.8520395	-4.1615705	0.0000000	-1.8505340
C	-2.7612279	0.0000000	-1.8529812	-2.7651473	0.0000000	-1.8573093
C	5.8461784	0.0000000	0.1048052	5.8305848	0.0000000	0.0982548
O	6.9415563	0.0000000	-0.7086790	6.9296368	0.0000000	-0.7200675
C	8.0370516	0.0000000	0.1017226	8.0248446	0.0000000	0.0899814
C	7.6607149	0.0000000	1.4055750	7.6508120	0.0000000	1.3988074
C	6.2344849	0.0000000	1.4138063	6.2315819	0.0000000	1.4163449
C	-5.8461784	0.0000000	0.1048052	-5.8305848	0.0000000	0.0982548
O	-6.9415563	0.0000000	-0.7086790	-6.9296368	0.0000000	-0.7200675
H	-4.8453235	0.0000000	-2.7039640	-4.8287759	0.0000000	-2.6996967
H	-2.1000405	0.0000000	-2.7062161	-2.1025502	0.0000000	-2.7096643
H	-1.3721298	0.0000000	2.2670215	-1.3586127	0.0000000	2.2755084
H	1.3721298	0.0000000	2.2670215	1.3586127	0.0000000	2.2755084

H	2.1000405	0.0000000	-2.7062161	2.1025502	0.0000000	-2.7096643
H	4.8453235	0.0000000	-2.7039640	4.8287759	0.0000000	-2.6996967
H	5.5759900	0.0000000	2.2690235	5.5763947	0.0000000	2.2741849
H	8.3199230	0.0000000	2.2608089	8.3157867	0.0000000	2.2499539
C	-8.0370516	0.0000000	0.1017226	-8.0248446	0.0000000	0.0899814
C	-7.6607149	0.0000000	1.4055750	-7.6508120	0.0000000	1.3988074
C	-6.2344849	0.0000000	1.4138063	-6.2315819	0.0000000	1.4163449
H	-5.5759900	0.0000000	2.2690235	-5.5763947	0.0000000	2.2741849
H	-8.9917087	0.0000000	-0.3997589	-8.9800930	0.0000000	-0.4110383
H	-8.3199230	0.0000000	2.2608089	-8.3157867	0.0000000	2.2499539
H	8.9917087	0.0000000	-0.3997589	8.9800930	0.0000000	-0.4110383
**6O/ tuned CAM-B3LYP**	**Ground state**			**Excited state**		
O	1.8033412	-0.0110325	0.0000000	1.8090104	0.0188916	0.0000000
C	0.5225420	-0.4872248	0.0000000	0.5175202	-0.4636064	0.0000000
C	0.5313049	-1.8541485	0.0000000	0.5405713	-1.8635146	0.0000000
C	1.8960825	-2.2484966	0.0000000	1.8767395	-2.2466586	0.0000000
C	2.6325786	-1.0969337	0.0000000	2.6393063	-1.0785852	0.0000000
C	4.0366243	-0.8302811	0.0000000	4.0169831	-0.8406356	0.0000000
O	4.8655232	-1.9166643	0.0000000	4.8515658	-1.9336660	0.0000000
C	6.1459333	-1.4411086	0.0000000	6.1338461	-1.4533313	0.0000000
C	6.1385895	-0.0750701	0.0000000	6.1213124	-0.0735283	0.0000000
C	4.7736520	0.3206446	0.0000000	4.7760240	0.3235495	0.0000000
H	7.0120944	0.5591404	0.0000000	6.9982843	0.5565473	0.0000000
H	2.2950824	-3.2514682	0.0000000	2.2799114	-3.2483626	0.0000000
H	-0.3411242	-2.4897900	0.0000000	-0.3335775	-2.4971854	0.0000000
C	7.1932387	-2.4170962	0.0000000	7.1792323	-2.4112185	0.0000000
O	8.4714639	-1.9398770	0.0000000	8.4605673	-1.9309531	0.0000000
C	9.2987189	-3.0226769	0.0000000	9.2883435	-3.0127339	0.0000000
C	8.5750251	-4.1706962	0.0000000	8.5659768	-4.1648581	0.0000000
C	7.2026503	-3.7824425	0.0000000	7.1962123	-3.7847324	0.0000000
H	6.3325290	-4.4211159	0.0000000	6.3282891	-4.4264478	0.0000000
H	10.3551087	-2.8061201	0.0000000	10.3449002	-2.7958906	0.0000000
H	8.9707289	-5.1753821	0.0000000	8.9667325	-5.1677711	0.0000000
C	-0.5225420	0.4872248	0.0000000	-0.5175202	0.4636064	0.0000000
O	-1.8033412	0.0110325	0.0000000	-1.8090104	-0.0188916	0.0000000
C	-2.6325786	1.0969337	0.0000000	-2.6393063	1.0785852	0.0000000
C	-1.8960825	2.2484966	0.0000000	-1.8767395	2.2466586	0.0000000
C	-0.5313049	1.8541485	0.0000000	-0.5405713	1.8635146	0.0000000
H	-2.2950824	3.2514682	0.0000000	-2.2799114	3.2483626	0.0000000
H	0.3411242	2.4897900	0.0000000	0.3335775	2.4971854	0.0000000
H	4.3755703	1.3239958	0.0000000	4.3788197	1.3273836	0.0000000
C	-4.0366243	0.8302811	0.0000000	-4.0169831	0.8406356	0.0000000
C	-4.7736520	-0.3206446	0.0000000	-4.7760240	-0.3235495	0.0000000
C	-6.1385895	0.0750701	0.0000000	-6.1213124	0.0735283	0.0000000
C	-6.1459333	1.4411086	0.0000000	-6.1338461	1.4533313	0.0000000
O	-4.8655232	1.9166643	0.0000000	-4.8515658	1.9336660	0.0000000

H	-7.0120944	-0.5591404	0.0000000	-6.9982843	-0.5565473	0.0000000
H	-4.3755703	-1.3239958	0.0000000	-4.3788197	-1.3273836	0.0000000
C	-7.1932387	2.4170962	0.0000000	-7.1792323	2.4112185	0.0000000
C	-7.2026503	3.7824425	0.0000000	-7.1962123	3.7847324	0.0000000
C	-8.5750251	4.1706962	0.0000000	-8.5659768	4.1648581	0.0000000
C	-9.2987189	3.0226769	0.0000000	-9.2883435	3.0127339	0.0000000
O	-8.4714639	1.9398770	0.0000000	-8.4605673	1.9309531	0.0000000
H	-8.9707289	5.1753821	0.0000000	-8.9667325	5.1677711	0.0000000
H	-10.3551087	2.8061201	0.0000000	-10.344900	2.7958906	0.0000000
H	-6.3325290	4.4211159	0.0000000	-6.3282891	4.4264478	0.0000000
**7O/ tuned CAM-B3LYP**	**Ground state**			**Excited state**		
C	-9.7053444	0.0000000	-1.4892442	-9.6966289	0.0000000	-1.4940902
C	-9.3169604	0.0000000	-0.1802740	-9.3018199	0.0000000	-0.1811149
O	-10.4122600	0.0000000	0.6333064	-10.398847	0.0000000	0.6348066
C	-11.5077836	0.0000000	-0.1770166	-11.494292	0.0000000	-0.1748571
C	-11.1315580	0.0000000	-1.4809079	-11.119503	0.0000000	-1.4811643
C	-8.0397441	0.0000000	0.4663467	-8.0298247	0.0000000	0.4542509
O	-6.9418382	0.0000000	-0.3461614	-6.9308930	0.0000000	-0.3601331
C	-5.8437997	0.0000000	0.4672511	-5.8280598	0.0000000	0.4576474
C	-6.2321223	0.0000000	1.7776012	-6.2313168	0.0000000	1.7812491
C	-7.6532560	0.0000000	1.7765989	-7.6386748	0.0000000	1.7733839
C	-4.5690830	0.0000000	-0.1789133	-4.5662175	0.0000000	-0.1601390
O	-3.4708546	0.0000000	0.6338640	-3.4652480	0.0000000	0.6604551
C	-2.3727520	0.0000000	-0.1794098	-2.3591619	0.0000000	-0.1591133
C	-2.7609170	0.0000000	-1.4900958	-2.7676267	0.0000000	-1.4931173
C	-4.1815041	0.0000000	-1.4897809	-4.1626734	0.0000000	-1.4888007
C	-1.0981638	0.0000000	0.4663893	-1.1046577	0.0000000	0.4472090
O	0.0000000	0.0000000	-0.3466884	0.0000000	0.0000000	-0.3751125
C	1.0981638	0.0000000	0.4663893	1.1046577	0.0000000	0.4472090
C	0.7102448	0.0000000	1.7772236	0.6948308	0.0000000	1.7831200
C	-0.7102448	0.0000000	1.7772236	-0.6948308	0.0000000	1.7831200
C	2.3727520	0.0000000	-0.1794098	2.3591619	0.0000000	-0.1591133
O	3.4708546	0.0000000	0.6338640	3.4652480	0.0000000	0.6604551
C	4.5690830	0.0000000	-0.1789133	4.5662175	0.0000000	-0.1601390
C	4.1815041	0.0000000	-1.4897809	4.1626734	0.0000000	-1.4888007
C	2.7609170	0.0000000	-1.4900958	2.7676267	0.0000000	-1.4931173
C	5.8437997	0.0000000	0.4672511	5.8280598	0.0000000	0.4576474
O	6.9418382	0.0000000	-0.3461614	6.9308930	0.0000000	-0.3601331
C	8.0397441	0.0000000	0.4663467	8.0298247	0.0000000	0.4542509
C	7.6532560	0.0000000	1.7765989	7.6386748	0.0000000	1.7733839
C	6.2321223	0.0000000	1.7776012	6.2313168	0.0000000	1.7812491
C	9.3169604	0.0000000	-0.1802740	9.3018199	0.0000000	-0.1811149
C	9.7053444	0.0000000	-1.4892442	9.6966289	0.0000000	-1.4940902
C	11.1315580	0.0000000	-1.4809079	11.1195034	0.0000000	-1.4811643
C	11.5077836	0.0000000	-0.1770166	11.4942927	0.0000000	-0.1748571
O	10.4122600	0.0000000	0.6333064	10.3988475	0.0000000	0.6348066

H	-1.3719010	0.0000000	2.6300851	-1.3591886	0.0000000	2.6342531
H	2.0994619	0.0000000	-2.3431214	2.1052072	0.0000000	-2.3456211
H	4.8434188	0.0000000	-2.3424322	4.8281046	0.0000000	-2.3391773
H	-2.0994619	0.0000000	-2.3431214	-2.1052072	0.0000000	-2.3456211
H	-4.8434188	0.0000000	-2.3424322	-4.8281046	0.0000000	-2.3391773
H	8.3162086	0.0000000	2.6284924	8.3060176	0.0000000	2.6222333
H	5.5710343	0.0000000	2.6309171	5.5720971	0.0000000	2.6361191
H	1.3719010	0.0000000	2.6300851	1.3591886	0.0000000	2.6342531
H	11.7908100	0.0000000	-2.3361035	11.7816005	0.0000000	-2.3343518
H	9.0469457	0.0000000	-2.3445377	9.0401981	0.0000000	-2.3509169
H	-5.5710343	0.0000000	2.6309171	-5.5720971	0.0000000	2.6361191
H	-8.3162086	0.0000000	2.6284924	-8.3060176	0.0000000	2.6222333
H	-9.0469457	0.0000000	-2.3445377	-9.0401981	0.0000000	-2.3509169
H	-11.7908100	0.0000000	-2.3361035	-11.781600	0.0000000	-2.3343518
H	-12.4623931	0.0000000	0.3245602	-12.449084	0.0000000	0.3267186
H	12.4623931	0.0000000	0.3245602	12.4490842	0.0000000	0.3267186
**8O/ tuned CAM-B3LYP**	**Ground state**			**Excited state**		
O	-11.8862147	2.5556973	0.0000000	-11.872693	2.5502642	0.0000000
C	-10.6226283	3.0702730	0.0000000	-10.607592	3.0665140	0.0000000
C	-10.6721424	4.4346926	0.0000000	-10.661286	4.4348334	0.0000000
C	-12.0553345	4.7824708	0.0000000	-12.043076	4.7784274	0.0000000
C	-12.7449218	3.6136878	0.0000000	-12.731770	3.6074896	0.0000000
C	-9.5470433	2.1255189	0.0000000	-9.5335873	2.1306002	0.0000000
C	-9.4996092	0.7603174	0.0000000	-9.4848876	0.7582708	0.0000000
C	-8.1236239	0.4048686	0.0000000	-8.1202331	0.4000554	0.0000000
C	-7.4207065	1.5768495	0.0000000	-7.4036988	1.5785094	0.0000000
O	-8.2811603	2.6384285	0.0000000	-8.2667538	2.6441569	0.0000000
C	-6.0249654	1.8844156	0.0000000	-6.0230805	1.8677197	0.0000000
O	-5.1642559	0.8233795	0.0000000	-5.1612507	0.8015427	0.0000000
C	-3.8979799	1.3369657	0.0000000	-3.8875584	1.3186255	0.0000000
C	-3.9468446	2.7029782	0.0000000	-3.9493401	2.7070328	0.0000000
C	-5.3226529	3.0571144	0.0000000	-5.3063710	3.0511181	0.0000000
C	-2.8246366	0.3937289	0.0000000	-2.8217687	0.4130135	0.0000000
O	-1.5584817	0.9073099	0.0000000	-1.5489009	0.9330677	0.0000000
C	-0.6976814	-0.1538294	0.0000000	-0.6831294	-0.1370822	0.0000000
C	-1.4000867	-1.3265340	0.0000000	-1.4120704	-1.3275136	0.0000000
C	-2.7758010	-0.9723993	0.0000000	-2.7598382	-0.9785274	0.0000000
C	0.6976814	0.1538294	0.0000000	0.6831294	0.1370822	0.0000000
O	1.5584817	-0.9073099	0.0000000	1.5489009	-0.9330677	0.0000000
C	2.8246366	-0.3937289	0.0000000	2.8217687	-0.4130135	0.0000000
C	2.7758010	0.9723993	0.0000000	2.7598382	0.9785274	0.0000000
C	1.4000867	1.3265340	0.0000000	1.4120704	1.3275136	0.0000000
C	3.8979799	-1.3369657	0.0000000	3.8875584	-1.3186255	0.0000000
O	5.1642559	-0.8233795	0.0000000	5.1612507	-0.8015427	0.0000000
C	6.0249654	-1.8844156	0.0000000	6.0230805	-1.8677197	0.0000000
C	5.3226529	-3.0571144	0.0000000	5.3063710	-3.0511181	0.0000000

C	3.9468446	-2.7029782	0.0000000	3.9493401	-2.7070328	0.0000000
H	3.6294559	1.6330046	0.0000000	3.6156494	1.6368833	0.0000000
H	-0.9715678	-2.3172518	0.0000000	-0.9821046	-2.3178656	0.0000000
H	-3.6294559	-1.6330046	0.0000000	-3.6156494	-1.6368833	0.0000000
H	3.0932151	-3.3636283	0.0000000	3.0962487	-3.3686232	0.0000000
C	7.4207065	-1.5768495	0.0000000	7.4036988	-1.5785094	0.0000000
H	5.7512577	-4.0477817	0.0000000	5.7388515	-4.0404628	0.0000000
H	-5.7512577	4.0477817	0.0000000	-5.7388515	4.0404628	0.0000000
H	-3.0932151	3.3636283	0.0000000	-3.0962487	3.3686232	0.0000000
H	0.9715678	2.3172518	0.0000000	0.9821046	2.3178656	0.0000000
H	-10.3542000	0.1008914	0.0000000	-10.342148	0.1018907	0.0000000
H	-7.6960294	-0.5862412	0.0000000	-7.6949696	-0.5921461	0.0000000
H	-12.4804415	5.7750477	0.0000000	-12.470723	5.7700519	0.0000000
H	-13.7944961	3.3661990	0.0000000	-13.781383	3.3596466	0.0000000
H	-9.8209998	5.0984068	0.0000000	-9.8116472	5.1005254	0.0000000
O	8.2811603	-2.6384285	0.0000000	8.2667538	-2.6441569	0.0000000
C	9.5470433	-2.1255189	0.0000000	9.5335873	-2.1306002	0.0000000
C	9.4996092	-0.7603174	0.0000000	9.4848876	-0.7582708	0.0000000
C	8.1236239	-0.4048686	0.0000000	8.1202331	-0.4000554	0.0000000
H	7.6960294	0.5862412	0.0000000	7.6949696	0.5921461	0.0000000
H	10.3542000	-0.1008914	0.0000000	10.3421484	-0.1018907	0.0000000
C	10.6226283	-3.0702730	0.0000000	10.6075928	-3.0665140	0.0000000
O	11.8862147	-2.5556973	0.0000000	11.8726933	-2.5502642	0.0000000
C	12.7449218	-3.6136878	0.0000000	12.7317700	-3.6074896	0.0000000
C	12.0553345	-4.7824708	0.0000000	12.0430768	-4.7784274	0.0000000
C	10.6721424	-4.4346926	0.0000000	10.6612864	-4.4348334	0.0000000
H	9.8209998	-5.0984068	0.0000000	9.8116472	-5.1005254	0.0000000
H	12.4804415	-5.7750477	0.0000000	12.4707239	-5.7700519	0.0000000
H	13.7944961	-3.3661990	0.0000000	13.7813835	-3.3596466	0.0000000
**9O/ tuned CAM-B3LYP**	**Ground state**			**Excited state**		
C	-13.1761832	0.0000000	-1.5255997	-13.164501	0.0000000	-1.5282725
C	-12.7877059	0.0000000	-0.2166575	-12.772385	0.0000000	-0.2174040
O	-13.8829278	0.0000000	0.5970090	-13.868346	0.0000000	0.5976870
C	-14.9785043	0.0000000	-0.2132261	-14.964018	0.0000000	-0.2118929
C	-14.6023860	0.0000000	-1.5171499	-14.588957	0.0000000	-1.5171610
C	-11.5104444	0.0000000	0.4298666	-11.497880	0.0000000	0.4232402
O	-10.4125884	0.0000000	-0.3826733	-10.399320	0.0000000	-0.3894667
C	-9.3145279	0.0000000	0.4307095	-9.2987277	0.0000000	0.4265390
C	-9.7028019	0.0000000	1.7410705	-9.6961147	0.0000000	1.7438624
C	-11.1239174	0.0000000	1.7401208	-11.109548	0.0000000	1.7384193
C	-8.0398472	0.0000000	-0.2155107	-8.0315081	0.0000000	-0.2029709
O	-6.9415788	0.0000000	0.5971589	-6.9314923	0.0000000	0.6127125
C	-5.8435545	0.0000000	-0.2162377	-5.8283400	0.0000000	-0.2046405
C	-6.2318374	0.0000000	-1.5268805	-6.2315649	0.0000000	-1.5296621
C	-7.6523970	0.0000000	-1.5264398	-7.6357781	0.0000000	-1.5245982
C	-4.5689422	0.0000000	0.4294825	-4.5673023	0.0000000	0.4123613

O	-3.4708129	0.0000000	-0.3835415	-3.4653618	0.0000000	-0.4066742
C	-2.3726697	0.0000000	0.4296000	-2.3606348	0.0000000	0.4124932
C	-2.7606439	0.0000000	1.7404228	-2.7681481	0.0000000	1.7440895
C	-4.1810809	0.0000000	1.7403806	-4.1640223	0.0000000	1.7408522
C	-1.0981359	0.0000000	-0.2161812	-1.1040765	0.0000000	-0.1962448
O	0.0000000	0.0000000	0.5968879	0.0000000	0.0000000	0.6240912
C	1.0981359	0.0000000	-0.2161812	1.1040765	0.0000000	-0.1962448
C	0.7102114	0.0000000	-1.5270562	0.6962035	0.0000000	-1.5292018
C	-0.7102114	0.0000000	-1.5270562	-0.6962035	0.0000000	-1.5292018
C	2.3726697	0.0000000	0.4296000	2.3606348	0.0000000	0.4124932
O	3.4708129	0.0000000	-0.3835415	3.4653618	0.0000000	-0.4066742
C	4.5689422	0.0000000	0.4294825	4.5673023	0.0000000	0.4123613
C	4.1810809	0.0000000	1.7403806	4.1640223	0.0000000	1.7408522
C	2.7606439	0.0000000	1.7404228	2.7681481	0.0000000	1.7440895
C	5.8435545	0.0000000	-0.2162377	5.8283400	0.0000000	-0.2046405
C	6.2318374	0.0000000	-1.5268805	6.2315649	0.0000000	-1.5296621
C	7.6523970	0.0000000	-1.5264398	7.6357781	0.0000000	-1.5245982
C	8.0398472	0.0000000	-0.2155107	8.0315081	0.0000000	-0.2029709
O	6.9415788	0.0000000	0.5971589	6.9314923	0.0000000	0.6127125
C	9.3145279	0.0000000	0.4307095	9.2987277	0.0000000	0.4265390
O	10.4125884	0.0000000	-0.3826733	10.3993200	0.0000000	-0.3894667
C	11.5104444	0.0000000	0.4298666	11.4978808	0.0000000	0.4232402
C	11.1239174	0.0000000	1.7401208	11.1095482	0.0000000	1.7384193
C	9.7028019	0.0000000	1.7410705	9.6961147	0.0000000	1.7438624
H	-4.8427771	0.0000000	2.5932052	-4.8288256	0.0000000	2.5916554
H	-1.3718527	0.0000000	-2.3799284	-1.3600183	0.0000000	-2.3807254
H	1.3718527	0.0000000	-2.3799284	1.3600183	0.0000000	-2.3807254
H	-5.5704771	0.0000000	-2.3799839	-5.5710171	0.0000000	-2.3835229
H	-8.3143995	0.0000000	-2.3790197	-8.3009741	0.0000000	-2.3750349
H	4.8427771	0.0000000	2.5932052	4.8288256	0.0000000	2.5916554
H	2.0990581	0.0000000	2.5933443	2.1059278	0.0000000	2.5967512
H	-2.0990581	0.0000000	2.5933443	-2.1059278	0.0000000	2.5967512
H	8.3143995	0.0000000	-2.3790197	8.3009741	0.0000000	-2.3750349
H	5.5704771	0.0000000	-2.3799839	5.5710171	0.0000000	-2.3835229
H	11.7868488	0.0000000	2.5920293	11.7752848	0.0000000	2.5883640
C	12.7877059	0.0000000	-0.2166575	12.7723857	0.0000000	-0.2174040
H	9.0416941	0.0000000	2.5943734	9.0366609	0.0000000	2.5984965
H	-9.0416941	0.0000000	2.5943734	-9.0366609	0.0000000	2.5984965
H	-11.7868488	0.0000000	2.5920293	-11.775284	0.0000000	2.5883640
H	-12.5178633	0.0000000	-2.3809546	-12.507468	0.0000000	-2.3846249
H	-15.2617026	0.0000000	-2.3722946	-15.249918	0.0000000	-2.3711366
H	-15.9330788	0.0000000	0.2884173	-15.918581	0.0000000	0.2899648
O	13.8829278	0.0000000	0.5970090	13.8683469	0.0000000	0.5976870
C	14.9785043	0.0000000	-0.2132261	14.9640180	0.0000000	-0.2118929
C	14.6023860	0.0000000	-1.5171499	14.5889577	0.0000000	-1.5171610
C	13.1761832	0.0000000	-1.5255997	13.1645010	0.0000000	-1.5282725
H	12.5178633	0.0000000	-2.3809546	12.5074685	0.0000000	-2.3846249
H	15.2617026	0.0000000	-2.3722946	15.2499189	0.0000000	-2.3711366
H	15.9330788	0.0000000	0.2884173	15.9185817	0.0000000	0.2899648

Part 9.

2O/ ωB97X	Ground state			Excited state		
C	-2.8067887	0.4318525	0.0000000	-2.7891546	0.4436938	0.0000000
C	-2.7922279	-0.9176872	0.0000000	-2.7662993	-0.9326693	0.0000000
C	-1.4153695	-1.3002742	0.0000000	-1.4330497	-1.3292765	0.0000000
C	-0.7077119	-0.1442062	0.0000000	-0.6743198	-0.1260607	0.0000000
C	0.7077119	0.1442062	0.0000000	0.6743198	0.1260607	0.0000000
C	1.4153695	1.3002742	0.0000000	1.4330497	1.3292765	0.0000000
C	2.7922279	0.9176872	0.0000000	2.7662993	0.9326693	0.0000000
C	2.8067887	-0.4318525	0.0000000	2.7891546	-0.4436938	0.0000000
O	-1.5435821	0.9213667	0.0000000	-1.5331691	0.9606076	0.0000000
O	1.5435821	-0.9213667	0.0000000	1.5331691	-0.9606076	0.0000000
H	-3.5952348	1.1644547	0.0000000	-3.5952566	1.1583279	0.0000000
H	-3.6523890	-1.5660668	0.0000000	-3.6382771	-1.5664786	0.0000000
H	-1.0029248	-2.2949731	0.0000000	-1.0201807	-2.3236109	0.0000000
H	1.0029248	2.2949731	0.0000000	1.0201807	2.3236109	0.0000000
H	3.6523890	1.5660668	0.0000000	3.6382771	1.5664786	0.0000000
H	3.5952348	-1.1644547	0.0000000	3.5952566	-1.1583279	0.0000000
**3O/ ωB97X**	**Ground state**			**Excited state**		
O	3.4645687	0.0000000	0.8302645	3.4585995	0.0000000	0.8493106
C	4.5540662	0.0000000	0.0252049	4.5351334	0.0000000	0.0268878
C	4.1845202	0.0000000	-1.2729024	4.1511706	0.0000000	-1.2792162
C	2.7557625	0.0000000	-1.2795006	2.7420465	0.0000000	-1.2904539
C	2.3773291	0.0000000	0.0225505	2.3499357	0.0000000	0.0453520
C	1.0901608	0.0000000	0.6743712	1.0995348	0.0000000	0.6513529
O	0.0000000	0.0000000	-0.1324390	0.0000000	0.0000000	-0.1754311
C	-1.0901608	0.0000000	0.6743712	-1.0995348	0.0000000	0.6513529
C	-0.7126512	0.0000000	1.9767866	-0.6866359	0.0000000	2.0013878
C	0.7126512	0.0000000	1.9767866	0.6866359	0.0000000	2.0013878
C	-2.3773291	0.0000000	0.0225505	-2.3499357	0.0000000	0.0453520
O	-3.4645687	0.0000000	0.8302645	-3.4585995	0.0000000	0.8493106
C	-4.5540662	0.0000000	0.0252049	-4.5351334	0.0000000	0.0268878
C	-4.1845202	0.0000000	-1.2729024	-4.1511706	0.0000000	-1.2792162
C	-2.7557625	0.0000000	-1.2795006	-2.7420465	0.0000000	-1.2904539
H	5.5076838	0.0000000	0.5241371	5.4978850	0.0000000	0.5093632
H	4.8435894	0.0000000	-2.1248789	4.8131297	0.0000000	-2.1296731
H	2.0965433	0.0000000	-2.1309087	2.0775186	0.0000000	-2.1380215
H	-2.0965433	0.0000000	-2.1309087	-2.0775186	0.0000000	-2.1380215
H	-5.5076838	0.0000000	0.5241371	-5.4978850	0.0000000	0.5093632
H	-4.8435894	0.0000000	-2.1248789	-4.8131297	0.0000000	-2.1296731
H	1.3746510	0.0000000	2.8260975	1.3546637	0.0000000	2.8464282
H	-1.3746510	0.0000000	2.8260975	-1.3546637	0.0000000	2.8464282
**4O/ ωB97X**	**Ground state**			**Excited state**		
O	-1.7655283	0.3407221	0.0000000	-1.7636007	0.3818177	0.0000000
C	-2.7878208	-0.5504095	0.0000000	-2.7871738	-0.5226352	0.0000000

C	-2.3076012	-1.8187453	0.0000000	-2.2817265	-1.8191500	0.0000000
C	-0.8871599	-1.7053706	0.0000000	-0.9021106	-1.7197088	0.0000000
C	-0.6142746	-0.3764964	0.0000000	-0.5976860	-0.3453885	0.0000000
C	0.6142746	0.3764964	0.0000000	0.5976860	0.3453885	0.0000000
O	1.7655283	-0.3407221	0.0000000	1.7636007	-0.3818177	0.0000000
C	2.7878208	0.5504095	0.0000000	2.7871738	0.5226352	0.0000000
C	2.3076012	1.8187453	0.0000000	2.2817265	1.8191500	0.0000000
C	0.8871599	1.7053706	0.0000000	0.9021106	1.7197088	0.0000000
C	4.1226649	0.0032169	0.0000000	4.0992338	0.0104816	0.0000000
O	5.1422492	0.8948110	0.0000000	5.1268963	0.9058097	0.0000000
C	6.2921887	0.1788240	0.0000000	6.2740870	0.1869940	0.0000000
C	6.0269736	-1.1445868	0.0000000	6.0090558	-1.1427988	0.0000000
C	4.6033408	-1.2646981	0.0000000	4.5973789	-1.2721826	0.0000000
H	-2.8998118	-2.7180985	0.0000000	-2.8825013	-2.7134875	0.0000000
H	-0.1603227	-2.4998628	0.0000000	-0.1746014	-2.5139391	0.0000000
H	4.0139566	-2.1658614	0.0000000	4.0105335	-2.1751930	0.0000000
H	7.2031517	0.7519484	0.0000000	7.1876168	0.7567824	0.0000000
H	6.7515926	-1.9415484	0.0000000	6.7395592	-1.9348673	0.0000000
H	0.1603227	2.4998628	0.0000000	0.1746014	2.5139391	0.0000000
H	2.8998118	2.7180985	0.0000000	2.8825013	2.7134875	0.0000000
C	-4.1226649	-0.0032169	0.0000000	-4.0992338	-0.0104816	0.0000000
O	-5.1422492	-0.8948110	0.0000000	-5.1268963	-0.9058097	0.0000000
C	-6.2921887	-0.1788240	0.0000000	-6.2740870	-0.1869940	0.0000000
C	-6.0269736	1.1445868	0.0000000	-6.0090558	1.1427988	0.0000000
C	-4.6033408	1.2646981	0.0000000	-4.5973789	1.2721826	0.0000000
H	-4.0139566	2.1658614	0.0000000	-4.0105335	2.1751930	0.0000000
H	-7.2031517	-0.7519484	0.0000000	-7.1876168	-0.7567824	0.0000000
H	-6.7515926	1.9415484	0.0000000	-6.7395592	1.9348673	0.0000000
**5O/ ωB97X**	**Ground state**			**Excited state**		
O	3.4658470	0.0000000	0.2636695	3.4560776	0.0000000	0.2936333
C	4.5550359	0.0000000	-0.5443049	4.5451848	0.0000000	-0.5220322
C	4.1761684	0.0000000	-1.8465506	4.1514517	0.0000000	-1.8431194
C	2.7513377	0.0000000	-1.8453776	2.7564717	0.0000000	-1.8515429
C	2.3746685	0.0000000	-0.5420941	2.3527727	0.0000000	-0.5172266
C	1.0903659	0.0000000	0.1108704	1.0993070	0.0000000	0.0834495
O	0.0000000	0.0000000	-0.6958360	0.0000000	0.0000000	-0.7350137
C	-1.0903659	0.0000000	0.1108704	-1.0993070	0.0000000	0.0834495
C	-0.7123166	0.0000000	1.4139017	-0.6898214	0.0000000	1.4243273
C	0.7123166	0.0000000	1.4139017	0.6898214	0.0000000	1.4243273
C	-2.3746685	0.0000000	-0.5420941	-2.3527727	0.0000000	-0.5172266
O	-3.4658470	0.0000000	0.2636695	-3.4560776	0.0000000	0.2936333
C	-4.5550359	0.0000000	-0.5443049	-4.5451848	0.0000000	-0.5220322
C	-4.1761684	0.0000000	-1.8465506	-4.1514517	0.0000000	-1.8431194
C	-2.7513377	0.0000000	-1.8453776	-2.7564717	0.0000000	-1.8515429
C	5.8430050	0.0000000	0.1055483	5.8221780	0.0000000	0.1012906
O	6.9289103	0.0000000	-0.7039695	6.9106569	0.0000000	-0.7133968
C	8.0195852	0.0000000	0.0993548	8.0021216	0.0000000	0.0873974
C	7.6521520	0.0000000	1.3981058	7.6390120	0.0000000	1.3911518

C	6.2234952	0.0000000	1.4070280	6.2171359	0.0000000	1.4107276
C	-5.8430050	0.0000000	0.1055483	-5.8221780	0.0000000	0.1012906
O	-6.9289103	0.0000000	-0.7039695	-6.9106569	0.0000000	-0.7133968
H	-4.8373569	0.0000000	-2.6964706	-4.8189240	0.0000000	-2.6887470
H	-2.0893330	0.0000000	-2.6946488	-2.0950906	0.0000000	-2.7015732
H	-1.3735495	0.0000000	2.2637617	-1.3549941	0.0000000	2.2716173
H	1.3735495	0.0000000	2.2637617	1.3549941	0.0000000	2.2716173
H	2.0893330	0.0000000	-2.6946488	2.0950906	0.0000000	-2.7015732
H	4.8373569	0.0000000	-2.6964706	4.8189240	0.0000000	-2.6887470
H	5.5657747	0.0000000	2.2595991	5.5640381	0.0000000	2.2669536
H	8.3125650	0.0000000	2.2490241	8.3059062	0.0000000	2.2372984
C	-8.0195852	0.0000000	0.0993548	-8.0021216	0.0000000	0.0873974
C	-7.6521520	0.0000000	1.3981058	-7.6390120	0.0000000	1.3911518
C	-6.2234952	0.0000000	1.4070280	-6.2171359	0.0000000	1.4107276
H	-5.5657747	0.0000000	2.2595991	-5.5640381	0.0000000	2.2669536
H	-8.9724281	0.0000000	-0.4010608	-8.9546256	0.0000000	-0.4142332
H	-8.3125650	0.0000000	2.2490241	-8.3059062	0.0000000	2.2372984
H	8.9724281	0.0000000	-0.4010608	8.9546256	0.0000000	-0.4142332
**6O/ ωB97X**	**Ground state**			**Excited state**		
O	1.7979234	-0.0194548	0.0000000	1.8030182	0.0167403	0.0000000
C	0.5263967	-0.4916734	0.0000000	0.5190486	-0.4607229	0.0000000
C	0.5276558	-1.8484881	0.0000000	0.5405730	-1.8597414	0.0000000
C	1.8961094	-2.2441167	0.0000000	1.8729790	-2.2390283	0.0000000
C	2.6212258	-1.0972898	0.0000000	2.6289617	-1.0709577	0.0000000
C	4.0364771	-0.8274488	0.0000000	4.0112251	-0.8351040	0.0000000
O	4.8598072	-1.9054214	0.0000000	4.8409846	-1.9192984	0.0000000
C	6.1310179	-1.4331832	0.0000000	6.1134478	-1.4447160	0.0000000
C	6.1302155	-0.0769019	0.0000000	6.1090366	-0.0741916	0.0000000
C	4.7615456	0.3191776	0.0000000	4.7627533	0.3251106	0.0000000
H	7.0020525	0.5551021	0.0000000	6.9853008	0.5523041	0.0000000
H	2.2952047	-3.2442148	0.0000000	2.2781617	-3.2371598	0.0000000
H	-0.3435045	-2.4813975	0.0000000	-0.3313863	-2.4920481	0.0000000
C	7.1864796	-2.4166652	0.0000000	7.1665716	-2.4103758	0.0000000
O	8.4551642	-1.9424448	0.0000000	8.4376811	-1.9334386	0.0000000
C	9.2782378	-3.0182674	0.0000000	9.2618261	-3.0075648	0.0000000
C	8.5628756	-4.1628585	0.0000000	8.5484115	-4.1560251	0.0000000
C	7.1885413	-3.7726394	0.0000000	7.1762553	-3.7734957	0.0000000
H	6.3190556	-4.4078320	0.0000000	6.3091299	-4.4119823	0.0000000
H	10.3328800	-2.8036403	0.0000000	10.3164473	-2.7919477	0.0000000
H	8.9594198	-5.1643363	0.0000000	8.9497511	-5.1557545	0.0000000
C	-0.5263967	0.4916734	0.0000000	-0.5190486	0.4607229	0.0000000
O	-1.7979234	0.0194548	0.0000000	-1.8030182	-0.0167403	0.0000000
C	-2.6212258	1.0972898	0.0000000	-2.6289617	1.0709577	0.0000000
C	-1.8961094	2.2441167	0.0000000	-1.8729790	2.2390283	0.0000000
C	-0.5276558	1.8484881	0.0000000	-0.5405730	1.8597414	0.0000000
H	-2.2952047	3.2442148	0.0000000	-2.2781617	3.2371598	0.0000000
H	0.3435045	2.4813975	0.0000000	0.3313863	2.4920481	0.0000000
H	4.3626997	1.3193998	0.0000000	4.3667904	1.3266450	0.0000000

C	-4.0364771	0.8274488	0.0000000	-4.0112251	0.8351040	0.0000000
C	-4.7615456	-0.3191776	0.0000000	-4.7627533	-0.3251106	0.0000000
C	-6.1302155	0.0769019	0.0000000	-6.1090366	0.0741916	0.0000000
C	-6.1310179	1.4331832	0.0000000	-6.1134478	1.4447160	0.0000000
O	-4.8598072	1.9054214	0.0000000	-4.8409846	1.9192984	0.0000000
H	-7.0020525	-0.5551021	0.0000000	-6.9853008	-0.5523041	0.0000000
H	-4.3626997	-1.3193998	0.0000000	-4.3667904	-1.3266450	0.0000000
C	-7.1864796	2.4166652	0.0000000	-7.1665716	2.4103758	0.0000000
C	-7.1885413	3.7726394	0.0000000	-7.1762553	3.7734957	0.0000000
C	-8.5628756	4.1628585	0.0000000	-8.5484115	4.1560251	0.0000000
C	-9.2782378	3.0182674	0.0000000	-9.2618261	3.0075648	0.0000000
O	-8.4551642	1.9424448	0.0000000	-8.4376811	1.9334386	0.0000000
H	-8.9594198	5.1643363	0.0000000	-8.9497511	5.1557545	0.0000000
H	-10.3328800	2.8036403	0.0000000	-10.316447	2.7919477	0.0000000
H	-6.3190556	4.4078320	0.0000000	-6.3091299	4.4119823	0.0000000
**7O/ ωB97X**	**Ground state**			**Excited state**		
C	-9.6917992	0.0000000	-1.4751685	-9.6752417	0.0000000	-1.4814416
C	-9.3086759	0.0000000	-0.1744690	-9.2873162	0.0000000	-0.1774348
O	-10.3929125	0.0000000	0.6372715	-10.373339	0.0000000	0.6353719
C	-11.4851965	0.0000000	-0.1638295	-11.465203	0.0000000	-0.1654204
C	-11.1204196	0.0000000	-1.4633384	-11.101135	0.0000000	-1.4666333
C	-8.0194159	0.0000000	0.4728265	-8.0034835	0.0000000	0.4607196
O	-6.9318057	0.0000000	-0.3372028	-6.9144815	0.0000000	-0.3488367
C	-5.8391216	0.0000000	0.4665168	-5.8173585	0.0000000	0.4590376
C	-6.2133093	0.0000000	1.7705090	-6.2087441	0.0000000	1.7760391
C	-7.6380977	0.0000000	1.7743866	-7.6195200	0.0000000	1.7711740
C	-4.5560519	0.0000000	-0.1888286	-4.5493345	0.0000000	-0.1645263
O	-3.4642679	0.0000000	0.6158148	-3.4547456	0.0000000	0.6459936
C	-2.3754004	0.0000000	-0.1930010	-2.3560860	0.0000000	-0.1681323
C	-2.7559050	0.0000000	-1.4953421	-2.7630321	0.0000000	-1.4985485
C	-4.1804530	0.0000000	-1.4926437	-4.1565725	0.0000000	-1.4892551
C	-1.0902259	0.0000000	0.4578160	-1.0983622	0.0000000	0.4332227
O	0.0000000	0.0000000	-0.3491275	0.0000000	0.0000000	-0.3819995
C	1.0902259	0.0000000	0.4578160	1.0983622	0.0000000	0.4332227
C	0.7121758	0.0000000	1.7609278	0.6921673	0.0000000	1.7685957
C	-0.7121758	0.0000000	1.7609278	-0.6921673	0.0000000	1.7685957
C	2.3754004	0.0000000	-0.1930010	2.3560860	0.0000000	-0.1681323
O	3.4642679	0.0000000	0.6158148	3.4547456	0.0000000	0.6459936
C	4.5560519	0.0000000	-0.1888286	4.5493345	0.0000000	-0.1645263
C	4.1804530	0.0000000	-1.4926437	4.1565725	0.0000000	-1.4892551
C	2.7559050	0.0000000	-1.4953421	2.7630321	0.0000000	-1.4985485
C	5.8391216	0.0000000	0.4665168	5.8173585	0.0000000	0.4590376
O	6.9318057	0.0000000	-0.3372028	6.9144815	0.0000000	-0.3488367
C	8.0194159	0.0000000	0.4728265	8.0034835	0.0000000	0.4607196
C	7.6380977	0.0000000	1.7743866	7.6195200	0.0000000	1.7711740
C	6.2133093	0.0000000	1.7705090	6.2087441	0.0000000	1.7760391
C	9.3086759	0.0000000	-0.1744690	9.2873162	0.0000000	-0.1774348
C	9.6917992	0.0000000	-1.4751685	9.6752417	0.0000000	-1.4814416

C	11.1204196	0.0000000	-1.4633384	11.1011357	0.0000000	-1.4666333
C	11.4851965	0.0000000	-0.1638295	11.4652030	0.0000000	-0.1654204
O	10.3929125	0.0000000	0.6372715	10.3733390	0.0000000	0.6353719
H	-1.3733789	0.0000000	2.6107997	-1.3563341	0.0000000	2.6166138
H	2.0963955	0.0000000	-2.3465448	2.1039201	0.0000000	-2.3503434
H	4.8432300	0.0000000	-2.3412848	4.8242129	0.0000000	-2.3345999
H	-2.0963955	0.0000000	-2.3465448	-2.1039201	0.0000000	-2.3503434
H	-4.8432300	0.0000000	-2.3412848	-4.8242129	0.0000000	-2.3345999
H	8.2976794	0.0000000	2.6255481	8.2844088	0.0000000	2.6185348
H	5.5497524	0.0000000	2.6185719	5.5485054	0.0000000	2.6268185
H	1.3733789	0.0000000	2.6107997	1.3563341	0.0000000	2.6166138
H	11.7825626	0.0000000	-2.3129028	11.7651008	0.0000000	-2.3149046
H	9.0358243	0.0000000	-2.3290888	9.0206177	0.0000000	-2.3364235
H	-5.5497524	0.0000000	2.6185719	-5.5485054	0.0000000	2.6268185
H	-8.2976794	0.0000000	2.6255481	-8.2844088	0.0000000	2.6185348
H	-9.0358243	0.0000000	-2.3290888	-9.0206177	0.0000000	-2.3364235
H	-11.7825626	0.0000000	-2.3129028	-11.765100	0.0000000	-2.3149046
H	-12.4370265	0.0000000	0.3385129	-12.417300	0.0000000	0.3366719
H	12.4370265	0.0000000	0.3385129	12.4173004	0.0000000	0.3366719
**8O/ ωB97X**	**Ground state**			**Excited state**		
O	-11.8641856	2.5573716	0.0000000	-11.844186	2.5508014	0.0000000
C	-10.6099093	3.0683089	0.0000000	-10.588966	3.0631243	0.0000000
C	-10.6513892	4.4235823	0.0000000	-10.634079	4.4212927	0.0000000
C	-12.0364516	4.7736841	0.0000000	-12.018209	4.7679071	0.0000000
C	-12.7181737	3.6087781	0.0000000	-12.698827	3.6012117	0.0000000
C	-9.5261669	2.1161388	0.0000000	-9.5066372	2.1182401	0.0000000
C	-9.4856623	0.7605004	0.0000000	-9.4655140	0.7564205	0.0000000
C	-8.1059756	0.4046892	0.0000000	-8.0964141	0.3977701	0.0000000
C	-7.4148163	1.5719908	0.0000000	-7.3920688	1.5708011	0.0000000
O	-8.2693530	2.6253629	0.0000000	-8.2493832	2.6272191	0.0000000
C	-6.0080510	1.8832403	0.0000000	-6.0013275	1.8639943	0.0000000
O	-5.1531965	0.8303838	0.0000000	-5.1448943	0.8080925	0.0000000
C	-3.8962705	1.3401519	0.0000000	-3.8798781	1.3203186	0.0000000
C	-3.9377506	2.6962617	0.0000000	-3.9376230	2.7019561	0.0000000
C	-5.3173596	3.0510846	0.0000000	-5.2965196	3.0438551	0.0000000
C	-2.8148259	0.3884644	0.0000000	-2.8092461	0.4138160	0.0000000
O	-1.5578300	0.8977986	0.0000000	-1.5449437	0.9279526	0.0000000
C	-0.7033144	-0.1554557	0.0000000	-0.6836822	-0.1343275	0.0000000
C	-1.3944340	-1.3230446	0.0000000	-1.4096767	-1.3241513	0.0000000
C	-2.7738421	-0.9677397	0.0000000	-2.7531411	-0.9739596	0.0000000
C	0.7033144	0.1554557	0.0000000	0.6836822	0.1343275	0.0000000
O	1.5578300	-0.8977986	0.0000000	1.5449437	-0.9279526	0.0000000
C	2.8148259	-0.3884644	0.0000000	2.8092461	-0.4138160	0.0000000
C	2.7738421	0.9677397	0.0000000	2.7531411	0.9739596	0.0000000
C	1.3944340	1.3230446	0.0000000	1.4096767	1.3241513	0.0000000
C	3.8962705	-1.3401519	0.0000000	3.8798781	-1.3203186	0.0000000
O	5.1531965	-0.8303838	0.0000000	5.1448943	-0.8080925	0.0000000
C	6.0080510	-1.8832403	0.0000000	6.0013275	-1.8639943	0.0000000

C	5.3173596	-3.0510846	0.0000000	5.2965196	-3.0438551	0.0000000
C	3.9377506	-2.6962617	0.0000000	3.9376230	-2.7019561	0.0000000
H	3.6263283	1.6255317	0.0000000	3.6087248	1.6283783	0.0000000
H	-0.9659017	-2.3108772	0.0000000	-0.9806598	-2.3120940	0.0000000
H	-3.6263283	-1.6255317	0.0000000	-3.6087248	-1.6283783	0.0000000
H	3.0855202	-3.3544100	0.0000000	3.0870188	-3.3625000	0.0000000
C	7.4148163	-1.5719908	0.0000000	7.3920688	-1.5708011	0.0000000
H	5.7464207	-4.0386744	0.0000000	5.7306545	-4.0296017	0.0000000
H	-5.7464207	4.0386744	0.0000000	-5.7306545	4.0296017	0.0000000
H	-3.0855202	3.3544100	0.0000000	-3.0870188	3.3625000	0.0000000
H	0.9659017	2.3108772	0.0000000	0.9806598	2.3120940	0.0000000
H	-10.3387590	0.1034813	0.0000000	-10.321052	0.1022268	0.0000000
H	-7.6776925	-0.5832707	0.0000000	-7.6709378	-0.5914885	0.0000000
H	-12.4620419	5.7631494	0.0000000	-12.445903	5.7565514	0.0000000
H	-13.7661278	3.3635891	0.0000000	-13.746710	3.3553310	0.0000000
H	-9.8004751	5.0834167	0.0000000	-9.7846767	5.0831198	0.0000000
O	8.2693530	-2.6253629	0.0000000	8.2493832	-2.6272191	0.0000000
C	9.5261669	-2.1161388	0.0000000	9.5066372	-2.1182401	0.0000000
C	9.4856623	-0.7605004	0.0000000	9.4655140	-0.7564205	0.0000000
C	8.1059756	-0.4046892	0.0000000	8.0964141	-0.3977701	0.0000000
H	7.6776925	0.5832707	0.0000000	7.6709378	0.5914885	0.0000000
H	10.3387590	-0.1034813	0.0000000	10.3210523	-0.1022268	0.0000000
C	10.6099093	-3.0683089	0.0000000	10.5889668	-3.0631243	0.0000000
O	11.8641856	-2.5573716	0.0000000	11.8441864	-2.5508014	0.0000000
C	12.7181737	-3.6087781	0.0000000	12.6988278	-3.6012117	0.0000000
C	12.0364516	-4.7736841	0.0000000	12.0182098	-4.7679071	0.0000000
C	10.6513892	-4.4235823	0.0000000	10.6340798	-4.4212927	0.0000000
H	9.8004751	-5.0834167	0.0000000	9.7846767	-5.0831198	0.0000000
H	12.4620419	-5.7631494	0.0000000	12.4459032	-5.7565514	0.0000000
H	13.7661278	-3.3635891	0.0000000	13.7467107	-3.3553310	0.0000000
**9O/ ωB97X**	**Ground state**			**Excited state**		
C	-13.1605404	0.0000000	-1.4978356	-13.141343	0.0000000	-1.4997772
C	-12.7743733	0.0000000	-0.1980396	-12.752673	0.0000000	-0.1986794
O	-13.8566988	0.0000000	0.6162403	-13.835554	0.0000000	0.6163715
C	-14.9508534	0.0000000	-0.1822932	-14.929731	0.0000000	-0.1816699
C	-14.5891275	0.0000000	-1.4826572	-14.568701	0.0000000	-1.4829010
C	-11.4836024	0.0000000	0.4462536	-11.464229	0.0000000	0.4414297
O	-10.3978736	0.0000000	-0.3662754	-10.377904	0.0000000	-0.3704761
C	-9.3033425	0.0000000	0.4349292	-9.2812602	0.0000000	0.4325902
C	-9.6745209	0.0000000	1.7397775	-9.6607141	0.0000000	1.7432184
C	-11.0992884	0.0000000	1.7469408	-11.078976	0.0000000	1.7461539
C	-8.0217898	0.0000000	-0.2233778	-8.0071106	0.0000000	-0.2105005
O	-6.9281730	0.0000000	0.5787311	-6.9118605	0.0000000	0.5926025
C	-5.8411773	0.0000000	-0.2326044	-5.8196058	0.0000000	-0.2220191
C	-6.2246757	0.0000000	-1.5340553	-6.2202140	0.0000000	-1.5380903
C	-7.6492025	0.0000000	-1.5280741	-7.6277745	0.0000000	-1.5259170
C	-4.5545727	0.0000000	0.4153835	-4.5494411	0.0000000	0.3939477
O	-3.4660479	0.0000000	-0.3937866	-3.4569584	0.0000000	-0.4197079

C	-2.3741880	0.0000000	0.4109529	-2.3568834	0.0000000	0.3902573
C	-2.7495648	0.0000000	1.7148317	-2.7573121	0.0000000	1.7196892
C	-4.1738650	0.0000000	1.7177546	-4.1517024	0.0000000	1.7170711
C	-1.0902947	0.0000000	-0.2423497	-1.0977063	0.0000000	-0.2167820
O	0.0000000	0.0000000	0.5643868	0.0000000	0.0000000	0.5965435
C	1.0902947	0.0000000	-0.2423497	1.0977063	0.0000000	-0.2167820
C	0.7122359	0.0000000	-1.5454824	0.6940329	0.0000000	-1.5484975
C	-0.7122359	0.0000000	-1.5454824	-0.6940329	0.0000000	-1.5484975
C	2.3741880	0.0000000	0.4109529	2.3568834	0.0000000	0.3902573
O	3.4660479	0.0000000	-0.3937866	3.4569584	0.0000000	-0.4197079
C	4.5545727	0.0000000	0.4153835	4.5494411	0.0000000	0.3939477
C	4.1738650	0.0000000	1.7177546	4.1517024	0.0000000	1.7170711
C	2.7495648	0.0000000	1.7148317	2.7573121	0.0000000	1.7196892
C	5.8411773	0.0000000	-0.2326044	5.8196058	0.0000000	-0.2220191
C	6.2246757	0.0000000	-1.5340553	6.2202140	0.0000000	-1.5380903
C	7.6492025	0.0000000	-1.5280741	7.6277745	0.0000000	-1.5259170
C	8.0217898	0.0000000	-0.2233778	8.0071106	0.0000000	-0.2105005
O	6.9281730	0.0000000	0.5787311	6.9118605	0.0000000	0.5926025
C	9.3033425	0.0000000	0.4349292	9.2812602	0.0000000	0.4325902
O	10.3978736	0.0000000	-0.3662754	10.3779049	0.0000000	-0.3704761
C	11.4836024	0.0000000	0.4462536	11.4642296	0.0000000	0.4414297
C	11.0992884	0.0000000	1.7469408	11.0789765	0.0000000	1.7461539
C	9.6745209	0.0000000	1.7397775	9.6607141	0.0000000	1.7432184
H	-4.8333337	0.0000000	2.5689661	-4.8154448	0.0000000	2.5654060
H	-1.3732947	0.0000000	-2.3954742	-1.3575034	0.0000000	-2.3970232
H	1.3732947	0.0000000	-2.3954742	1.3575034	0.0000000	-2.3970232
H	-5.5671733	0.0000000	-2.3868172	-5.5647417	0.0000000	-2.3925581
H	-8.3139441	0.0000000	-2.3751754	-8.2964502	0.0000000	-2.3702381
H	4.8333337	0.0000000	2.5689661	4.8154448	0.0000000	2.5654060
H	2.0866658	0.0000000	2.5633850	2.0948465	0.0000000	2.5688586
H	-2.0866658	0.0000000	2.5633850	-2.0948465	0.0000000	2.5688586
H	8.3139441	0.0000000	-2.3751754	8.2964502	0.0000000	-2.3702381
H	5.5671733	0.0000000	-2.3868172	5.5647417	0.0000000	-2.3925581
H	11.7568888	0.0000000	2.5996319	11.7391110	0.0000000	2.5970470
C	12.7743733	0.0000000	-0.1980396	12.7526732	0.0000000	-0.1986794
H	9.0090281	0.0000000	2.5863240	8.9970543	0.0000000	2.5912584
H	-9.0090281	0.0000000	2.5863240	-8.9970543	0.0000000	2.5912584
H	-11.7568888	0.0000000	2.5996319	-11.739111	0.0000000	2.5970470
H	-12.5065998	0.0000000	-2.3533117	-12.488295	0.0000000	-2.3559469
H	-15.2532517	0.0000000	-2.3306759	-15.233856	0.0000000	-2.3301694
H	-15.9014975	0.0000000	0.3222897	-15.880353	0.0000000	0.3230799
O	13.8566988	0.0000000	0.6162403	13.8355541	0.0000000	0.6163715
C	14.9508534	0.0000000	-0.1822932	14.9297318	0.0000000	-0.1816699
C	14.5891275	0.0000000	-1.4826572	14.5687015	0.0000000	-1.4829010
C	13.1605404	0.0000000	-1.4978356	13.1413435	0.0000000	-1.4997772
H	12.5065998	0.0000000	-2.3533117	12.4882957	0.0000000	-2.3559469
H	15.2532517	0.0000000	-2.3306759	15.2338567	0.0000000	-2.3301694
H	15.9014975	0.0000000	0.3222897	15.8803535	0.0000000	0.3230799

Part 10.

2O/ CC2	Ground state			Excited state		
C	-2.8191046	0.4361825	0.0000000	-2.8010893	0.4502933	0.0000000
C	-2.7984455	-0.9288921	0.0000000	-2.7725994	-0.9421680	0.0000000
C	-1.4255912	-1.3126309	0.0000000	-1.4377773	-1.3440648	0.0000000
C	-0.7021728	-0.1460385	0.0000000	-0.6722342	-0.1353455	0.0000000
C	0.7021728	0.1460385	0.0000000	0.6722342	0.1353455	0.0000000
C	1.4255912	1.3126309	0.0000000	1.4377773	1.3440648	0.0000000
C	2.7984455	0.9288921	0.0000000	2.7725994	0.9421680	0.0000000
C	2.8191046	-0.4361825	0.0000000	2.8010893	-0.4502933	0.0000000
O	-1.5433475	0.9354033	0.0000000	-1.5350652	0.9739632	0.0000000
O	1.5433475	-0.9354033	0.0000000	1.5350652	-0.9739632	0.0000000
H	-3.6027594	1.1716188	0.0000000	-3.6037101	1.1675264	0.0000000
H	-3.6585261	-1.5759251	0.0000000	-3.6465226	-1.5728789	0.0000000
H	-1.0140409	-2.3069428	0.0000000	-1.0260996	-2.3384746	0.0000000
H	1.0140409	2.3069428	0.0000000	1.0260996	2.3384746	0.0000000
H	3.6585261	1.5759251	0.0000000	3.6465226	1.5728789	0.0000000
H	3.6027594	-1.1716188	0.0000000	3.6037101	-1.1675264	0.0000000
3O/ CC2	Ground state			Excited state		
O	3.4730317	0.0000000	0.8436679	3.4712753	0.0000000	0.8627926
C	4.5697412	0.0000000	0.0226257	4.5525950	0.0000000	0.0230776
C	4.1864400	0.0000000	-1.2880010	4.1516194	0.0000000	-1.2961930
C	2.7615591	0.0000000	-1.2931529	2.7452928	0.0000000	-1.3045899
C	2.3739077	0.0000000	0.0249759	2.3490542	0.0000000	0.0473573
C	1.1005680	0.0000000	0.6785335	1.1088564	0.0000000	0.6629274
O	0.0000000	0.0000000	-0.1417334	0.0000000	0.0000000	-0.1783493
C	-1.1005680	0.0000000	0.6785335	-1.1088564	0.0000000	0.6629274
C	-0.7098425	0.0000000	1.9968941	-0.6900978	0.0000000	2.0208156
C	0.7098425	0.0000000	1.9968941	0.6900978	0.0000000	2.0208156
H	1.3721057	0.0000000	2.8456549	1.3587985	0.0000000	2.8655155
H	-1.3721057	0.0000000	2.8456549	-1.3587985	0.0000000	2.8655155
H	2.1008227	0.0000000	-2.1426668	2.0766375	0.0000000	-2.1489570
H	5.5209837	0.0000000	0.5228422	5.5149118	0.0000000	0.5042737
H	4.8435015	0.0000000	-2.1405067	4.8115506	0.0000000	-2.1478452
C	-2.3739077	0.0000000	0.0249759	-2.3490542	0.0000000	0.0473573
O	-3.4730317	0.0000000	0.8436679	-3.4712753	0.0000000	0.8627926
C	-4.5697412	0.0000000	0.0226257	-4.5525950	0.0000000	0.0230776
C	-4.1864400	0.0000000	-1.2880010	-4.1516194	0.0000000	-1.2961930
C	-2.7615591	0.0000000	-1.2931529	-2.7452928	0.0000000	-1.3045899
H	-2.1008227	0.0000000	-2.1426668	-2.0766375	0.0000000	-2.1489570
H	-5.5209837	0.0000000	0.5228422	-5.5149118	0.0000000	0.5042737
H	-4.8435015	0.0000000	-2.1405067	-4.8115506	0.0000000	-2.1478452
4O/ CC2	Ground state			Excited state		
O	-1.5209479	0.9728153	0.0000000	-1.5064155	1.0097914	0.0000000
C	-2.8108006	0.5031317	0.0000000	-2.8029584	0.5286744	0.0000000

C	-2.8151192	-0.8725185	0.0000000	-2.7958125	-0.8789099	0.0000000
C	-1.4564907	-1.2807896	0.0000000	-1.4720583	-1.2865191	0.0000000
C	-0.7019455	-0.1293685	0.0000000	-0.6802417	-0.1087082	0.0000000
C	0.7019455	0.1293685	0.0000000	0.6802417	0.1087082	0.0000000
O	1.5209479	-0.9728153	0.0000000	1.5064155	-1.0097914	0.0000000
C	2.8108006	-0.5031317	0.0000000	2.8029584	-0.5286744	0.0000000
C	2.8151192	0.8725185	0.0000000	2.7958125	0.8789099	0.0000000
C	1.4564907	1.2807896	0.0000000	1.4720583	1.2865191	0.0000000
H	1.0667387	2.2844450	0.0000000	1.0801736	2.2902566	0.0000000
H	3.6935727	1.4949653	0.0000000	3.6807197	1.4939827	0.0000000
H	-1.0667387	-2.2844450	0.0000000	-1.0801736	-2.2902566	0.0000000
H	-3.6935727	-1.4949653	0.0000000	-3.6807197	-1.4939827	0.0000000
C	3.8429327	-1.4939487	0.0000000	3.8273570	-1.4802636	0.0000000
O	5.1307465	-1.0247803	0.0000000	5.1253709	-1.0096041	0.0000000
C	5.9458468	-2.1258748	0.0000000	5.9361968	-2.1122349	0.0000000
C	5.2027479	-3.2716142	0.0000000	5.1899305	-3.2651334	0.0000000
C	3.8363687	-2.8681131	0.0000000	3.8315792	-2.8759505	0.0000000
H	2.9598227	-3.4925820	0.0000000	2.9560717	-3.5030307	0.0000000
H	7.0006123	-1.9195298	0.0000000	6.9927780	-1.9109937	0.0000000
H	5.5877685	-4.2767365	0.0000000	5.5835521	-4.2677273	0.0000000
C	-3.8429327	1.4939487	0.0000000	-3.8273570	1.4802636	0.0000000
O	-5.1307465	1.0247803	0.0000000	-5.1253709	1.0096041	0.0000000
C	-5.9458468	2.1258748	0.0000000	-5.9361968	2.1122349	0.0000000
C	-5.2027479	3.2716142	0.0000000	-5.1899305	3.2651334	0.0000000
C	-3.8363687	2.8681131	0.0000000	-3.8315792	2.8759505	0.0000000
H	-7.0006123	1.9195298	0.0000000	-6.9927780	1.9109937	0.0000000
H	-5.5877685	4.2767365	0.0000000	-5.5835521	4.2677273	0.0000000
H	-2.9598227	3.4925820	0.0000000	-2.9560717	3.5030307	0.0000000
**5O/ CC2**	**Ground state**			**Excited state**		
O	0.0000000	0.0000000	0.7160720	0.0000000	0.0000000	0.7524122
C	1.1011363	0.0000000	-0.1045293	1.1092399	0.0000000	-0.0807231
C	0.7087424	0.0000000	-1.4247847	0.6909588	0.0000000	-1.4329479
C	-0.7087424	0.0000000	-1.4247847	-0.6909588	0.0000000	-1.4329479
C	-1.1011363	0.0000000	-0.1045293	-1.1092399	0.0000000	-0.0807231
C	-2.3713930	0.0000000	0.5453666	-2.3547339	0.0000000	0.5206767
O	-3.4720856	0.0000000	-0.2758613	-3.4665006	0.0000000	-0.3078825
C	-4.5731184	0.0000000	0.5438845	-4.5685374	0.0000000	0.5194769
C	-4.1828029	0.0000000	1.8631978	-4.1633571	0.0000000	1.8580365
C	-2.7644449	0.0000000	1.8650206	-2.7717627	0.0000000	1.8688426
H	-4.8459926	0.0000000	2.7113278	-4.8334201	0.0000000	2.7020166
H	-1.3707417	0.0000000	-2.2739758	-1.3576151	0.0000000	-2.2797262
H	1.3707417	0.0000000	-2.2739758	1.3576151	0.0000000	-2.2797262
C	-5.8459885	0.0000000	-0.1091603	-5.8304628	0.0000000	-0.1045304
O	-6.9450703	0.0000000	0.7097591	-6.9323413	0.0000000	0.7217628
C	-8.0417720	0.0000000	-0.1111947	-8.0303089	0.0000000	-0.0955119
C	-7.6586246	0.0000000	-1.4219935	-7.6527221	0.0000000	-1.4130449
C	-6.2339782	0.0000000	-1.4274939	-6.2359891	0.0000000	-1.4322591
H	-5.5734942	0.0000000	-2.2772381	-5.5809030	0.0000000	-2.2867651

H	-8.9930026	0.0000000	0.3890795	-8.9812980	0.0000000	0.4064354
H	-8.3158353	0.0000000	-2.2743931	-8.3185942	0.0000000	-2.2593609
C	2.3713930	0.0000000	0.5453666	2.3547339	0.0000000	0.5206767
O	3.4720856	0.0000000	-0.2758613	3.4665006	0.0000000	-0.3078825
C	4.5731184	0.0000000	0.5438845	4.5685374	0.0000000	0.5194769
C	4.1828029	0.0000000	1.8631978	4.1633571	0.0000000	1.8580365
C	2.7644449	0.0000000	1.8650206	2.7717627	0.0000000	1.8688426
H	4.8459926	0.0000000	2.7113278	4.8334201	0.0000000	2.7020166
H	2.1034620	0.0000000	2.7149530	2.1100893	0.0000000	2.7192985
H	-2.1034620	0.0000000	2.7149530	-2.1100893	0.0000000	2.7192985
C	5.8459885	0.0000000	-0.1091603	5.8304628	0.0000000	-0.1045304
C	6.2339782	0.0000000	-1.4274939	6.2359891	0.0000000	-1.4322591
C	7.6586246	0.0000000	-1.4219935	7.6527221	0.0000000	-1.4130449
C	8.0417720	0.0000000	-0.1111947	8.0303089	0.0000000	-0.0955119
O	6.9450703	0.0000000	0.7097591	6.9323413	0.0000000	0.7217628
H	8.3158353	0.0000000	-2.2743931	8.3185942	0.0000000	-2.2593609
H	8.9930026	0.0000000	0.3890795	8.9812980	0.0000000	0.4064354
H	5.5734942	0.0000000	-2.2772381	5.5809030	0.0000000	-2.2867651
**6O/ CC2**	**Ground state**			**Excited state**		
O	1.8055341	0.0096084	0.0000000	1.8123930	0.0441333	0.0000000
C	0.5238156	-0.4838230	0.0000000	0.5189731	-0.4557841	0.0000000
C	0.5459667	-1.8613206	0.0000000	0.5580386	-1.8696675	0.0000000
C	1.9107136	-2.2431969	0.0000000	1.8923847	-2.2386179	0.0000000
C	2.6446576	-1.0774563	0.0000000	2.6545088	-1.0534497	0.0000000
C	4.0429809	-0.7943722	0.0000000	4.0229230	-0.8059216	0.0000000
O	4.8813573	-1.8820852	0.0000000	4.8671899	-1.9017008	0.0000000
C	6.1626737	-1.3897371	0.0000000	6.1507858	-1.4074628	0.0000000
C	6.1427176	-0.0139815	0.0000000	6.1268329	-0.0154501	0.0000000
C	4.7775223	0.3703853	0.0000000	4.7843202	0.3729793	0.0000000
H	7.0101363	0.6237756	0.0000000	6.9997709	0.6161577	0.0000000
H	2.3193579	-3.2393910	0.0000000	2.3069920	-3.2334454	0.0000000
H	-0.3203840	-2.5007430	0.0000000	-0.3104969	-2.5076948	0.0000000
C	7.2122619	-2.3618148	0.0000000	7.1981726	-2.3580771	0.0000000
O	8.4914468	-1.8695856	0.0000000	8.4804013	-1.8611371	0.0000000
C	9.3261288	-2.9558584	0.0000000	9.3171410	-2.9446081	0.0000000
C	8.6037150	-4.1147987	0.0000000	8.5977856	-4.1093240	0.0000000
C	7.2303769	-3.7359498	0.0000000	7.2269957	-3.7414855	0.0000000
H	6.3652243	-4.3761218	0.0000000	6.3649628	-4.3863939	0.0000000
H	10.3770291	-2.7306320	0.0000000	10.3680670	-2.7176220	0.0000000
H	9.0067347	-5.1128387	0.0000000	9.0079773	-5.1048285	0.0000000
C	-0.5238156	0.4838230	0.0000000	-0.5189731	0.4557841	0.0000000
O	-1.8055341	-0.0096084	0.0000000	-1.8123930	-0.0441333	0.0000000
C	-2.6446576	1.0774563	0.0000000	-2.6545088	1.0534497	0.0000000
C	-1.9107136	2.2431969	0.0000000	-1.8923847	2.2386179	0.0000000
C	-0.5459667	1.8613206	0.0000000	-0.5580386	1.8696675	0.0000000
H	-2.3193579	3.2393910	0.0000000	-2.3069920	3.2334454	0.0000000
H	0.3203840	2.5007430	0.0000000	0.3104969	2.5076948	0.0000000
H	4.3703412	1.3671306	0.0000000	4.3793720	1.3714078	0.0000000

C	-4.0429809	0.7943722	0.0000000	-4.0229230	0.8059216	0.0000000
C	-4.7775223	-0.3703853	0.0000000	-4.7843202	-0.3729793	0.0000000
C	-6.1427176	0.0139815	0.0000000	-6.1268329	0.0154501	0.0000000
C	-6.1626737	1.3897371	0.0000000	-6.1507858	1.4074628	0.0000000
O	-4.8813573	1.8820852	0.0000000	-4.8671899	1.9017008	0.0000000
H	-7.0101363	-0.6237756	0.0000000	-6.9997709	-0.6161577	0.0000000
H	-4.3703412	-1.3671306	0.0000000	-4.3793720	-1.3714078	0.0000000
C	-7.2122619	2.3618148	0.0000000	-7.1981726	2.3580771	0.0000000
C	-7.2303769	3.7359498	0.0000000	-7.2269957	3.7414855	0.0000000
C	-8.6037150	4.1147987	0.0000000	-8.5977856	4.1093240	0.0000000
C	-9.3261288	2.9558584	0.0000000	-9.3171410	2.9446081	0.0000000
O	-8.4914468	1.8695856	0.0000000	-8.4804013	1.8611371	0.0000000
H	-9.0067347	5.1128387	0.0000000	-9.0079773	5.1048285	0.0000000
H	-10.3770291	2.7306320	0.0000000	-10.368067	2.7176220	0.0000000
H	-6.3652243	4.3761218	0.0000000	-6.3649628	4.3863939	0.0000000
**7O/ CC2**	**Ground state**			**Excited state**		
C	-9.7061488	0.0000000	-1.4878127	-9.6999763	0.0000000	-1.4954639
C	-9.3179805	0.0000000	-0.1695154	-9.3036077	0.0000000	-0.1728228
O	-10.4169294	0.0000000	0.6495701	-10.404285	0.0000000	0.6492531
C	-11.5137222	0.0000000	-0.1712364	-11.501397	0.0000000	-0.1698530
C	-11.1307635	0.0000000	-1.4821010	-11.120812	0.0000000	-1.4837948
C	-8.0450888	0.0000000	0.4833999	-8.0370066	0.0000000	0.4672243
O	-6.9440530	0.0000000	-0.3362831	-6.9336165	0.0000000	-0.3516669
C	-5.8433864	0.0000000	0.4850264	-5.8280116	0.0000000	0.4755163
C	-6.2365758	0.0000000	1.8047424	-6.2407820	0.0000000	1.8100419
C	-7.6548165	0.0000000	1.8028074	-7.6432485	0.0000000	1.7979034
C	-4.5732772	0.0000000	-0.1646991	-4.5723788	0.0000000	-0.1391252
O	-3.4720077	0.0000000	0.6556634	-3.4669720	0.0000000	0.6873898
C	-2.3709121	0.0000000	-0.1652745	-2.3567718	0.0000000	-0.1402749
C	-2.7639438	0.0000000	-1.4857846	-2.7739933	0.0000000	-1.4871851
C	-4.1810244	0.0000000	-1.4852770	-4.1627617	0.0000000	-1.4807829
C	-1.1012247	0.0000000	0.4837805	-1.1091389	0.0000000	0.4603572
O	0.0000000	0.0000000	-0.3369205	0.0000000	0.0000000	-0.3690875
C	1.1012247	0.0000000	0.4837805	1.1091389	0.0000000	0.4603572
C	0.7084227	0.0000000	1.8044901	0.6916654	0.0000000	1.8095622
C	-0.7084227	0.0000000	1.8044901	-0.6916654	0.0000000	1.8095622
C	2.3709121	0.0000000	-0.1652745	2.3567718	0.0000000	-0.1402749
O	3.4720077	0.0000000	0.6556634	3.4669720	0.0000000	0.6873898
C	4.5732772	0.0000000	-0.1646991	4.5723788	0.0000000	-0.1391252
C	4.1810244	0.0000000	-1.4852770	4.1627617	0.0000000	-1.4807829
C	2.7639438	0.0000000	-1.4857846	2.7739933	0.0000000	-1.4871851
C	5.8433864	0.0000000	0.4850264	5.8280116	0.0000000	0.4755163
O	6.9440530	0.0000000	-0.3362831	6.9336165	0.0000000	-0.3516669
C	8.0450888	0.0000000	0.4833999	8.0370066	0.0000000	0.4672243
C	7.6548165	0.0000000	1.8028074	7.6432485	0.0000000	1.7979034
C	6.2365758	0.0000000	1.8047424	6.2407820	0.0000000	1.8100419
C	9.3179805	0.0000000	-0.1695154	9.3036077	0.0000000	-0.1728228
C	9.7061488	0.0000000	-1.4878127	9.6999763	0.0000000	-1.4954639

C	11.1307635	0.0000000	-1.4821010	11.1208127	0.0000000	-1.4837948
C	11.5137222	0.0000000	-0.1712364	11.5013979	0.0000000	-0.1698530
O	10.4169294	0.0000000	0.6495701	10.4042850	0.0000000	0.6492531
H	-1.3703591	0.0000000	2.6537771	-1.3573146	0.0000000	2.6572048
H	2.1022872	0.0000000	-2.3352843	2.1114009	0.0000000	-2.3370326
H	4.8432763	0.0000000	-2.3342879	4.8303461	0.0000000	-2.3268235
H	-2.1022872	0.0000000	-2.3352843	-2.1114009	0.0000000	-2.3370326
H	-4.8432763	0.0000000	-2.3342879	-4.8303461	0.0000000	-2.3268235
H	8.3180632	0.0000000	2.6508952	8.3132787	0.0000000	2.6414796
H	5.5756960	0.0000000	2.6547677	5.5830166	0.0000000	2.6631975
H	1.3703591	0.0000000	2.6537771	1.3573146	0.0000000	2.6572048
H	11.7880856	0.0000000	-2.3344128	11.7819125	0.0000000	-2.3334930
H	9.0458130	0.0000000	-2.3376759	9.0421551	0.0000000	-2.3475581
H	-5.5756960	0.0000000	2.6547677	-5.5830166	0.0000000	2.6631975
H	-8.3180632	0.0000000	2.6508952	-8.3132787	0.0000000	2.6414796
H	-9.0458130	0.0000000	-2.3376759	-9.0421551	0.0000000	-2.3475581
H	-11.7880856	0.0000000	-2.3344128	-11.781912	0.0000000	-2.3334930
H	-12.4648778	0.0000000	0.3291847	-12.452487	0.0000000	0.3312904
H	12.4648778	0.0000000	0.3291847	12.4524873	0.0000000	0.3312904
**8O/ CC2**	**Ground state**			**Excited state**		
O	-11.8921393	2.5513182	0.0000000	-11.879824	2.5471693	0.0000000
C	-10.6238342	3.0709671	0.0000000	-10.609996	3.0688549	0.0000000
C	-10.6715058	4.4443927	0.0000000	-10.662898	4.4466236	0.0000000
C	-12.0526747	4.7936019	0.0000000	-12.042597	4.7907486	0.0000000
C	-12.7499677	3.6193575	0.0000000	-12.738474	3.6138202	0.0000000
C	-9.5536543	2.1216013	0.0000000	-9.5417411	2.1296436	0.0000000
C	-9.5042527	0.7465608	0.0000000	-9.4923851	0.7460559	0.0000000
C	-8.1311699	0.3915092	0.0000000	-8.1327619	0.3861991	0.0000000
C	-7.4217295	1.5717442	0.0000000	-7.4058797	1.5731001	0.0000000
O	-8.2831936	2.6412707	0.0000000	-8.2699113	2.6477301	0.0000000
C	-6.0298496	1.8847971	0.0000000	-6.0316644	1.8629495	0.0000000
O	-5.1675021	0.8161032	0.0000000	-5.1664407	0.7905770	0.0000000
C	-3.8966942	1.3370766	0.0000000	-3.8870977	1.3153822	0.0000000
C	-3.9485988	2.7138775	0.0000000	-3.9553890	2.7183700	0.0000000
C	-5.3211705	3.0661642	0.0000000	-5.3070533	3.0577017	0.0000000
C	-2.8286371	0.3923311	0.0000000	-2.8263376	0.4171320	0.0000000
O	-1.5577390	0.9129056	0.0000000	-1.5475131	0.9431145	0.0000000
C	-0.6955794	-0.1562033	0.0000000	-0.6787765	-0.1351522	0.0000000
C	-1.4050056	-1.3375279	0.0000000	-1.4189390	-1.3369958	0.0000000
C	-2.7771236	-0.9846566	0.0000000	-2.7598978	-0.9894472	0.0000000
C	0.6955794	0.1562033	0.0000000	0.6787765	0.1351522	0.0000000
O	1.5577390	-0.9129056	0.0000000	1.5475131	-0.9431145	0.0000000
C	2.8286371	-0.3923311	0.0000000	2.8263376	-0.4171320	0.0000000
C	2.7771236	0.9846566	0.0000000	2.7598978	0.9894472	0.0000000
C	1.4050056	1.3375279	0.0000000	1.4189390	1.3369958	0.0000000
C	3.8966942	-1.3370766	0.0000000	3.8870977	-1.3153822	0.0000000
O	5.1675021	-0.8161032	0.0000000	5.1664407	-0.7905770	0.0000000
C	6.0298496	-1.8847971	0.0000000	6.0316644	-1.8629495	0.0000000

C	5.3211705	-3.0661642	0.0000000	5.3070533	-3.0577017	0.0000000
C	3.9485988	-2.7138775	0.0000000	3.9553890	-2.7183700	0.0000000
H	3.6297141	1.6423321	0.0000000	3.6161223	1.6440192	0.0000000
H	-0.9755086	-2.3249442	0.0000000	-0.9869032	-2.3243258	0.0000000
H	-3.6297141	-1.6423321	0.0000000	-3.6161223	-1.6440192	0.0000000
H	3.0963116	-3.3719421	0.0000000	3.1034371	-3.3780777	0.0000000
C	7.4217295	-1.5717442	0.0000000	7.4058797	-1.5731001	0.0000000
H	5.7511829	-4.0533225	0.0000000	5.7434189	-4.0429864	0.0000000
H	-5.7511829	4.0533225	0.0000000	-5.7434189	4.0429864	0.0000000
H	-3.0963116	3.3719421	0.0000000	-3.1034371	3.3780777	0.0000000
H	0.9755086	2.3249442	0.0000000	0.9869032	2.3243258	0.0000000
H	-10.3577968	0.0903537	0.0000000	-10.350181	0.0945868	0.0000000
H	-7.7028123	-0.5963242	0.0000000	-7.7076800	-0.6035272	0.0000000
H	-12.4770499	5.7827457	0.0000000	-12.470256	5.7786678	0.0000000
H	-13.7958086	3.3716878	0.0000000	-13.784307	3.3652736	0.0000000
H	-9.8203473	5.1030582	0.0000000	-9.8133001	5.1075565	0.0000000
O	8.2831936	-2.6412707	0.0000000	8.2699113	-2.6477301	0.0000000
C	9.5536543	-2.1216013	0.0000000	9.5417411	-2.1296436	0.0000000
C	9.5042527	-0.7465608	0.0000000	9.4923851	-0.7460559	0.0000000
C	8.1311699	-0.3915092	0.0000000	8.1327619	-0.3861991	0.0000000
H	7.7028123	0.5963242	0.0000000	7.7076800	0.6035272	0.0000000
H	10.3577968	-0.0903537	0.0000000	10.3501817	-0.0945868	0.0000000
C	10.6238342	-3.0709671	0.0000000	10.6099962	-3.0688549	0.0000000
O	11.8921393	-2.5513182	0.0000000	11.8798246	-2.5471693	0.0000000
C	12.7499677	-3.6193575	0.0000000	12.7384742	-3.6138202	0.0000000
C	12.0526747	-4.7936019	0.0000000	12.0425978	-4.7907486	0.0000000
C	10.6715058	-4.4443927	0.0000000	10.6628989	-4.4466236	0.0000000
H	9.8203473	-5.1030582	0.0000000	9.8133001	-5.1075565	0.0000000
H	12.4770499	-5.7827457	0.0000000	12.4702568	-5.7786678	0.0000000
H	13.7958086	-3.3716878	0.0000000	13.7843074	-3.3652736	0.0000000
**9O/ CC2**	**Ground state**			**Excited state**		
C	-13.1781107	0.0000000	-1.5220002	-13.168586	0.0000000	-1.5258058
C	-12.7898800	0.0000000	-0.2037205	-12.776021	0.0000000	-0.2054874
O	-13.8887769	0.0000000	0.6154249	-13.875641	0.0000000	0.6152882
C	-14.9855978	0.0000000	-0.2053169	-14.972719	0.0000000	-0.2043459
C	-14.6027175	0.0000000	-1.5162126	-14.591277	0.0000000	-1.5168442
C	-11.5169660	0.0000000	0.4491447	-11.506363	0.0000000	0.4409500
O	-10.4159569	0.0000000	-0.3705378	-10.403919	0.0000000	-0.3772716
C	-9.3152865	0.0000000	0.4507604	-9.3006272	0.0000000	0.4474443
C	-9.7084616	0.0000000	1.7704902	-9.7056997	0.0000000	1.7749198
C	-11.1266844	0.0000000	1.7685689	-11.115137	0.0000000	1.7665525
C	-8.0452157	0.0000000	-0.1990018	-8.0391436	0.0000000	-0.1819203
O	-6.9438987	0.0000000	0.6212554	-6.9348572	0.0000000	0.6394323
C	-5.8428748	0.0000000	-0.1997862	-5.8279711	0.0000000	-0.1862918
C	-6.2360311	0.0000000	-1.5202890	-6.2403017	0.0000000	-1.5235110
C	-7.6530672	0.0000000	-1.5196502	-7.6384889	0.0000000	-1.5158106
C	-4.5732128	0.0000000	0.4491844	-4.5737834	0.0000000	0.4264953
O	-3.4719523	0.0000000	-0.3714094	-3.4671792	0.0000000	-0.3989258

C	-2.3707695	0.0000000	0.4494008	-2.3579993	0.0000000	0.4281089
C	-2.7637638	0.0000000	1.7701713	-2.7747475	0.0000000	1.7733724
C	-4.1804824	0.0000000	1.7700140	-4.1634374	0.0000000	1.7685276
C	-1.1012335	0.0000000	-0.1994534	-1.1088190	0.0000000	-0.1740930
O	0.0000000	0.0000000	0.6212389	0.0000000	0.0000000	0.6534950
C	1.1012335	0.0000000	-0.1994534	1.1088190	0.0000000	-0.1740930
C	0.7083306	0.0000000	-1.5203010	0.6924721	0.0000000	-1.5211085
C	-0.7083306	0.0000000	-1.5203010	-0.6924721	0.0000000	-1.5211085
C	2.3707695	0.0000000	0.4494008	2.3579993	0.0000000	0.4281089
O	3.4719523	0.0000000	-0.3714094	3.4671792	0.0000000	-0.3989258
C	4.5732128	0.0000000	0.4491844	4.5737834	0.0000000	0.4264953
C	4.1804824	0.0000000	1.7700140	4.1634374	0.0000000	1.7685276
C	2.7637638	0.0000000	1.7701713	2.7747475	0.0000000	1.7733724
C	5.8428748	0.0000000	-0.1997862	5.8279711	0.0000000	-0.1862918
C	6.2360311	0.0000000	-1.5202890	6.2403017	0.0000000	-1.5235110
C	7.6530672	0.0000000	-1.5196502	7.6384889	0.0000000	-1.5158106
C	8.0452157	0.0000000	-0.1990018	8.0391436	0.0000000	-0.1819203
O	6.9438987	0.0000000	0.6212554	6.9348572	0.0000000	0.6394323
C	9.3152865	0.0000000	0.4507604	9.3006272	0.0000000	0.4474443
O	10.4159569	0.0000000	-0.3705378	10.4039193	0.0000000	-0.3772716
C	11.5169660	0.0000000	0.4491447	11.5063636	0.0000000	0.4409500
C	11.1266844	0.0000000	1.7685689	11.1151378	0.0000000	1.7665525
C	9.7084616	0.0000000	1.7704902	9.7056997	0.0000000	1.7749198
H	-4.8425121	0.0000000	2.6192293	-4.8302325	0.0000000	2.6151851
H	-1.3702548	0.0000000	-2.3696074	-1.3576402	0.0000000	-2.3691039
H	1.3702548	0.0000000	-2.3696074	1.3576402	0.0000000	-2.3691039
H	-5.5744697	0.0000000	-2.3698675	-5.5801552	0.0000000	-2.3749649
H	-8.3153984	0.0000000	-2.3685974	-8.3059455	0.0000000	-2.3616183
H	4.8425121	0.0000000	2.6192293	4.8302325	0.0000000	2.6151851
H	2.1019517	0.0000000	2.6195672	2.1120471	0.0000000	2.6231498
H	-2.1019517	0.0000000	2.6195672	-2.1120471	0.0000000	2.6231498
H	8.3153984	0.0000000	-2.3685974	8.3059455	0.0000000	-2.3616183
H	5.5744697	0.0000000	-2.3698675	5.5801552	0.0000000	-2.3749649
H	11.7899280	0.0000000	2.6166583	11.7825974	0.0000000	2.6118022
C	12.7898800	0.0000000	-0.2037205	12.7760212	0.0000000	-0.2054874
H	9.0475868	0.0000000	2.6205213	9.0472507	0.0000000	2.6272501
H	-9.0475868	0.0000000	2.6205213	-9.0472507	0.0000000	2.6272501
H	-11.7899280	0.0000000	2.6166583	-11.782597	0.0000000	2.6118022
H	-12.5178333	0.0000000	-2.3719085	-12.509786	0.0000000	-2.3770018
H	-15.2600863	0.0000000	-2.3684861	-15.250638	0.0000000	-2.3677295
H	-15.9367364	0.0000000	0.2951353	-15.923754	0.0000000	0.2966084
O	13.8887769	0.0000000	0.6154249	13.8756417	0.0000000	0.6152882
C	14.9855978	0.0000000	-0.2053169	14.9727196	0.0000000	-0.2043459
C	14.6027175	0.0000000	-1.5162126	14.5912778	0.0000000	-1.5168442
C	13.1781107	0.0000000	-1.5220002	13.1685863	0.0000000	-1.5258058
H	12.5178333	0.0000000	-2.3719085	12.5097869	0.0000000	-2.3770018
H	15.2600863	0.0000000	-2.3684861	15.2506389	0.0000000	-2.3677295
H	15.9367364	0.0000000	0.2951353	15.9237544	0.0000000	0.2966084

Table S2. The excited state and the ground state reorganization energies calculated using the studied exchange-correlation functionals with the def2-TZVPP basis set. For CC2 geometry optimization for the excited state was not completed due to prohibitively large computational cost.

		2O	3O	4O	5O	6O	7O	8O	9O
**BP**	**exc. st.**	0,200	0,132	0,100	0,085	0,078	0,074	0,073	0,073
	**gr. st.**	0,193	0,123	0,090	0,075	0,069	0,066	0,065	0,065
**TPSSh**	**exc. st.**	0,236	0,172	0,143	0,130	0,124	0,131	0,129	0,128
	**gr. st.**	0,242	0,173	0,141	0,126	0,119	0,117	0,116	0,116
**B3LYP**	**exc. st.**	0,281	0,221	0,194	0,181	0,175	0,172	0,169	0,167
	**gr. st.**	0,264	0,202	0,173	0,161	0,156	0,155	0,154	0,154
**PBE0**	**exc. st.**	0,293	0,236	0,210	0,198	0,192	0,188	0,185	0,181
	**gr. st.**	0,277	0,217	0,190	0,179	0,174	0,172	0,171	0,170
**MN15**	**exc. st.**	0,346	0,296	0,274	0,263	0,254	0,246	0,232	0,229
	**gr. st.**	0,324	0,273	0,252	0,243	0,239	0,235	0,231	0,227
**BHLYP**	**gr. st.**	0,390	0,343	0,322	0,309	0,299	0,289	0,278	0,267
	**exc. st.**	0,366	0,317	0,297	0,289	0,284	0,280	0,275	0,270
**CAM-B3LYP**	**gr. st.**	0,376	0,332	0,312	0,299	0,288	0,276	0,265	0,252
	**exc. st.**	0,354	0,309	0,291	0,284	0,278	0,272	0,266	0,259
**tuned CAM-B3LYP**	**gr. st.**	0,295	0,252	0,234	0,224	0,217	0,209	0,202	0,192
	**exc. st.**	0,279	0,235	0,217	0,210	0,206	0,202	0,197	0,192
**ωB97X**	**gr. st.**	0,422	0,382	0,360	0,341	0,323	0,304	0,287	0,269

	exc. st.	0,401	0,364	0,348	0,338	0,328	0,317	0,305	0,294
**CC2**	**gr. st.**	0,336	0,292	0,279	0,266	0,273	0,269		
	**exc. st.**	0,307	0,261	0,249	0,249	0,253	0,256		

Table S3. Vibrational frequencies, FC parameters, HR factors, and the related reorganization energies for the totally symmetric normal modes of 2O, 3O, and 4O, calculated using the studied exchange-correlation functionals with the def2-TZVPP basis set.

Part 1.

2O/BP									
Emission					Absorption				
$\tilde{\nu}$ [cm^{-1}]	B_i	S_i	λ_i [cm^{-1}]	%(λ)	$\tilde{\nu}$ [cm^{-1}]	B_i	S_i	λ_i [cm^{-1}]	%(λ)
384	-0,248	0,031	11,8	0,7%	376	-0,27	0,036	13,7	0,9%
447	-0,227	0,026	11,5	0,7%	445	-0,182	0,017	7,4	0,5%
873	-0,316	0,050	43,6	2,7%	816	-0,302	0,046	37,2	2,4%
918	-1,046	0,547	502,3	30,6%	847	-1,018	0,518	439,2	28,7%
1006	-0,545	0,149	149,4	9,1%	1009	-0,652	0,213	214,6	14,0%
1079	-0,35	0,061	66,3	4,0%	1050	-0,138	0,010	10,1	0,7%
1144	0,309	0,048	54,5	3,3%	1089	0,204	0,021	22,8	1,5%
1221	-0,089	0,004	4,8	0,3%	1181	-0,226	0,026	30,0	2,0%
1273	-0,269	0,036	46,1	2,8%	1273	-0,19	0,018	22,9	1,5%
1392	-0,299	0,045	62,1	3,8%	1403	0,3	0,045	63,3	4,1%
1498	0,541	0,146	219,4	13,3%	1434	-0,176	0,015	22,1	1,4%
1621	-0,761	0,290	469,5	28,6%	1587	-0,901	0,406	643,8	42,1%
3180	0,007	0,000	0,1	0,0%	3172	0,003	0,000	0,0	0,0%
3197	-0,032	0,001	1,6	0,1%	3199	-0,011	0,000	0,2	0,0%
3213	-0,018	0,000	0,5	0,0%	3209	-0,024	0,000	0,9	0,1%
3O/BP									
Emission					Absorption				
$\tilde{\nu}$ [cm^{-1}]	B_i	S_i	λ_i [cm^{-1}]	%(λ)	$\tilde{\nu}$ [cm^{-1}]	B_i	S_i	λ_i [cm^{-1}]	%(λ)
69	-0,363	0,066	4,5	0,4%	64	-0,359	0,064	4,1	0,4%
279	-0,176	0,015	4,3	0,4%	279	-0,195	0,019	5,3	0,5%
413	-0,348	0,061	25,0	2,4%	413	-0,327	0,053	22,1	2,1%
863	0,004	0,000	0,0	0,0%	810	-0,111	0,006	5,0	0,5%
875	-0,184	0,017	14,8	1,4%	859	-0,112	0,006	5,4	0,5%
938	-0,809	0,327	306,9	29,9%	893	-0,823	0,339	302,7	29,4%
998	0,022	0,000	0,2	0,0%	975	-0,033	0,001	0,5	0,0%
1009	-0,454	0,103	104,1	10,1%	1015	-0,432	0,093	94,6	9,2%
1066	0,196	0,019	20,4	2,0%	1051	0,073	0,003	2,8	0,3%
1082	-0,004	0,000	0,0	0,0%	1087	0,000	0,000	0,0	0,0%
1153	0,134	0,009	10,4	1,0%	1121	0,100	0,005	5,6	0,5%
1203	-0,054	0,001	1,7	0,2%	1175	-0,121	0,007	8,5	0,8%
1279	-0,249	0,031	39,5	3,8%	1276	-0,164	0,013	17,2	1,7%

1333	-0,035	0,001	0,8	0,1%	1321	-0,152	0,012	15,3	1,5%
1388	-0,189	0,018	24,9	2,4%	1385	-0,112	0,006	8,7	0,8%
1428	-0,005	0,000	0,0	0,0%	1402	0,175	0,015	21,5	2,1%
1508	-0,355	0,063	94,9	9,2%	1466	-0,149	0,011	16,2	1,6%
1602	-0,684	0,234	374,4	36,4%	1624	-0,780	0,304	494,0	48,0%
3180	0,006	0,000	0,1	0,0%	3172	0,004	0,000	0,0	0,0%
3197	0,012	0,000	0,2	0,0%	3194	-0,002	0,000	0,0	0,0%
3200	-0,021	0,000	0,7	0,1%	3202	-0,014	0,000	0,3	0,0%
3213	0,006	0,000	0,1	0,0%	3209	-0,008	0,000	0,1	0,0%
4O/BP									
Emission					Absorption				
$\tilde{\nu}$ [cm^{-1}]	B_i	S_i	λ_i [cm^{-1}]	%(λ)	$\tilde{\nu}$ [cm^{-1}]	B_i	S_i	λ_i [cm^{-1}]	%(λ)
97	0,109	0,006	0,6	0,1%	94	0,115	0,007	0,6	0,1%
220	-0,148	0,011	2,4	0,3%	220	-0,162	0,013	2,9	0,4%
400	-0,352	0,062	24,8	3,3%	401	-0,339	0,057	23,1	2,9%
458	0,007	0,000	0,0	0,0%	454	0,01	0,000	0,0	0,0%
552	0,004	0,000	0,0	0,0%	556	0,003	0,000	0,0	0,0%
862	0,004	0,000	0,0	0,0%	835	-0,044	0,001	0,8	0,1%
875	0,109	0,006	5,2	0,7%	866	-0,085	0,004	3,1	0,4%
931	-0,355	0,063	58,7	7,9%	894	0,019	0,000	0,2	0,0%
950	0,57	0,162	154,2	20,7%	915	0,669	0,224	204,8	26,1%
1004	-0,152	0,012	11,7	1,6%	995	-0,049	0,001	1,2	0,2%
1010	0,353	0,062	63,0	8,4%	1020	0,363	0,066	67,2	8,6%
1063	-0,109	0,006	6,3	0,8%	1048	-0,043	0,001	1,0	0,1%
1090	-0,024	0,000	0,3	0,0%	1086	-0,001	0,000	0,0	0,0%
1150	-0,092	0,004	4,9	0,7%	1132	-0,082	0,003	3,8	0,5%
1189	0,017	0,000	0,2	0,0%	1157	0,044	0,001	1,1	0,1%
1214	0,032	0,001	0,6	0,1%	1198	0,049	0,001	1,5	0,2%
1282	-0,233	0,027	34,8	4,7%	1286	-0,048	0,001	1,5	0,2%
1291	0,057	0,002	2,1	0,3%	1294	0,196	0,019	24,8	3,2%
1355	-0,03	0,000	0,6	0,1%	1352	-0,087	0,004	5,2	0,7%
1389	-0,158	0,012	17,4	2,3%	1392	0,164	0,013	18,8	2,4%
1443	-0,034	0,001	0,8	0,1%	1411	0,068	0,002	3,2	0,4%
1510	0,279	0,039	58,9	7,9%	1479	-0,128	0,008	12,1	1,5%
1588	-0,599	0,179	284,6	38,1%	1547	-0,113	0,006	9,8	1,2%
1623	-0,131	0,009	14,0	1,9%	1634	-0,698	0,244	397,7	50,7%
3180	-0,005	0,000	0,0	0,0%	3174	-0,005	0,000	0,0	0,0%
3188	0,001	0,000	0,0	0,0%	3186	0,002	0,000	0,0	0,0%
3197	0,007	0,000	0,1	0,0%	3195	0,007	0,000	0,1	0,0%
3201	0,016	0,000	0,4	0,1%	3200	0,011	0,000	0,2	0,0%
3213	-0,004	0,000	0,0	0,0%	3210	-0,006	0,000	0,0	0,0%

Part 2.

2O/TPSSh									
Emission					Absorption				
$\tilde{\nu}$ [cm^{-1}]	B_i	S_i	λ_i [cm^{-1}]	%(λ)	$\tilde{\nu}$ [cm^{-1}]	B_i	S_i	λ_i [cm^{-1}]	%(λ)
389	-0,308	0,047	18,4	0,9%	384	-0,323	0,052	20,1	1,0%
457	-0,22	0,024	11,1	0,5%	457	-0,16	0,013	5,8	0,3%
890	0,341	0,058	51,8	2,5%	844	-0,171	0,015	12,3	0,6%
943	-1,075	0,578	544,2	26,3%	865	-1,1	0,605	523,1	26,8%
1035	-0,607	0,184	190,5	9,2%	1042	-0,672	0,226	235,5	12,1%

1109	-0,3	0,045	49,8	2,4%	1085	-0,084	0,004	3,9	0,2%
1180	0,344	0,059	69,7	3,4%	1127	-0,184	0,017	19	1,0%
1264	-0,094	0,004	5,6	0,3%	1218	-0,262	0,034	41,7	2,1%
1303	0,267	0,036	46,4	2,2%	1316	0,223	0,025	32,7	1,7%
1436	-0,355	0,063	90,4	4,4%	1445	0,341	0,058	84	4,3%
1542	0,634	0,201	310,2	15,0%	1480	0,167	0,014	20,7	1,1%
1664	-0,901	0,406	675,2	32,7%	1652	-1,074	0,577	952,6	48,8%
3243	0,011	0,000	0,2	0,0%	3235	0,006	0,000	0,1	0,0%
3261	-0,037	0,001	2,2	0,1%	3263	-0,014	0,000	0,3	0,0%
3280	-0,018	0,000	0,5	0,0%	3275	-0,024	0,000	0,9	0,0%
3O/TPSSh									
Emission					Absorption				
$\tilde{\nu}$ [cm^{-1}]	B_i	S_i	λ_i [cm^{-1}]	%(λ)	$\tilde{\nu}$ [cm^{-1}]	B_i	S_i	λ_i [cm^{-1}]	%(λ)
68	-0,292	0,043	2,9	0,2%	63	0,294	0,043	2,7	0,2%
284	-0,245	0,030	8,5	0,6%	285	-0,267	0,036	10,2	0,7%
421	-0,414	0,086	36	2,5%	423	-0,383	0,073	31	2,1%
881	-0,018	0,000	0,1	0,0%	823	-0,161	0,013	10,7	0,7%
892	0,18	0,016	14,4	1,0%	879	-0,172	0,015	12,9	0,9%
962	0,846	0,358	344,7	23,8%	913	0,87	0,378	345,7	23,5%
1028	-0,052	0,001	1,4	0,1%	1004	-0,006	0,000	0	0,0%
1038	-0,574	0,165	170,9	11,8%	1048	-0,489	0,120	125,2	8,5%
1098	0,159	0,013	13,9	1,0%	1085	0,011	0,000	0,1	0,0%
1106	-0,025	0,000	0,4	0,0%	1118	0,024	0,000	0,3	0,0%
1189	0,161	0,013	15,4	1,1%	1160	-0,121	0,007	8,5	0,6%
1243	0,049	0,001	1,5	0,1%	1211	0,119	0,007	8,6	0,6%
1307	0,286	0,041	53,5	3,7%	1310	0,218	0,024	31	2,1%
1373	-0,027	0,000	0,5	0,0%	1361	-0,181	0,016	22,2	1,5%
1430	0,197	0,019	27,8	1,9%	1425	0,104	0,005	7,6	0,5%
1468	0,088	0,004	5,7	0,4%	1455	-0,215	0,023	33,7	2,3%
1553	0,481	0,116	179,9	12,4%	1515	-0,189	0,018	27,2	1,9%
1643	0,832	0,346	568,1	39,3%	1675	-0,972	0,472	791	53,8%
3243	0,008	0,000	0,1	0,0%	3235	-0,006	0,000	0,1	0,0%
3261	0,014	0,000	0,3	0,0%	3257	-0,006	0,000	0,1	0,0%
3265	-0,022	0,000	0,8	0,1%	3266	-0,013	0,000	0,3	0,0%
3280	0,005	0,000	0	0,0%	3275	0,007	0,000	0,1	0,0%
4O/TPSSh									
Emission					Absorption				
$\tilde{\nu}$ [cm^{-1}]	B_i	S_i	λ_i [cm^{-1}]	%(λ)	$\tilde{\nu}$ [cm^{-1}]	B_i	S_i	λ_i [cm^{-1}]	%(λ)
96	0,035	0,001	0,1	0,0%	94	0,045	0,001	0,1	0,0%
224	-0,23	0,026	5,9	0,5%	224	-0,249	0,031	6,9	0,6%
408	-0,45	0,101	41,2	3,5%	410	-0,429	0,092	37,7	3,1%
466	-0,005	0,000	0	0,0%	463	-0,003	0,000	0	0,0%
564	0,001	0,000	0	0,0%	569	-0,002	0,000	0	0,0%
880	-0,015	0,000	0,1	0,0%	852	0,092	0,004	3,6	0,3%
892	-0,106	0,006	5	0,4%	885	0,106	0,006	4,9	0,4%
956	0,365	0,067	63,7	5,5%	918	0,122	0,007	6,9	0,6%
974	-0,616	0,190	185	15,8%	934	-0,724	0,262	245	19,9%
1033	0,205	0,021	21,7	1,9%	1027	0,041	0,001	0,9	0,1%
1039	0,49	0,120	124,8	10,7%	1054	-0,451	0,102	107	8,7%
1096	-0,076	0,003	3,2	0,3%	1082	-0,034	0,001	0,6	0,0%
1116	-0,01	0,000	0,1	0,0%	1114	-0,026	0,000	0,4	0,0%
1186	-0,109	0,006	7,1	0,6%	1171	0,099	0,005	5,7	0,5%

1230	0,012	0,000	0,1	0,0%	1196	0,044	0,001	1,2	0,1%
1256	-0,029	0,000	0,5	0,0%	1238	-0,038	0,001	0,9	0,1%
1310	0,297	0,044	57,6	4,9%	1326	0,147	0,011	14,4	1,2%
1323	-0,049	0,001	1,6	0,1%	1330	-0,234	0,027	36,3	2,9%
1396	0,04	0,001	1,1	0,1%	1397	0,12	0,007	10,1	0,8%
1432	-0,171	0,015	20,9	1,8%	1431	-0,142	0,010	14,5	1,2%
1485	-0,051	0,001	1,9	0,2%	1459	-0,159	0,013	18,4	1,5%
1556	0,409	0,084	130,2	11,2%	1528	-0,168	0,014	21,6	1,8%
1628	0,776	0,301	489,6	41,9%	1604	0,135	0,009	14,7	1,2%
1668	-0,082	0,003	5,6	0,5%	1675	-0,901	0,406	679,9	55,2%
3244	0,006	0,000	0,1	0,0%	3237	-0,006	0,000	0,1	0,0%
3252	0,003	0,000	0	0,0%	3248	0,001	0,000	0	0,0%
3261	0,008	0,000	0,1	0,0%	3259	0,008	0,000	0,1	0,0%
3265	0,018	0,000	0,5	0,0%	3264	0,009	0,000	0,1	0,0%
3280	0,002	0,000	0	0,0%	3276	-0,005	0,000	0	0,0%

Part 3.

2O/B3LYP									
Emission					Absorption				
$\tilde{\nu}$ [cm^{-1}]	B_i	S_i	λ_i [cm^{-1}]	%(λ)	$\tilde{\nu}$ [cm^{-1}]	B_i	S_i	λ_i [cm^{-1}]	%(λ)
393	-0,063	0,002	0,8	0,0%	387	-0,102	0,005	2	0,1%
461	0,265	0,035	16,1	1,0%	472	0,269	0,036	17	1,1%
901	-0,171	0,015	13,1	0,8%	860	-0,156	0,012	10,5	0,7%
951	-0,372	0,069	65,8	3,9%	902	-0,378	0,071	64,5	4,2%
1032	-0,046	0,001	1,1	0,1%	1009	-0,062	0,002	1,9	0,1%
1111	-0,692	0,239	265,7	15,9%	1066	0,813	0,330	352,7	22,7%
1180	-0,398	0,079	93,3	5,6%	1131	0,272	0,037	41,7	2,7%
1268	-0,292	0,043	54,1	3,2%	1236	-0,29	0,042	52	3,3%
1302	0,139	0,010	12,5	0,7%	1267	0,092	0,004	5,3	0,3%
1439	-0,121	0,007	10,5	0,6%	1382	0,01	0,000	0,1	0,0%
1544	0,914	0,418	644,7	38,5%	1454	0,511	0,131	189,5	12,2%
1667	-0,756	0,286	475,9	28,4%	1665	-0,979	0,479	798,2	51,4%
3249	-0,025	0,000	1	0,1%	3267	-0,03	0,000	1,5	0,1%
3265	-0,07	0,002	8,1	0,5%	3282	-0,054	0,001	4,8	0,3%
3283	0,091	0,004	13,5	0,8%	3299	0,086	0,004	12,3	0,8%
3O/ B3LYP									
Emission					Absorption				
$\tilde{\nu}$ [cm^{-1}]	B_i	S_i	λ_i [cm^{-1}]	%(λ)	$\tilde{\nu}$ [cm^{-1}]	B_i	S_i	λ_i [cm^{-1}]	%(λ)
71	-0,223	0,025	1,8	0,1%	68	-0,232	0,027	1,8	0,1%
285	0,295	0,044	12,4	0,7%	287	0,315	0,050	14,3	0,8%
425	0,434	0,094	40	2,4%	427	0,399	0,080	33,9	2,0%
895	0,047	0,001	1	0,1%	836	0,198	0,020	16,4	0,9%
905	-0,174	0,015	13,7	0,8%	891	-0,156	0,012	10,8	0,6%
972	-0,873	0,381	370,8	21,9%	921	-0,901	0,406	373,6	21,6%
1027	-0,1	0,005	5,1	0,3%	1006	-0,001	0,000	0	0,0%
1037	-0,624	0,195	201,5	11,9%	1051	-0,524	0,137	144,5	8,3%
1099	-0,14	0,010	10,8	0,6%	1089	-0,044	0,001	1	0,1%
1104	-0,079	0,003	3,4	0,2%	1116	-0,032	0,001	0,6	0,0%
1189	0,173	0,015	17,9	1,1%	1166	0,131	0,009	10	0,6%
1247	0,041	0,001	1	0,1%	1212	0,114	0,006	7,9	0,5%
1304	-0,32	0,051	66,9	4,0%	1311	-0,263	0,035	45,5	2,6%
1374	0,015	0,000	0,2	0,0%	1363	0,206	0,021	28,9	1,7%

1432	-0,173	0,015	21,6	1,3%	1426	-0,087	0,004	5,4	0,3%
1469	-0,117	0,007	10,1	0,6%	1462	-0,234	0,027	40,2	2,3%
1556	-0,523	0,137	212,6	12,6%	1517	-0,214	0,023	34,8	2,0%
1647	0,92	0,423	697,5	41,3%	1685	-1,069	0,571	963,1	55,6%
3249	0,008	0,000	0,1	0,0%	3243	0,009	0,000	0,1	0,0%
3266	-0,016	0,000	0,4	0,0%	3265	-0,009	0,000	0,1	0,0%
3269	-0,024	0,000	0,9	0,1%	3269	-0,012	0,000	0,2	0,0%
3283	0,005	0,000	0	0,0%	3279	0,009	0,000	0,1	0,0%
4O/ B3LYP									
Emission					Absorption				
$\tilde{\nu}$ [cm^{-1}]	B_i	S_i	λ_i [cm^{-1}]	%(λ)	$\tilde{\nu}$ [cm^{-1}]	B_i	S_i	λ_i [cm^{-1}]	%(λ)
98	-0,055	0,002	0,1	0,0%	104	-0,04	0,001	0,1	0,0%
225	-0,247	0,031	6,9	0,5%	227	-0,269	0,036	8,2	0,6%
411	-0,467	0,109	44,8	3,3%	415	-0,444	0,099	41	2,8%
468	-0,009	0,000	0	0,0%	467	-0,005	0,000	0	0,0%
565	0,005	0,000	0	0,0%	573	0,008	0,000	0	0,0%
887	0,036	0,001	0,6	0,0%	861	-0,111	0,006	5,3	0,4%
897	-0,089	0,004	3,6	0,3%	890	-0,08	0,003	2,8	0,2%
961	0,298	0,044	42,6	3,1%	923	-0,171	0,015	13,4	0,9%
982	0,616	0,190	186,6	13,6%	942	0,737	0,272	255,8	17,5%
1017	0,425	0,090	91,9	6,7%	1015	0,056	0,002	1,6	0,1%
1019	0,456	0,104	105,9	7,7%	1036	0,487	0,119	122,8	8,4%
1091	0,11	0,006	6,6	0,5%	1081	0,035	0,001	0,7	0,0%
1121	-0,005	0,000	0	0,0%	1120	-0,025	0,000	0,4	0,0%
1181	0,101	0,005	6	0,4%	1170	0,098	0,005	5,6	0,4%
1225	0,03	0,000	0,6	0,0%	1194	0,002	0,000	0	0,0%
1239	0,013	0,000	0,1	0,0%	1231	0,022	0,000	0,3	0,0%
1320	0,361	0,065	86,1	6,3%	1338	0,046	0,001	1,4	0,1%
1339	-0,042	0,001	1,2	0,1%	1350	-0,299	0,045	60,5	4,1%
1398	0,031	0,000	0,7	0,1%	1400	0,154	0,012	16,7	1,1%
1439	0,159	0,013	18,2	1,3%	1438	0,147	0,011	15,6	1,1%
1496	-0,071	0,003	3,8	0,3%	1472	-0,201	0,020	29,6	2,0%
1566	-0,389	0,076	118,5	8,6%	1536	0,179	0,016	24,7	1,7%
1648	0,884	0,391	643,8	46,9%	1634	-0,069	0,002	3,9	0,3%
1686	-0,064	0,002	3,5	0,3%	1703	-0,998	0,498	847,5	58,1%
3249	0,007	0,000	0,1	0,0%	3248	0,008	0,000	0,1	0,0%
3259	-0,005	0,000	0	0,0%	3256	-0,003	0,000	0	0,0%
3269	0,009	0,000	0,1	0,0%	3266	0,007	0,000	0,1	0,0%
3272	-0,02	0,000	0,7	0,1%	3272	-0,009	0,000	0,1	0,0%
3286	-0,002	0,000	0	0,0%	3279	0,006	0,000	0,1	0,0%

Part 4.

2O/PBE0									
Emission					Absorption				
$\tilde{\nu}$ [cm^{-1}]	B_i	S_i	λ_i [cm^{-1}]	%(λ)	$\tilde{\nu}$ [cm^{-1}]	B_i	S_i	λ_i [cm^{-1}]	%(λ)
0	0	0	0	0,0%	0	0	0,000	0	0,0%
397	0,379	0,072	28,4	1,2%	392	0,39	0,076	29,9	1,3%
463	0,203	0,021	9,6	0,4%	465	0,124	0,008	3,6	0,2%
903	-0,319	0,051	45,9	1,9%	866	0,142	0,010	8,8	0,4%
965	1,042	0,543	523,3	22,2%	890	1,109	0,615	547,3	24,2%

1037	-0,666	0,222	229,7	9,7%	1050	0,655	0,215	225,4	10,0%
1129	-0,37	0,068	77,2	3,3%	1107	-0,262	0,034	38,1	1,7%
1204	0,312	0,049	58,6	2,5%	1154	-0,101	0,005	5,9	0,3%
1272	0,02	0,000	0,3	0,0%	1236	0,22	0,024	30	1,3%
1336	-0,3	0,045	60,2	2,6%	1341	-0,268	0,036	48,3	2,1%
1462	0,332	0,055	80,8	3,4%	1463	-0,357	0,064	93,3	4,1%
1567	-0,658	0,216	339,4	14,4%	1503	0,198	0,020	29,4	1,3%
1698	-1,03	0,530	900,5	38,2%	1709	-1,187	0,704	1203,5	53,1%
3269	0,016	0,000	0,4	0,0%	3261	0,01	0,000	0,2	0,0%
3286	-0,039	0,001	2,6	0,1%	3288	-0,011	0,000	0,2	0,0%
3O/ PBE0									
Emission					Absorption				
$\tilde{\nu}$ [cm^{-1}]	B_i	S_i	λ_i [cm^{-1}]	%(λ)	$\tilde{\nu}$ [cm^{-1}]	B_i	S_i	λ_i [cm^{-1}]	%(λ)
70	-0,239	0,029	2	0,1%	65	0,246	0,030	2	70
290	-0,321	0,052	14,9	0,8%	292	-0,349	0,061	17,8	290
427	-0,466	0,109	46,3	2,6%	429	-0,425	0,090	38,9	427
897	-0,093	0,004	3,9	0,2%	841	0,198	0,020	16,4	897
907	-0,142	0,010	9,2	0,5%	893	-0,168	0,014	12,7	907
985	-0,811	0,329	324,2	17,9%	936	-0,89	0,396	370,7	985
1035	-0,327	0,053	55,5	3,1%	1020	-0,016	0,000	0,1	1035
1041	-0,609	0,185	193	10,7%	1052	-0,534	0,143	150,3	1041
1113	-0,21	0,022	24,6	1,4%	1102	-0,058	0,002	1,9	1113
1137	0,002	0,000	0	0,0%	1144	-0,036	0,001	0,7	1137
1214	0,162	0,013	16	0,9%	1191	0,135	0,009	10,8	1214
1262	0,024	0,000	0,4	0,0%	1228	0,075	0,003	3,5	1262
1336	-0,34	0,058	77,1	4,3%	1346	-0,212	0,022	30,2	1336
1393	0,004	0,000	0	0,0%	1386	0,26	0,034	47	1393
1457	-0,155	0,012	17,4	1,0%	1444	-0,089	0,004	5,7	1457
1496	0,121	0,007	10,9	0,6%	1487	0,236	0,028	41,4	1496
1579	0,51	0,130	205,3	11,3%	1539	0,223	0,025	38,2	1579
1678	-0,981	0,481	807,1	44,6%	1722	1,114	0,620	1067,8	1678
3269	0,009	0,000	0,1	0,0%	3262	-0,008	0,000	0,1	3269
3286	0,016	0,000	0,4	0,0%	3283	-0,008	0,000	0,1	3286
3290	-0,024	0,000	1	0,1%	3291	-0,011	0,000	0,2	3290
3301	0,006	0,000	0,1	0,0%	3296	-0,008	0,000	0,1	3301
4O/ PBE0									
Emission					Absorption				
$\tilde{\nu}$ [cm^{-1}]	B_i	S_i	λ_i [cm^{-1}]	%(λ)	$\tilde{\nu}$ [cm^{-1}]	B_i	S_i	λ_i [cm^{-1}]	%(λ)
99	-0,462	0,107	10,6	0,8%	105	-0,478	0,114	12	0,9%
229	0,094	0,004	1	0,1%	229	0,083	0,003	0,8	0,1%
413	0,06	0,002	0,7	0,0%	418	0,098	0,005	2	0,2%
472	0,208	0,022	10,2	0,7%	459	0,204	0,021	9,6	0,7%
573	-0,026	0,000	0,2	0,0%	585	-0,027	0,000	0,2	0,0%
896	0,08	0,003	2,9	0,2%	864	0,223	0,025	21,5	1,7%
905	0,263	0,035	31,2	2,2%	893	-0,249	0,031	27,8	2,1%
980	0,677	0,229	224,9	16,0%	945	0,731	0,267	252,4	19,4%
1000	0,333	0,055	55,5	3,9%	980	0,271	0,037	36,1	2,8%
1037	0,443	0,098	101,6	7,2%	1025	0,106	0,006	5,7	0,4%
1040	-0,058	0,002	1,7	0,1%	1044	0,413	0,085	88,8	6,8%
1111	0,38	0,072	80,2	5,7%	1086	0,357	0,064	69,3	5,3%
1147	0,113	0,006	7,4	0,5%	1135	0,12	0,007	8,2	0,6%
1205	0,081	0,003	4	0,3%	1176	0,082	0,003	4	0,3%

1256	-0,066	0,002	2,7	0,2%	1215	-0,109	0,006	7,2	0,6%
1263	0,102	0,005	6,5	0,5%	1245	0,103	0,005	6,6	0,5%
1338	0,116	0,007	9	0,6%	1334	0,081	0,003	4,4	0,3%
1361	0,004	0,000	0	0,0%	1350	0,067	0,002	3	0,2%
1419	0,216	0,023	33,1	2,3%	1410	0,017	0,000	0,2	0,0%
1461	-0,237	0,028	40,9	2,9%	1445	-0,432	0,093	134,6	10,4%
1514	-0,512	0,131	198,6	14,1%	1466	-0,367	0,067	98,8	7,6%
1582	0,288	0,041	65,8	4,7%	1517	0,119	0,007	10,7	0,8%
1664	0,376	0,071	117,6	8,3%	1625	0,396	0,078	127,2	9,8%
1702	-0,684	0,234	398,3	28,3%	1701	-0,655	0,215	364,7	28,1%
3270	-0,012	0,000	0,2	0,0%	3265	0,008	0,000	0,1	0,0%
3278	-0,019	0,000	0,6	0,0%	3283	-0,008	0,000	0,1	0,0%
3286	0,022	0,000	0,8	0,1%	3290	0,012	0,000	0,2	0,0%
3290	-0,032	0,001	1,7	0,1%	3297	-0,032	0,001	1,7	0,1%
3301	-0,027	0,000	1,2	0,1%	3302	-0,022	0,000	0,8	0,1%

Part 5.

2O/MN15									
Emission					Absorption				
$\tilde{\nu}$ [cm^{-1}]	B_i	S_i	λ_i [cm^{-1}]	%(λ)	$\tilde{\nu}$ [cm^{-1}]	B_i	S_i	λ_i [cm^{-1}]	%(λ)
391	0,464	0,108	42,1	1,5%	388	0,455	0,103	40,1	1,5%
456	0,143	0,010	4,7	0,2%	461	0,068	0,002	1,1	0,0%
885	0,341	0,058	51,5	1,9%	854	0,387	0,075	63,9	2,4%
961	0,983	0,483	463,9	16,8%	890	1,055	0,556	495,2	18,6%
1024	0,738	0,272	278,8	10,1%	1041	0,629	0,198	206,2	7,7%
1126	0,387	0,075	84,5	3,1%	1107	0,299	0,045	49,6	1,9%
1206	0,307	0,047	56,8	2,1%	1165	0,085	0,004	4,3	0,2%
1268	0,067	0,002	2,9	0,1%	1233	0,256	0,033	40,5	1,5%
1338	0,310	0,048	64,5	2,3%	1347	0,307	0,047	63,6	2,4%
1465	0,347	0,060	88,5	3,2%	1458	0,386	0,074	108,5	4,1%
1567	0,765	0,293	458,7	16,6%	1499	0,219	0,024	35,9	1,3%
1704	1,170	0,684	1165,9	42,1%	1728	1,339	0,896	1548,6	58,1%
3288	0,002	0,000	0,0	0,0%	3282	0,052	0,001	4,5	0,2%
3303	0,024	0,000	1,0	0,0%	3303	0,030	0,000	1,4	0,1%
3323	0,051	0,001	4,4	0,2%	3315	0,019	0,000	0,6	0,0%
3O/ MN15									
Emission					Absorption				
$\tilde{\nu}$ [cm^{-1}]	B_i	S_i	λ_i [cm^{-1}]	%(λ)	$\tilde{\nu}$ [cm^{-1}]	B_i	S_i	λ_i [cm^{-1}]	%(λ)
67	0,264	0,035	2,3	0,1%	63	0,261	0,034	2,1	0,1%
286	0,414	0,086	24,5	1,1%	289	0,440	0,097	28,0	1,2%
419	0,501	0,125	52,5	2,3%	423	0,465	0,108	45,7	2,0%
882	0,151	0,011	10,0	0,4%	825	0,241	0,029	24,0	1,0%
890	0,073	0,003	2,4	0,1%	877	0,195	0,019	16,6	0,7%
979	0,702	0,246	241,3	10,7%	931	0,861	0,371	345,4	14,9%
1023	0,569	0,162	165,4	7,3%	1042	0,596	0,177	184,7	8,0%
1027	0,612	0,187	192,5	8,5%	1013	0,023	0,000	0,3	0,0%
1108	0,185	0,017	19,0	0,8%	1096	0,059	0,002	1,9	0,1%
1141	0,035	0,001	0,7	0,0%	1144	0,015	0,000	0,1	0,0%
1212	0,121	0,007	8,9	0,4%	1195	0,120	0,007	8,7	0,4%
1270	0,065	0,002	2,7	0,1%	1239	0,041	0,001	1,1	0,0%
1331	0,412	0,085	112,8	5,0%	1388	0,326	0,053	73,8	3,2%
1392	0,012	0,000	0,1	0,0%	1347	0,273	0,037	50,1	2,2%

1460	0,108	0,006	8,6	0,4%	1438	0,011	0,000	0,1	0,0%
1498	0,182	0,017	24,9	1,1%	1489	0,287	0,041	61,2	2,6%
1580	0,611	0,187	295,0	13,0%	1541	0,294	0,043	66,8	2,9%
1684	1,137	0,646	1088,0	48,1%	1726	1,275	0,813	1403,8	60,4%
3288	0,051	0,001	4,3	0,2%	3283	0,016	0,000	0,4	0,0%
3303	0,047	0,001	3,7	0,2%	3300	0,068	0,002	7,7	0,3%
3307	0,006	0,000	0,1	0,0%	3306	0,013	0,000	0,3	0,0%
3323	0,046	0,001	3,5	0,2%	3318	0,023	0,000	0,9	0,0%

4O/ MN15									
Emission					Absorption				
$\tilde{\nu}$ [cm^{-1}]	B_i	S_i	λ_i [cm^{-1}]	%(λ)	$\tilde{\nu}$ [cm^{-1}]	B_i	S_i	λ_i [cm^{-1}]	%(λ)
94	0,004	0,000	0,0	0,0%	92	0,000	0,000	0,0	0,0%
225	0,423	0,089	20,1	1,0%	227	0,437	0,096	21,8	1,0%
405	0,566	0,160	64,8	3,1%	409	0,546	0,149	60,9	2,7%
464	0,019	0,000	0,1	0,0%	461	0,017	0,000	0,1	0,0%
563	0,028	0,000	0,2	0,0%	574	0,034	0,001	0,3	0,0%
880	0,090	0,004	3,6	0,2%	856	0,143	0,010	8,7	0,4%
888	0,072	0,003	2,3	0,1%	882	0,120	0,007	6,3	0,3%
977	0,313	0,049	47,8	2,3%	950	0,626	0,196	186,3	8,4%
993	0,452	0,102	101,4	4,8%	938	0,449	0,101	94,6	4,3%
1024	0,673	0,227	232,2	11,0%	1043	0,570	0,162	169,5	7,6%
1026	0,546	0,149	153,1	7,3%	1025	0,182	0,017	16,9	0,8%
1107	0,139	0,010	10,8	0,5%	1097	0,036	0,001	0,7	0,0%
1149	0,025	0,000	0,4	0,0%	1149	0,051	0,001	1,5	0,1%
1202	0,062	0,002	2,3	0,1%	1192	0,117	0,007	8,2	0,4%
1252	0,071	0,002	3,1	0,1%	1236	0,034	0,001	0,7	0,0%
1272	0,087	0,004	4,8	0,2%	1257	0,001	0,000	0,0	0,0%
1330	0,419	0,088	116,6	5,5%	1373	0,446	0,099	136,6	6,1%
1368	0,029	0,000	0,6	0,0%	1361	0,013	0,000	0,1	0,0%
1422	0,039	0,001	1,1	0,1%	1420	0,220	0,024	34,4	1,5%
1464	0,082	0,003	4,9	0,2%	1448	0,024	0,000	0,4	0,0%
1517	0,146	0,011	16,2	0,8%	1486	0,232	0,027	39,9	1,8%
1585	0,527	0,139	220,1	10,4%	1555	0,244	0,030	46,3	2,1%
1670	1,144	0,654	1092,8	51,9%	1717	1,261	0,796	1365,8	61,4%
1709	0,003	0,000	0,0	0,0%	1646	0,059	0,002	2,9	0,1%
3289	0,032	0,000	1,6	0,1%	3285	0,036	0,001	2,1	0,1%
3294	0,017	0,000	0,5	0,0%	3289	0,032	0,001	1,7	0,1%
3303	0,028	0,000	1,3	0,1%	3301	0,042	0,001	2,9	0,1%
3307	0,051	0,001	4,3	0,2%	3305	0,084	0,003	11,6	0,5%
3323	0,015	0,000	0,4	0,0%	3320	0,032	0,001	1,7	0,1%

Part 6.

2O/BHLYP									
Emission					Absorption				
$\tilde{\nu}$ [cm^{-1}]	B_i	S_i	λ_i [cm^{-1}]	%(λ)	$\tilde{\nu}$ [cm^{-1}]	B_i	S_i	λ_i [cm^{-1}]	%(λ)
406	0,43	0,092	37,5	1,2%	405	0,426	0,091	36,7	1,2%
477	-0,184	0,017	8,1	0,3%	482	-0,077	0,003	1,4	0,0%
936	-0,333	0,055	52,0	1,7%	901	-0,26	0,034	30,5	1,0%
998	-1,06	0,562	560,5	17,9%	920	1,135	0,644	592,8	19,5%
1064	-0,767	0,294	313,1	10,0%	1088	0,727	0,264	287,3	9,5%
1163	-0,298	0,044	51,5	1,6%	1144	0,165	0,014	15,5	0,5%

1236	-0,382	0,073	90,4	2,9%	1193	-0,152	0,012	13,8	0,5%
1320	-0,026	0,000	0,4	0,0%	1284	-0,29	0,042	54,0	1,8%
1362	0,317	0,050	68,3	2,2%	1386	0,362	0,066	90,7	3,0%
1510	0,376	0,071	106,8	3,4%	1502	-0,394	0,078	116,3	3,8%
1618	0,793	0,314	509,2	16,3%	1550	-0,161	0,013	20,1	0,7%
1751	-1,228	0,754	1319,6	42,3%	1772	-1,416	1,003	1777,2	58,5%
3344	0,018	0,000	0,6	0,0%	3335	0,012	0,000	0,2	0,0%
3360	-0,048	0,001	3,8	0,1%	3363	-0,017	0,000	0,5	0,0%
3378	-0,021	0,000	0,8	0,0%	3375	-0,027	0,000	1,3	0,0%
3O/BHLYP									
Emission					Absorption				
$\tilde{\nu}$ [cm^{-1}]	B_i	S_i	λ_i [cm^{-1}]	%(λ)	$\tilde{\nu}$ [cm^{-1}]	B_i	S_i	λ_i [cm^{-1}]	%(λ)
73	0,153	0,012	0,9	0,0%	68	-0,169	0,014	1,0	0,0%
296	0,425	0,090	26,7	1,0%	300	0,454	0,103	30,9	1,1%
439	0,509	0,130	56,8	2,1%	444	0,449	0,101	44,8	1,6%
932	0,127	0,008	7,5	0,3%	870	-0,279	0,039	33,9	1,2%
940	-0,133	0,009	8,3	0,3%	927	-0,206	0,021	19,6	0,7%
1020	-0,818	0,335	341,6	12,9%	965	-0,95	0,451	435,6	16,0%
1063	0,457	0,104	111,1	4,2%	1052	0,037	0,001	0,7	0,0%
1071	0,756	0,286	306,4	11,5%	1090	0,635	0,202	219,5	8,1%
1146	0,154	0,012	13,6	0,5%	1141	0,045	0,001	1,1	0,0%
1155	0,107	0,006	6,6	0,2%	1167	-0,069	0,002	2,8	0,1%
1248	-0,2	0,020	24,9	0,9%	1228	0,168	0,014	17,3	0,6%
1305	-0,025	0,000	0,4	0,0%	1270	-0,051	0,001	1,7	0,1%
1355	0,377	0,071	96,1	3,6%	1377	0,341	0,058	80,3	3,0%
1436	-0,022	0,000	0,3	0,0%	1424	-0,289	0,042	59,3	2,2%
1503	-0,129	0,008	12,5	0,5%	1483	0,008	0,000	0,0	0,0%
1543	0,216	0,023	35,9	1,4%	1538	0,308	0,047	73,1	2,7%
1632	-0,68	0,231	377,4	14,2%	1593	-0,288	0,041	66,2	2,4%
1731	-1,19	0,708	1224,8	46,1%	1767	-1,358	0,922	1630,4	60,0%
3344	-0,011	0,000	0,2	0,0%	3336	-0,008	0,000	0,1	0,0%
3361	-0,02	0,000	0,7	0,0%	3357	-0,012	0,000	0,2	0,0%
3364	-0,03	0,000	1,5	0,1%	3365	-0,01	0,000	0,2	0,0%
3378	0,005	0,000	0,0	0,0%	3374	0,007	0,000	0,1	0,0%
4O/BHLYP									
Emission					Absorption				
$\tilde{\nu}$ [cm^{-1}]	B_i	S_i	λ_i [cm^{-1}]	%(λ)	$\tilde{\nu}$ [cm^{-1}]	B_i	S_i	λ_i [cm^{-1}]	%(λ)
102	-0,05	0,001	0,1	0,0%	101	-0,03	0,000	0,0	0,0%
233	-0,453	0,103	24,0	1,0%	236	-0,483	0,117	27,6	1,1%
425	-0,591	0,175	74,1	3,0%	429	-0,546	0,149	64,0	2,5%
485	-0,026	0,000	0,2	0,0%	483	-0,001	0,000	0,0	0,0%
590	0,017	0,000	0,1	0,0%	602	0,027	0,000	0,2	0,0%
931	0,088	0,004	3,6	0,1%	903	0,197	0,019	17,6	0,7%
939	-0,08	0,003	3,0	0,1%	933	0,111	0,006	5,7	0,2%
1013	-0,279	0,039	39,4	1,6%	969	-0,441	0,097	94,3	3,7%
1034	-0,613	0,188	194,4	7,9%	986	-0,741	0,275	270,5	10,5%
1065	-0,473	0,112	118,9	4,8%	1067	0,142	0,010	10,7	0,4%
1070	0,778	0,303	323,8	13,1%	1096	-0,641	0,205	225,3	8,8%
1150	0,071	0,003	2,9	0,1%	1139	0,051	0,001	1,5	0,1%
1167	0,004	0,000	0,0	0,0%	1167	0,065	0,002	2,5	0,1%
1242	0,134	0,009	11,1	0,4%	1232	0,124	0,008	9,5	0,4%
1295	0,049	0,001	1,5	0,1%	1267	0,005	0,000	0,0	0,0%

1312	0,005	0,000	0,0	0,0%	1303	0,004	0,000	0,0	0,0%
1355	0,422	0,089	120,8	4,9%	1397	-0,268	0,036	50,2	2,0%
1393	0,021	0,000	0,3	0,0%	1399	0,404	0,082	114,3	4,5%
1464	-0,008	0,000	0,0	0,0%	1467	-0,207	0,021	31,5	1,2%
1507	-0,116	0,007	10,2	0,4%	1494	-0,048	0,001	1,7	0,1%
1563	0,18	0,016	25,5	1,0%	1533	0,282	0,040	60,8	2,4%
1637	0,596	0,178	291,2	11,8%	1606	0,271	0,037	58,8	2,3%
1717	-1,196	0,715	1226,9	49,6%	1683	-0,024	0,000	0,5	0,0%
1757	0,024	0,000	0,5	0,0%	1757	1,315	0,865	1518,7	59,2%
3344	0,007	0,000	0,1	0,0%	3338	-0,006	0,000	0,1	0,0%
3353	-0,01	0,000	0,2	0,0%	3348	-0,005	0,000	0,0	0,0%
3361	0,011	0,000	0,2	0,0%	3358	0,011	0,000	0,2	0,0%
3365	0,028	0,000	1,3	0,1%	3363	0,008	0,000	0,1	0,0%
3379	0,002	0,000	0,0	0,0%	3374	0,004	0,000	0,0	0,0%

Part 7.

2O/CAM-B3LYP									
Emission					Absorption				
$\tilde{\nu}$ [cm^{-1}]	B_i	S_i	λ_i [cm^{-1}]	%(λ)	$\tilde{\nu}$ [cm^{-1}]	B_i	S_i	λ_i [cm^{-1}]	%(λ)
397	0,45	0,101	40,2	1,3%	396	0,448	0,100	39,7	1,4%
465	-0,19	0,018	8,4	0,3%	469	-0,085	0,004	1,7	0,1%
913	-0,327	0,053	48,7	1,6%	876	-0,091	0,004	3,6	0,1%
972	-1,074	0,577	560,9	18,6%	895	1,165	0,679	606,9	20,7%
1039	0,729	0,266	276,0	9,2%	1057	0,694	0,241	254,4	8,7%
1133	-0,283	0,040	45,2	1,5%	1115	0,176	0,015	17,3	0,6%
1202	-0,385	0,074	88,9	2,9%	1157	0,142	0,010	11,7	0,4%
1283	0,027	0,000	0,5	0,0%	1245	0,282	0,040	49,6	1,7%
1331	0,302	0,046	60,7	2,0%	1348	0,336	0,056	76,1	2,6%
1468	0,359	0,064	94,4	3,1%	1470	0,393	0,077	113,7	3,9%
1580	0,763	0,291	460,3	15,3%	1510	0,184	0,017	25,7	0,9%
1716	-1,243	0,773	1325,8	44,0%	1740	-1,41	0,994	1729,7	59,0%
3272	0,019	0,000	0,6	0,0%	3263	0,012	0,000	0,2	0,0%
3288	-0,048	0,001	3,8	0,1%	3291	-0,015	0,000	0,4	0,0%
3305	-0,021	0,000	0,7	0,0%	3301	-0,028	0,000	1,3	0,0%
3O/ CAM-B3LYP									
Emission					Absorption				
$\tilde{\nu}$ [cm^{-1}]	B_i	S_i	λ_i [cm^{-1}]	%(λ)	$\tilde{\nu}$ [cm^{-1}]	B_i	S_i	λ_i [cm^{-1}]	%(λ)
71	-0,152	0,012	0,8	0,0%	66	-0,168	0,014	0,9	0,0%
289	0,444	0,099	28,5	1,1%	293	-0,475	0,113	33,0	1,2%
429	-0,524	0,137	58,8	2,3%	433	-0,466	0,109	46,9	1,8%
909	-0,124	0,008	7,0	0,3%	846	0,287	0,041	34,8	1,3%
917	0,128	0,008	7,5	0,3%	904	-0,186	0,017	15,7	0,6%
994	-0,827	0,342	339,7	13,2%	940	-0,949	0,450	423,5	16,0%
1037	0,324	0,052	54,5	2,1%	1024	0,011	0,000	0,1	0,0%
1044	-0,782	0,306	318,8	12,3%	1060	-0,607	0,184	195,2	7,4%
1118	-0,175	0,015	17,1	0,7%	1111	-0,027	0,000	0,4	0,0%
1128	0,039	0,001	0,9	0,0%	1141	-0,046	0,001	1,2	0,0%
1214	0,197	0,019	23,6	0,9%	1194	0,157	0,012	14,7	0,6%
1270	-0,029	0,000	0,5	0,0%	1236	-0,057	0,002	2,0	0,1%
1326	0,376	0,071	93,9	3,6%	1342	0,316	0,050	66,8	2,5%
1399	0,004	0,000	0,0	0,0%	1391	0,276	0,038	53,0	2,0%
1463	-0,151	0,011	16,6	0,6%	1448	-0,031	0,000	0,7	0,0%

1507	-0,204	0,021	31,4	1,2%	1505	-0,322	0,052	78,2	3,0%
1595	-0,636	0,202	323,1	12,5%	1555	0,295	0,044	67,7	2,6%
1698	-1,217	0,741	1257,7	48,7%	1734	-1,36	0,925	1605,2	60,8%
3273	0,011	0,000	0,2	0,0%	3265	0,009	0,000	0,1	0,0%
3288	0,02	0,000	0,7	0,0%	3286	0,012	0,000	0,2	0,0%
3292	-0,03	0,000	1,5	0,1%	3293	-0,01	0,000	0,2	0,0%
3305	0,005	0,000	0,0	0,0%	3300	-0,007	0,000	0,1	0,0%
4O/ CAM-B3LYP									
Emission					Absorption				
$\tilde{\nu}$ [cm^{-1}]	B_i	S_i	λ_i [cm^{-1}]	%(λ)	$\tilde{\nu}$ [cm^{-1}]	B_i	S_i	λ_i [cm^{-1}]	%(λ)
100	0,047	0,001	0,1	0,0%	98	0,027	0,000	0,0	0,0%
228	0,474	0,112	25,6	1,1%	230	0,506	0,128	29,5	1,2%
415	-0,605	0,183	75,9	3,1%	419	-0,561	0,157	65,9	2,6%
474	-0,029	0,000	0,2	0,0%	472	-0,003	0,000	0,0	0,0%
574	0,02	0,000	0,1	0,0%	586	0,03	0,000	0,3	0,0%
907	-0,088	0,004	3,5	0,1%	880	-0,199	0,020	17,4	0,7%
915	-0,074	0,003	2,5	0,1%	910	-0,101	0,005	4,7	0,2%
986	-0,256	0,033	32,4	1,3%	941	-0,407	0,083	78,1	3,1%
1008	-0,636	0,202	204,1	8,4%	961	-0,755	0,285	273,9	11,0%
1039	-0,373	0,070	72,1	3,0%	1039	-0,106	0,006	5,9	0,2%
1043	-0,78	0,304	317,7	13,1%	1065	-0,615	0,189	201,3	8,0%
1118	-0,065	0,002	2,4	0,1%	1109	-0,044	0,001	1,1	0,0%
1140	-0,009	0,000	0,0	0,0%	1140	0,05	0,001	1,4	0,1%
1208	0,13	0,008	10,2	0,4%	1198	-0,119	0,007	8,5	0,3%
1260	0,056	0,002	2,0	0,1%	1230	0,003	0,000	0,0	0,0%
1274	0,005	0,000	0,0	0,0%	1266	0,004	0,000	0,0	0,0%
1327	0,421	0,089	117,7	4,9%	1360	-0,162	0,013	17,8	0,7%
1359	0,025	0,000	0,4	0,0%	1364	0,423	0,089	121,9	4,9%
1425	0,026	0,000	0,5	0,0%	1432	0,202	0,020	29,1	1,2%
1466	-0,134	0,009	13,2	0,5%	1457	-0,091	0,004	6,1	0,2%
1527	0,174	0,015	23,1	1,0%	1499	-0,313	0,049	73,5	2,9%
1600	0,54	0,146	232,9	9,6%	1568	0,265	0,035	55,0	2,2%
1686	1,232	0,759	1279,6	52,9%	1651	-0,039	0,001	1,3	0,1%
1721	-0,035	0,001	1,0	0,0%	1725	-1,322	0,874	1507,6	60,3%
3273	0,007	0,000	0,1	0,0%	3267	0,006	0,000	0,1	0,0%
3281	-0,011	0,000	0,2	0,0%	3276	-0,005	0,000	0,0	0,0%
3288	-0,01	0,000	0,2	0,0%	3286	-0,011	0,000	0,2	0,0%
3293	0,028	0,000	1,3	0,1%	3291	0,008	0,000	0,1	0,0%
3305	0,002	0,000	0,0	0,0%	3301	0,004	0,000	0,0	0,0%

Part 8.

2O/tuned-CAM-B3LYP									
Emission					Absorption				
$\tilde{\nu}$ [cm^{-1}]	B_i	S_i	λ_i [cm^{-1}]	%(λ)	$\tilde{\nu}$ [cm^{-1}]	B_i	S_i	λ_i [cm^{-1}]	%(λ)
390	-0,444	0,099	38,5	1,3%	396	-0,448	0,100	39,7	1,4%
454	-0,188	0,018	8,0	0,3%	469	-0,085	0,004	1,7	0,1%
891	0,319	0,051	45,3	1,6%	876	0,091	0,004	3,6	0,1%
945	1,076	0,579	546,8	19,1%	895	1,165	0,679	606,9	20,7%
1015	0,706	0,249	253,0	8,9%	1057	0,694	0,241	254,4	8,7%
1101	-0,26	0,034	37,4	1,3%	1115	-0,176	0,015	17,3	0,6%
1169	-0,386	0,074	86,9	3,0%	1157	0,142	0,010	11,7	0,4%

1245	0,051	0,001	1,6	0,1%	1245	0,282	0,040	49,6	1,7%
1300	0,349	0,061	79,1	2,8%	1348	0,336	0,056	76,1	2,6%
1424	-0,326	0,053	75,6	2,6%	1470	0,393	0,077	113,7	3,9%
1534	0,697	0,243	372,1	13,0%	1510	0,184	0,017	25,7	0,9%
1672	1,25	0,781	1307,2	45,8%	1740	1,41	0,994	1729,7	59,0%
3219	0,02	0,000	0,6	0,0%	3263	0,012	0,000	0,2	0,0%
3233	-0,044	0,001	3,1	0,1%	3291	-0,015	0,000	0,4	0,0%
3252	0,019	0,000	0,6	0,0%	3301	0,028	0,000	1,3	0,0%
3O/ tuned-CAM-B3LYP									
Emission					Absorption				
$\tilde{\nu}$ [cm^{-1}]	B_i	S_i	λ_i [cm^{-1}]	%(λ)	$\tilde{\nu}$ [cm^{-1}]	B_i	S_i	λ_i [cm^{-1}]	%(λ)
68	0,295	0,044	3,0	0,2%	62	0,3	0,045	2,8	0,1%
284	-0,364	0,066	18,8	1,0%	286	-0,393	0,077	22,1	1,1%
419	-0,49	0,120	50,4	2,6%	422	-0,447	0,100	42,1	2,1%
885	-0,083	0,003	3,1	0,2%	824	0,248	0,031	25,4	1,3%
895	0,146	0,011	9,5	0,5%	882	0,157	0,012	10,9	0,5%
966	-0,852	0,363	350,5	17,9%	915	-0,929	0,432	395,1	19,8%
1011	0,019	0,000	0,2	0,0%	994	0,018	0,000	0,2	0,0%
1017	0,715	0,256	260,0	13,3%	1030	0,533	0,142	146,1	7,3%
1088	-0,182	0,017	18,0	0,9%	1079	-0,021	0,000	0,2	0,0%
1102	-0,003	0,000	0,0	0,0%	1113	0,027	0,000	0,4	0,0%
1181	0,181	0,016	19,3	1,0%	1157	-0,136	0,009	10,8	0,5%
1231	-0,001	0,000	0,0	0,0%	1200	-0,087	0,004	4,5	0,2%
1300	-0,36	0,065	84,2	4,3%	1306	-0,256	0,033	42,9	2,2%
1360	-0,014	0,000	0,1	0,0%	1355	-0,242	0,029	39,5	2,0%
1420	0,144	0,010	14,7	0,8%	1412	0,093	0,004	6,1	0,3%
1464	-0,137	0,009	13,8	0,7%	1461	-0,277	0,038	56,0	2,8%
1548	-0,466	0,109	168,3	8,6%	1509	0,231	0,027	40,2	2,0%
1658	-1,064	0,566	939,1	48,0%	1701	-1,162	0,675	1148,1	57,6%
3219	0,01	0,000	0,2	0,0%	3211	-0,007	0,000	0,1	0,0%
3233	0,017	0,000	0,5	0,0%	3230	0,009	0,000	0,1	0,0%
3237	0,026	0,000	1,1	0,1%	3237	0,012	0,000	0,2	0,0%
3252	0,003	0,000	0,0	0,0%	3247	0,004	0,000	0,0	0,0%
4O/ tuned-CAM-B3LYP									
Emission					Absorption				
$\tilde{\nu}$ [cm^{-1}]	B_i	S_i	λ_i [cm^{-1}]	%(λ)	$\tilde{\nu}$ [cm^{-1}]	B_i	S_i	λ_i [cm^{-1}]	%(λ)
95	0,016	0,000	0,0	0,0%	93	0,032	0,001	0,0	0,0%
224	0,377	0,071	15,8	0,9%	225	-0,405	0,082	18,5	1,0%
405	0,56	0,157	63,5	3,5%	409	0,527	0,139	56,9	3,0%
464	-0,024	0,000	0,1	0,0%	462	-0,004	0,000	0,0	0,0%
561	-0,015	0,000	0,1	0,0%	570	0,022	0,000	0,1	0,0%
884	-0,063	0,002	1,7	0,1%	857	0,164	0,013	11,5	0,6%
894	0,081	0,003	2,9	0,2%	888	-0,094	0,004	3,9	0,2%
958	0,274	0,038	36,1	2,0%	915	-0,303	0,046	41,9	2,2%
979	-0,68	0,231	226,7	12,6%	935	-0,766	0,293	274,5	14,7%
1015	-0,219	0,024	24,4	1,4%	1012	-0,042	0,001	0,9	0,0%
1017	-0,672	0,226	229,5	12,8%	1035	-0,532	0,142	146,5	7,9%
1086	0,073	0,003	2,9	0,2%	1076	0,015	0,000	0,1	0,0%
1113	0,006	0,000	0,0	0,0%	1112	-0,035	0,001	0,7	0,0%
1175	0,119	0,007	8,4	0,5%	1165	-0,113	0,006	7,4	0,4%
1220	0,038	0,001	0,9	0,1%	1188	0,01	0,000	0,1	0,0%
1237	-0,011	0,000	0,1	0,0%	1226	-0,01	0,000	0,1	0,0%

1302	0,393	0,077	100,8	5,6%	1321	0,135	0,009	12,0	0,6%
1322	0,04	0,001	1,1	0,1%	1328	0,34	0,058	76,8	4,1%
1384	-0,034	0,001	0,8	0,0%	1390	-0,177	0,016	21,8	1,2%
1422	0,127	0,008	11,4	0,6%	1419	0,142	0,010	14,2	0,8%
1483	-0,112	0,006	9,3	0,5%	1458	-0,26	0,034	49,4	2,6%
1552	0,373	0,070	108,1	6,0%	1523	-0,198	0,020	30,0	1,6%
1648	1,072	0,575	946,8	52,8%	1629	0,04	0,001	1,3	0,1%
1675	0,008	0,000	0,1	0,0%	1696	1,138	0,648	1097,2	58,8%
3219	-0,007	0,000	0,1	0,0%	3213	0,006	0,000	0,1	0,0%
3226	-0,009	0,000	0,1	0,0%	3221	-0,005	0,000	0,0	0,0%
3234	-0,009	0,000	0,1	0,0%	3231	-0,01	0,000	0,2	0,0%
3237	0,023	0,000	0,8	0,0%	3235	0,009	0,000	0,1	0,0%
3252	0,001	0,000	0,0	0,0%	3248	0,002	0,000	0,0	0,0%

Part 9.

2O/ωB97X									
Emission					Absorption				
$\tilde{\nu}$ [cm^{-1}]	B_i	S_i	λ_i [cm^{-1}]	%(λ)	$\tilde{\nu}$ [cm^{-1}]	B_i	S_i	λ_i [cm^{-1}]	%(λ)
396	0,509	0,130	51,3	1,5%	396	0,505	0,128	50,5	1,5%
464	0,195	0,019	8,8	0,3%	470	0,069	0,002	1,1	0,0%
908	-0,33	0,054	49,3	1,4%	876	-0,25	0,031	27,4	0,8%
973	-1,044	0,545	530,7	15,6%	897	-1,148	0,659	591,3	17,8%
1037	0,78	0,304	315,4	9,3%	1055	0,703	0,247	260,5	7,9%
1135	-0,278	0,039	44,0	1,3%	1117	0,193	0,019	20,8	0,6%
1207	-0,383	0,073	88,6	2,6%	1166	-0,125	0,008	9,2	0,3%
1279	0,012	0,000	0,1	0,0%	1246	0,265	0,035	43,8	1,3%
1340	0,286	0,041	54,9	1,6%	1357	0,362	0,066	89,0	2,7%
1476	0,383	0,073	108,1	3,2%	1472	0,406	0,082	121,5	3,7%
1592	-0,806	0,325	517,1	15,2%	1518	0,199	0,020	30,0	0,9%
1733	-1,373	0,943	1633,5	47,9%	1756	-1,535	1,178	2068,6	62,4%
3282	0,025	0,000	1,0	0,0%	3274	-0,015	0,000	0,4	0,0%
3297	0,05	0,001	4,2	0,1%	3299	0,014	0,000	0,3	0,0%
3313	0,024	0,000	0,9	0,0%	3309	0,029	0,000	1,4	0,0%
3O/ ωB97X									
Emission					Absorption				
$\tilde{\nu}$ [cm^{-1}]	B_i	S_i	λ_i [cm^{-1}]	%(λ)	$\tilde{\nu}$ [cm^{-1}]	B_i	S_i	λ_i [cm^{-1}]	%(λ)
71	-0,114	0,006	0,5	0,0%	67	-0,136	0,009	0,6	0,0%
288	-0,512	0,131	37,8	1,2%	293	0,55	0,151	44,3	1,5%
427	0,571	0,163	69,6	2,3%	432	0,503	0,127	54,6	1,8%
904	-0,162	0,013	11,8	0,4%	842	-0,332	0,055	46,3	1,5%
914	0,086	0,004	3,4	0,1%	901	0,177	0,016	14,1	0,5%
995	0,764	0,292	290,1	9,6%	941	0,953	0,454	427,6	14,0%
1036	-0,42	0,088	91,3	3,0%	1024	-0,012	0,000	0,1	0,0%
1042	0,837	0,350	364,9	12,0%	1058	0,644	0,207	219,0	7,2%
1120	0,185	0,017	19,1	0,6%	1112	0,023	0,000	0,3	0,0%
1134	-0,004	0,000	0,0	0,0%	1146	0,042	0,001	1,0	0,0%
1219	-0,197	0,019	23,6	0,8%	1201	-0,159	0,013	15,1	0,5%
1272	0,07	0,002	3,1	0,1%	1243	-0,022	0,000	0,3	0,0%
1332	0,382	0,073	97,1	3,2%	1351	-0,321	0,052	69,6	2,3%
1404	-0,006	0,000	0,0	0,0%	1400	-0,3	0,045	62,9	2,1%
1472	-0,153	0,012	17,3	0,6%	1450	-0,023	0,000	0,4	0,0%
1522	-0,253	0,032	48,7	1,6%	1515	-0,365	0,067	100,9	3,3%

1608	0,678	0,230	369,5	12,2%	1566	-0,344	0,059	92,7	3,0%
1717	1,359	0,923	1584,9	52,2%	1739	-1,479	1,094	1901,8	62,3%
3282	-0,013	0,000	0,3	0,0%	3275	0,01	0,000	0,2	0,0%
3297	0,022	0,000	0,8	0,0%	3294	0,014	0,000	0,3	0,0%
3301	-0,033	0,001	1,8	0,1%	3302	-0,007	0,000	0,1	0,0%
3313	0,006	0,000	0,1	0,0%	3308	0,007	0,000	0,1	0,0%
4O/ ωB97X									
Emission					Absorption				
$\tilde{\nu}$ [cm^{-1}]	B_i	S_i	λ_i [cm^{-1}]	%(λ)	$\tilde{\nu}$ [cm^{-1}]	B_i	S_i	λ_i [cm^{-1}]	%(λ)
99	0,066	0,002	0,2	0,0%	98	0,043	0,001	0,1	0,0%
228	0,552	0,152	34,8	1,2%	231	0,592	0,175	40,4	1,4%
413	-0,657	0,216	89,3	3,1%	418	-0,605	0,183	76,6	2,6%
472	-0,042	0,001	0,4	0,0%	471	-0,01	0,000	0,0	0,0%
572	0,03	0,000	0,3	0,0%	585	0,045	0,001	0,6	0,0%
903	0,113	0,006	5,8	0,2%	877	0,23	0,026	23,2	0,8%
911	0,049	0,001	1,1	0,0%	906	0,081	0,003	3,0	0,1%
988	0,222	0,025	24,4	0,8%	941	-0,504	0,127	119,5	4,1%
1008	0,541	0,146	147,3	5,1%	963	0,707	0,250	240,7	8,3%
1038	0,329	0,054	56,2	1,9%	1038	-0,119	0,007	7,3	0,3%
1042	-0,912	0,416	433,4	15,0%	1062	0,661	0,218	232,1	8,0%
1119	0,063	0,002	2,2	0,1%	1110	0,035	0,001	0,7	0,0%
1146	0,017	0,000	0,2	0,0%	1146	-0,046	0,001	1,2	0,0%
1211	0,124	0,008	9,3	0,3%	1202	0,112	0,006	7,5	0,3%
1263	0,072	0,003	3,3	0,1%	1238	-0,019	0,000	0,2	0,0%
1273	-0,059	0,002	2,2	0,1%	1266	-0,016	0,000	0,2	0,0%
1332	-0,432	0,093	124,3	4,3%	1367	0,105	0,006	7,6	0,3%
1368	0,01	0,000	0,1	0,0%	1377	-0,482	0,116	159,7	5,5%
1433	-0,03	0,000	0,7	0,0%	1440	-0,234	0,027	39,4	1,4%
1475	0,131	0,009	12,7	0,4%	1461	-0,059	0,002	2,5	0,1%
1542	0,221	0,024	37,8	1,3%	1506	0,359	0,064	96,9	3,3%
1613	-0,558	0,156	251,5	8,7%	1578	0,297	0,044	69,8	2,4%
1707	-1,386	0,960	1639,2	56,8%	1648	-0,026	0,000	0,6	0,0%
1739	-0,098	0,005	8,4	0,3%	1729	-1,432	1,025	1773,0	61,1%
3282	0,008	0,000	0,1	0,0%	3277	-0,006	0,000	0,1	0,0%
3290	0,014	0,000	0,3	0,0%	3284	-0,006	0,000	0,1	0,0%
3297	0,011	0,000	0,2	0,0%	3294	0,011	0,000	0,2	0,0%
3302	0,032	0,001	1,6	0,1%	3300	0,008	0,000	0,1	0,0%
3313	0,002	0,000	0,0	0,0%	3309	-0,004	0,000	0,0	0,0%

Part 10.

2O/CC2									
Emission					Absorption				
$\tilde{\nu}$ [cm^{-1}]	B_i	S_i	λ_i [cm^{-1}]	%(λ)	$\tilde{\nu}$ [cm^{-1}]	B_i	S_i	λ_i [cm^{-1}]	%(λ)
388	0,457	0,104	41	1,6%	383,0	0,445	0,099	37,9	1,4%
451	0,140	0,010	4	0,2%	453,0	0,083	0,003	1,6	0,1%
876	-0,153	0,012	10	0,4%	816,0	-0,687	0,236	192,7	7,3%
930	1,193	0,712	662	25,4%	853,0	0,962	0,463	394,6	15,0%
1021	-0,612	0,187	191	7,3%	1020,0	0,622	0,193	197,1	7,5%
1093	0,187	0,017	19	0,7%	1070,0	0,096	0,005	4,9	0,2%
1174	0,445	0,099	116	4,4%	1106,0	0,154	0,012	13,1	0,5%
1243	-0,008	0,000	0	0,0%	1193,0	-0,271	0,037	43,8	1,7%

1333	0,222	0,025	33	1,3%	1308,0	0,216	0,023	30,4	1,2%
1431	-0,352	0,062	89	3,4%	1447,0	0,192	0,018	26,5	1,0%
1537	0,605	0,183	282	10,8%	1492,0	-0,430	0,092	137,7	5,2%
1687	-1,173	0,688	1159	44,4%	1776,0	-1,321	0,873	1548,2	58,9%
3289	0,028	0,000	1	0,0%	3271,0	0,022	0,000	0,8	0,0%
3305	-0,039	0,001	3	0,1%	3301,0	-0,017	0,000	0,5	0,0%
3324	0,010	0,000	0	0,0%	3310,0	0,014	0,000	0,3	0,0%
3O/ CC2									
Emission					Absorption				
$\tilde{\nu}$ [cm^{-1}]	B_i	S_i	λ_i [cm^{-1}]	%(λ)	$\tilde{\nu}$ [cm^{-1}]	B_i	S_i	λ_i [cm^{-1}]	%(λ)
66	0,014	0,000	0	0,0%	62,0	-0,004	0,000	0,0	0,0%
282	0,380	0,072	20	0,9%	284,0	0,410	0,084	23,9	1,0%
418	-0,521	0,136	57	2,6%	421,0	-0,477	0,114	47,9	2,1%
868	0,027	0,000	0	0,0%	807,0	0,174	0,015	12,3	0,5%
879	-0,110	0,006	5	0,2%	867,0	0,071	0,003	2,2	0,1%
948	-0,879	0,386	366	17,0%	895,0	-0,927	0,430	384,7	16,6%
1010	-0,177	0,016	16	0,7%	983,0	-0,068	0,002	2,3	0,1%
1021	0,649	0,211	215	10,0%	1030,0	-0,499	0,125	128,3	5,5%
1081	-0,078	0,003	3	0,2%	1068,0	-0,077	0,003	3,1	0,1%
1112	-0,042	0,001	1	0,0%	1117,0	0,005	0,000	0,0	0,0%
1184	-0,216	0,023	28	1,3%	1155,0	0,156	0,012	14,1	0,6%
1231	0,038	0,001	1	0,0%	1194,0	0,091	0,004	5,0	0,2%
1335	0,358	0,064	86	4,0%	1314,0	0,187	0,017	23,0	1,0%
1365	0,029	0,000	1	0,0%	1363,0	0,230	0,026	36,0	1,6%
1425	-0,183	0,017	24	1,1%	1435,0	0,199	0,020	28,5	1,2%
1472	0,135	0,009	14	0,6%	1494,0	0,002	0,000	0,0	0,0%
1545	-0,428	0,092	142	6,6%	1516,0	0,337	0,057	86,2	3,7%
1672	-1,183	0,700	1170	54,5%	1759,0	1,317	0,867	1524,1	65,6%
3288	-0,015	0,000	0	0,0%	3276,0	0,015	0,000	0,4	0,0%
3303	0,002	0,000	0	0,0%	3293,0	-0,006	0,000	0,1	0,0%
3304	-0,023	0,000	1	0,0%	3297,0	0,000	0,000	0,0	0,0%
3324	-0,003	0,000	0	0,0%	3312,0	-0,003	0,000	0,0	0,0%
4O/ CC2									
Emission					Absorption				
$\tilde{\nu}$ [cm^{-1}]	B_i	S_i	λ_i [cm^{-1}]	%(λ)	$\tilde{\nu}$ [cm^{-1}]	B_i	S_i	λ_i [cm^{-1}]	%(λ)
93	0,207	0,021	2	0,1%	92,0	0,185	0,017	1,6	0,1%
222	0,384	0,074	16	0,8%	223,0	0,418	0,087	19,5	0,9%
405	0,615	0,189	76	3,7%	408,0	0,581	0,169	68,8	3,1%
460	0,032	0,001	0	0,0%	458,0	0,009	0,000	0,0	0,0%
549	0,018	0,000	0	0,0%	556,0	0,024	0,000	0,2	0,0%
867	-0,029	0,000	0	0,0%	839,0	0,108	0,006	4,9	0,2%
878	-0,064	0,002	2	0,1%	872,0	0,078	0,003	2,7	0,1%
943	0,284	0,040	38	1,8%	888,0	0,338	0,057	50,7	2,3%
959	-0,718	0,258	247	12,1%	915,0	-0,748	0,280	255,8	11,4%
1017	-0,171	0,015	15	0,7%	1009,0	-0,017	0,000	0,1	0,0%
1021	-0,636	0,202	207	10,1%	1033,0	-0,506	0,128	132,3	5,9%
1078	0,000	0,000	0	0,0%	1069,0	0,099	0,005	5,2	0,2%
1114	0,019	0,000	0	0,0%	1112,0	0,026	0,000	0,4	0,0%
1177	-0,128	0,008	10	0,5%	1163,0	-0,107	0,006	6,7	0,3%
1220	-0,078	0,003	4	0,2%	1171,0	-0,054	0,001	1,7	0,1%
1234	0,011	0,000	0	0,0%	1220,0	0,009	0,000	0,0	0,0%
1337	0,423	0,089	119	5,8%	1319,0	0,151	0,011	15,1	0,7%

1342	-0,056	0,002	2	0,1%	1347,0	0,269	0,036	48,6	2,2%
1387	-0,055	0,002	2	0,1%	1392,0	-0,182	0,017	22,9	1,0%
1425	0,148	0,011	16	0,8%	1430,0	-0,162	0,013	18,7	0,8%
1487	-0,142	0,010	15	0,7%	1488,0	-0,274	0,038	55,7	2,5%
1547	-0,337	0,057	88	4,3%	1519,0	0,272	0,037	56,4	2,5%
1660	1,193	0,712	1183	57,7%	1660,0	0,355	0,063	104,7	4,7%
1683	0,087	0,004	6	0,3%	1751,0	1,249	0,780	1365,5	61,0%
3289	0,009	0,000	0	0,0%	3277,0	0,007	0,000	0,1	0,0%
3289	0,010	0,000	0	0,0%	3281,0	0,010	0,000	0,2	0,0%
3303	-0,008	0,000	0	0,0%	3294,0	-0,005	0,000	0,0	0,0%
3304	0,014	0,000	0	0,0%	3295,0	-0,001	0,000	0,0	0,0%
3324	-0,004	0,000	0	0,0%	3312,0	0,003	0,000	0,0	0,0%

Table S4. Vertical excitation energies for 2O calculated at the DFT/TDDFT/def2-TZVPP level of theory in vacuum, as well as in acetonitrile and 1,4-dioxane using the COSMO model of solvation (for MN15 the calculations were also done using the PCM model for comparative purposes).

	**Vertical excitation energy [eV]**			**Gas to Solvent shift [eV]**	
	**Vacuum**	**Acetonitrile**	**1,4-dioxane**	**Acetonitrile**	**1,4-dioxane**
**BP**	4,165	4,023	4,007	0,135	0,158
**TPSSh**	4,380	4,241	4,218	0,139	0,162
**B3LYP**	4,418	4,279	4,256	0,139	0,162
**PBE0**	4,541	4,401	4,378	0,140	0,163
**MN15**	4,676	4,554	4,519	0,122	0,156
**MN15 / PCM**	4,593	4,455	4,440	0,138	0,153
**BHLYP**	4,771	4,631	4,608	0,140	0,164
**CAM-B3LYP**	4,720	4,584	4,562	0,136	0,158
**Tuned-CAM-B3LYP**	4,456	4,319	4,297	0,137	0,159
**wB97X**	4,864	4,731	4,710	0,134	0,155

Figure S1. Schematic drawing of the molecular structure of bifuran (2O), tertfuran (3O), and quaterfuran (4O).

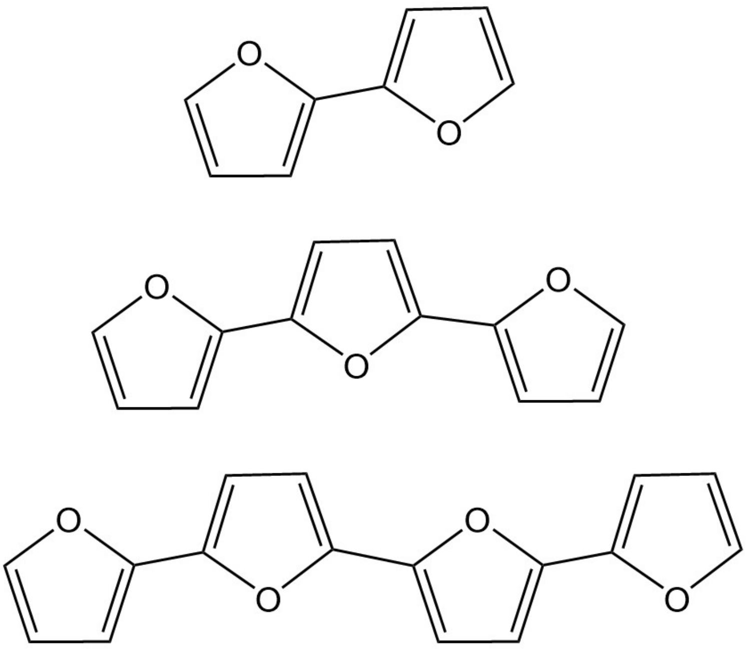

Figure S2. Deviations from the experimental values of the vertical absorption energies calculated using all the studied theoretical methods with the def2-TZVPP basis set.

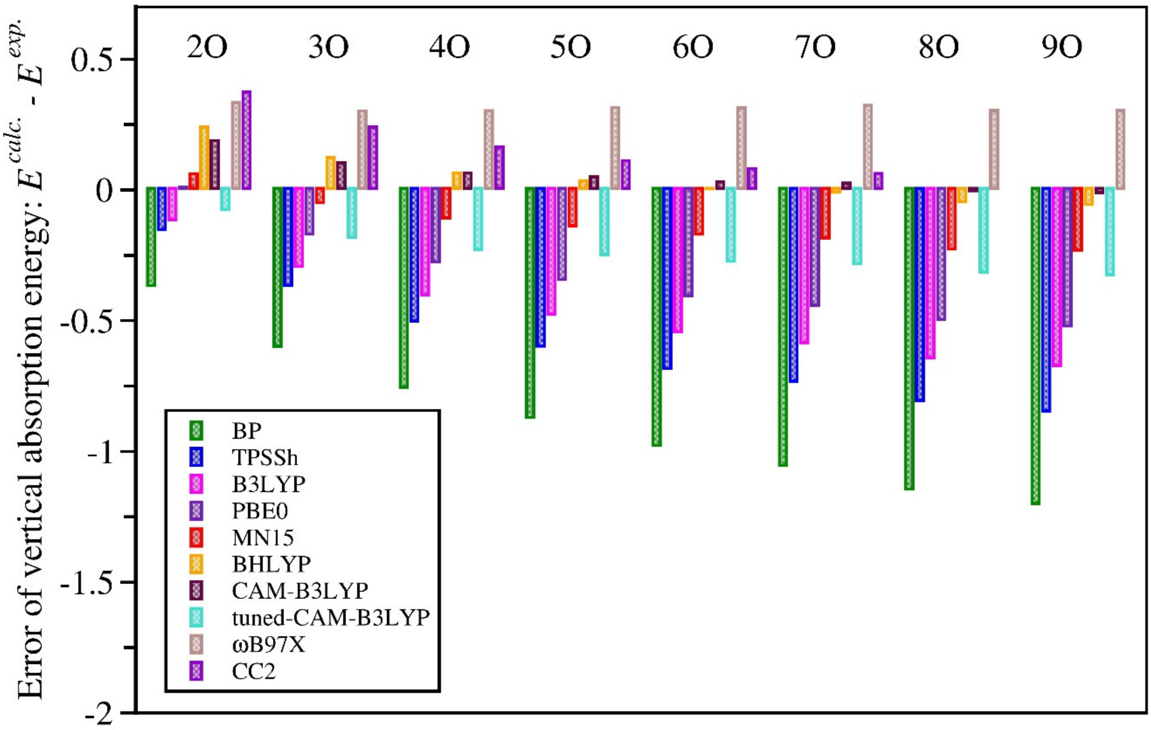

Figure S3. Deviations from the experimental values of the vertical emission energies calculated using all the studied theoretical methods with the def2-TZVPP basis set.

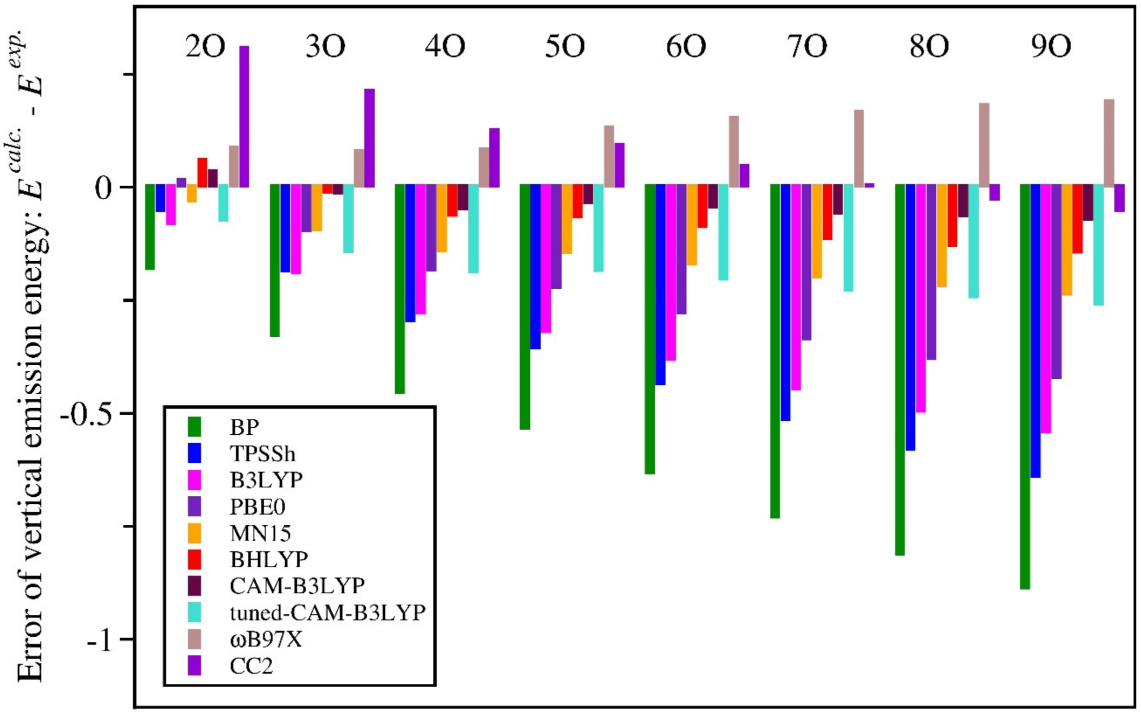

Figure S4. Deviations from the experimental values of the averaged reorganization energies: 0.5*(λ_{gr}+λ_{ex}) calculated using all the studied theoretical methods with the def2-TZVPP basis set. The experimental reference values have been estimated as 0.5*($E^{v.abs.}$-$E^{v.em.}$)

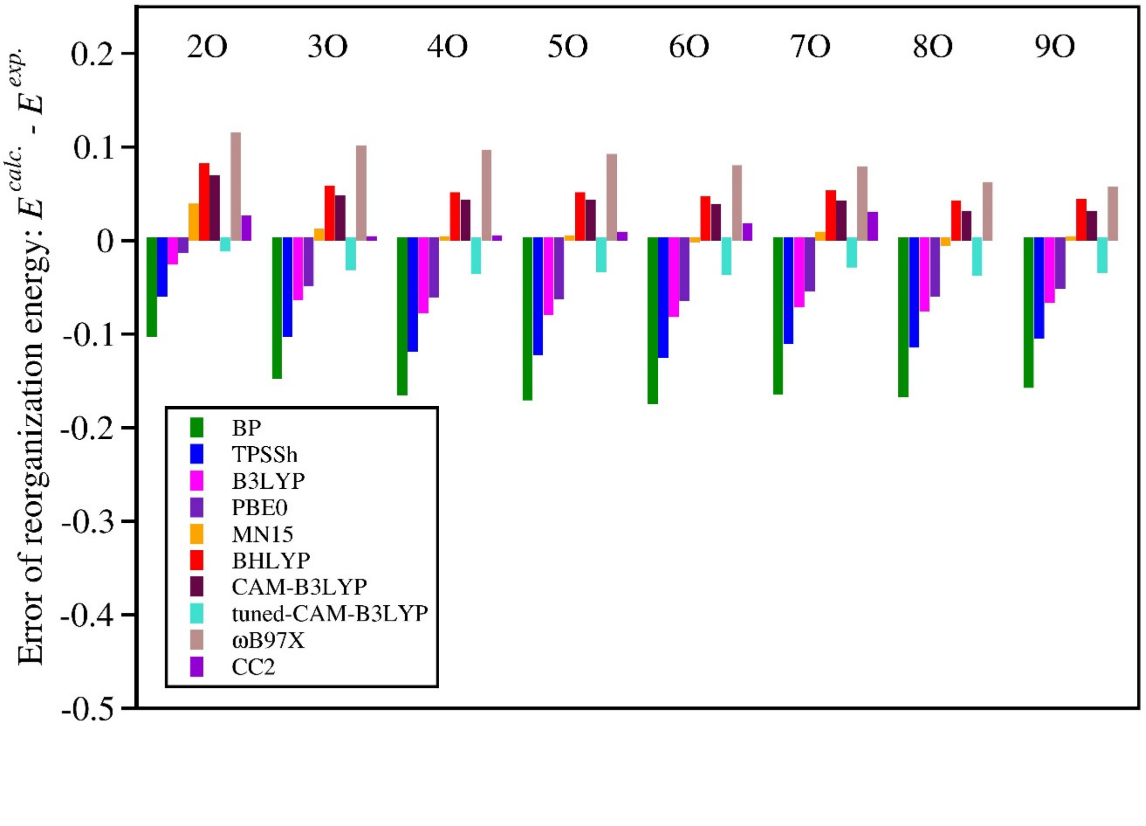

Figure S5. Comparison of the absorption and emission spectrum of 2O obtained at the CC2/def2-TZVPP level of theory with and without the Dushinsky rotation of the normal modes.

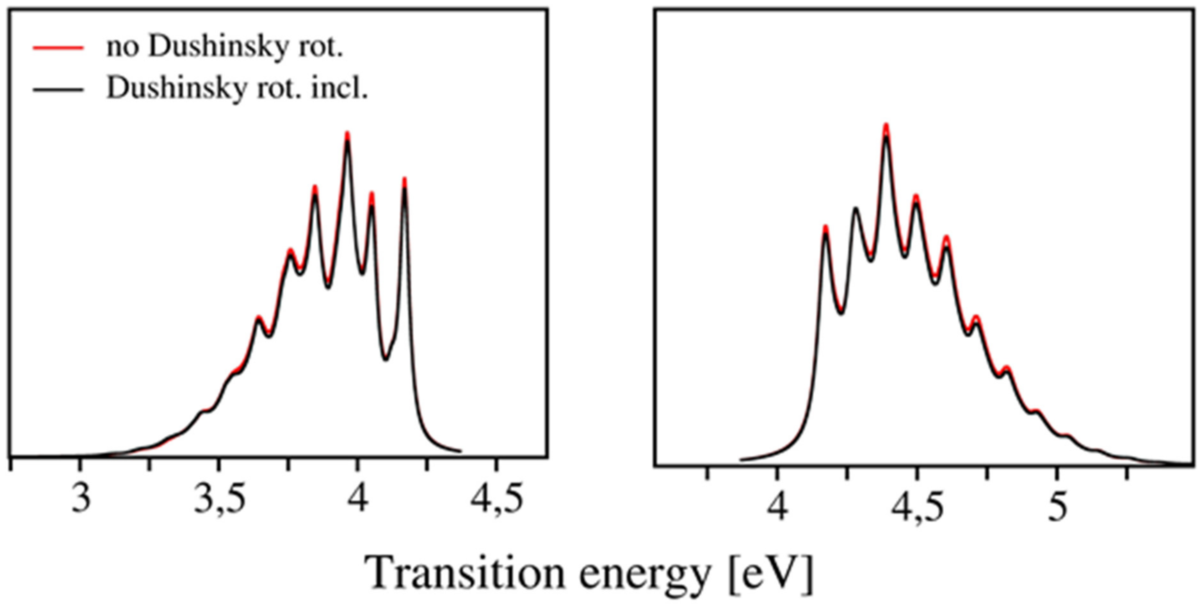

Supplement: Supplementary file 1 [file molecules-26-07163-s001.zip › molecules-1444235-supplementary.pdf]
